# Annotation of the Transcriptome from *Taenia pisiformis* and Its Comparative Analysis with Three Taeniidae Species

**DOI:** 10.1371/journal.pone.0032283

**Published:** 2012-04-13

**Authors:** Deying Yang, Yan Fu, Xuhang Wu, Yue Xie, Huaming Nie, Lin Chen, Xiang Nong, Xiaobin Gu, Shuxian Wang, Xuerong Peng, Ning Yan, Runhui Zhang, Wanpeng Zheng, Guangyou Yang

**Affiliations:** 1 Department of Parasitology, College of Veterinary Medicine, Sichuan Agricultural University, Ya'an, China; 2 Department of Chemistry, College of Life and Basic Science, Sichuan Agricultural University, Ya'an, China; New England Biolabs, United States of America

## Abstract

**Background:**

*Taenia pisiformis* is one of the most common intestinal tapeworms and can cause infections in canines. Adult *T. pisiformis* (canines as definitive hosts) and *Cysticercus pisiformis* (rabbits as intermediate hosts) cause significant health problems to the host and considerable socio-economic losses as a consequence. No complete genomic data regarding *T. pisiformis* are currently available in public databases. RNA-seq provides an effective approach to analyze the eukaryotic transcriptome to generate large functional gene datasets that can be used for further studies.

**Methodology/Principal Findings:**

In this study, 2.67 million sequencing clean reads and 72,957 unigenes were generated using the RNA-seq technique. Based on a sequence similarity search with known proteins, a total of 26,012 unigenes (no redundancy) were identified after quality control procedures via the alignment of four databases. Overall, 15,920 unigenes were mapped to 203 Kyoto Encyclopedia of Genes and Genomes (KEGG) pathways. Through analyzing the glycolysis/gluconeogenesis and axonal guidance pathways, we achieved an in-depth understanding of the biochemistry of *T. pisiformis*. Here, we selected four unigenes at random and obtained their full-length cDNA clones using RACE PCR. Functional distribution characteristics were gained through comparing four cestode species (72,957 unigenes of *T. pisiformis*, 30,700 ESTs of *T. solium*, 1,058 ESTs of Eg+Em [conserved ESTs between *Echinococcus granulosus* and *Echinococcus multilocularis*]), with the cluster of orthologous groups (COG) and gene ontology (GO) functional classification systems. Furthermore, the conserved common genes in these four cestode species were obtained and aligned by the KEGG database.

**Conclusion:**

This study provides an extensive transcriptome dataset obtained from the deep sequencing of *T. pisiformis* in a non-model whole genome. The identification of conserved genes may provide novel approaches for potential drug targets and vaccinations against cestode infections. Research can now accelerate into the functional genomics, immunity and gene expression profiles of cestode species.

## Introduction

More than 70 nominal species having been attributed to the ancient genus of *Taenia* [1], and approximately 42 valid species and three subspecies are currently recognized [2]. Taeniidae have two genera, *Echinococcus* and *Taenia*. *Echinococcus* comprises the tapeworm family, including *E. granulosus* and *E. multilocularis*, which causes morbidity in both humans and livestock [3], [4]. The genus *Taenia* contains six diverse species, including cestodes and metacestodes, which cause significant health problems in humans and socio-economic losses in the livestock industry. Species of *Taenia* parasitize in different hosts, including fish, reptiles and mammals. The adult stage of *T. pisiformis* (Cestoidea; Cyclophyllidea; Taeniidae; *Taenia*) parasitizes and matures in the small intestine of canids and felines [1], [5]–[7]. Lagomorphs are the most common intermediate hosts of *Cysticercus pisiformis* tapeworms, which usually live within the liver capsule, greater omentum and mesentery [8], [9]. Both *T. pisiformis* and *C. pisiformis* are widely distributed worldwide [7], [10]–[13]. Infections may occur when canines ingest the internal organs of rodents infected with *C. pisiformis* or when lagomorphs consume food polluted by the infected canines with the proglottids of *T. pisiformis*. *T. pisiformis* and *C. pisiformis* can cause significant health problems and even death [14], [15].

To date, the focus of research in Taeniidae has concentrated mostly on the search for functional genes, genetic variation and immune mechanisms between cestode and their hosts that could lead to medications [16]–[19]. The genome project for *T. solium* was established by a consortium of key laboratories at the National Autonomous University of Mexico in 2006 [20], but the genome-wide data has not yet been presented. Almeida et al. (2009) analyzed the transcriptome of *T. solium* cysticerci using open reading frames (ORESTES) [21]. A total of 1,520 high quality expressed sequence tags (ESTs) were generated from 20 ORESTES cDNA mini-libraries [21], and a genome-wide sequencing project for *E. multilocularis* is currently being carried out in a cooperation between the Parasite Sequencing Unit of the Wellcome Trust Sanger Centre and Brehm et al. [22]. The whole sequenced genomes of *E. granulosus*, *E. multilocularis*, and *Hymenolepis microstoma* have been presented on the National Center of Biotechnology Information (NCBI) website. The ESTs of *E. granulosus* and *E. multilocularis* are available on the Sanger database (http://www.sanger.ac.uk/), and the transcriptome of *E. multilocularis* is available through the sequence read archive (SRA) of the NCBI database (http://www.ncbi.nlm.nih.gov/sra/). Genome and transcriptome analyses provide powerful resources for the study of cestodes in terms of biochemistry, neurobiology, pharmacotherapy, vaccine development, and host-parasite interrelations. Thus, it is important to improve the genome-wide transcriptome exploration of *T. pisiformis*. However, research into *T. pisiformis* and *C. pisiformis* has been confined to their etiology, epidemiology and pharmacotherapy alone.

High-throughput sequencing (RNA-seq) provides an approach to analyze the *T. pisiformis* transcriptome in unparalleled depth and sensitivity. In this study, we performed the first *de novo* transcriptome analysis of *T. pisiformis*, which obtained high coverage and depth of gene content. Furthermore, the transcriptome of *T. pisiformis* was compared with the ESTs of *T. solium* and Eg+Em (conserved ESTs between *E. granulosus* and *E. multilocularis*) with the GO and COG functional classification systems. The intersection of common genes that were conserved between *T. pisiformis*, *T. solium* and Eg+Em was analyzed.

To the best of our knowledge, this is the first study of the characteristic transcriptome of *T. pisiformis* using Solexa/Illumina sequencing technology. The results are expected to determine antigens, give insights into the biochemistry and neurobiology of *T. pisiformis*, reveal the gene distributions characteristic of four Taeniidae species and accelerate research into functional genomics and host-parasite interrelations in cestodes.

## Results

### Annotation of adult T. pisiformis transcriptome data

#### 1) Coverage and quality of consensus sequences

**Figure 1:**
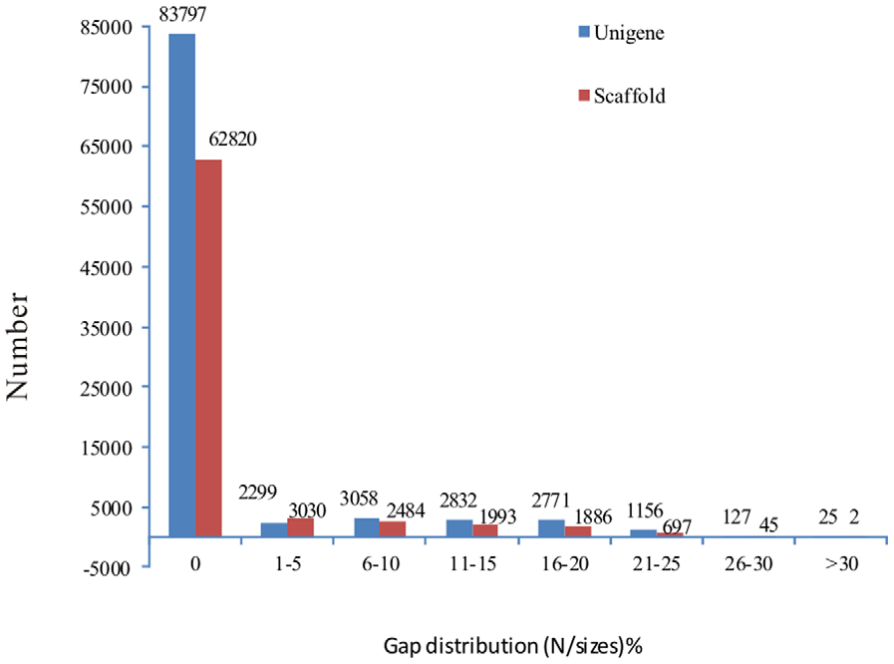
Gap distribution of assembled scaffolds and unigenes of adult *Taenia pisiformis* transcriptome. Gap distribution (N/size) %: gap percentage (N amount/sequence length) distribution. 87.23% of scaffold (83,797/96,065) and 86.11% of unigene (62,820/72,957) consensus sequences had no gap.

RNA was extracted from the whole organism of an adult *T. pisiformis*. Approximately 2.67 million cle*an reads and* 240 million total nucleotides were obtained using Solexa/Illumina RNA-seq deep sequencing. All clean reads were submitted to the SRA database at NCBI (accession no.: SRA037310). The SQ20 (sequencing quality 20) refers to a quality score that is greater than the proportion of bases over 20 (an error probability of 0.01). The SQ20 and GC percentage were 89.19% and 50.8%, respectively. The length percentage of contigs was highest within the range of 75–100 bp (66.5%), and scaffolds and unigenes were within 100–500 bp (84.62% and 79.76%, respectively). Overall, 72,957 non-redundant consensus sequences were obtained through the removal of partial overlapping sequences, with an average unigene length of 398 bp and an N50 of 462 bp. All unigenes were provided as Dataset S1. It was determined that 87.23% (83,797/96,065) scaffolds and 86.11% (62,820/72,957) unigene consensus sequences had no gap (Figure 1). Only 25 scaffolds and two unigenes exceeded the 3% gap (Figure 1). In order to evaluate the quality of assembly, unigenes were aligned with the clean reads of *T. pisiformis*. The results showed that 99.79% (*72,802/72,9*57) unigenes mapped to clean reads. The high mapped scores between unigenes and clean reads, and no gap percent in scaffolds and unigenes, suggested that the SOAPdenovo assembly was reliable.

RACE PCR was utilized to validate the quality of the adult *T. pisiformis* transcriptome. Specifically, four unigene consensus sequences were randomly selected as templates. As a result, the four antigens obtained the full-length cDNA clones using RACE PCR, including homologous antigen 18KD (GenBank: JN247398), cC1 (GenBank: JN247399), TP1 (GenBank: JN228964) and TPP2 (GenBank: JN228965).

#### 2) Functional annotation of unigenes in adult *T. pisiformis*

In order to detect known gene sequences in existing species, the RNA-seq data set of *T. pisiformis* was successively compared with the Nr, Swiss-prot, COG and KEGG databases successively, using the BLASTx program. A number of unigene consensus sequences unambiguously matched the previously annotated genes or overlapping annotated ORFS when aligned through the four respective databases (Table 1). And the respective annotations were presented in Dataset S2 (Nr), Dataset S3 (Swiss-prot), Dataset S4 (KEGG), and Dataset S5 (COG), respectively, via BLAST (basic local alignment on search tool).

**Table 1:** Summary of sequence assembly and annotations of the *Taenia pisiformis* transcriptome.

	Sequences (n)	Mean length (bp)	N50 (bp)	Annotations (n)	Functional classification
Clean reads (paired-end)	2.67 million	-	-	-	-
All assembled contigs	330, 316	154	169	-	-
All assembled scaffords	96,065	331	414	-	-
All assembled unigenes	72,957	398	462	-	-
Gene against Nr	25,701	-	-	201,908	-
GO for Nr protein hits	7,706	-	-	56,217	4,580 GO terms
Gene against Swiss-prot	19,564	-	-	148,048	-
Gene against COG	7,760	-	-	47,241	25 molecular families/98 categories
Gene against KEGG	15,920	-	-	134,829	203 KEGG pathways
CDS against four database	25,633	-	-	25,633	-
All annotated unigenes	26,012	-	-	-	-

N50 = median length of all non-redundant consensus sequences, mean = average length of all consensus sequences.

**Figure 2:**
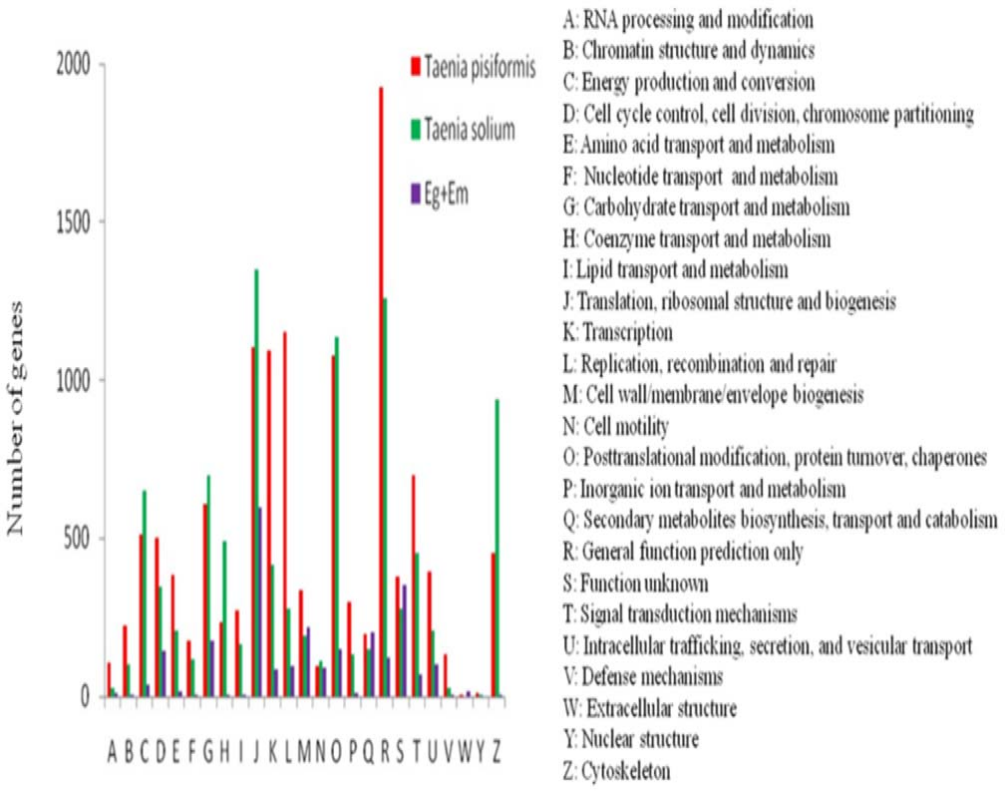
COG functional annotations of putative proteins among unigenes of four species in Taeniidae. Overall, 459 ESTs of Eg+Em (common genes in *Echinococcus granulosis* and *Echinococcus multilocularis*), 7,760 unigenes of *Taenia pisiformis*, and 6,852 ESTs of *Taenia solium* were classified into at least 25 molecular families.

After alignment through the COG database, the functions of 7,760 unigenes were classified into at least 25 molecular families and 98 functional-categories (**Figure 2**). Among these molecular families, R (general function prediction only) included only 7.04% (1,830/26,012) unigene**s**. The next commonest, L (replication, recombination and repair), J (translation, ribosomal structure and biogenesis), K (transcription) and O (post-translational modification, protein turnover, chaperones) had 4.30% (1,119), 4.02% (1,046), 4.00% (1,040) and 3.96% (1,029) unigenes, respectively. W (extracellular structures) had two unigenes. Apart from these molecular families, S (function unknown) had 0.47% (322) unigenes. Of the 98 functional-categories, the four largest categories also included ‘general function prediction’, ‘post-translational modification, protein turnover and chaperones’, ‘translation, ribosomal structure and biogenesis’ and ‘replication, recombination and repair’.

**Figure 3:**
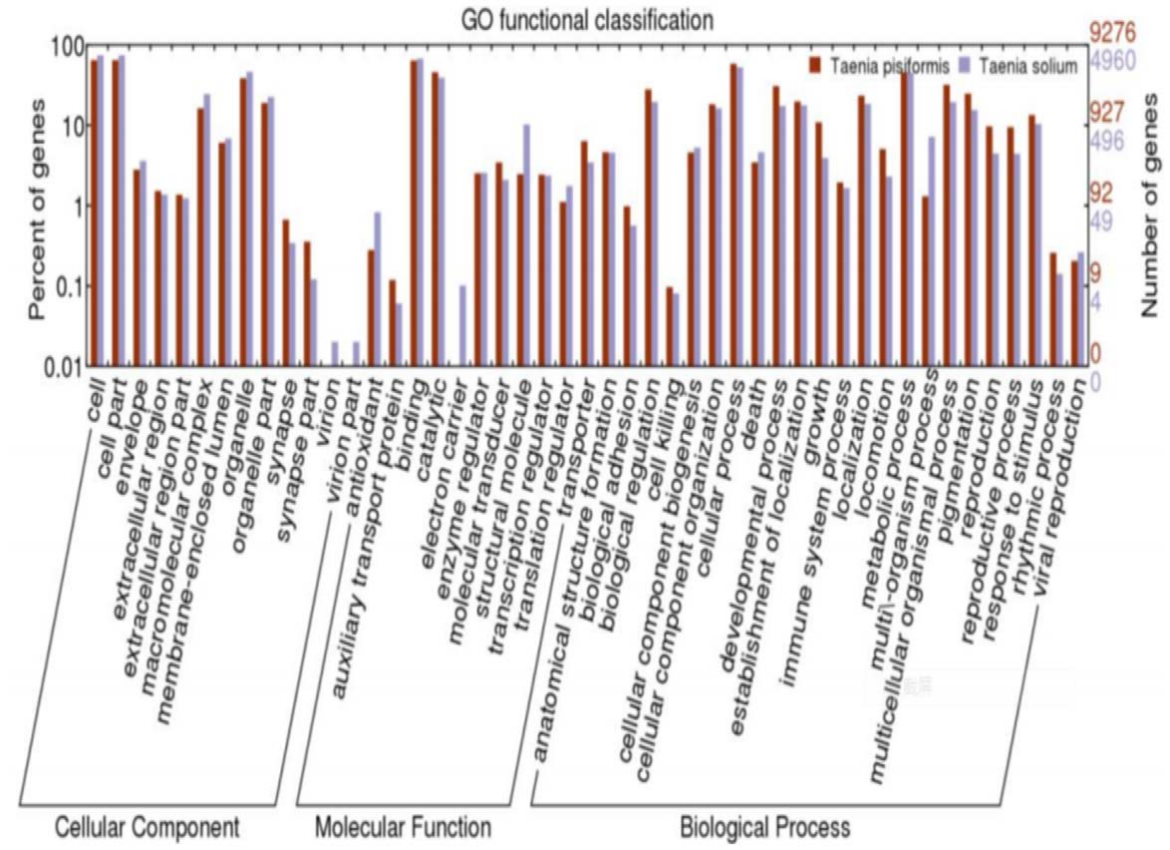
GO annotations of unigenes in *Taenia pisiformis* transcriptome and ESTs in *Taenia solium*. In total, 7,706 unigenes of *Taenia pisiformis* and 4,960 ESTs of *Taenia solium* obtained 88,471 GO annotations.

An analysis of GO function provided a GO functional classification annotation for differentially expressed genes, as well as an enrichment analysis for differentially expressed genes. Of the most significant BLASTx hits against the Nr known species dataset, a total of 7,706 unigenes were assigned GO term annotations using BLAST2GO (**Table 1**). In order to categorize the genome-wide transcriptome of *T. pisiformis* functionally, these GO terms were summarized into the three main GO categories and 47 sub-categories. GO has three functional categories: molecular function (including 23 sub-categories), cellular components (including 11 sub-categories) and biological process (including 13 sub-categories) (**Figure 3**). Biological process made up the majority of the GO annotations (29,831, 53.06% of the total), followed by molecular function (16,684, 29.68%) and cellular components (9,702, 17.26%).

Further biological process categories were prominently represented in ‘cellular process’, ‘metabolic process’ and ‘multicellular organismal process’, which indicated that some important cellular activities may occur in *T. pisiformis*. In addition, ‘immune system process’, ‘metabolic process’, and ‘response to stimulus’ related to the immune responses and immune defenses of *T. pisiformis*. In the molecular function category, ‘binding’ and ‘catalytic activity’ represented the most abundant classification. Accordingly, ‘cell’, ‘cell part’, and ‘organelle’ were represented in the cellular component category, while 1,615 annotations belonged to the extracellular region.

#### 3) KEGG pathway of unigene consensus sequences

**Figure 4:**
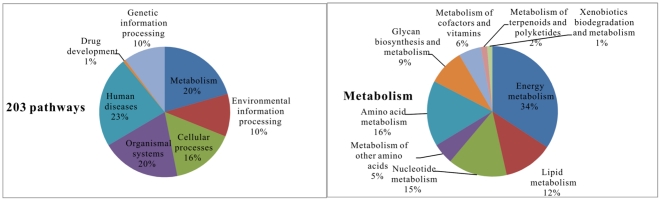
A total of 15,920 unigenes of adult *Taenia pisiformis* transcriptome were assigned to 203 KEGG pathways. The KEGG pathways embodied in metabolism, genetic information processing, environmental information processing, cellular processes, organismal systems, drug development and human diseases.

In order to identify the active biological pathways in *T. pisiformis*, a total of 15,920 unigenes were assigned to 203 KEGG pathways (Dataset S6) from the KEGG database. The KEGG pathways embodied metabolism (9 members), genetic information processing (16 members), environmental information processing (17 members), cellular processes (13 members), organismal systems (34 members), drug development (2 members) and human diseases (37 members) (Figure 4).

In order to gain further insights into the biochemistry and physiology of *T. pisiformis*, we selected the glycolysis/gluconeogenesis pathway of energy metabolism within the metabolism and axon guidance pathway (**Dataset S7**) of organismal systems for further analysis. The pathway of glycolysis/gluconeogenesis relates to energy generation, and 215 unigenes were related to this pathway that mapped to 32 enzyme genes. Three key enzymes in glycolysis/gluconeogenesis, hexokinase, phosphofructokinase and pyruvate kinase, were mapped by 21, 12, and 21 unigenes, respectively (**Table 2**). No unigenes mapped to pyruvic decarboxylase, malic dehydrogenase, and ADP-forming acetyl-COA synthetase (**Table 2**).

**Table 2:** The key genes of the glycolysis/gluconeogenesis and the axon guidance pathways for *Taenia pisiformis*.

Genes name	Ko	Definition	Disease
HK	K00844	hexokinase [EC:2.7.1.1]	Anemia due to disorders of glycolytic enzymes (H00664)
PK	K00873	pyruvate kinase [EC:2.7.1.40]	-
ALDO	K01623	fructose-bisphosphate aldolase, class I [EC:4.1.2.13]	Hereditary fructose intolerance (H00071)
E6.2.1.13	K01905	acetyl-CoA synthetase (ADP-forming) [EC:6.2.1.13]	-
E4.1.1.1, pdc	k01568	pyruvate decarboxylase [E4.1.1.1]	-
NTN1	K06843	netrin 1	-
EFNB	K05463	ephrin-B	Craniosynostosis (H00458)
SLIT1	K06838	Slit 1	-
SLIT2	K06839	Slit 2	-
SLIT3	K06850	Slit 3	-
SEMA4	K06521	Semaphoring 4	Cone-rod dystrophy and cone dystrophy ( H00481)Retinitis pigmentosa (RP)( H00527)
SEMA5	K06841	Semaphoring 5	Cri du chat syndrome (H00764)
SEMA6	K06842	Semaphoring 6	-
DCC	K06765	deleted in colorectal carcinoma	Gastric cancer (H00018), Colorectal cancer (H00020), Cancer of the anal canal (H00044)
UNC5	K07521	netrin receptor unc-5	-
EPHA1	K05102	Eph receptor A1 [EC:2.7.10.1]	-
EPHB1	K05110	Eph receptor B1 [EC:2.7.10.1]	-
ROBO1	K06753	roundabout, axon guidance receptor 1	-
ROBO2	K06754	roundabout, axon guidance receptor 2	-
ROBO3	K06755	roundabout, axon guidance receptor 3	-

The other important pathway was axon guidance, which is one of the most important organismal systems for neurotransmission. During the development of the nervous system, axons are guided along specific pathways by different classes of guidance cues within the extracellular environment. Attractive and repulsive guidance molecules play vital roles in axon path-finding through long-range or short-range modes. A total of 262 unigenes were mapped to this pathway. There are four highly conservative axon guidance molecular protein families, netrins (netrin 1), slits (slit1, slit2, and slit3), semaphorins (sema4D, sema5, and sema6), and ephrins (ephrin E) (**Table 2**). The receptors of netrin 1 contain DCC and UNC-5, which mediate axon repulsion (via Ca^2+^ concentration) and axon outgrowth, respectively. The receptors for slit1, slit2, and slit3 include Robo1, Robo2, and Robo3, which mediate axon attraction and axon repulsion. The common receptor of sema4D, sema5, and sema6 is plexin B, which mediates axonal repulsion. Eph A and Eph B are receptors for ephrin E, which mediate axonal attraction and axonal repulsion. Through analyzing the glycolysis/gluconeogenesis pathway and the signaling pathway for axonal guidance, we achieved a more in-depth understanding of the biochemistry of adult *T. pisiformis*.

#### 4) Alignment CDS of unigene consensus sequences

**Figure 5:**
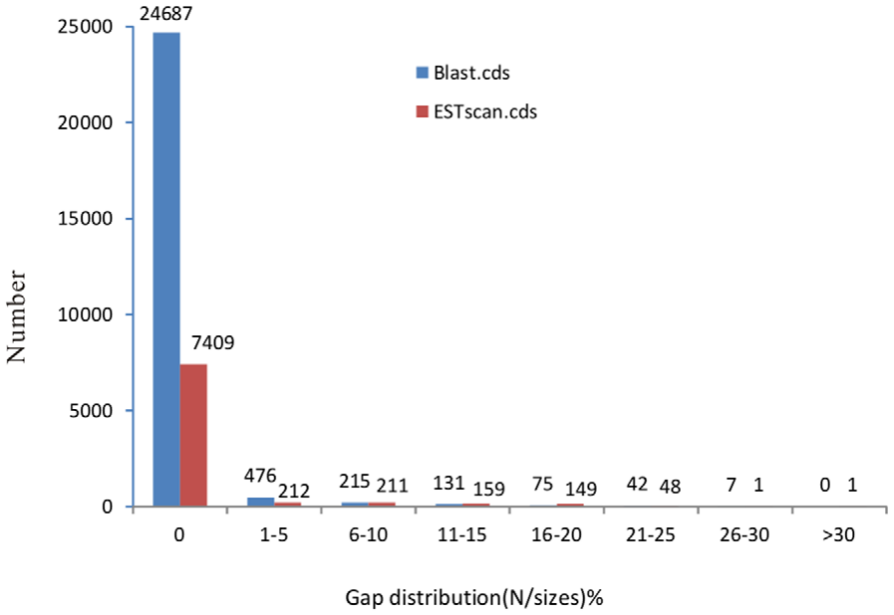
Gap distribution of assembled blast CDS and ESTscan CDS of adult *Taenia pisiformis* transcriptome. Gap distribution (N/size) %: gap percentage (N amount/sequence length) distribution. 97.09% (24887/25633) blast CDS and 90.46% (7409/8190) ESTScan CDS had no gap.

The results showed that among the annotated unigenes, 25,633 coding sequences (CDS) were obtained by the BLASTx algorithm, where one unigene corresponded to one CDS. In particular, 519 CDS perfectly matched the known genes from 10 other tapeworm species. However, the failed-hit unigenes were predicted by the ESTScan software and 8,190 CDS were obtained. The orientation of all CDS was 5′-3′. Furthermore, 97.09% (24,887/25,633) blast CDS and 90.46% (7,409/8,190) ESTScan CDS had no gap (**Figure 5**), which demonstrated the high quality of the assemblies.

Overall, those CDS belonged to different species, including parasites, mammals, aquatic animals and others. Twenty-seven CDS were aligned to the *T. pisiformis* genome, including mitochondrial genes and antigen genes, such as NADH dehydrogenase subunit 1, subunit 2, subunits 4 to 6, cytochrome b, cathepsin L-like protease, and cysteine protease. The tapeworm species, *T. solium*, *T. saginata*, *T. taeniaeformis*, *T. asiatica*, *T. hydatigena*, *T. ovis*, *E. granulosus*, *E. multilocularis*, *E. ortleppi*, and *M. corti*, showed a high similarity with *T. pisiformis*. Among these 10 tapeworm species, apart from normal physiological activities and the structure of their organs, a number of antigens that had been reported were aligned, particularly in *T. asiatica*, *T. solium* and *E. granulosus*. For example, TSES33, Tso31d, GP50, GP50c precursor, Tso22b, cC1, T24, cathepsin L-like cysteine proteinase, heat shock protein (HSP) and paramyosin had been reported in other cestode species, and were present in the CDS of unigene consensus sequences in *T. pisiformis*. Otherwise, there were some CDS of oncosphere proteins, such as Tso31a, Tso22b, antigen A, and Tm18, which could be used to protect intermediate host (rabbit) as vaccine antigen.

In addition, some CDS of coding antioxidants in eukaryotic cells were identified from the Nr, Swiss-prot, COG and KEGG databases, including thioredoxin peroxidase, glutathione peroxidase, peroxidase, superoxide dismutase, and glutathione reductase. Furthermore, the parasite species not only included cestode species, but also protozoa (*Leishmania infantum*), nematodes (such as *Brugia malayi*, *Caenorhabditis briggsae*, *C. elegans*, *Necator americanus*, *Ancylostoma caninum*, *and A. duodenale*) and trematodes (such as *Schistosoma mansoni*, *S. haematobium*, *S. japonicum*).

### Analysis of comparative transcripts among four Taeniidae species

#### 1) Common genes

In total, 10,983 ESTs of *E. granulosis* and 12,701 ESTs of *E. multilocularis* were downloaded from the databases found at ftp://ftp.sanger.ac.uk/pub/pathogens/Echinococcus/granulosis/ESTs/fasta.gz and ftp://ftp.sanger.ac.uk/pub/pathogens/Echinococcus/multilocularis/ESTs/fasta.gz, respectively. As described in the Methods section, 1,058 common ESTs (Eg+Em) were obtained. Then, the 1,058 ESTs of Eg+Em, 72,957 unigenes of *T. pisiformis*, and 30,700 ESTs (http://www.ncbi.nlm.nih.gov/nucest/?term=Taenia%20solium) were aligned using BLASTx. Six-hundred common genes (M) were found to be conserved between *T. pisiformis*, *T. solium*, *E. granulosis* and *E. multilocularis*.

#### 2) COG and GO functional classification of *T. pisiformis*, *T. solium* and Eg+Em

The 1,058 ESTs of Eg+Em, 72,957 unigenes of *T. pisiformis*, and 30,700 ESTs of *T. solium* were aligned with the COG and Nr database and categorized by the COG and GO functional classification systems, respectively. After alignment through the COG database, 459 ESTs of Eg+Em (Dataset S8), 7,760 unigenes of *T. pisiformis*, and 6,852 ESTs of *T. solium* unigenes (Dataset S9) were classified into at least 25 molecular families (Figure 2). Functional classifications concentrated on J (translation, ribosomal structure and biogenesis), O (second metabolites biosynthesis, transport and catabolism), R (general function prediction only), and S (function unknown) (Figure 2). Overall, 1,058 ESTs of Eg+Em could not be given a GO functional classification in the Nr database; 7,706 unigenes of *T. pisiformis* and 4,960 ESTs (Dataset S10) of *T. solium* obtained the GO annotations according to Nr database (Figure 3). Biological process made up the majority of the GO annotations (47,022, 53.15% of the total) in *T. pisiformis* and *T. solium*, followed by cellular component (27,567, 31.16%) and molecular function (13,882, 15.69%). The functions determined by the GO classification mostly focused on cell, cell part, organelle, binding, catalytic activity, cellular process, localization, and metabolic process. Three GO terms only existed in *T. solium*: virion (4 ESTs), virion part (4 ESTs), and electron carrier (8 ESTs).

#### 3) M genes (*T. pisiformis/T. solium/E. granulosis/E. multilocularis*)

We obtained 600 common genes (M) that existed in four species of Taeniidae. M was aligned in the KEGG database using BLASTx; 109 genes of M obtained 118 annotations (**Dataset S11**) and 21 pathways (**Dataset S12**). A series of key genes were identified that were closely related to the biochemistry of cestode, such as the G protein-coupled receptor 128 (ko08464), formin 2 (ko12821), survival motor neuron protein (ko13129), MKP (ko04459) of the MAPK signaling pathway, WASP (ko05747) in the adherens junction and chemokine signaling pathway, SF3a (ko12826) and FBP11 (ko12821) in splicesomes. In 21 pathways, approximately 36.7% genes were conserved in amoebas and *Vibrio cholerae*. The intestinal mucin was found in these two pathways. Furthermore, the adenomatosis polyposis coli (APC) protein was present in the ‘Regulation of actin cytoskeleton’, ‘Wnt signaling pathway’, ‘Colorectal cancer’, ‘Pathways in cancer’, and ‘Endometrial cancer’ (**Table 3**).

**Table 3:** M (common genes in *T. pisiformis*, *T. solium*, *E. granulosis*, and *E. multilocularis*) was aligned KEGG database using BLASTx, and 109 genes obtained 21 pathways.

Pathway	Count (109 genes)	Pathway ID
Amoebiasis	20	ko05146
*Vibrio cholerae* infection	20	ko05110
Dorso-ventral axis formation	18	ko04320
Salivary secretion	14	ko04970
Regulation of actin cytoskeleton	9	ko04810
Pathogenic *Escherichia coli* infection	4	ko05130
Spliceosome	4	ko03040
Shigellosis	4	ko05131
Fc gamma R-mediated phagocytosis	3	ko04666
Chemokine signaling pathway	3	ko04062
Bacterial invasion of epithelial cells	3	ko05100
Adherens junction	3	ko04520
Basal cell carcinoma	2	ko05217
Wnt signaling pathway	2	ko04310
Systemic lupus erythematosus	2	ko05322
Endometrial cancer	2	ko05213
Pathways in cancer	2	ko05200
Colorectal cancer	2	ko05210
Focal adhesion	1	ko04510
MAPK signaling pathway	1	ko04010
RNA degradation	1	ko03018

## Discussion

The characterization of the transcriptome is essential for deciphering the functional complexity of the genome and to obtain a better understanding of cellular activities in organisms, including growth, development, disease, and immune defense [23]. The RNA-seq approach has not only proven to be an efficient method for transcriptome profiling analyzes [24], but is also effective in clarifying transcriptome complexity. In this study, the transcripts of *T. pisiformis* indicated that *de novo* short-read assembly and the Solexa/Illumina platform offered a larger number of distinct reads and increased physical coverage as a result of long fragment lengths. Those long fragments could be used to characterize gene expression, discover and identify new genes, and research metabolic pathways in non-reference whole genomes.

The great numbers of clean reads and unigenes resulted in a relatively deep coverage. Assembly results showed that the mean length of unigenes was much longer than that of contigs. In addition, the mean length of *T. pisiformis* unigenes was longer than those assembled in previous studies, such as *Eucalyptus grandis* (247 bp) [25]. The percentages of no gaps and high matching scores between unigenes and clean reads indicated high quality and validity level of the SOAPdenovo assembly. Annotated unigenes were assigned with not only gene or protein name descriptions, but also putative conserved domains, GO terms, and metabolic or signaling pathways (only in the KEGG database). The annotations of unigenes provided the biological functions, metabolic and signaling pathways of candidate genes in a given time. We therefore gained a better understanding of the gene expression of *T. pisiformis* in the terminal host, and searched for antigens of *C. pisiformis* in intermediate hosts. Functional annotations in the Nr database laid a foundation for the analysis of gene ontology. All annotated unigenes in the Swiss-prot database played an important role in functional annotations.

A large number of unigenes were assigned to a wide range of COG classifications, which indicated that our RNA-seq data represented a wide diversity of transcripts. COGs consist of protein sequences encoded in 21 complete genomes, including bacteria, algae and eukaryotes, and were built on classifications according to phylogenetic relationships [26]. Each COG consists of individual proteins or groups of analogs from at least three lineages and thus corresponds to an ancient conserved domain [26]. In 25 molecular families, H (coenzyme transport and metabolism), L (replication, recombination and repair), U (intracellular trafficking secretion, and vesicular transport) and V (defense mechanisms) played a significant role, and were closely associated with the immunology and physiology of *T. pisiformis*. The analysis of gene expression levels, a GO classification of genetic functions and prediction of protein metabolic pathways, predicted the screening of differentially expressed genes, GO functional classification of different genes and positioning of metabolic pathways. These sequence data and statistical analysis have provided abundant information on *T. pisiformis* infections, enabling a better understanding of antigens and the basic functional distribution of the gene.

At present, there are just 26 ESTs in NCBI, and only three antigens of *T. pisiformis* have been registered on NCBI: T24 (GenBank: GU321333.1), cathepsin L-like cysteine protease (GenBank: JF798507.1), and cysteine protease (GenBank: JF718743.1). The substantial amounts of antigens were aligned and had clusters of orthologous group with other tapeworm species. For example, Tso31d [27], Tm18 [27], cC1 [28], and paramyosin [29] could be used as vaccine antigens; GP50 [30], cathepsin L-like cysteine proteinase [31], and heat shock protein [32] could be used as effective diagnostic antigens. In addition, vast antigens of oncosphere can be found in annotated and no hit unigenes for protecting intermediate host. *T. pisiformis* also has a homologous CDS with *Caenorhabditis elegans*, *Ancylostoma caninum*, *Schistosoma mansoni* and *Leishmania infantum*. This indicates that RNA-seq data indeed had the necessary depth, coverage and diversity. Therefore, we could screen mass new genes for *T. pisiformis* from those annotated CDS. Paramyosin is not only utilized as an antigen, but may also have other biological functions in *T. pisiformis*. For example, the metacestodes have elaborate means of evading complement-mediated destruction, including paramyosin (restraint C1q) [33]. Antioxidant like the CDS of thioredoxin peroxidase (TPX) plays a role in protecting against oxidative damage. Additionally, recombinant EgTPx may be useful for the screening of specific inhibitors that could serve as new drugs for the treatment of hydatid disease [34]. Thus, we predict that those key genes may play an important role in life activites of *T. pisiformis* in the small intestine of dogs, which need be analyzed in further study.

Functional unigenes closely relate to the metabolic or signaling pathways, which play an important role in life history of *T. pisiformis*. In this study, we analyzed the glycolysis/gluconeogenesis and axon guidance pathways. Phosphofructokinase is not only a key enzyme in the pathway of glycolysis/gluconeogenesis, but also the biochemical basis of the long-term utilization of antimony for the treatment of *schistosomiasis* [35]. Therefore, further research into analogous enzymes could provide new potential pharmacotherapeutic targets for the treatment of cestode infections. Meanwhile, we suppose that anerobic glycolysis is the principal way that cestodes obtain energy in the intestinal tract of a host. Cestodes have a developed nervous system that plays an important role in multiple aspects of life activity, such as growth and development, muscle movement, metabolism of salt and water, and reproduction. The secretory vesicles of nerve cells play a neural regulatory role via axonal guidance [36]. Therefore, axon guidance represents a key stage in the formation of neuronal networks. Netrins, slits, semaphorins, and ephrins are the highly conservative protein families that are involved in axonal guidance, which afford a research basis for the neurobiology of *T. pisiformis*. These guidance cues are read by growth cone receptors, and signal transduction pathways downstream of these receptors converge onto the Rho GTPases to elicit changes in cytoskeletal organization that determine the direction that the growth cone will turn [37]. As cestodes live in the small intestine of dogs, two aspects of the firm attachment to the host small intestine and high fertility rates are very important to *T. pisiformis*. Sensory organs in tapeworm have gradually diminished due to life in the host small intestine. Thus, the developed nervous system plays an important role in the life activities of *T. pisiformis*.

*T. pisiformis*, *T. solium*, *E. granulosis*, and *E. multilocularis* are important species of tapeworm that damage public health and bring huge economic losses in developing countries [38]–[40]. In particular, *T. solium*, *E. granulosis*, and *E. multilocularis* can severely damage human health. Thus, prophylaxis and treatment against these tapeworms are particularly important. A large number of COG and GO functional annotations were related to the basic life activities of the cestode. Functional distribution characteristics were consequently obtained from these data. Some of common genes (M) from four species of Taeniidae in the KEGG pathway were identified to be related to cestode biochemistry. The G protein-coupled receptor [41] and survival motor neuron protein [42] mediate signal transduction. MKP is a dual-specificity phosphatase family that is involved in the MAPK signaling pathway, and includes MKP-2 that dephosphorylates and inactivates mitogen-activated protein kinases (MAPKs) [43].

Interestingly, we found two common genes in four cestode species through the analysis of comparative transcripts for intestinal mucin and APC genes. Amoebiasis and cholera are serious human gastrointestinal diseases caused by *Entamoebahistolytica* and *Vibrio cholerae*, respectively [44], [45]. Lee et al. (2010) indicated that *Gymnophalloides seoi* antigen upregulates the expression of Toll-like receptor 2 and mucin-related 2 by human intestinal epithelial cells, which reflects a helminth-induced, IFN-c–dependent, and innate mucosal immune mechanism in this human intestinal cell line [46]. In addition, APC participates in the Wnt signaling pathway. When spontaneous mutations occur that means APC cannot play a normal physiological function, the individual is at a higher risk of cancers, including colorectal cancer [47]. The APC gene has been reported in canines in the NCBI database, whereas intestinal mucin is not conserved in canines. Scholl (2003) argued that horizontal gene transfer between parasites and hosts, sometimes involving viral-bacterial-parasite-host chains, might be of great consequence for the evolution of hosts and parasites [48]. We cannot as yet whether the APC gene of *T. pisiformis* has been transferred from dogs, but this will undergo further analysis. Thus, there is major merit in undertaking comparative functional genomics, transcriptomic and proteomic investigations to establish whether intestinal mucin 2 and APC play a similar role in the development of these four cestode species and their transition to parasitism in canine intestines. These genes may be putative parasitism-related genes in cestodes that are adapted to avoid host immunity. Further gene expression profiling and experimental validation will be needed to test the functional role of these new *T. pisiformis* genes.

To the best of our knowledge, this is the first study that uses a *de novo* short-read assembly and the Solexa/Illumina platform to generate transcriptome (RNA-seq) data for *T. pisiformis*. In this study, we obtained 72,957 assembled unigenes, and 26,012 unigenes of them acquired the annotations. The identification of metabolic and signaling pathways in the present study will accelerate the understanding of the pathways of energy, anti-oxidation, immune system formation and development. This study demonstrates that this form of sequencing platform can be used as a rapid and cost-effective method for the analysis of non-model whole genomes. We utilized the unigenes of *T. pisiformis* to align against ESTs of *T. solium*, *E. granulosis*, and *E. multilocularis*. Functional distribution characteristics and common genes sets were obtained. We believe that this transcriptome dataset will serve as an important public information platform for *T. pisiformis*, which will accelerate research into gene expression, genomics, and functional genomics of cestodes. The identification of these common genes will provide novel platform for the development of vaccine candidates and drug targets.

## Materials and Methods

### Parasite material

Under supervision of a licensed veterinarian, the larvae (*C. pisiformis*) were collected (during a routine autopsy) from the gastric omentum majus of two dead New Zealand white rabbit naturally infected by this tapeworm in a farm, Sichuan, China. After morphological identification, 20 *C. pisiformis* from the rabbits were used for the dog infection. Adult worms (*T. pisiformis*) were taken out of small intestine, and then washed in warm physiological saline for three times to avoid contamination before they were frozen immediately and stored in liquid nitrogen. All animals were handled in strict accordance with animal protection law of the People's Republic of China (a draft of an animal protection law in China released on September 18, 2009). All study protocols (including the collection of the larvae from the rabbits, and the infection and sacrifice of the dogs) were reviewed and approved by the National Institute of Animal Health Animal Care and Use Committee at Sichuan Agricultural University, China (approval ID number 2009-013, approved for three years beginning 06/2/2009).

### RNA isolation and Illumina sequencing

Total RNA was isolated from single adult *T. pisiformis* (2.5 g, including scoles, neck, and strobila) using Trizol reagent (Invitrogen, Life Technologies, Carlsbad, CA, USA) according to the manufacturer's protocol. Total RNA of independent adult *T. pisiformis* were stored at −80°C until their use. The RNA quality was verified by an Agilent 2100 RNA Nanochip (Agilent, Santa Clara, CA, USA) in terms of concentration (2.57 ng/µl), RNA integrity number (RIN: 5.3) and the 28S∶18S ratio (1.0). A total of 244.15 µg of RNA was pooled from adult *T. pisiformis* for the preparation of the cDNA library.

The OligoTex mRNA mini kit (Qiagen) was used to isolate poly (A) mRNA after total RNA was collected from adult *T. pisiformis* according to the manufacturer's protocol. Fragmentation buffer was added to interrupt mRNA to short fragments (100–400 bp). Following the agarose gel electrophoresis (2% TAE-agarose gel), a range of cDNA fragments (200±25 bp) were excised from the gel, and selected for the PCR amplification as templates using the SuperScript Double-Stranded cDNA Synthesis kit (Invitrogen, Camarillo, CA) following the manufacturer's protocol, and PCR Primer PE 2.0 (Illumina, San Diego, CA). Illumina HiSeq™ 2000 was applied to sequencing at the Beijing Genomics Institute (BGI)-Shenzhen, Shenzhen, China (http://www.genomics.cn/index.php) according to the manufacturer's instructions (Illumina).

### De novo assembly

Prior to assembly, the high-quality clean reads were obtained from raw reads by removing adaptor sequences, duplication sequences, reads that contained more than 10% “N” rates (the “N” character representing ambiguous bases in reads), and low-quality reads containing more than 10% bases with Q-value≤20. Then, 21-bp K-mers was utilized in the assembly of the short reads when using the SOAPdenovo program (http://soap.genomics.org.cn/soapdenovo.html) [49]. The detailed process of the assembly is described as supplemental Dataset S13. Finally, non-redundant unigenes were obtained with as long as length as possible.

### Pipeline of bioinformatics analysis

BLASTx alignment (e-value<10^−5^) between unigenes and protein databases was performed, such as NCBI non-redundant protein (Nr) database (http://www.ncbi.nlm.nih.gov), Swiss-prot protein database (http://www.expasy.ch/sprot), the Kyoto Encyclopedia of Genes and Genomes (KEGG) pathway database (http://www.genome.jp/kegg), and the Cluster of Orthologous Groups (COG) database (http://www.ncbi.nlm.nih.gov/COG). The sequence direction of unigenes, and expression and functional annotation of unigenes were then decided by the best alignment results. BLASTx first aligned unigene sequences in four protein databases (e-value<10^−5^), which retrieved proteins with the highest sequence similarity with the given unigenes along with their protein functional annotations. Annotated unigenes were classified by Gene Ontology (GO) that was aligned by Nr database. With Nr annotations, Blast2GO program [50] was used to obtain GO annotations of unigenes. After receiving GO annotations for all unigenes, WEGO software [51] classified them to understand the distribution of gene functions of the species macroscopically. In addition, there had a high density of coding sequences (CDS) in comparison to most eukaryotes. Unigenes aligned to databases with higher priority did not enter the next circle. The alignments ended when all circles were finished. Both the nucleotide sequences (5′-3′) and amino sequences of the unigene coding regions were obtained. Unigenes that could not be aligned to any database were scanned by ESTScan [52] to obtain the nucleotide (5′-3′) and amino sequences of the coding regions.

### Assessment to quality and reliability of transcriptome of adult *T. pisiformis*

Unigenes were aligned with clean reads of the *T. pisiformis* transcriptome. The map percentages were obtained between them, which assisted the analysis of the quality of the assembly. Furthermore, the CDS of four antigens were amplified by RACE PCR using cDNA of adult *T. pisiformis* (primers and annealing temperatures are shown in Table S1). Oligonucleotide primers of four antigens were designed using Primer software version 5.0.

### Comparative transcripts analysis

Firstly, the ESTs of *E. granulosus* (ftp://ftp.sanger.ac.uk/pub/pathogens/Echinococcus/granulosis/ESTs/fasta.gz) and *E. multilocularis* (ftp://ftp.sanger.ac.uk/pub/pathogens/Echinococcus/multilocularis/ESTs/fasta.gz) were translated into amino acid sequences respectively, before the two tapeworm species were aligned with the BLASTx algorithm (e-value<10^−5^). Consequently, we obtained the common ESTs in *E. granulosus* and *E. multilocularis* that were termed Eg+Em. Secondly, the Eg+Em, unigenes of *T. pisiformis*, and ESTs of *T. solium* (http://www.ncbi.nlm.nih.gov/nucest?term=Taenia%20solium) were classified in COG and GO functional terms. The mutual-best matches among Eg+Em, *T. pisiformis*, and *T. solium* were carried out using the BLASTx algorithm. Finally, the common genes (termed M) that were conserved between *T. pisiformis*, *T. solium*, *E. granulosus*, and *E. multilocularis* were aligned to the KEGG database using the BLASTx algorithm.

## Supporting Information

Table S1 **Oligonucleotide primers for RACE-PCR of four antigens.** The 3′- and 5′-end of the gene was amplified by RACE PCR using the five oligonucleotide primers (reverse transcription, first-round nested PCR, and second-round nested-PCR) and TIANscript RT Kit (TianGenBioteh CO., LTD, Beijing), according to the manufacturer's manual.
(DOC)

Dataset S1 **A total of 72,957 unigenes were generated using the RNA-seq technique or** ***Taenia pisiformis*** **.** The serial numbers of unigenes were used in keeping with the Dataset S2 to S7, S10 to S
(RAR)

Dataset S2 **The transcriptome annotations of** ***Taenia pisiformis*** **in non-redundant protein (Nr) database.** Overall, 25,701 unigenes and 201,908 subject functional annotations were obtained from the Nr database.
(XLS)

Dataset S3 **The transcriptome annotations of** ***Taenia pisiformis*** **in Swiss-prot database.** A total of 19,564 unigenes and 148,048 subject functional annotations were obtained from the Swiss-prot database.
(XLS)

Dataset S4 **The transcriptome annotations of** ***Taenia pisiformis*** **in KEGG database.** Overall, 15,920 unigenes and 134,829 subject functional annotations were obtained from the KEGG database.
(XLSX)

Dataset S5 **The transcriptome annotations of** ***Taenia pisiformis*** **in COG database.** A total of 7,760 unigenes and 47,241 subject functional annotations were obtained from the COG database.
(XLS)

Dataset S6 **The 203 KEGG pathways of** ***Taenia pisiformis*** **.** In order to identify the active biological pathways in *T. pisiformis*, a total of 15,920 unigenes were assigned to 203 KEGG pathways.
(HTM)

Dataset S7 **The glycolysis/gluconeogenesis and axon guidance pathway of** ***Taenia pisiformis*** **.** There were three key enzymes in glycolysis/gluconeogenesis: hexokinase (EC 2.7.1.1), phosphofructokinase (EC 4.1.2.13), and pyruvate kinase (EC 2.7.1.40). Additionally, there were four highly conservative axon guidance molecular families, netrins (netrin 1), slits (slits 1, slits 2, and slits 3), semaphorins (sema4D, sema5, and sema6), and ephrins (ephrin E).
(DOCX)

Dataset S8 **The ESTs of Eg+Em were classified into at least 25 molecular families against COG database.**
(RAR)

Dataset S9 **The ESTs of** ***Taenia solium*** **were classified into at least 25 molecular families against COG database.**
(RAR)

Dataset S10 **The ESTs of** ***Taenia solium*** **obtained the GO annotations according to Nr database.**
(RAR)

Dataset S11 **Annotation of M (common genes of** ***Taenia pisiformis*** **,** ***Taenia solium*** **,** ***Echinococcus granulosis*** **and** ***Echinococcus multilocularis*** **).** The genes annotations of M (*T. pisiformis*, *T. solium*, *E. granulosis*, and *E. multilocularis*) against the KEGG database.
(RAR)

Dataset S12 **21 KEGG pathways of** ***Taenia pisiformis*** **,** ***Taenia solium*** **,** ***Echinococcus granulosis*** **and** ***Echinococcus multilocularis*** **.** M was aligned in the KEGG database using BLASTx, and 109 genes obtained 21 pathways.
(HTM)

Dataset S13 **The process of the SOAPdenovo assembly.**
(DOCX)
