# Supplementary material for: Annotation of the Transcriptome from Taenia pisiformis and Its Comparative Analysis with Three Taeniidae Species
Source: PLoS One. 2012 Apr 13;7(4):e32283. doi: 10.1371/journal.pone.0032283 (PMC3326008; doi:10.1371/journal.pone.0032283)
Supplement: Table S1 — Oligonucleotide primers for RACE-PCR of four antigens. The 3′- and 5′-end of the gene was amplified by RACE PCR using the five oligonucleotide primers (reverse transcription, first-round nested PCR, and second-round nested-PCR) and TIANscript RT Kit (TianGenBioteh CO., LTD, Beijing), according to the manufacturer's manual. (DOC) [file pone.0032283.s001.doc]

**Table S1** Oligonucleotide primers were designed for RACE-PCR of four antigens from adult *Taenia pisiformis* transcriptome.

|  | | Primer name | Primer sequence (5’-3’) |
| --- | --- | --- | --- |
| Reverse transcription | | SMART III | AAGCAGTGGTATCAACGCAGAGTGGCCATTATGGCCGGG |
|  |  | BRL-A1 | GGCCACGCGTCGACTAGTACTTTTTTTTTTTTTTTTT |
| Universal primers | First nested PCR | 5'Smart P1 | AAGCAGTGGTATCAACGCAGAGT |
|  |  | 3' BRL-A2 | GGCCACGCGTCGACTAGTAC |
|  | Second nested-PCR | 5'Smart P1 | ATCAACGCAGAGTGGCCATTATG |
|  |  | 3' BRL-A2 | GGCCACGCGTCGACTAGTAC |
| homologous antigen 18KD | First nested-PCR | 3' F1 | AACAATCGTCCTTACCGTATCATCT |
|  | Second nested-PCR | 3' F2 | GGTCTGCCCTTGCGATGGAAAATAC |
| Cc1 | First nested-PCR | 3' F1 | GCAATAAAGAAGGAGACGACGAGAG |
|  |  | 5' R1 | AGCCAATGAGTTTCAAGCAGAGAGT |
|  | Second nested-PCR | 3' F2 | TGAGGGCTGATACCGATTTGGGAAG |
|  |  | 5' R2 | ATGGCAAAGTGGAGCAGTTCGGC |
| TP1 | First nested-PCR | 3' F1 | CCAAACAAAACCAAATGGAGACTG |
|  |  | 5' R1 | CATCGTCCATAGCGTCGGAAATCAT |
|  | Second nested-PCR | 3' F2 | GCTGAAATCTACTGCTCAAATGTCG |
|  |  | 5' R2 | CGTTCGTGTTCGGACCAGAT |
| TPP2 | First nested-PCR | 3' F1 | ATGGGTGTTCTTCAAGGTGG |
|  | Second nested-PCR | 3' F2 | TTAATAAACCGCATAACT |
