# Supplementary material for: Annotation of the Transcriptome from Taenia pisiformis and Its Comparative Analysis with Three Taeniidae Species
Source: PLoS One. 2012 Apr 13;7(4):e32283. doi: 10.1371/journal.pone.0032283 (PMC3326008; doi:10.1371/journal.pone.0032283)
Supplement: Dataset S6 — The 203 KEGG pathways of Taenia pisiformis . In order to identify the active biological pathways in T. pisiformis, a total of 15,920 unigenes were assigned to 203 KEGG pathways. (HTM) [file pone.0032283.s007.htm]

TAEpaeTARAAPEI-5-Unigene.fa

1. TAEpaeTARAAPEI-5-Unigene.fa

| # | Pathway | Count (15921) | Pathway ID |
| 1 | Metabolic pathways | 2511 | ko01100 |
| 2 | Spliceosome | 752 | ko03040 |
| 3 | Regulation of actin cytoskeleton | 668 | ko04810 |
| 4 | Huntington's disease | 651 | ko05016 |
| 5 | Focal adhesion | 637 | ko04510 |
| 6 | Pathways in cancer | 612 | ko05200 |
| 7 | Tight junction | 552 | ko04530 |
| 8 | Endocytosis | 548 | ko04144 |
| 9 | Purine metabolism | 543 | ko00230 |
| 10 | MAPK signaling pathway | 485 | ko04010 |
| 11 | Adherens junction | 456 | ko04520 |
| 12 | Cell cycle | 441 | ko04110 |
| 13 | Insulin signaling pathway | 406 | ko04910 |
| 14 | Vascular smooth muscle contraction | 392 | ko04270 |
| 15 | Ubiquitin mediated proteolysis | 387 | ko04120 |
| 16 | Alzheimer's disease | 385 | ko05010 |
| 17 | Wnt signaling pathway | 376 | ko04310 |
| 18 | Lysosome | 371 | ko04142 |
| 19 | Dilated cardiomyopathy | 348 | ko05414 |
| 20 | Pathogenic Escherichia coli infection | 338 | ko05130 |
| 21 | Hypertrophic cardiomyopathy (HCM) | 336 | ko05410 |
| 22 | Chemokine signaling pathway | 329 | ko04062 |
| 23 | Fc gamma R-mediated phagocytosis | 320 | ko04666 |
| 24 | Pyrimidine metabolism | 319 | ko00240 |
| 25 | Oocyte meiosis | 314 | ko04114 |
| 26 | Vibrio cholerae infection | 310 | ko05110 |
| 27 | Calcium signaling pathway | 302 | ko04020 |
| 28 | Oxidative phosphorylation | 301 | ko00190 |
| 29 | Cardiac muscle contraction | 286 | ko04260 |
| 30 | Parkinson's disease | 282 | ko05012 |
| 31 | Axon guidance | 262 | ko04360 |
| 32 | Neurotrophin signaling pathway | 255 | ko04722 |
| 33 | RNA degradation | 254 | ko03018 |
| 34 | Leukocyte transendothelial migration | 252 | ko04670 |
| 35 | Viral myocarditis | 240 | ko05416 |
| 36 | Gap junction | 222 | ko04540 |
| 37 | GnRH signaling pathway | 217 | ko04912 |
| 38 | Progesterone-mediated oocyte maturation | 216 | ko04914 |
| 39 | Glycolysis / Gluconeogenesis | 215 | ko00010 |
| 40 | ErbB signaling pathway | 210 | ko04012 |
| 41 | Aminoacyl-tRNA biosynthesis | 210 | ko00970 |
| 42 | ECM-receptor interaction | 207 | ko04512 |
| 43 | Ribosome | 202 | ko03010 |
| 44 | T cell receptor signaling pathway | 199 | ko04660 |
| 45 | Melanogenesis | 194 | ko04916 |
| 46 | Epithelial cell signaling in Helicobacter pylori infection | 194 | ko05120 |
| 47 | Phosphatidylinositol signaling system | 193 | ko04070 |
| 48 | Prostate cancer | 193 | ko05215 |
| 49 | Long-term potentiation | 192 | ko04720 |
| 50 | Pyruvate metabolism | 182 | ko00620 |
| 51 | Vasopressin-regulated water reabsorption | 181 | ko04962 |
| 52 | TGF-beta signaling pathway | 178 | ko04350 |
| 53 | Glycerophospholipid metabolism | 177 | ko00564 |
| 54 | Nucleotide excision repair | 174 | ko03420 |
| 55 | Renal cell carcinoma | 171 | ko05211 |
| 56 | Lysine degradation | 165 | ko00310 |
| 57 | Antigen processing and presentation | 164 | ko04612 |
| 58 | Amyotrophic lateral sclerosis (ALS) | 163 | ko05014 |
| 59 | Arrhythmogenic right ventricular cardiomyopathy (ARVC) | 161 | ko05412 |
| 60 | DNA replication | 160 | ko03030 |
| 61 | Olfactory transduction | 160 | ko04740 |
| 62 | Prion diseases | 159 | ko05020 |
| 63 | Adipocytokine signaling pathway | 158 | ko04920 |
| 64 | PPAR signaling pathway | 156 | ko03320 |
| 65 | Proteasome | 156 | ko03050 |
| 66 | N-Glycan biosynthesis | 155 | ko00510 |
| 67 | Amino sugar and nucleotide sugar metabolism | 152 | ko00520 |
| 68 | Long-term depression | 152 | ko04730 |
| 69 | Peroxisome | 151 | ko04146 |
| 70 | Fructose and mannose metabolism | 148 | ko00051 |
| 71 | Chronic myeloid leukemia | 148 | ko05220 |
| 72 | Natural killer cell mediated cytotoxicity | 147 | ko04650 |
| 73 | Colorectal cancer | 144 | ko05210 |
| 74 | Notch signaling pathway | 143 | ko04330 |
| 75 | Inositol phosphate metabolism | 141 | ko00562 |
| 76 | VEGF signaling pathway | 140 | ko04370 |
| 77 | Fc epsilon RI signaling pathway | 139 | ko04664 |
| 78 | Starch and sucrose metabolism | 137 | ko00500 |
| 79 | Glioma | 137 | ko05214 |
| 80 | Small cell lung cancer | 136 | ko05222 |
| 81 | Type II diabetes mellitus | 136 | ko04930 |
| 82 | mTOR signaling pathway | 135 | ko04150 |
| 83 | Citrate cycle (TCA cycle) | 134 | ko00020 |
| 84 | Glycerolipid metabolism | 130 | ko00561 |
| 85 | Riboflavin metabolism | 128 | ko00740 |
| 86 | Dorso-ventral axis formation | 127 | ko04320 |
| 87 | Basal transcription factors | 126 | ko03022 |
| 88 | B cell receptor signaling pathway | 125 | ko04662 |
| 89 | Glutathione metabolism | 123 | ko00480 |
| 90 | Pancreatic cancer | 119 | ko05212 |
| 91 | Endometrial cancer | 118 | ko05213 |
| 92 | Jak-STAT signaling pathway | 117 | ko04630 |
| 93 | Galactose metabolism | 116 | ko00052 |
| 94 | Neuroactive ligand-receptor interaction | 114 | ko04080 |
| 95 | ABC transporters | 114 | ko02010 |
| 96 | Fatty acid metabolism | 109 | ko00071 |
| 97 | RNA polymerase | 107 | ko03020 |
| 98 | Acute myeloid leukemia | 105 | ko05221 |
| 99 | Propanoate metabolism | 104 | ko00640 |
| 100 | Valine, leucine and isoleucine degradation | 103 | ko00280 |
| 101 | Cell adhesion molecules (CAMs) | 101 | ko04514 |
| 102 | Base excision repair | 100 | ko03410 |
| 103 | Tyrosine metabolism | 99 | ko00350 |
| 104 | Pentose phosphate pathway | 98 | ko00030 |
| 105 | Non-small cell lung cancer | 97 | ko05223 |
| 106 | Hedgehog signaling pathway | 97 | ko04340 |
| 107 | Mismatch repair | 97 | ko03430 |
| 108 | p53 signaling pathway | 95 | ko04115 |
| 109 | Toll-like receptor signaling pathway | 92 | ko04620 |
| 110 | Arginine and proline metabolism | 92 | ko00330 |
| 111 | Alanine, aspartate and glutamate metabolism | 91 | ko00250 |
| 112 | Aldosterone-regulated sodium reabsorption | 89 | ko04960 |
| 113 | Apoptosis | 86 | ko04210 |
| 114 | Drug metabolism - other enzymes | 85 | ko00983 |
| 115 | Taste transduction | 84 | ko04742 |
| 116 | Basal cell carcinoma | 82 | ko05217 |
| 117 | Tryptophan metabolism | 80 | ko00380 |
| 118 | Homologous recombination | 79 | ko03440 |
| 119 | NOD-like receptor signaling pathway | 77 | ko04621 |
| 120 | Butanoate metabolism | 74 | ko00650 |
| 121 | MAPK signaling pathway - fly | 74 | ko04013 |
| 122 | Valine, leucine and isoleucine biosynthesis | 73 | ko00290 |
| 123 | Selenoamino acid metabolism | 71 | ko00450 |
| 124 | Protein export | 70 | ko03060 |
| 125 | Systemic lupus erythematosus | 68 | ko05322 |
| 126 | Melanoma | 67 | ko05218 |
| 127 | Cysteine and methionine metabolism | 67 | ko00270 |
| 128 | Sphingolipid metabolism | 67 | ko00600 |
| 129 | Phenylalanine metabolism | 67 | ko00360 |
| 130 | RIG-I-like receptor signaling pathway | 66 | ko04622 |
| 131 | Bladder cancer | 66 | ko05219 |
| 132 | Regulation of autophagy | 65 | ko04140 |
| 133 | beta-Alanine metabolism | 62 | ko00410 |
| 134 | Glycine, serine and threonine metabolism | 60 | ko00260 |
| 135 | Thyroid cancer | 58 | ko05216 |
| 136 | Hematopoietic cell lineage | 58 | ko04640 |
| 137 | Arachidonic acid metabolism | 58 | ko00590 |
| 138 | Glycosylphosphatidylinositol(GPI)-anchor biosynthesis | 56 | ko00563 |
| 139 | Ether lipid metabolism | 56 | ko00565 |
| 140 | SNARE interactions in vesicular transport | 56 | ko04130 |
| 141 | Nicotinate and nicotinamide metabolism | 55 | ko00760 |
| 142 | Pentose and glucuronate interconversions | 53 | ko00040 |
| 143 | Porphyrin and chlorophyll metabolism | 52 | ko00860 |
| 144 | O-Glycan biosynthesis | 52 | ko00512 |
| 145 | Complement and coagulation cascades | 52 | ko04610 |
| 146 | Limonene and pinene degradation | 48 | ko00903 |
| 147 | Cytokine-cytokine receptor interaction | 48 | ko04060 |
| 148 | Biosynthesis of unsaturated fatty acids | 46 | ko01040 |
| 149 | Histidine metabolism | 44 | ko00340 |
| 150 | Pantothenate and CoA biosynthesis | 44 | ko00770 |
| 151 | Nitrogen metabolism | 44 | ko00910 |
| 152 | Terpenoid backbone biosynthesis | 42 | ko00900 |
| 153 | Metabolism of xenobiotics by cytochrome P450 | 40 | ko00980 |
| 154 | One carbon pool by folate | 39 | ko00670 |
| 155 | Drug metabolism - cytochrome P450 | 37 | ko00982 |
| 156 | Methane metabolism | 36 | ko00680 |
| 157 | Other glycan degradation | 36 | ko00511 |
| 158 | Glycosaminoglycan biosynthesis - heparan sulfate | 36 | ko00534 |
| 159 | Cytosolic DNA-sensing pathway | 35 | ko04623 |
| 160 | Glyoxylate and dicarboxylate metabolism | 34 | ko00630 |
| 161 | Fatty acid biosynthesis | 32 | ko00061 |
| 162 | Non-homologous end-joining | 32 | ko03450 |
| 163 | Glycosaminoglycan biosynthesis - chondroitin sulfate | 29 | ko00532 |
| 164 | Ascorbate and aldarate metabolism | 28 | ko00053 |
| 165 | Maturity onset diabetes of the young | 28 | ko04950 |
| 166 | Renin-angiotensin system | 27 | ko04614 |
| 167 | Primary immunodeficiency | 25 | ko05340 |
| 168 | Fatty acid elongation in mitochondria | 25 | ko00062 |
| 169 | Linoleic acid metabolism | 22 | ko00591 |
| 170 | Retinol metabolism | 22 | ko00830 |
| 171 | Circadian rhythm - fly | 22 | ko04711 |
| 172 | Thiamine metabolism | 21 | ko00730 |
| 173 | Ubiquinone and other terpenoid-quinone biosynthesis | 20 | ko00130 |
| 174 | Cyanoamino acid metabolism | 19 | ko00460 |
| 175 | Glycosaminoglycan degradation | 18 | ko00531 |
| 176 | Sulfur metabolism | 17 | ko00920 |
| 177 | alpha-Linolenic acid metabolism | 17 | ko00592 |
| 178 | Steroid hormone biosynthesis | 17 | ko00140 |
| 179 | Glycosphingolipid biosynthesis - lacto and neolacto series | 16 | ko00601 |
| 180 | Glycosphingolipid biosynthesis - ganglio series | 15 | ko00604 |
| 181 | Taurine and hypotaurine metabolism | 15 | ko00430 |
| 182 | Glycosphingolipid biosynthesis - globo series | 14 | ko00603 |
| 183 | Folate biosynthesis | 14 | ko00790 |
| 184 | Primary bile acid biosynthesis | 13 | ko00120 |
| 185 | Type I diabetes mellitus | 13 | ko04940 |
| 186 | Lipoic acid metabolism | 11 | ko00785 |
| 187 | Phenylalanine, tyrosine and tryptophan biosynthesis | 11 | ko00400 |
| 188 | Synthesis and degradation of ketone bodies | 11 | ko00072 |
| 189 | D-Glutamine and D-glutamate metabolism | 10 | ko00471 |
| 190 | Glycosaminoglycan biosynthesis - keratan sulfate | 9 | ko00533 |
| 191 | Circadian rhythm - mammal | 9 | ko04710 |
| 192 | Autoimmune thyroid disease | 9 | ko05320 |
| 193 | Steroid biosynthesis | 7 | ko00100 |
| 194 | Lysine biosynthesis | 7 | ko00300 |
| 195 | Biotin metabolism | 7 | ko00780 |
| 196 | Vitamin B6 metabolism | 7 | ko00750 |
| 197 | D-Arginine and D-ornithine metabolism | 5 | ko00472 |
| 198 | Intestinal immune network for IgA production | 5 | ko04672 |
| 199 | Allograft rejection | 4 | ko05330 |
| 200 | O-Mannosyl glycan biosynthesis | 4 | ko00514 |
| 201 | Asthma | 3 | ko05310 |
| 202 | Graft-versus-host disease | 3 | ko05332 |
| 203 | Polyketide sugar unit biosynthesis | 2 | ko00523 |

| # | Pathway | Differentially expressed genes |
| --- | --- | --- |
| 1 | Metabolic pathways (no map in kegg database) | Unigene10046\_TAEpaeTARAAPEI-5, Unigene1005\_TAEpaeTARAAPEI-5, Unigene10103\_TAEpaeTARAAPEI-5, Unigene10130\_TAEpaeTARAAPEI-5, Unigene10137\_TAEpaeTARAAPEI-5, Unigene10180\_TAEpaeTARAAPEI-5, Unigene10240\_TAEpaeTARAAPEI-5, Unigene10260\_TAEpaeTARAAPEI-5, Unigene10264\_TAEpaeTARAAPEI-5, Unigene10290\_TAEpaeTARAAPEI-5, Unigene10292\_TAEpaeTARAAPEI-5, Unigene10293\_TAEpaeTARAAPEI-5, Unigene102\_TAEpaeTARAAPEI-5, Unigene10314\_TAEpaeTARAAPEI-5, Unigene10327\_TAEpaeTARAAPEI-5, Unigene10348\_TAEpaeTARAAPEI-5, Unigene10377\_TAEpaeTARAAPEI-5, Unigene1038\_TAEpaeTARAAPEI-5, Unigene10428\_TAEpaeTARAAPEI-5, Unigene10430\_TAEpaeTARAAPEI-5, Unigene10435\_TAEpaeTARAAPEI-5, Unigene10460\_TAEpaeTARAAPEI-5, Unigene10487\_TAEpaeTARAAPEI-5, Unigene10492\_TAEpaeTARAAPEI-5, Unigene10528\_TAEpaeTARAAPEI-5, Unigene105\_TAEpaeTARAAPEI-5, Unigene10630\_TAEpaeTARAAPEI-5, Unigene10639\_TAEpaeTARAAPEI-5, Unigene10647\_TAEpaeTARAAPEI-5, Unigene10670\_TAEpaeTARAAPEI-5, Unigene10683\_TAEpaeTARAAPEI-5, Unigene10705\_TAEpaeTARAAPEI-5, Unigene10713\_TAEpaeTARAAPEI-5, Unigene10742\_TAEpaeTARAAPEI-5, Unigene10807\_TAEpaeTARAAPEI-5, Unigene10808\_TAEpaeTARAAPEI-5, Unigene10837\_TAEpaeTARAAPEI-5, Unigene10838\_TAEpaeTARAAPEI-5, Unigene10847\_TAEpaeTARAAPEI-5, Unigene10871\_TAEpaeTARAAPEI-5, Unigene10879\_TAEpaeTARAAPEI-5, Unigene10884\_TAEpaeTARAAPEI-5, Unigene10885\_TAEpaeTARAAPEI-5, Unigene10892\_TAEpaeTARAAPEI-5, Unigene10942\_TAEpaeTARAAPEI-5, Unigene10956\_TAEpaeTARAAPEI-5, Unigene10975\_TAEpaeTARAAPEI-5, Unigene10983\_TAEpaeTARAAPEI-5, Unigene10989\_TAEpaeTARAAPEI-5, Unigene11027\_TAEpaeTARAAPEI-5, Unigene1125\_TAEpaeTARAAPEI-5, Unigene11314\_TAEpaeTARAAPEI-5, Unigene11331\_TAEpaeTARAAPEI-5, Unigene11354\_TAEpaeTARAAPEI-5, Unigene11359\_TAEpaeTARAAPEI-5, Unigene11368\_TAEpaeTARAAPEI-5, Unigene1139\_TAEpaeTARAAPEI-5, Unigene11418\_TAEpaeTARAAPEI-5, Unigene11460\_TAEpaeTARAAPEI-5, Unigene11502\_TAEpaeTARAAPEI-5, Unigene11540\_TAEpaeTARAAPEI-5, Unigene11597\_TAEpaeTARAAPEI-5, Unigene11598\_TAEpaeTARAAPEI-5, Unigene11659\_TAEpaeTARAAPEI-5, Unigene11689\_TAEpaeTARAAPEI-5, Unigene116\_TAEpaeTARAAPEI-5, Unigene11721\_TAEpaeTARAAPEI-5, Unigene11775\_TAEpaeTARAAPEI-5, Unigene1178\_TAEpaeTARAAPEI-5, Unigene11851\_TAEpaeTARAAPEI-5, Unigene11869\_TAEpaeTARAAPEI-5, Unigene11900\_TAEpaeTARAAPEI-5, Unigene11902\_TAEpaeTARAAPEI-5, Unigene11905\_TAEpaeTARAAPEI-5, Unigene11956\_TAEpaeTARAAPEI-5, Unigene11964\_TAEpaeTARAAPEI-5, Unigene11982\_TAEpaeTARAAPEI-5, Unigene12008\_TAEpaeTARAAPEI-5, Unigene12078\_TAEpaeTARAAPEI-5, Unigene12090\_TAEpaeTARAAPEI-5, Unigene12128\_TAEpaeTARAAPEI-5, Unigene12151\_TAEpaeTARAAPEI-5, Unigene12173\_TAEpaeTARAAPEI-5, Unigene12180\_TAEpaeTARAAPEI-5, Unigene1226\_TAEpaeTARAAPEI-5, Unigene12277\_TAEpaeTARAAPEI-5, Unigene12290\_TAEpaeTARAAPEI-5, Unigene12301\_TAEpaeTARAAPEI-5, Unigene12387\_TAEpaeTARAAPEI-5, Unigene12396\_TAEpaeTARAAPEI-5, Unigene12408\_TAEpaeTARAAPEI-5, Unigene12417\_TAEpaeTARAAPEI-5, Unigene12420\_TAEpaeTARAAPEI-5, Unigene12444\_TAEpaeTARAAPEI-5, Unigene1249\_TAEpaeTARAAPEI-5, Unigene12538\_TAEpaeTARAAPEI-5, Unigene12557\_TAEpaeTARAAPEI-5, Unigene12569\_TAEpaeTARAAPEI-5, Unigene12608\_TAEpaeTARAAPEI-5, Unigene12664\_TAEpaeTARAAPEI-5, Unigene12718\_TAEpaeTARAAPEI-5, Unigene12753\_TAEpaeTARAAPEI-5, Unigene12756\_TAEpaeTARAAPEI-5, Unigene12765\_TAEpaeTARAAPEI-5, Unigene12805\_TAEpaeTARAAPEI-5, Unigene12830\_TAEpaeTARAAPEI-5, Unigene12834\_TAEpaeTARAAPEI-5, Unigene12885\_TAEpaeTARAAPEI-5, Unigene1289\_TAEpaeTARAAPEI-5, Unigene12903\_TAEpaeTARAAPEI-5, Unigene1300\_TAEpaeTARAAPEI-5, Unigene13014\_TAEpaeTARAAPEI-5, Unigene13042\_TAEpaeTARAAPEI-5, Unigene13061\_TAEpaeTARAAPEI-5, Unigene13063\_TAEpaeTARAAPEI-5, Unigene13066\_TAEpaeTARAAPEI-5, Unigene13086\_TAEpaeTARAAPEI-5, Unigene13139\_TAEpaeTARAAPEI-5, Unigene13162\_TAEpaeTARAAPEI-5, Unigene13180\_TAEpaeTARAAPEI-5, Unigene13245\_TAEpaeTARAAPEI-5, Unigene13250\_TAEpaeTARAAPEI-5, Unigene13267\_TAEpaeTARAAPEI-5, Unigene13288\_TAEpaeTARAAPEI-5, Unigene13298\_TAEpaeTARAAPEI-5, Unigene13315\_TAEpaeTARAAPEI-5, Unigene13369\_TAEpaeTARAAPEI-5, Unigene13474\_TAEpaeTARAAPEI-5, Unigene13485\_TAEpaeTARAAPEI-5, Unigene1349\_TAEpaeTARAAPEI-5, Unigene134\_TAEpaeTARAAPEI-5, Unigene13546\_TAEpaeTARAAPEI-5, Unigene13547\_TAEpaeTARAAPEI-5, Unigene13555\_TAEpaeTARAAPEI-5, Unigene13576\_TAEpaeTARAAPEI-5, Unigene13630\_TAEpaeTARAAPEI-5, Unigene13634\_TAEpaeTARAAPEI-5, Unigene13717\_TAEpaeTARAAPEI-5, Unigene1371\_TAEpaeTARAAPEI-5, Unigene13763\_TAEpaeTARAAPEI-5, Unigene13812\_TAEpaeTARAAPEI-5, Unigene13822\_TAEpaeTARAAPEI-5, Unigene13842\_TAEpaeTARAAPEI-5, Unigene13869\_TAEpaeTARAAPEI-5, Unigene13913\_TAEpaeTARAAPEI-5, Unigene13956\_TAEpaeTARAAPEI-5, Unigene1395\_TAEpaeTARAAPEI-5, Unigene1403\_TAEpaeTARAAPEI-5, Unigene14057\_TAEpaeTARAAPEI-5, Unigene14087\_TAEpaeTARAAPEI-5, Unigene14095\_TAEpaeTARAAPEI-5, Unigene14098\_TAEpaeTARAAPEI-5, Unigene14105\_TAEpaeTARAAPEI-5, Unigene14136\_TAEpaeTARAAPEI-5, Unigene14138\_TAEpaeTARAAPEI-5, Unigene14154\_TAEpaeTARAAPEI-5, Unigene14247\_TAEpaeTARAAPEI-5, Unigene14264\_TAEpaeTARAAPEI-5, Unigene14285\_TAEpaeTARAAPEI-5, Unigene14330\_TAEpaeTARAAPEI-5, Unigene14383\_TAEpaeTARAAPEI-5, Unigene14388\_TAEpaeTARAAPEI-5, Unigene14394\_TAEpaeTARAAPEI-5, Unigene14395\_TAEpaeTARAAPEI-5, Unigene14408\_TAEpaeTARAAPEI-5, Unigene14411\_TAEpaeTARAAPEI-5, Unigene1442\_TAEpaeTARAAPEI-5, Unigene14433\_TAEpaeTARAAPEI-5, Unigene14434\_TAEpaeTARAAPEI-5, Unigene14480\_TAEpaeTARAAPEI-5, Unigene1452\_TAEpaeTARAAPEI-5, Unigene14534\_TAEpaeTARAAPEI-5, Unigene14581\_TAEpaeTARAAPEI-5, Unigene14631\_TAEpaeTARAAPEI-5, Unigene14663\_TAEpaeTARAAPEI-5, Unigene14675\_TAEpaeTARAAPEI-5, Unigene14676\_TAEpaeTARAAPEI-5, Unigene14678\_TAEpaeTARAAPEI-5, Unigene14701\_TAEpaeTARAAPEI-5, Unigene14717\_TAEpaeTARAAPEI-5, Unigene14724\_TAEpaeTARAAPEI-5, Unigene14741\_TAEpaeTARAAPEI-5, Unigene1475\_TAEpaeTARAAPEI-5, Unigene14778\_TAEpaeTARAAPEI-5, Unigene1478\_TAEpaeTARAAPEI-5, Unigene14793\_TAEpaeTARAAPEI-5, Unigene14794\_TAEpaeTARAAPEI-5, Unigene14842\_TAEpaeTARAAPEI-5, Unigene14847\_TAEpaeTARAAPEI-5, Unigene14852\_TAEpaeTARAAPEI-5, Unigene14860\_TAEpaeTARAAPEI-5, Unigene14923\_TAEpaeTARAAPEI-5, Unigene14928\_TAEpaeTARAAPEI-5, Unigene14940\_TAEpaeTARAAPEI-5, Unigene14951\_TAEpaeTARAAPEI-5, Unigene14955\_TAEpaeTARAAPEI-5, Unigene14967\_TAEpaeTARAAPEI-5, Unigene15054\_TAEpaeTARAAPEI-5, Unigene15061\_TAEpaeTARAAPEI-5, Unigene15078\_TAEpaeTARAAPEI-5, Unigene15096\_TAEpaeTARAAPEI-5, Unigene15120\_TAEpaeTARAAPEI-5, Unigene1512\_TAEpaeTARAAPEI-5, Unigene15142\_TAEpaeTARAAPEI-5, Unigene15165\_TAEpaeTARAAPEI-5, Unigene15181\_TAEpaeTARAAPEI-5, Unigene15232\_TAEpaeTARAAPEI-5, Unigene15242\_TAEpaeTARAAPEI-5, Unigene1524\_TAEpaeTARAAPEI-5, Unigene15368\_TAEpaeTARAAPEI-5, Unigene15449\_TAEpaeTARAAPEI-5, Unigene15483\_TAEpaeTARAAPEI-5, Unigene15497\_TAEpaeTARAAPEI-5, Unigene15511\_TAEpaeTARAAPEI-5, Unigene15537\_TAEpaeTARAAPEI-5, Unigene15545\_TAEpaeTARAAPEI-5, Unigene15550\_TAEpaeTARAAPEI-5, Unigene15553\_TAEpaeTARAAPEI-5, Unigene15566\_TAEpaeTARAAPEI-5, Unigene15571\_TAEpaeTARAAPEI-5, Unigene15574\_TAEpaeTARAAPEI-5, Unigene15610\_TAEpaeTARAAPEI-5, Unigene15614\_TAEpaeTARAAPEI-5, Unigene15622\_TAEpaeTARAAPEI-5, Unigene15636\_TAEpaeTARAAPEI-5, Unigene1563\_TAEpaeTARAAPEI-5, Unigene15668\_TAEpaeTARAAPEI-5, Unigene15689\_TAEpaeTARAAPEI-5, Unigene15713\_TAEpaeTARAAPEI-5, Unigene15738\_TAEpaeTARAAPEI-5, Unigene15819\_TAEpaeTARAAPEI-5, Unigene15844\_TAEpaeTARAAPEI-5, Unigene1588\_TAEpaeTARAAPEI-5, Unigene15941\_TAEpaeTARAAPEI-5, Unigene15946\_TAEpaeTARAAPEI-5, Unigene15975\_TAEpaeTARAAPEI-5, Unigene15976\_TAEpaeTARAAPEI-5, Unigene15995\_TAEpaeTARAAPEI-5, Unigene15997\_TAEpaeTARAAPEI-5, Unigene159\_TAEpaeTARAAPEI-5, Unigene1606\_TAEpaeTARAAPEI-5, Unigene16070\_TAEpaeTARAAPEI-5, Unigene16071\_TAEpaeTARAAPEI-5, Unigene160\_TAEpaeTARAAPEI-5, Unigene16104\_TAEpaeTARAAPEI-5, Unigene16124\_TAEpaeTARAAPEI-5, Unigene1612\_TAEpaeTARAAPEI-5, Unigene16140\_TAEpaeTARAAPEI-5, Unigene16150\_TAEpaeTARAAPEI-5, Unigene16189\_TAEpaeTARAAPEI-5, Unigene16219\_TAEpaeTARAAPEI-5, Unigene16230\_TAEpaeTARAAPEI-5, Unigene16243\_TAEpaeTARAAPEI-5, Unigene16250\_TAEpaeTARAAPEI-5, Unigene16263\_TAEpaeTARAAPEI-5, Unigene16264\_TAEpaeTARAAPEI-5, Unigene16265\_TAEpaeTARAAPEI-5, Unigene16268\_TAEpaeTARAAPEI-5, Unigene16318\_TAEpaeTARAAPEI-5, Unigene16331\_TAEpaeTARAAPEI-5, Unigene16356\_TAEpaeTARAAPEI-5, Unigene16384\_TAEpaeTARAAPEI-5, Unigene16389\_TAEpaeTARAAPEI-5, Unigene16412\_TAEpaeTARAAPEI-5, Unigene16426\_TAEpaeTARAAPEI-5, Unigene16441\_TAEpaeTARAAPEI-5, Unigene16446\_TAEpaeTARAAPEI-5, Unigene16455\_TAEpaeTARAAPEI-5, Unigene16495\_TAEpaeTARAAPEI-5, Unigene16550\_TAEpaeTARAAPEI-5, Unigene16602\_TAEpaeTARAAPEI-5, Unigene16612\_TAEpaeTARAAPEI-5, Unigene16621\_TAEpaeTARAAPEI-5, Unigene16623\_TAEpaeTARAAPEI-5, Unigene16625\_TAEpaeTARAAPEI-5, Unigene16662\_TAEpaeTARAAPEI-5, Unigene16678\_TAEpaeTARAAPEI-5, Unigene16682\_TAEpaeTARAAPEI-5, Unigene16706\_TAEpaeTARAAPEI-5, Unigene16711\_TAEpaeTARAAPEI-5, Unigene16717\_TAEpaeTARAAPEI-5, Unigene16735\_TAEpaeTARAAPEI-5, Unigene16743\_TAEpaeTARAAPEI-5, Unigene16749\_TAEpaeTARAAPEI-5, Unigene16770\_TAEpaeTARAAPEI-5, Unigene16780\_TAEpaeTARAAPEI-5, Unigene16798\_TAEpaeTARAAPEI-5, Unigene167\_TAEpaeTARAAPEI-5, Unigene16861\_TAEpaeTARAAPEI-5, Unigene16921\_TAEpaeTARAAPEI-5, Unigene16985\_TAEpaeTARAAPEI-5, Unigene17016\_TAEpaeTARAAPEI-5, Unigene17034\_TAEpaeTARAAPEI-5, Unigene17071\_TAEpaeTARAAPEI-5, Unigene17108\_TAEpaeTARAAPEI-5, Unigene1710\_TAEpaeTARAAPEI-5, Unigene17113\_TAEpaeTARAAPEI-5, Unigene17130\_TAEpaeTARAAPEI-5, Unigene17137\_TAEpaeTARAAPEI-5, Unigene17160\_TAEpaeTARAAPEI-5, Unigene17172\_TAEpaeTARAAPEI-5, Unigene171\_TAEpaeTARAAPEI-5, Unigene17201\_TAEpaeTARAAPEI-5, Unigene17214\_TAEpaeTARAAPEI-5, Unigene17241\_TAEpaeTARAAPEI-5, Unigene17268\_TAEpaeTARAAPEI-5, Unigene17301\_TAEpaeTARAAPEI-5, Unigene17308\_TAEpaeTARAAPEI-5, Unigene17316\_TAEpaeTARAAPEI-5, Unigene17326\_TAEpaeTARAAPEI-5, Unigene1732\_TAEpaeTARAAPEI-5, Unigene17374\_TAEpaeTARAAPEI-5, Unigene17389\_TAEpaeTARAAPEI-5, Unigene17390\_TAEpaeTARAAPEI-5, Unigene17396\_TAEpaeTARAAPEI-5, Unigene17404\_TAEpaeTARAAPEI-5, Unigene1741\_TAEpaeTARAAPEI-5, Unigene17434\_TAEpaeTARAAPEI-5, Unigene17439\_TAEpaeTARAAPEI-5, Unigene17440\_TAEpaeTARAAPEI-5, Unigene17487\_TAEpaeTARAAPEI-5, Unigene17521\_TAEpaeTARAAPEI-5, Unigene17523\_TAEpaeTARAAPEI-5, Unigene17529\_TAEpaeTARAAPEI-5, Unigene1754\_TAEpaeTARAAPEI-5, Unigene17558\_TAEpaeTARAAPEI-5, Unigene17564\_TAEpaeTARAAPEI-5, Unigene17614\_TAEpaeTARAAPEI-5, Unigene17630\_TAEpaeTARAAPEI-5, Unigene17631\_TAEpaeTARAAPEI-5, Unigene17634\_TAEpaeTARAAPEI-5, Unigene17653\_TAEpaeTARAAPEI-5, Unigene17675\_TAEpaeTARAAPEI-5, Unigene17679\_TAEpaeTARAAPEI-5, Unigene17737\_TAEpaeTARAAPEI-5, Unigene17753\_TAEpaeTARAAPEI-5, Unigene17774\_TAEpaeTARAAPEI-5, Unigene17802\_TAEpaeTARAAPEI-5, Unigene17814\_TAEpaeTARAAPEI-5, Unigene17862\_TAEpaeTARAAPEI-5, Unigene17866\_TAEpaeTARAAPEI-5, Unigene17870\_TAEpaeTARAAPEI-5, Unigene17893\_TAEpaeTARAAPEI-5, Unigene17920\_TAEpaeTARAAPEI-5, Unigene1795\_TAEpaeTARAAPEI-5, Unigene17972\_TAEpaeTARAAPEI-5, Unigene17980\_TAEpaeTARAAPEI-5, Unigene17981\_TAEpaeTARAAPEI-5, Unigene18000\_TAEpaeTARAAPEI-5, Unigene18014\_TAEpaeTARAAPEI-5, Unigene18019\_TAEpaeTARAAPEI-5, Unigene1806\_TAEpaeTARAAPEI-5, Unigene18087\_TAEpaeTARAAPEI-5, Unigene18091\_TAEpaeTARAAPEI-5, Unigene18151\_TAEpaeTARAAPEI-5, Unigene18175\_TAEpaeTARAAPEI-5, Unigene18274\_TAEpaeTARAAPEI-5, Unigene18276\_TAEpaeTARAAPEI-5, Unigene18333\_TAEpaeTARAAPEI-5, Unigene1833\_TAEpaeTARAAPEI-5, Unigene1839\_TAEpaeTARAAPEI-5, Unigene18431\_TAEpaeTARAAPEI-5, Unigene18544\_TAEpaeTARAAPEI-5, Unigene18604\_TAEpaeTARAAPEI-5, Unigene18652\_TAEpaeTARAAPEI-5, Unigene18721\_TAEpaeTARAAPEI-5, Unigene18764\_TAEpaeTARAAPEI-5, Unigene18765\_TAEpaeTARAAPEI-5, Unigene18772\_TAEpaeTARAAPEI-5, Unigene18941\_TAEpaeTARAAPEI-5, Unigene1901\_TAEpaeTARAAPEI-5, Unigene19042\_TAEpaeTARAAPEI-5, Unigene19111\_TAEpaeTARAAPEI-5, Unigene19114\_TAEpaeTARAAPEI-5, Unigene19126\_TAEpaeTARAAPEI-5, Unigene19212\_TAEpaeTARAAPEI-5, Unigene19225\_TAEpaeTARAAPEI-5, Unigene19232\_TAEpaeTARAAPEI-5, Unigene19241\_TAEpaeTARAAPEI-5, Unigene19267\_TAEpaeTARAAPEI-5, Unigene19353\_TAEpaeTARAAPEI-5, Unigene19367\_TAEpaeTARAAPEI-5, Unigene19430\_TAEpaeTARAAPEI-5, Unigene19448\_TAEpaeTARAAPEI-5, Unigene19481\_TAEpaeTARAAPEI-5, Unigene1953\_TAEpaeTARAAPEI-5, Unigene19588\_TAEpaeTARAAPEI-5, Unigene1968\_TAEpaeTARAAPEI-5, Unigene19768\_TAEpaeTARAAPEI-5, Unigene19774\_TAEpaeTARAAPEI-5, Unigene19791\_TAEpaeTARAAPEI-5, Unigene19975\_TAEpaeTARAAPEI-5, Unigene1\_TAEpaeTARAAPEI-5, Unigene20050\_TAEpaeTARAAPEI-5, Unigene20065\_TAEpaeTARAAPEI-5, Unigene20089\_TAEpaeTARAAPEI-5, Unigene20124\_TAEpaeTARAAPEI-5, Unigene20158\_TAEpaeTARAAPEI-5, Unigene2016\_TAEpaeTARAAPEI-5, Unigene20172\_TAEpaeTARAAPEI-5, Unigene2019\_TAEpaeTARAAPEI-5, Unigene20226\_TAEpaeTARAAPEI-5, Unigene20265\_TAEpaeTARAAPEI-5, Unigene20280\_TAEpaeTARAAPEI-5, Unigene20369\_TAEpaeTARAAPEI-5, Unigene20426\_TAEpaeTARAAPEI-5, Unigene20502\_TAEpaeTARAAPEI-5, Unigene20503\_TAEpaeTARAAPEI-5, Unigene20575\_TAEpaeTARAAPEI-5, Unigene20583\_TAEpaeTARAAPEI-5, Unigene20632\_TAEpaeTARAAPEI-5, Unigene20638\_TAEpaeTARAAPEI-5, Unigene20745\_TAEpaeTARAAPEI-5, Unigene20828\_TAEpaeTARAAPEI-5, Unigene20831\_TAEpaeTARAAPEI-5, Unigene20845\_TAEpaeTARAAPEI-5, Unigene20858\_TAEpaeTARAAPEI-5, Unigene208\_TAEpaeTARAAPEI-5, Unigene20906\_TAEpaeTARAAPEI-5, Unigene20940\_TAEpaeTARAAPEI-5, Unigene21056\_TAEpaeTARAAPEI-5, Unigene21093\_TAEpaeTARAAPEI-5, Unigene21099\_TAEpaeTARAAPEI-5, Unigene2109\_TAEpaeTARAAPEI-5, Unigene21150\_TAEpaeTARAAPEI-5, Unigene21154\_TAEpaeTARAAPEI-5, Unigene21278\_TAEpaeTARAAPEI-5, Unigene21299\_TAEpaeTARAAPEI-5, Unigene2135\_TAEpaeTARAAPEI-5, Unigene21408\_TAEpaeTARAAPEI-5, Unigene21511\_TAEpaeTARAAPEI-5, Unigene21575\_TAEpaeTARAAPEI-5, Unigene21586\_TAEpaeTARAAPEI-5, Unigene2159\_TAEpaeTARAAPEI-5, Unigene21707\_TAEpaeTARAAPEI-5, Unigene21736\_TAEpaeTARAAPEI-5, Unigene21831\_TAEpaeTARAAPEI-5, Unigene21846\_TAEpaeTARAAPEI-5, Unigene21858\_TAEpaeTARAAPEI-5, Unigene21903\_TAEpaeTARAAPEI-5, Unigene21911\_TAEpaeTARAAPEI-5, Unigene21960\_TAEpaeTARAAPEI-5, Unigene21967\_TAEpaeTARAAPEI-5, Unigene22008\_TAEpaeTARAAPEI-5, Unigene22045\_TAEpaeTARAAPEI-5, Unigene22113\_TAEpaeTARAAPEI-5, Unigene22169\_TAEpaeTARAAPEI-5, Unigene22180\_TAEpaeTARAAPEI-5, Unigene22182\_TAEpaeTARAAPEI-5, Unigene22195\_TAEpaeTARAAPEI-5, Unigene22196\_TAEpaeTARAAPEI-5, Unigene22205\_TAEpaeTARAAPEI-5, Unigene22237\_TAEpaeTARAAPEI-5, Unigene22367\_TAEpaeTARAAPEI-5, Unigene22472\_TAEpaeTARAAPEI-5, Unigene22526\_TAEpaeTARAAPEI-5, Unigene22535\_TAEpaeTARAAPEI-5, Unigene22557\_TAEpaeTARAAPEI-5, Unigene22561\_TAEpaeTARAAPEI-5, Unigene22592\_TAEpaeTARAAPEI-5, Unigene22657\_TAEpaeTARAAPEI-5, Unigene22704\_TAEpaeTARAAPEI-5, Unigene22856\_TAEpaeTARAAPEI-5, Unigene22906\_TAEpaeTARAAPEI-5, Unigene22931\_TAEpaeTARAAPEI-5, Unigene22944\_TAEpaeTARAAPEI-5, Unigene22974\_TAEpaeTARAAPEI-5, Unigene22982\_TAEpaeTARAAPEI-5, Unigene23003\_TAEpaeTARAAPEI-5, Unigene23099\_TAEpaeTARAAPEI-5, Unigene23157\_TAEpaeTARAAPEI-5, Unigene23181\_TAEpaeTARAAPEI-5, Unigene23183\_TAEpaeTARAAPEI-5, Unigene23210\_TAEpaeTARAAPEI-5, Unigene23254\_TAEpaeTARAAPEI-5, Unigene23368\_TAEpaeTARAAPEI-5, Unigene23446\_TAEpaeTARAAPEI-5, Unigene234\_TAEpaeTARAAPEI-5, Unigene23518\_TAEpaeTARAAPEI-5, Unigene23530\_TAEpaeTARAAPEI-5, Unigene23645\_TAEpaeTARAAPEI-5, Unigene23647\_TAEpaeTARAAPEI-5, Unigene23651\_TAEpaeTARAAPEI-5, Unigene23701\_TAEpaeTARAAPEI-5, Unigene23721\_TAEpaeTARAAPEI-5, Unigene23745\_TAEpaeTARAAPEI-5, Unigene23751\_TAEpaeTARAAPEI-5, Unigene23761\_TAEpaeTARAAPEI-5, Unigene23770\_TAEpaeTARAAPEI-5, Unigene23804\_TAEpaeTARAAPEI-5, Unigene23817\_TAEpaeTARAAPEI-5, Unigene23872\_TAEpaeTARAAPEI-5, Unigene23885\_TAEpaeTARAAPEI-5, Unigene23887\_TAEpaeTARAAPEI-5, Unigene2388\_TAEpaeTARAAPEI-5, Unigene23948\_TAEpaeTARAAPEI-5, Unigene23955\_TAEpaeTARAAPEI-5, Unigene23970\_TAEpaeTARAAPEI-5, Unigene23997\_TAEpaeTARAAPEI-5, Unigene23999\_TAEpaeTARAAPEI-5, Unigene24039\_TAEpaeTARAAPEI-5, Unigene24146\_TAEpaeTARAAPEI-5, Unigene24169\_TAEpaeTARAAPEI-5, Unigene24217\_TAEpaeTARAAPEI-5, Unigene2421\_TAEpaeTARAAPEI-5, Unigene24236\_TAEpaeTARAAPEI-5, Unigene24300\_TAEpaeTARAAPEI-5, Unigene24335\_TAEpaeTARAAPEI-5, Unigene24407\_TAEpaeTARAAPEI-5, Unigene2453\_TAEpaeTARAAPEI-5, Unigene24548\_TAEpaeTARAAPEI-5, Unigene24556\_TAEpaeTARAAPEI-5, Unigene24570\_TAEpaeTARAAPEI-5, Unigene24643\_TAEpaeTARAAPEI-5, Unigene24659\_TAEpaeTARAAPEI-5, Unigene24729\_TAEpaeTARAAPEI-5, Unigene2474\_TAEpaeTARAAPEI-5, Unigene24773\_TAEpaeTARAAPEI-5, Unigene24831\_TAEpaeTARAAPEI-5, Unigene2488\_TAEpaeTARAAPEI-5, Unigene24924\_TAEpaeTARAAPEI-5, Unigene25071\_TAEpaeTARAAPEI-5, Unigene2508\_TAEpaeTARAAPEI-5, Unigene25129\_TAEpaeTARAAPEI-5, Unigene25195\_TAEpaeTARAAPEI-5, Unigene25215\_TAEpaeTARAAPEI-5, Unigene25261\_TAEpaeTARAAPEI-5, Unigene25269\_TAEpaeTARAAPEI-5, Unigene25277\_TAEpaeTARAAPEI-5, Unigene25288\_TAEpaeTARAAPEI-5, Unigene25342\_TAEpaeTARAAPEI-5, Unigene25365\_TAEpaeTARAAPEI-5, Unigene25368\_TAEpaeTARAAPEI-5, Unigene2536\_TAEpaeTARAAPEI-5, Unigene25387\_TAEpaeTARAAPEI-5, Unigene25415\_TAEpaeTARAAPEI-5, Unigene25430\_TAEpaeTARAAPEI-5, Unigene25456\_TAEpaeTARAAPEI-5, Unigene25464\_TAEpaeTARAAPEI-5, Unigene25481\_TAEpaeTARAAPEI-5, Unigene25555\_TAEpaeTARAAPEI-5, Unigene25592\_TAEpaeTARAAPEI-5, Unigene25610\_TAEpaeTARAAPEI-5, Unigene25611\_TAEpaeTARAAPEI-5, Unigene25709\_TAEpaeTARAAPEI-5, Unigene25745\_TAEpaeTARAAPEI-5, Unigene25770\_TAEpaeTARAAPEI-5, Unigene25819\_TAEpaeTARAAPEI-5, Unigene25886\_TAEpaeTARAAPEI-5, Unigene25889\_TAEpaeTARAAPEI-5, Unigene25985\_TAEpaeTARAAPEI-5, Unigene25998\_TAEpaeTARAAPEI-5, Unigene26004\_TAEpaeTARAAPEI-5, Unigene26078\_TAEpaeTARAAPEI-5, Unigene26100\_TAEpaeTARAAPEI-5, Unigene26124\_TAEpaeTARAAPEI-5, Unigene26135\_TAEpaeTARAAPEI-5, Unigene2613\_TAEpaeTARAAPEI-5, Unigene26199\_TAEpaeTARAAPEI-5, Unigene26219\_TAEpaeTARAAPEI-5, Unigene26231\_TAEpaeTARAAPEI-5, Unigene26251\_TAEpaeTARAAPEI-5, Unigene26278\_TAEpaeTARAAPEI-5, Unigene26296\_TAEpaeTARAAPEI-5, Unigene26304\_TAEpaeTARAAPEI-5, Unigene26351\_TAEpaeTARAAPEI-5, Unigene26403\_TAEpaeTARAAPEI-5, Unigene26404\_TAEpaeTARAAPEI-5, Unigene26425\_TAEpaeTARAAPEI-5, Unigene26463\_TAEpaeTARAAPEI-5, Unigene26531\_TAEpaeTARAAPEI-5, Unigene26534\_TAEpaeTARAAPEI-5, Unigene2657\_TAEpaeTARAAPEI-5, Unigene26606\_TAEpaeTARAAPEI-5, Unigene26623\_TAEpaeTARAAPEI-5, Unigene26669\_TAEpaeTARAAPEI-5, Unigene26782\_TAEpaeTARAAPEI-5, Unigene26939\_TAEpaeTARAAPEI-5, Unigene27023\_TAEpaeTARAAPEI-5, Unigene27048\_TAEpaeTARAAPEI-5, Unigene27098\_TAEpaeTARAAPEI-5, Unigene27180\_TAEpaeTARAAPEI-5, Unigene27267\_TAEpaeTARAAPEI-5, Unigene27293\_TAEpaeTARAAPEI-5, Unigene27386\_TAEpaeTARAAPEI-5, Unigene27469\_TAEpaeTARAAPEI-5, Unigene27478\_TAEpaeTARAAPEI-5, Unigene27491\_TAEpaeTARAAPEI-5, Unigene27541\_TAEpaeTARAAPEI-5, Unigene27553\_TAEpaeTARAAPEI-5, Unigene27582\_TAEpaeTARAAPEI-5, Unigene27593\_TAEpaeTARAAPEI-5, Unigene275\_TAEpaeTARAAPEI-5, Unigene27629\_TAEpaeTARAAPEI-5, Unigene27656\_TAEpaeTARAAPEI-5, Unigene27668\_TAEpaeTARAAPEI-5, Unigene27680\_TAEpaeTARAAPEI-5, Unigene27897\_TAEpaeTARAAPEI-5, Unigene27931\_TAEpaeTARAAPEI-5, Unigene27981\_TAEpaeTARAAPEI-5, Unigene27994\_TAEpaeTARAAPEI-5, Unigene27\_TAEpaeTARAAPEI-5, Unigene28053\_TAEpaeTARAAPEI-5, Unigene2812\_TAEpaeTARAAPEI-5, Unigene28191\_TAEpaeTARAAPEI-5, Unigene28252\_TAEpaeTARAAPEI-5, Unigene28257\_TAEpaeTARAAPEI-5, Unigene28263\_TAEpaeTARAAPEI-5, Unigene28273\_TAEpaeTARAAPEI-5, Unigene28349\_TAEpaeTARAAPEI-5, Unigene28368\_TAEpaeTARAAPEI-5, Unigene28428\_TAEpaeTARAAPEI-5, Unigene28475\_TAEpaeTARAAPEI-5, Unigene28492\_TAEpaeTARAAPEI-5, Unigene28506\_TAEpaeTARAAPEI-5, Unigene28507\_TAEpaeTARAAPEI-5, Unigene2852\_TAEpaeTARAAPEI-5, Unigene28534\_TAEpaeTARAAPEI-5, Unigene28615\_TAEpaeTARAAPEI-5, Unigene28631\_TAEpaeTARAAPEI-5, Unigene28660\_TAEpaeTARAAPEI-5, Unigene28792\_TAEpaeTARAAPEI-5, Unigene28844\_TAEpaeTARAAPEI-5, Unigene28876\_TAEpaeTARAAPEI-5, Unigene28988\_TAEpaeTARAAPEI-5, Unigene29044\_TAEpaeTARAAPEI-5, Unigene29097\_TAEpaeTARAAPEI-5, Unigene29132\_TAEpaeTARAAPEI-5, Unigene29139\_TAEpaeTARAAPEI-5, Unigene29158\_TAEpaeTARAAPEI-5, Unigene29178\_TAEpaeTARAAPEI-5, Unigene29193\_TAEpaeTARAAPEI-5, Unigene29272\_TAEpaeTARAAPEI-5, Unigene29286\_TAEpaeTARAAPEI-5, Unigene29295\_TAEpaeTARAAPEI-5, Unigene29301\_TAEpaeTARAAPEI-5, Unigene2936\_TAEpaeTARAAPEI-5, Unigene29381\_TAEpaeTARAAPEI-5, Unigene29393\_TAEpaeTARAAPEI-5, Unigene293\_TAEpaeTARAAPEI-5, Unigene29417\_TAEpaeTARAAPEI-5, Unigene29444\_TAEpaeTARAAPEI-5, Unigene29460\_TAEpaeTARAAPEI-5, Unigene29470\_TAEpaeTARAAPEI-5, Unigene29493\_TAEpaeTARAAPEI-5, Unigene29565\_TAEpaeTARAAPEI-5, Unigene2961\_TAEpaeTARAAPEI-5, Unigene29667\_TAEpaeTARAAPEI-5, Unigene2966\_TAEpaeTARAAPEI-5, Unigene29672\_TAEpaeTARAAPEI-5, Unigene29714\_TAEpaeTARAAPEI-5, Unigene29718\_TAEpaeTARAAPEI-5, Unigene29770\_TAEpaeTARAAPEI-5, Unigene29815\_TAEpaeTARAAPEI-5, Unigene29827\_TAEpaeTARAAPEI-5, Unigene29899\_TAEpaeTARAAPEI-5, Unigene29903\_TAEpaeTARAAPEI-5, Unigene30002\_TAEpaeTARAAPEI-5, Unigene30003\_TAEpaeTARAAPEI-5, Unigene30015\_TAEpaeTARAAPEI-5, Unigene30021\_TAEpaeTARAAPEI-5, Unigene30027\_TAEpaeTARAAPEI-5, Unigene30040\_TAEpaeTARAAPEI-5, Unigene30097\_TAEpaeTARAAPEI-5, Unigene300\_TAEpaeTARAAPEI-5, Unigene30101\_TAEpaeTARAAPEI-5, Unigene30102\_TAEpaeTARAAPEI-5, Unigene30135\_TAEpaeTARAAPEI-5, Unigene30161\_TAEpaeTARAAPEI-5, Unigene3019\_TAEpaeTARAAPEI-5, Unigene30316\_TAEpaeTARAAPEI-5, Unigene30325\_TAEpaeTARAAPEI-5, Unigene30412\_TAEpaeTARAAPEI-5, Unigene30497\_TAEpaeTARAAPEI-5, Unigene30502\_TAEpaeTARAAPEI-5, Unigene30505\_TAEpaeTARAAPEI-5, Unigene30508\_TAEpaeTARAAPEI-5, Unigene30536\_TAEpaeTARAAPEI-5, Unigene30537\_TAEpaeTARAAPEI-5, Unigene30548\_TAEpaeTARAAPEI-5, Unigene30562\_TAEpaeTARAAPEI-5, Unigene30573\_TAEpaeTARAAPEI-5, Unigene30581\_TAEpaeTARAAPEI-5, Unigene30605\_TAEpaeTARAAPEI-5, Unigene30698\_TAEpaeTARAAPEI-5, Unigene30828\_TAEpaeTARAAPEI-5, Unigene30861\_TAEpaeTARAAPEI-5, Unigene30869\_TAEpaeTARAAPEI-5, Unigene30961\_TAEpaeTARAAPEI-5, Unigene30986\_TAEpaeTARAAPEI-5, Unigene31007\_TAEpaeTARAAPEI-5, Unigene31016\_TAEpaeTARAAPEI-5, Unigene31093\_TAEpaeTARAAPEI-5, Unigene31114\_TAEpaeTARAAPEI-5, Unigene31119\_TAEpaeTARAAPEI-5, Unigene3113\_TAEpaeTARAAPEI-5, Unigene31169\_TAEpaeTARAAPEI-5, Unigene3116\_TAEpaeTARAAPEI-5, Unigene31344\_TAEpaeTARAAPEI-5, Unigene31361\_TAEpaeTARAAPEI-5, Unigene31412\_TAEpaeTARAAPEI-5, Unigene31425\_TAEpaeTARAAPEI-5, Unigene31492\_TAEpaeTARAAPEI-5, Unigene31494\_TAEpaeTARAAPEI-5, Unigene31525\_TAEpaeTARAAPEI-5, Unigene31560\_TAEpaeTARAAPEI-5, Unigene31697\_TAEpaeTARAAPEI-5, Unigene31709\_TAEpaeTARAAPEI-5, Unigene31740\_TAEpaeTARAAPEI-5, Unigene31841\_TAEpaeTARAAPEI-5, Unigene31923\_TAEpaeTARAAPEI-5, Unigene32050\_TAEpaeTARAAPEI-5, Unigene32054\_TAEpaeTARAAPEI-5, Unigene32059\_TAEpaeTARAAPEI-5, Unigene32122\_TAEpaeTARAAPEI-5, Unigene32142\_TAEpaeTARAAPEI-5, Unigene32164\_TAEpaeTARAAPEI-5, Unigene32196\_TAEpaeTARAAPEI-5, Unigene32285\_TAEpaeTARAAPEI-5, Unigene32338\_TAEpaeTARAAPEI-5, Unigene32368\_TAEpaeTARAAPEI-5, Unigene32384\_TAEpaeTARAAPEI-5, Unigene32393\_TAEpaeTARAAPEI-5, Unigene32425\_TAEpaeTARAAPEI-5, Unigene32508\_TAEpaeTARAAPEI-5, Unigene32518\_TAEpaeTARAAPEI-5, Unigene32567\_TAEpaeTARAAPEI-5, Unigene32570\_TAEpaeTARAAPEI-5, Unigene32582\_TAEpaeTARAAPEI-5, Unigene32583\_TAEpaeTARAAPEI-5, Unigene3261\_TAEpaeTARAAPEI-5, Unigene32665\_TAEpaeTARAAPEI-5, Unigene3268\_TAEpaeTARAAPEI-5, Unigene32717\_TAEpaeTARAAPEI-5, Unigene32729\_TAEpaeTARAAPEI-5, Unigene32734\_TAEpaeTARAAPEI-5, Unigene32755\_TAEpaeTARAAPEI-5, Unigene32814\_TAEpaeTARAAPEI-5, Unigene32864\_TAEpaeTARAAPEI-5, Unigene32875\_TAEpaeTARAAPEI-5, Unigene32934\_TAEpaeTARAAPEI-5, Unigene32986\_TAEpaeTARAAPEI-5, Unigene33092\_TAEpaeTARAAPEI-5, Unigene33137\_TAEpaeTARAAPEI-5, Unigene33158\_TAEpaeTARAAPEI-5, Unigene33164\_TAEpaeTARAAPEI-5, Unigene33175\_TAEpaeTARAAPEI-5, Unigene33183\_TAEpaeTARAAPEI-5, Unigene33288\_TAEpaeTARAAPEI-5, Unigene3331\_TAEpaeTARAAPEI-5, Unigene33327\_TAEpaeTARAAPEI-5, Unigene33377\_TAEpaeTARAAPEI-5, Unigene33391\_TAEpaeTARAAPEI-5, Unigene33422\_TAEpaeTARAAPEI-5, Unigene33506\_TAEpaeTARAAPEI-5, Unigene33507\_TAEpaeTARAAPEI-5, Unigene33516\_TAEpaeTARAAPEI-5, Unigene33530\_TAEpaeTARAAPEI-5, Unigene33549\_TAEpaeTARAAPEI-5, Unigene33558\_TAEpaeTARAAPEI-5, Unigene3355\_TAEpaeTARAAPEI-5, Unigene33584\_TAEpaeTARAAPEI-5, Unigene33609\_TAEpaeTARAAPEI-5, Unigene33618\_TAEpaeTARAAPEI-5, Unigene33638\_TAEpaeTARAAPEI-5, Unigene33691\_TAEpaeTARAAPEI-5, Unigene33710\_TAEpaeTARAAPEI-5, Unigene33747\_TAEpaeTARAAPEI-5, Unigene33751\_TAEpaeTARAAPEI-5, Unigene33796\_TAEpaeTARAAPEI-5, Unigene33860\_TAEpaeTARAAPEI-5, Unigene33862\_TAEpaeTARAAPEI-5, Unigene33935\_TAEpaeTARAAPEI-5, Unigene33966\_TAEpaeTARAAPEI-5, Unigene33973\_TAEpaeTARAAPEI-5, Unigene33995\_TAEpaeTARAAPEI-5, Unigene34000\_TAEpaeTARAAPEI-5, Unigene34024\_TAEpaeTARAAPEI-5, Unigene34043\_TAEpaeTARAAPEI-5, Unigene34117\_TAEpaeTARAAPEI-5, Unigene34120\_TAEpaeTARAAPEI-5, Unigene34176\_TAEpaeTARAAPEI-5, Unigene34212\_TAEpaeTARAAPEI-5, Unigene34254\_TAEpaeTARAAPEI-5, Unigene34262\_TAEpaeTARAAPEI-5, Unigene34285\_TAEpaeTARAAPEI-5, Unigene34343\_TAEpaeTARAAPEI-5, Unigene34366\_TAEpaeTARAAPEI-5, Unigene34440\_TAEpaeTARAAPEI-5, Unigene34449\_TAEpaeTARAAPEI-5, Unigene34488\_TAEpaeTARAAPEI-5, Unigene34594\_TAEpaeTARAAPEI-5, Unigene34606\_TAEpaeTARAAPEI-5, Unigene34694\_TAEpaeTARAAPEI-5, Unigene34699\_TAEpaeTARAAPEI-5, Unigene34739\_TAEpaeTARAAPEI-5, Unigene34814\_TAEpaeTARAAPEI-5, Unigene34824\_TAEpaeTARAAPEI-5, Unigene34838\_TAEpaeTARAAPEI-5, Unigene34870\_TAEpaeTARAAPEI-5, Unigene34877\_TAEpaeTARAAPEI-5, Unigene34882\_TAEpaeTARAAPEI-5, Unigene34891\_TAEpaeTARAAPEI-5, Unigene34972\_TAEpaeTARAAPEI-5, Unigene34975\_TAEpaeTARAAPEI-5, Unigene34998\_TAEpaeTARAAPEI-5, Unigene35001\_TAEpaeTARAAPEI-5, Unigene35037\_TAEpaeTARAAPEI-5, Unigene3514\_TAEpaeTARAAPEI-5, Unigene35153\_TAEpaeTARAAPEI-5, Unigene35156\_TAEpaeTARAAPEI-5, Unigene35198\_TAEpaeTARAAPEI-5, Unigene3521\_TAEpaeTARAAPEI-5, Unigene3528\_TAEpaeTARAAPEI-5, Unigene35305\_TAEpaeTARAAPEI-5, Unigene35313\_TAEpaeTARAAPEI-5, Unigene35321\_TAEpaeTARAAPEI-5, Unigene35335\_TAEpaeTARAAPEI-5, Unigene35360\_TAEpaeTARAAPEI-5, Unigene35387\_TAEpaeTARAAPEI-5, Unigene35393\_TAEpaeTARAAPEI-5, Unigene35436\_TAEpaeTARAAPEI-5, Unigene35553\_TAEpaeTARAAPEI-5, Unigene35577\_TAEpaeTARAAPEI-5, Unigene35619\_TAEpaeTARAAPEI-5, Unigene35653\_TAEpaeTARAAPEI-5, Unigene35671\_TAEpaeTARAAPEI-5, Unigene35739\_TAEpaeTARAAPEI-5, Unigene35777\_TAEpaeTARAAPEI-5, Unigene35793\_TAEpaeTARAAPEI-5, Unigene35825\_TAEpaeTARAAPEI-5, Unigene35827\_TAEpaeTARAAPEI-5, Unigene35858\_TAEpaeTARAAPEI-5, Unigene35909\_TAEpaeTARAAPEI-5, Unigene35919\_TAEpaeTARAAPEI-5, Unigene35932\_TAEpaeTARAAPEI-5, Unigene35981\_TAEpaeTARAAPEI-5, Unigene36056\_TAEpaeTARAAPEI-5, Unigene36094\_TAEpaeTARAAPEI-5, Unigene36117\_TAEpaeTARAAPEI-5, Unigene36123\_TAEpaeTARAAPEI-5, Unigene36129\_TAEpaeTARAAPEI-5, Unigene36152\_TAEpaeTARAAPEI-5, Unigene36260\_TAEpaeTARAAPEI-5, Unigene3628\_TAEpaeTARAAPEI-5, Unigene36319\_TAEpaeTARAAPEI-5, Unigene36412\_TAEpaeTARAAPEI-5, Unigene36413\_TAEpaeTARAAPEI-5, Unigene36489\_TAEpaeTARAAPEI-5, Unigene36524\_TAEpaeTARAAPEI-5, Unigene36566\_TAEpaeTARAAPEI-5, Unigene36608\_TAEpaeTARAAPEI-5, Unigene36612\_TAEpaeTARAAPEI-5, Unigene36693\_TAEpaeTARAAPEI-5, Unigene36801\_TAEpaeTARAAPEI-5, Unigene36803\_TAEpaeTARAAPEI-5, Unigene36830\_TAEpaeTARAAPEI-5, Unigene36839\_TAEpaeTARAAPEI-5, Unigene36848\_TAEpaeTARAAPEI-5, Unigene36865\_TAEpaeTARAAPEI-5, Unigene36893\_TAEpaeTARAAPEI-5, Unigene36929\_TAEpaeTARAAPEI-5, Unigene37026\_TAEpaeTARAAPEI-5, Unigene37031\_TAEpaeTARAAPEI-5, Unigene37057\_TAEpaeTARAAPEI-5, Unigene37067\_TAEpaeTARAAPEI-5, Unigene37134\_TAEpaeTARAAPEI-5, Unigene37139\_TAEpaeTARAAPEI-5, Unigene37141\_TAEpaeTARAAPEI-5, Unigene37156\_TAEpaeTARAAPEI-5, Unigene37169\_TAEpaeTARAAPEI-5, Unigene37262\_TAEpaeTARAAPEI-5, Unigene37278\_TAEpaeTARAAPEI-5, Unigene37367\_TAEpaeTARAAPEI-5, Unigene37373\_TAEpaeTARAAPEI-5, Unigene37392\_TAEpaeTARAAPEI-5, Unigene37426\_TAEpaeTARAAPEI-5, Unigene37448\_TAEpaeTARAAPEI-5, Unigene37487\_TAEpaeTARAAPEI-5, Unigene37518\_TAEpaeTARAAPEI-5, Unigene37539\_TAEpaeTARAAPEI-5, Unigene3759\_TAEpaeTARAAPEI-5, Unigene37637\_TAEpaeTARAAPEI-5, Unigene37652\_TAEpaeTARAAPEI-5, Unigene37657\_TAEpaeTARAAPEI-5, Unigene37681\_TAEpaeTARAAPEI-5, Unigene37687\_TAEpaeTARAAPEI-5, Unigene37816\_TAEpaeTARAAPEI-5, Unigene37840\_TAEpaeTARAAPEI-5, Unigene3788\_TAEpaeTARAAPEI-5, Unigene37930\_TAEpaeTARAAPEI-5, Unigene37941\_TAEpaeTARAAPEI-5, Unigene37956\_TAEpaeTARAAPEI-5, Unigene37977\_TAEpaeTARAAPEI-5, Unigene38001\_TAEpaeTARAAPEI-5, Unigene38044\_TAEpaeTARAAPEI-5, Unigene38125\_TAEpaeTARAAPEI-5, Unigene38135\_TAEpaeTARAAPEI-5, Unigene38179\_TAEpaeTARAAPEI-5, Unigene38214\_TAEpaeTARAAPEI-5, Unigene38223\_TAEpaeTARAAPEI-5, Unigene38272\_TAEpaeTARAAPEI-5, Unigene38285\_TAEpaeTARAAPEI-5, Unigene38295\_TAEpaeTARAAPEI-5, Unigene38339\_TAEpaeTARAAPEI-5, Unigene38390\_TAEpaeTARAAPEI-5, Unigene38430\_TAEpaeTARAAPEI-5, Unigene38441\_TAEpaeTARAAPEI-5, Unigene38446\_TAEpaeTARAAPEI-5, Unigene38519\_TAEpaeTARAAPEI-5, Unigene38549\_TAEpaeTARAAPEI-5, Unigene38618\_TAEpaeTARAAPEI-5, Unigene38644\_TAEpaeTARAAPEI-5, Unigene38661\_TAEpaeTARAAPEI-5, Unigene38716\_TAEpaeTARAAPEI-5, Unigene38787\_TAEpaeTARAAPEI-5, Unigene38801\_TAEpaeTARAAPEI-5, Unigene38863\_TAEpaeTARAAPEI-5, Unigene38890\_TAEpaeTARAAPEI-5, Unigene38892\_TAEpaeTARAAPEI-5, Unigene3889\_TAEpaeTARAAPEI-5, Unigene38939\_TAEpaeTARAAPEI-5, Unigene39004\_TAEpaeTARAAPEI-5, Unigene3901\_TAEpaeTARAAPEI-5, Unigene39031\_TAEpaeTARAAPEI-5, Unigene39041\_TAEpaeTARAAPEI-5, Unigene39048\_TAEpaeTARAAPEI-5, Unigene39223\_TAEpaeTARAAPEI-5, Unigene39224\_TAEpaeTARAAPEI-5, Unigene39247\_TAEpaeTARAAPEI-5, Unigene39301\_TAEpaeTARAAPEI-5, Unigene39396\_TAEpaeTARAAPEI-5, Unigene39453\_TAEpaeTARAAPEI-5, Unigene39522\_TAEpaeTARAAPEI-5, Unigene39531\_TAEpaeTARAAPEI-5, Unigene3953\_TAEpaeTARAAPEI-5, Unigene39546\_TAEpaeTARAAPEI-5, Unigene3954\_TAEpaeTARAAPEI-5, Unigene39558\_TAEpaeTARAAPEI-5, Unigene39559\_TAEpaeTARAAPEI-5, Unigene3961\_TAEpaeTARAAPEI-5, Unigene39680\_TAEpaeTARAAPEI-5, Unigene39683\_TAEpaeTARAAPEI-5, Unigene39700\_TAEpaeTARAAPEI-5, Unigene39716\_TAEpaeTARAAPEI-5, Unigene39829\_TAEpaeTARAAPEI-5, Unigene39850\_TAEpaeTARAAPEI-5, Unigene39855\_TAEpaeTARAAPEI-5, Unigene39867\_TAEpaeTARAAPEI-5, Unigene39885\_TAEpaeTARAAPEI-5, Unigene39914\_TAEpaeTARAAPEI-5, Unigene3991\_TAEpaeTARAAPEI-5, Unigene39937\_TAEpaeTARAAPEI-5, Unigene39976\_TAEpaeTARAAPEI-5, Unigene39978\_TAEpaeTARAAPEI-5, Unigene40014\_TAEpaeTARAAPEI-5, Unigene40048\_TAEpaeTARAAPEI-5, Unigene40074\_TAEpaeTARAAPEI-5, Unigene40148\_TAEpaeTARAAPEI-5, Unigene40155\_TAEpaeTARAAPEI-5, Unigene40205\_TAEpaeTARAAPEI-5, Unigene40225\_TAEpaeTARAAPEI-5, Unigene40243\_TAEpaeTARAAPEI-5, Unigene40244\_TAEpaeTARAAPEI-5, Unigene40252\_TAEpaeTARAAPEI-5, Unigene4030\_TAEpaeTARAAPEI-5, Unigene40504\_TAEpaeTARAAPEI-5, Unigene40505\_TAEpaeTARAAPEI-5, Unigene40526\_TAEpaeTARAAPEI-5, Unigene40569\_TAEpaeTARAAPEI-5, Unigene40617\_TAEpaeTARAAPEI-5, Unigene4069\_TAEpaeTARAAPEI-5, Unigene40700\_TAEpaeTARAAPEI-5, Unigene40703\_TAEpaeTARAAPEI-5, Unigene40716\_TAEpaeTARAAPEI-5, Unigene40719\_TAEpaeTARAAPEI-5, Unigene40734\_TAEpaeTARAAPEI-5, Unigene40760\_TAEpaeTARAAPEI-5, Unigene40806\_TAEpaeTARAAPEI-5, Unigene4087\_TAEpaeTARAAPEI-5, Unigene40911\_TAEpaeTARAAPEI-5, Unigene40941\_TAEpaeTARAAPEI-5, Unigene40977\_TAEpaeTARAAPEI-5, Unigene40\_TAEpaeTARAAPEI-5, Unigene41039\_TAEpaeTARAAPEI-5, Unigene41112\_TAEpaeTARAAPEI-5, Unigene41116\_TAEpaeTARAAPEI-5, Unigene41196\_TAEpaeTARAAPEI-5, Unigene41214\_TAEpaeTARAAPEI-5, Unigene41219\_TAEpaeTARAAPEI-5, Unigene41266\_TAEpaeTARAAPEI-5, Unigene41274\_TAEpaeTARAAPEI-5, Unigene41281\_TAEpaeTARAAPEI-5, Unigene4131\_TAEpaeTARAAPEI-5, Unigene41359\_TAEpaeTARAAPEI-5, Unigene41368\_TAEpaeTARAAPEI-5, Unigene41375\_TAEpaeTARAAPEI-5, Unigene41387\_TAEpaeTARAAPEI-5, Unigene41388\_TAEpaeTARAAPEI-5, Unigene41391\_TAEpaeTARAAPEI-5, Unigene41399\_TAEpaeTARAAPEI-5, Unigene41420\_TAEpaeTARAAPEI-5, Unigene41436\_TAEpaeTARAAPEI-5, Unigene41477\_TAEpaeTARAAPEI-5, Unigene41488\_TAEpaeTARAAPEI-5, Unigene41552\_TAEpaeTARAAPEI-5, Unigene41567\_TAEpaeTARAAPEI-5, Unigene41592\_TAEpaeTARAAPEI-5, Unigene41703\_TAEpaeTARAAPEI-5, Unigene41712\_TAEpaeTARAAPEI-5, Unigene41736\_TAEpaeTARAAPEI-5, Unigene4173\_TAEpaeTARAAPEI-5, Unigene41811\_TAEpaeTARAAPEI-5, Unigene41835\_TAEpaeTARAAPEI-5, Unigene41884\_TAEpaeTARAAPEI-5, Unigene41909\_TAEpaeTARAAPEI-5, Unigene41981\_TAEpaeTARAAPEI-5, Unigene42040\_TAEpaeTARAAPEI-5, Unigene42191\_TAEpaeTARAAPEI-5, Unigene42194\_TAEpaeTARAAPEI-5, Unigene42203\_TAEpaeTARAAPEI-5, Unigene42211\_TAEpaeTARAAPEI-5, Unigene42261\_TAEpaeTARAAPEI-5, Unigene42315\_TAEpaeTARAAPEI-5, Unigene42316\_TAEpaeTARAAPEI-5, Unigene4232\_TAEpaeTARAAPEI-5, Unigene42350\_TAEpaeTARAAPEI-5, Unigene42359\_TAEpaeTARAAPEI-5, Unigene42360\_TAEpaeTARAAPEI-5, Unigene42391\_TAEpaeTARAAPEI-5, Unigene42440\_TAEpaeTARAAPEI-5, Unigene42456\_TAEpaeTARAAPEI-5, Unigene42487\_TAEpaeTARAAPEI-5, Unigene42517\_TAEpaeTARAAPEI-5, Unigene42533\_TAEpaeTARAAPEI-5, Unigene42595\_TAEpaeTARAAPEI-5, Unigene42645\_TAEpaeTARAAPEI-5, Unigene42702\_TAEpaeTARAAPEI-5, Unigene42705\_TAEpaeTARAAPEI-5, Unigene42750\_TAEpaeTARAAPEI-5, Unigene4275\_TAEpaeTARAAPEI-5, Unigene42773\_TAEpaeTARAAPEI-5, Unigene42776\_TAEpaeTARAAPEI-5, Unigene42799\_TAEpaeTARAAPEI-5, Unigene42804\_TAEpaeTARAAPEI-5, Unigene42815\_TAEpaeTARAAPEI-5, Unigene42849\_TAEpaeTARAAPEI-5, Unigene42868\_TAEpaeTARAAPEI-5, Unigene42875\_TAEpaeTARAAPEI-5, Unigene42891\_TAEpaeTARAAPEI-5, Unigene42952\_TAEpaeTARAAPEI-5, Unigene42998\_TAEpaeTARAAPEI-5, Unigene43074\_TAEpaeTARAAPEI-5, Unigene43099\_TAEpaeTARAAPEI-5, Unigene43101\_TAEpaeTARAAPEI-5, Unigene43117\_TAEpaeTARAAPEI-5, Unigene43139\_TAEpaeTARAAPEI-5, Unigene43193\_TAEpaeTARAAPEI-5, Unigene43210\_TAEpaeTARAAPEI-5, Unigene4331\_TAEpaeTARAAPEI-5, Unigene43347\_TAEpaeTARAAPEI-5, Unigene43470\_TAEpaeTARAAPEI-5, Unigene43601\_TAEpaeTARAAPEI-5, Unigene43690\_TAEpaeTARAAPEI-5, Unigene43717\_TAEpaeTARAAPEI-5, Unigene43764\_TAEpaeTARAAPEI-5, Unigene43773\_TAEpaeTARAAPEI-5, Unigene43812\_TAEpaeTARAAPEI-5, Unigene43861\_TAEpaeTARAAPEI-5, Unigene43888\_TAEpaeTARAAPEI-5, Unigene43951\_TAEpaeTARAAPEI-5, Unigene43992\_TAEpaeTARAAPEI-5, Unigene44074\_TAEpaeTARAAPEI-5, Unigene44095\_TAEpaeTARAAPEI-5, Unigene44099\_TAEpaeTARAAPEI-5, Unigene44136\_TAEpaeTARAAPEI-5, Unigene44158\_TAEpaeTARAAPEI-5, Unigene4418\_TAEpaeTARAAPEI-5, Unigene44195\_TAEpaeTARAAPEI-5, Unigene44255\_TAEpaeTARAAPEI-5, Unigene44274\_TAEpaeTARAAPEI-5, Unigene44296\_TAEpaeTARAAPEI-5, Unigene44370\_TAEpaeTARAAPEI-5, Unigene44382\_TAEpaeTARAAPEI-5, Unigene44393\_TAEpaeTARAAPEI-5, Unigene44447\_TAEpaeTARAAPEI-5, Unigene44455\_TAEpaeTARAAPEI-5, Unigene44467\_TAEpaeTARAAPEI-5, Unigene44469\_TAEpaeTARAAPEI-5, Unigene44483\_TAEpaeTARAAPEI-5, Unigene44485\_TAEpaeTARAAPEI-5, Unigene44522\_TAEpaeTARAAPEI-5, Unigene44528\_TAEpaeTARAAPEI-5, Unigene44543\_TAEpaeTARAAPEI-5, Unigene44544\_TAEpaeTARAAPEI-5, Unigene44554\_TAEpaeTARAAPEI-5, Unigene44573\_TAEpaeTARAAPEI-5, Unigene44628\_TAEpaeTARAAPEI-5, Unigene44640\_TAEpaeTARAAPEI-5, Unigene44645\_TAEpaeTARAAPEI-5, Unigene44670\_TAEpaeTARAAPEI-5, Unigene44688\_TAEpaeTARAAPEI-5, Unigene4469\_TAEpaeTARAAPEI-5, Unigene4470\_TAEpaeTARAAPEI-5, Unigene4472\_TAEpaeTARAAPEI-5, Unigene44777\_TAEpaeTARAAPEI-5, Unigene44798\_TAEpaeTARAAPEI-5, Unigene44805\_TAEpaeTARAAPEI-5, Unigene44807\_TAEpaeTARAAPEI-5, Unigene44873\_TAEpaeTARAAPEI-5, Unigene44905\_TAEpaeTARAAPEI-5, Unigene44926\_TAEpaeTARAAPEI-5, Unigene44928\_TAEpaeTARAAPEI-5, Unigene44940\_TAEpaeTARAAPEI-5, Unigene44950\_TAEpaeTARAAPEI-5, Unigene44958\_TAEpaeTARAAPEI-5, Unigene44\_TAEpaeTARAAPEI-5, Unigene45041\_TAEpaeTARAAPEI-5, Unigene45096\_TAEpaeTARAAPEI-5, Unigene45119\_TAEpaeTARAAPEI-5, Unigene45168\_TAEpaeTARAAPEI-5, Unigene4519\_TAEpaeTARAAPEI-5, Unigene45235\_TAEpaeTARAAPEI-5, Unigene45250\_TAEpaeTARAAPEI-5, Unigene4525\_TAEpaeTARAAPEI-5, Unigene45348\_TAEpaeTARAAPEI-5, Unigene45351\_TAEpaeTARAAPEI-5, Unigene45393\_TAEpaeTARAAPEI-5, Unigene45421\_TAEpaeTARAAPEI-5, Unigene45439\_TAEpaeTARAAPEI-5, Unigene45444\_TAEpaeTARAAPEI-5, Unigene45484\_TAEpaeTARAAPEI-5, Unigene45487\_TAEpaeTARAAPEI-5, Unigene45489\_TAEpaeTARAAPEI-5, Unigene45493\_TAEpaeTARAAPEI-5, Unigene45494\_TAEpaeTARAAPEI-5, Unigene45538\_TAEpaeTARAAPEI-5, Unigene45565\_TAEpaeTARAAPEI-5, Unigene45569\_TAEpaeTARAAPEI-5, Unigene45577\_TAEpaeTARAAPEI-5, Unigene45599\_TAEpaeTARAAPEI-5, Unigene45636\_TAEpaeTARAAPEI-5, Unigene45669\_TAEpaeTARAAPEI-5, Unigene45676\_TAEpaeTARAAPEI-5, Unigene45769\_TAEpaeTARAAPEI-5, Unigene45775\_TAEpaeTARAAPEI-5, Unigene45785\_TAEpaeTARAAPEI-5, Unigene45796\_TAEpaeTARAAPEI-5, Unigene45867\_TAEpaeTARAAPEI-5, Unigene4587\_TAEpaeTARAAPEI-5, Unigene45899\_TAEpaeTARAAPEI-5, Unigene45901\_TAEpaeTARAAPEI-5, Unigene45957\_TAEpaeTARAAPEI-5, Unigene4595\_TAEpaeTARAAPEI-5, Unigene45979\_TAEpaeTARAAPEI-5, Unigene46031\_TAEpaeTARAAPEI-5, Unigene46060\_TAEpaeTARAAPEI-5, Unigene46148\_TAEpaeTARAAPEI-5, Unigene46202\_TAEpaeTARAAPEI-5, Unigene46209\_TAEpaeTARAAPEI-5, Unigene46218\_TAEpaeTARAAPEI-5, Unigene4621\_TAEpaeTARAAPEI-5, Unigene46246\_TAEpaeTARAAPEI-5, Unigene4625\_TAEpaeTARAAPEI-5, Unigene46268\_TAEpaeTARAAPEI-5, Unigene4626\_TAEpaeTARAAPEI-5, Unigene46297\_TAEpaeTARAAPEI-5, Unigene46301\_TAEpaeTARAAPEI-5, Unigene46330\_TAEpaeTARAAPEI-5, Unigene46365\_TAEpaeTARAAPEI-5, Unigene46377\_TAEpaeTARAAPEI-5, Unigene46409\_TAEpaeTARAAPEI-5, Unigene46416\_TAEpaeTARAAPEI-5, Unigene46444\_TAEpaeTARAAPEI-5, Unigene46470\_TAEpaeTARAAPEI-5, Unigene46471\_TAEpaeTARAAPEI-5, Unigene46483\_TAEpaeTARAAPEI-5, Unigene46567\_TAEpaeTARAAPEI-5, Unigene46568\_TAEpaeTARAAPEI-5, Unigene46581\_TAEpaeTARAAPEI-5, Unigene46587\_TAEpaeTARAAPEI-5, Unigene46602\_TAEpaeTARAAPEI-5, Unigene46603\_TAEpaeTARAAPEI-5, Unigene46606\_TAEpaeTARAAPEI-5, Unigene46614\_TAEpaeTARAAPEI-5, Unigene46617\_TAEpaeTARAAPEI-5, Unigene46676\_TAEpaeTARAAPEI-5, Unigene46733\_TAEpaeTARAAPEI-5, Unigene46736\_TAEpaeTARAAPEI-5, Unigene46746\_TAEpaeTARAAPEI-5, Unigene4678\_TAEpaeTARAAPEI-5, Unigene46813\_TAEpaeTARAAPEI-5, Unigene46831\_TAEpaeTARAAPEI-5, Unigene46859\_TAEpaeTARAAPEI-5, Unigene4687\_TAEpaeTARAAPEI-5, Unigene46934\_TAEpaeTARAAPEI-5, Unigene46939\_TAEpaeTARAAPEI-5, Unigene47001\_TAEpaeTARAAPEI-5, Unigene47019\_TAEpaeTARAAPEI-5, Unigene47049\_TAEpaeTARAAPEI-5, Unigene47109\_TAEpaeTARAAPEI-5, Unigene47195\_TAEpaeTARAAPEI-5, Unigene47308\_TAEpaeTARAAPEI-5, Unigene4736\_TAEpaeTARAAPEI-5, Unigene47372\_TAEpaeTARAAPEI-5, Unigene47411\_TAEpaeTARAAPEI-5, Unigene4741\_TAEpaeTARAAPEI-5, Unigene47446\_TAEpaeTARAAPEI-5, Unigene47454\_TAEpaeTARAAPEI-5, Unigene47459\_TAEpaeTARAAPEI-5, Unigene47501\_TAEpaeTARAAPEI-5, Unigene47578\_TAEpaeTARAAPEI-5, Unigene4758\_TAEpaeTARAAPEI-5, Unigene47608\_TAEpaeTARAAPEI-5, Unigene4761\_TAEpaeTARAAPEI-5, Unigene47647\_TAEpaeTARAAPEI-5, Unigene47683\_TAEpaeTARAAPEI-5, Unigene47700\_TAEpaeTARAAPEI-5, Unigene47705\_TAEpaeTARAAPEI-5, Unigene47713\_TAEpaeTARAAPEI-5, Unigene47847\_TAEpaeTARAAPEI-5, Unigene4791\_TAEpaeTARAAPEI-5, Unigene47935\_TAEpaeTARAAPEI-5, Unigene47946\_TAEpaeTARAAPEI-5, Unigene47977\_TAEpaeTARAAPEI-5, Unigene47982\_TAEpaeTARAAPEI-5, Unigene48082\_TAEpaeTARAAPEI-5, Unigene48176\_TAEpaeTARAAPEI-5, Unigene48237\_TAEpaeTARAAPEI-5, Unigene48269\_TAEpaeTARAAPEI-5, Unigene48274\_TAEpaeTARAAPEI-5, Unigene48277\_TAEpaeTARAAPEI-5, Unigene48305\_TAEpaeTARAAPEI-5, Unigene48344\_TAEpaeTARAAPEI-5, Unigene48355\_TAEpaeTARAAPEI-5, Unigene48366\_TAEpaeTARAAPEI-5, Unigene48447\_TAEpaeTARAAPEI-5, Unigene48448\_TAEpaeTARAAPEI-5, Unigene4845\_TAEpaeTARAAPEI-5, Unigene48496\_TAEpaeTARAAPEI-5, Unigene48502\_TAEpaeTARAAPEI-5, Unigene4855\_TAEpaeTARAAPEI-5, Unigene48691\_TAEpaeTARAAPEI-5, Unigene48696\_TAEpaeTARAAPEI-5, Unigene48708\_TAEpaeTARAAPEI-5, Unigene48734\_TAEpaeTARAAPEI-5, Unigene48735\_TAEpaeTARAAPEI-5, Unigene48744\_TAEpaeTARAAPEI-5, Unigene48829\_TAEpaeTARAAPEI-5, Unigene48878\_TAEpaeTARAAPEI-5, Unigene4890\_TAEpaeTARAAPEI-5, Unigene48910\_TAEpaeTARAAPEI-5, Unigene4891\_TAEpaeTARAAPEI-5, Unigene48926\_TAEpaeTARAAPEI-5, Unigene48970\_TAEpaeTARAAPEI-5, Unigene48973\_TAEpaeTARAAPEI-5, Unigene48993\_TAEpaeTARAAPEI-5, Unigene49025\_TAEpaeTARAAPEI-5, Unigene49038\_TAEpaeTARAAPEI-5, Unigene49045\_TAEpaeTARAAPEI-5, Unigene49101\_TAEpaeTARAAPEI-5, Unigene49108\_TAEpaeTARAAPEI-5, Unigene49141\_TAEpaeTARAAPEI-5, Unigene49149\_TAEpaeTARAAPEI-5, Unigene49203\_TAEpaeTARAAPEI-5, Unigene49233\_TAEpaeTARAAPEI-5, Unigene49292\_TAEpaeTARAAPEI-5, Unigene49302\_TAEpaeTARAAPEI-5, Unigene49307\_TAEpaeTARAAPEI-5, Unigene49315\_TAEpaeTARAAPEI-5, Unigene49327\_TAEpaeTARAAPEI-5, Unigene4933\_TAEpaeTARAAPEI-5, Unigene49351\_TAEpaeTARAAPEI-5, Unigene49369\_TAEpaeTARAAPEI-5, Unigene49425\_TAEpaeTARAAPEI-5, Unigene49439\_TAEpaeTARAAPEI-5, Unigene49456\_TAEpaeTARAAPEI-5, Unigene4945\_TAEpaeTARAAPEI-5, Unigene49481\_TAEpaeTARAAPEI-5, Unigene49526\_TAEpaeTARAAPEI-5, Unigene49530\_TAEpaeTARAAPEI-5, Unigene49533\_TAEpaeTARAAPEI-5, Unigene49540\_TAEpaeTARAAPEI-5, Unigene49574\_TAEpaeTARAAPEI-5, Unigene49613\_TAEpaeTARAAPEI-5, Unigene49620\_TAEpaeTARAAPEI-5, Unigene4969\_TAEpaeTARAAPEI-5, Unigene49703\_TAEpaeTARAAPEI-5, Unigene4974\_TAEpaeTARAAPEI-5, Unigene49768\_TAEpaeTARAAPEI-5, Unigene49799\_TAEpaeTARAAPEI-5, Unigene49933\_TAEpaeTARAAPEI-5, Unigene50000\_TAEpaeTARAAPEI-5, Unigene50069\_TAEpaeTARAAPEI-5, Unigene50088\_TAEpaeTARAAPEI-5, Unigene50104\_TAEpaeTARAAPEI-5, Unigene50131\_TAEpaeTARAAPEI-5, Unigene50145\_TAEpaeTARAAPEI-5, Unigene50169\_TAEpaeTARAAPEI-5, Unigene50173\_TAEpaeTARAAPEI-5, Unigene50209\_TAEpaeTARAAPEI-5, Unigene50304\_TAEpaeTARAAPEI-5, Unigene50351\_TAEpaeTARAAPEI-5, Unigene50382\_TAEpaeTARAAPEI-5, Unigene50386\_TAEpaeTARAAPEI-5, Unigene50410\_TAEpaeTARAAPEI-5, Unigene5043\_TAEpaeTARAAPEI-5, Unigene50453\_TAEpaeTARAAPEI-5, Unigene50466\_TAEpaeTARAAPEI-5, Unigene50543\_TAEpaeTARAAPEI-5, Unigene50548\_TAEpaeTARAAPEI-5, Unigene50597\_TAEpaeTARAAPEI-5, Unigene50604\_TAEpaeTARAAPEI-5, Unigene50641\_TAEpaeTARAAPEI-5, Unigene50648\_TAEpaeTARAAPEI-5, Unigene5066\_TAEpaeTARAAPEI-5, Unigene50673\_TAEpaeTARAAPEI-5, Unigene50674\_TAEpaeTARAAPEI-5, Unigene50740\_TAEpaeTARAAPEI-5, Unigene50742\_TAEpaeTARAAPEI-5, Unigene50753\_TAEpaeTARAAPEI-5, Unigene50801\_TAEpaeTARAAPEI-5, Unigene50809\_TAEpaeTARAAPEI-5, Unigene50810\_TAEpaeTARAAPEI-5, Unigene50829\_TAEpaeTARAAPEI-5, Unigene50850\_TAEpaeTARAAPEI-5, Unigene50899\_TAEpaeTARAAPEI-5, Unigene50907\_TAEpaeTARAAPEI-5, Unigene50922\_TAEpaeTARAAPEI-5, Unigene5092\_TAEpaeTARAAPEI-5, Unigene50942\_TAEpaeTARAAPEI-5, Unigene50979\_TAEpaeTARAAPEI-5, Unigene51029\_TAEpaeTARAAPEI-5, Unigene51133\_TAEpaeTARAAPEI-5, Unigene51134\_TAEpaeTARAAPEI-5, Unigene51139\_TAEpaeTARAAPEI-5, Unigene51171\_TAEpaeTARAAPEI-5, Unigene51223\_TAEpaeTARAAPEI-5, Unigene51248\_TAEpaeTARAAPEI-5, Unigene51262\_TAEpaeTARAAPEI-5, Unigene51274\_TAEpaeTARAAPEI-5, Unigene51283\_TAEpaeTARAAPEI-5, Unigene51308\_TAEpaeTARAAPEI-5, Unigene51324\_TAEpaeTARAAPEI-5, Unigene51352\_TAEpaeTARAAPEI-5, Unigene51368\_TAEpaeTARAAPEI-5, Unigene51378\_TAEpaeTARAAPEI-5, Unigene51392\_TAEpaeTARAAPEI-5, Unigene51465\_TAEpaeTARAAPEI-5, Unigene51513\_TAEpaeTARAAPEI-5, Unigene51523\_TAEpaeTARAAPEI-5, Unigene51561\_TAEpaeTARAAPEI-5, Unigene5158\_TAEpaeTARAAPEI-5, Unigene51619\_TAEpaeTARAAPEI-5, Unigene51637\_TAEpaeTARAAPEI-5, Unigene51644\_TAEpaeTARAAPEI-5, Unigene51645\_TAEpaeTARAAPEI-5, Unigene51684\_TAEpaeTARAAPEI-5, Unigene51697\_TAEpaeTARAAPEI-5, Unigene51708\_TAEpaeTARAAPEI-5, Unigene51735\_TAEpaeTARAAPEI-5, Unigene51772\_TAEpaeTARAAPEI-5, Unigene5182\_TAEpaeTARAAPEI-5, Unigene51843\_TAEpaeTARAAPEI-5, Unigene51847\_TAEpaeTARAAPEI-5, Unigene51880\_TAEpaeTARAAPEI-5, Unigene51883\_TAEpaeTARAAPEI-5, Unigene51890\_TAEpaeTARAAPEI-5, Unigene51902\_TAEpaeTARAAPEI-5, Unigene51955\_TAEpaeTARAAPEI-5, Unigene51977\_TAEpaeTARAAPEI-5, Unigene51984\_TAEpaeTARAAPEI-5, Unigene52012\_TAEpaeTARAAPEI-5, Unigene52020\_TAEpaeTARAAPEI-5, Unigene52059\_TAEpaeTARAAPEI-5, Unigene52078\_TAEpaeTARAAPEI-5, Unigene52110\_TAEpaeTARAAPEI-5, Unigene52168\_TAEpaeTARAAPEI-5, Unigene52224\_TAEpaeTARAAPEI-5, Unigene52243\_TAEpaeTARAAPEI-5, Unigene52252\_TAEpaeTARAAPEI-5, Unigene52264\_TAEpaeTARAAPEI-5, Unigene52278\_TAEpaeTARAAPEI-5, Unigene52356\_TAEpaeTARAAPEI-5, Unigene5240\_TAEpaeTARAAPEI-5, Unigene52429\_TAEpaeTARAAPEI-5, Unigene52432\_TAEpaeTARAAPEI-5, Unigene52446\_TAEpaeTARAAPEI-5, Unigene52490\_TAEpaeTARAAPEI-5, Unigene52540\_TAEpaeTARAAPEI-5, Unigene52548\_TAEpaeTARAAPEI-5, Unigene52566\_TAEpaeTARAAPEI-5, Unigene52602\_TAEpaeTARAAPEI-5, Unigene52609\_TAEpaeTARAAPEI-5, Unigene52611\_TAEpaeTARAAPEI-5, Unigene52665\_TAEpaeTARAAPEI-5, Unigene52684\_TAEpaeTARAAPEI-5, Unigene52689\_TAEpaeTARAAPEI-5, Unigene52781\_TAEpaeTARAAPEI-5, Unigene52797\_TAEpaeTARAAPEI-5, Unigene52799\_TAEpaeTARAAPEI-5, Unigene5279\_TAEpaeTARAAPEI-5, Unigene52815\_TAEpaeTARAAPEI-5, Unigene52825\_TAEpaeTARAAPEI-5, Unigene5284\_TAEpaeTARAAPEI-5, Unigene52891\_TAEpaeTARAAPEI-5, Unigene52903\_TAEpaeTARAAPEI-5, Unigene52921\_TAEpaeTARAAPEI-5, Unigene52927\_TAEpaeTARAAPEI-5, Unigene52953\_TAEpaeTARAAPEI-5, Unigene52958\_TAEpaeTARAAPEI-5, Unigene53052\_TAEpaeTARAAPEI-5, Unigene53081\_TAEpaeTARAAPEI-5, Unigene53085\_TAEpaeTARAAPEI-5, Unigene53161\_TAEpaeTARAAPEI-5, Unigene53182\_TAEpaeTARAAPEI-5, Unigene53197\_TAEpaeTARAAPEI-5, Unigene53213\_TAEpaeTARAAPEI-5, Unigene53243\_TAEpaeTARAAPEI-5, Unigene53273\_TAEpaeTARAAPEI-5, Unigene53304\_TAEpaeTARAAPEI-5, Unigene53355\_TAEpaeTARAAPEI-5, Unigene53388\_TAEpaeTARAAPEI-5, Unigene533\_TAEpaeTARAAPEI-5, Unigene53467\_TAEpaeTARAAPEI-5, Unigene53472\_TAEpaeTARAAPEI-5, Unigene53512\_TAEpaeTARAAPEI-5, Unigene53557\_TAEpaeTARAAPEI-5, Unigene53580\_TAEpaeTARAAPEI-5, Unigene53584\_TAEpaeTARAAPEI-5, Unigene53604\_TAEpaeTARAAPEI-5, Unigene53644\_TAEpaeTARAAPEI-5, Unigene53725\_TAEpaeTARAAPEI-5, Unigene53758\_TAEpaeTARAAPEI-5, Unigene53766\_TAEpaeTARAAPEI-5, Unigene53825\_TAEpaeTARAAPEI-5, Unigene53837\_TAEpaeTARAAPEI-5, Unigene53842\_TAEpaeTARAAPEI-5, Unigene53844\_TAEpaeTARAAPEI-5, Unigene53883\_TAEpaeTARAAPEI-5, Unigene5388\_TAEpaeTARAAPEI-5, Unigene53947\_TAEpaeTARAAPEI-5, Unigene53966\_TAEpaeTARAAPEI-5, Unigene5398\_TAEpaeTARAAPEI-5, Unigene54034\_TAEpaeTARAAPEI-5, Unigene54035\_TAEpaeTARAAPEI-5, Unigene54065\_TAEpaeTARAAPEI-5, Unigene54137\_TAEpaeTARAAPEI-5, Unigene54138\_TAEpaeTARAAPEI-5, Unigene54153\_TAEpaeTARAAPEI-5, Unigene54214\_TAEpaeTARAAPEI-5, Unigene54274\_TAEpaeTARAAPEI-5, Unigene54296\_TAEpaeTARAAPEI-5, Unigene54298\_TAEpaeTARAAPEI-5, Unigene542\_TAEpaeTARAAPEI-5, Unigene54366\_TAEpaeTARAAPEI-5, Unigene54427\_TAEpaeTARAAPEI-5, Unigene54434\_TAEpaeTARAAPEI-5, Unigene54472\_TAEpaeTARAAPEI-5, Unigene54534\_TAEpaeTARAAPEI-5, Unigene54593\_TAEpaeTARAAPEI-5, Unigene5464\_TAEpaeTARAAPEI-5, Unigene54662\_TAEpaeTARAAPEI-5, Unigene54669\_TAEpaeTARAAPEI-5, Unigene54685\_TAEpaeTARAAPEI-5, Unigene54699\_TAEpaeTARAAPEI-5, Unigene54725\_TAEpaeTARAAPEI-5, Unigene54766\_TAEpaeTARAAPEI-5, Unigene54767\_TAEpaeTARAAPEI-5, Unigene54802\_TAEpaeTARAAPEI-5, Unigene54820\_TAEpaeTARAAPEI-5, Unigene54850\_TAEpaeTARAAPEI-5, Unigene54914\_TAEpaeTARAAPEI-5, Unigene54929\_TAEpaeTARAAPEI-5, Unigene54934\_TAEpaeTARAAPEI-5, Unigene54973\_TAEpaeTARAAPEI-5, Unigene55000\_TAEpaeTARAAPEI-5, Unigene55014\_TAEpaeTARAAPEI-5, Unigene55037\_TAEpaeTARAAPEI-5, Unigene55040\_TAEpaeTARAAPEI-5, Unigene55060\_TAEpaeTARAAPEI-5, Unigene55101\_TAEpaeTARAAPEI-5, Unigene55103\_TAEpaeTARAAPEI-5, Unigene55117\_TAEpaeTARAAPEI-5, Unigene55161\_TAEpaeTARAAPEI-5, Unigene55194\_TAEpaeTARAAPEI-5, Unigene55239\_TAEpaeTARAAPEI-5, Unigene55265\_TAEpaeTARAAPEI-5, Unigene55268\_TAEpaeTARAAPEI-5, Unigene55363\_TAEpaeTARAAPEI-5, Unigene55369\_TAEpaeTARAAPEI-5, Unigene55378\_TAEpaeTARAAPEI-5, Unigene55389\_TAEpaeTARAAPEI-5, Unigene55419\_TAEpaeTARAAPEI-5, Unigene5542\_TAEpaeTARAAPEI-5, Unigene55437\_TAEpaeTARAAPEI-5, Unigene55443\_TAEpaeTARAAPEI-5, Unigene55478\_TAEpaeTARAAPEI-5, Unigene55487\_TAEpaeTARAAPEI-5, Unigene55492\_TAEpaeTARAAPEI-5, Unigene55495\_TAEpaeTARAAPEI-5, Unigene55550\_TAEpaeTARAAPEI-5, Unigene55554\_TAEpaeTARAAPEI-5, Unigene55557\_TAEpaeTARAAPEI-5, Unigene55649\_TAEpaeTARAAPEI-5, Unigene55656\_TAEpaeTARAAPEI-5, Unigene55661\_TAEpaeTARAAPEI-5, Unigene55681\_TAEpaeTARAAPEI-5, Unigene55682\_TAEpaeTARAAPEI-5, Unigene55684\_TAEpaeTARAAPEI-5, Unigene55732\_TAEpaeTARAAPEI-5, Unigene55759\_TAEpaeTARAAPEI-5, Unigene55767\_TAEpaeTARAAPEI-5, Unigene55791\_TAEpaeTARAAPEI-5, Unigene55813\_TAEpaeTARAAPEI-5, Unigene5582\_TAEpaeTARAAPEI-5, Unigene55830\_TAEpaeTARAAPEI-5, Unigene55841\_TAEpaeTARAAPEI-5, Unigene55924\_TAEpaeTARAAPEI-5, Unigene55958\_TAEpaeTARAAPEI-5, Unigene55998\_TAEpaeTARAAPEI-5, Unigene559\_TAEpaeTARAAPEI-5, Unigene56003\_TAEpaeTARAAPEI-5, Unigene56014\_TAEpaeTARAAPEI-5, Unigene56039\_TAEpaeTARAAPEI-5, Unigene56046\_TAEpaeTARAAPEI-5, Unigene56058\_TAEpaeTARAAPEI-5, Unigene56061\_TAEpaeTARAAPEI-5, Unigene56146\_TAEpaeTARAAPEI-5, Unigene56164\_TAEpaeTARAAPEI-5, Unigene56173\_TAEpaeTARAAPEI-5, Unigene5620\_TAEpaeTARAAPEI-5, Unigene56240\_TAEpaeTARAAPEI-5, Unigene56249\_TAEpaeTARAAPEI-5, Unigene56250\_TAEpaeTARAAPEI-5, Unigene56255\_TAEpaeTARAAPEI-5, Unigene5626\_TAEpaeTARAAPEI-5, Unigene56285\_TAEpaeTARAAPEI-5, Unigene56340\_TAEpaeTARAAPEI-5, Unigene56342\_TAEpaeTARAAPEI-5, Unigene56427\_TAEpaeTARAAPEI-5, Unigene56450\_TAEpaeTARAAPEI-5, Unigene56512\_TAEpaeTARAAPEI-5, Unigene56523\_TAEpaeTARAAPEI-5, Unigene56555\_TAEpaeTARAAPEI-5, Unigene56556\_TAEpaeTARAAPEI-5, Unigene56572\_TAEpaeTARAAPEI-5, Unigene56585\_TAEpaeTARAAPEI-5, Unigene56642\_TAEpaeTARAAPEI-5, Unigene56659\_TAEpaeTARAAPEI-5, Unigene56670\_TAEpaeTARAAPEI-5, Unigene56679\_TAEpaeTARAAPEI-5, Unigene56709\_TAEpaeTARAAPEI-5, Unigene56730\_TAEpaeTARAAPEI-5, Unigene56739\_TAEpaeTARAAPEI-5, Unigene56801\_TAEpaeTARAAPEI-5, Unigene56810\_TAEpaeTARAAPEI-5, Unigene56869\_TAEpaeTARAAPEI-5, Unigene56912\_TAEpaeTARAAPEI-5, Unigene56913\_TAEpaeTARAAPEI-5, Unigene56929\_TAEpaeTARAAPEI-5, Unigene56934\_TAEpaeTARAAPEI-5, Unigene56955\_TAEpaeTARAAPEI-5, Unigene56982\_TAEpaeTARAAPEI-5, Unigene56986\_TAEpaeTARAAPEI-5, Unigene57043\_TAEpaeTARAAPEI-5, Unigene57083\_TAEpaeTARAAPEI-5, Unigene57099\_TAEpaeTARAAPEI-5, Unigene57123\_TAEpaeTARAAPEI-5, Unigene57157\_TAEpaeTARAAPEI-5, Unigene57177\_TAEpaeTARAAPEI-5, Unigene57181\_TAEpaeTARAAPEI-5, Unigene57219\_TAEpaeTARAAPEI-5, Unigene57224\_TAEpaeTARAAPEI-5, Unigene57231\_TAEpaeTARAAPEI-5, Unigene57272\_TAEpaeTARAAPEI-5, Unigene57338\_TAEpaeTARAAPEI-5, Unigene57364\_TAEpaeTARAAPEI-5, Unigene57367\_TAEpaeTARAAPEI-5, Unigene57373\_TAEpaeTARAAPEI-5, Unigene57472\_TAEpaeTARAAPEI-5, Unigene57497\_TAEpaeTARAAPEI-5, Unigene57499\_TAEpaeTARAAPEI-5, Unigene57510\_TAEpaeTARAAPEI-5, Unigene5757\_TAEpaeTARAAPEI-5, Unigene57587\_TAEpaeTARAAPEI-5, Unigene57599\_TAEpaeTARAAPEI-5, Unigene57601\_TAEpaeTARAAPEI-5, Unigene57621\_TAEpaeTARAAPEI-5, Unigene57623\_TAEpaeTARAAPEI-5, Unigene57627\_TAEpaeTARAAPEI-5, Unigene57630\_TAEpaeTARAAPEI-5, Unigene57645\_TAEpaeTARAAPEI-5, Unigene57653\_TAEpaeTARAAPEI-5, Unigene57693\_TAEpaeTARAAPEI-5, Unigene57696\_TAEpaeTARAAPEI-5, Unigene57718\_TAEpaeTARAAPEI-5, Unigene57736\_TAEpaeTARAAPEI-5, Unigene57831\_TAEpaeTARAAPEI-5, Unigene57839\_TAEpaeTARAAPEI-5, Unigene57891\_TAEpaeTARAAPEI-5, Unigene57902\_TAEpaeTARAAPEI-5, Unigene57931\_TAEpaeTARAAPEI-5, Unigene57970\_TAEpaeTARAAPEI-5, Unigene58035\_TAEpaeTARAAPEI-5, Unigene58055\_TAEpaeTARAAPEI-5, Unigene58058\_TAEpaeTARAAPEI-5, Unigene58086\_TAEpaeTARAAPEI-5, Unigene58113\_TAEpaeTARAAPEI-5, Unigene58125\_TAEpaeTARAAPEI-5, Unigene58133\_TAEpaeTARAAPEI-5, Unigene58150\_TAEpaeTARAAPEI-5, Unigene58213\_TAEpaeTARAAPEI-5, Unigene58218\_TAEpaeTARAAPEI-5, Unigene58223\_TAEpaeTARAAPEI-5, Unigene58250\_TAEpaeTARAAPEI-5, Unigene58309\_TAEpaeTARAAPEI-5, Unigene58315\_TAEpaeTARAAPEI-5, Unigene58360\_TAEpaeTARAAPEI-5, Unigene58386\_TAEpaeTARAAPEI-5, Unigene58411\_TAEpaeTARAAPEI-5, Unigene58444\_TAEpaeTARAAPEI-5, Unigene58481\_TAEpaeTARAAPEI-5, Unigene58508\_TAEpaeTARAAPEI-5, Unigene58525\_TAEpaeTARAAPEI-5, Unigene58586\_TAEpaeTARAAPEI-5, Unigene58625\_TAEpaeTARAAPEI-5, Unigene58659\_TAEpaeTARAAPEI-5, Unigene58660\_TAEpaeTARAAPEI-5, Unigene58713\_TAEpaeTARAAPEI-5, Unigene58716\_TAEpaeTARAAPEI-5, Unigene58763\_TAEpaeTARAAPEI-5, Unigene58774\_TAEpaeTARAAPEI-5, Unigene58791\_TAEpaeTARAAPEI-5, Unigene58794\_TAEpaeTARAAPEI-5, Unigene5879\_TAEpaeTARAAPEI-5, Unigene58800\_TAEpaeTARAAPEI-5, Unigene58813\_TAEpaeTARAAPEI-5, Unigene58817\_TAEpaeTARAAPEI-5, Unigene58850\_TAEpaeTARAAPEI-5, Unigene58877\_TAEpaeTARAAPEI-5, Unigene58909\_TAEpaeTARAAPEI-5, Unigene58930\_TAEpaeTARAAPEI-5, Unigene58935\_TAEpaeTARAAPEI-5, Unigene58941\_TAEpaeTARAAPEI-5, Unigene58951\_TAEpaeTARAAPEI-5, Unigene58958\_TAEpaeTARAAPEI-5, Unigene59003\_TAEpaeTARAAPEI-5, Unigene59015\_TAEpaeTARAAPEI-5, Unigene59053\_TAEpaeTARAAPEI-5, Unigene59070\_TAEpaeTARAAPEI-5, Unigene59075\_TAEpaeTARAAPEI-5, Unigene59131\_TAEpaeTARAAPEI-5, Unigene59158\_TAEpaeTARAAPEI-5, Unigene59179\_TAEpaeTARAAPEI-5, Unigene59181\_TAEpaeTARAAPEI-5, Unigene59205\_TAEpaeTARAAPEI-5, Unigene59209\_TAEpaeTARAAPEI-5, Unigene59218\_TAEpaeTARAAPEI-5, Unigene59226\_TAEpaeTARAAPEI-5, Unigene59297\_TAEpaeTARAAPEI-5, Unigene59326\_TAEpaeTARAAPEI-5, Unigene59346\_TAEpaeTARAAPEI-5, Unigene59360\_TAEpaeTARAAPEI-5, Unigene59376\_TAEpaeTARAAPEI-5, Unigene59411\_TAEpaeTARAAPEI-5, Unigene59412\_TAEpaeTARAAPEI-5, Unigene59427\_TAEpaeTARAAPEI-5, Unigene59429\_TAEpaeTARAAPEI-5, Unigene59453\_TAEpaeTARAAPEI-5, Unigene59464\_TAEpaeTARAAPEI-5, Unigene59466\_TAEpaeTARAAPEI-5, Unigene59570\_TAEpaeTARAAPEI-5, Unigene59577\_TAEpaeTARAAPEI-5, Unigene59602\_TAEpaeTARAAPEI-5, Unigene59606\_TAEpaeTARAAPEI-5, Unigene59611\_TAEpaeTARAAPEI-5, Unigene59615\_TAEpaeTARAAPEI-5, Unigene59629\_TAEpaeTARAAPEI-5, Unigene59638\_TAEpaeTARAAPEI-5, Unigene59647\_TAEpaeTARAAPEI-5, Unigene59648\_TAEpaeTARAAPEI-5, Unigene59654\_TAEpaeTARAAPEI-5, Unigene59686\_TAEpaeTARAAPEI-5, Unigene59694\_TAEpaeTARAAPEI-5, Unigene59709\_TAEpaeTARAAPEI-5, Unigene59731\_TAEpaeTARAAPEI-5, Unigene59752\_TAEpaeTARAAPEI-5, Unigene59754\_TAEpaeTARAAPEI-5, Unigene59782\_TAEpaeTARAAPEI-5, Unigene59793\_TAEpaeTARAAPEI-5, Unigene59811\_TAEpaeTARAAPEI-5, Unigene5981\_TAEpaeTARAAPEI-5, Unigene59828\_TAEpaeTARAAPEI-5, Unigene59839\_TAEpaeTARAAPEI-5, Unigene59852\_TAEpaeTARAAPEI-5, Unigene59861\_TAEpaeTARAAPEI-5, Unigene59909\_TAEpaeTARAAPEI-5, Unigene59916\_TAEpaeTARAAPEI-5, Unigene59960\_TAEpaeTARAAPEI-5, Unigene59995\_TAEpaeTARAAPEI-5, Unigene59996\_TAEpaeTARAAPEI-5, Unigene59998\_TAEpaeTARAAPEI-5, Unigene60035\_TAEpaeTARAAPEI-5, Unigene60049\_TAEpaeTARAAPEI-5, Unigene60059\_TAEpaeTARAAPEI-5, Unigene60071\_TAEpaeTARAAPEI-5, Unigene60082\_TAEpaeTARAAPEI-5, Unigene60149\_TAEpaeTARAAPEI-5, Unigene60187\_TAEpaeTARAAPEI-5, Unigene60212\_TAEpaeTARAAPEI-5, Unigene60218\_TAEpaeTARAAPEI-5, Unigene60220\_TAEpaeTARAAPEI-5, Unigene60325\_TAEpaeTARAAPEI-5, Unigene60350\_TAEpaeTARAAPEI-5, Unigene60408\_TAEpaeTARAAPEI-5, Unigene60410\_TAEpaeTARAAPEI-5, Unigene60414\_TAEpaeTARAAPEI-5, Unigene60422\_TAEpaeTARAAPEI-5, Unigene60468\_TAEpaeTARAAPEI-5, Unigene60472\_TAEpaeTARAAPEI-5, Unigene60491\_TAEpaeTARAAPEI-5, Unigene60547\_TAEpaeTARAAPEI-5, Unigene60551\_TAEpaeTARAAPEI-5, Unigene60564\_TAEpaeTARAAPEI-5, Unigene60568\_TAEpaeTARAAPEI-5, Unigene60579\_TAEpaeTARAAPEI-5, Unigene60612\_TAEpaeTARAAPEI-5, Unigene60637\_TAEpaeTARAAPEI-5, Unigene60651\_TAEpaeTARAAPEI-5, Unigene60658\_TAEpaeTARAAPEI-5, Unigene60695\_TAEpaeTARAAPEI-5, Unigene60755\_TAEpaeTARAAPEI-5, Unigene60794\_TAEpaeTARAAPEI-5, Unigene60814\_TAEpaeTARAAPEI-5, Unigene60817\_TAEpaeTARAAPEI-5, Unigene60832\_TAEpaeTARAAPEI-5, Unigene60852\_TAEpaeTARAAPEI-5, Unigene60875\_TAEpaeTARAAPEI-5, Unigene60891\_TAEpaeTARAAPEI-5, Unigene60925\_TAEpaeTARAAPEI-5, Unigene60930\_TAEpaeTARAAPEI-5, Unigene60946\_TAEpaeTARAAPEI-5, Unigene60955\_TAEpaeTARAAPEI-5, Unigene60998\_TAEpaeTARAAPEI-5, Unigene60\_TAEpaeTARAAPEI-5, Unigene61043\_TAEpaeTARAAPEI-5, Unigene61058\_TAEpaeTARAAPEI-5, Unigene61077\_TAEpaeTARAAPEI-5, Unigene61092\_TAEpaeTARAAPEI-5, Unigene61104\_TAEpaeTARAAPEI-5, Unigene61118\_TAEpaeTARAAPEI-5, Unigene61123\_TAEpaeTARAAPEI-5, Unigene61126\_TAEpaeTARAAPEI-5, Unigene61179\_TAEpaeTARAAPEI-5, Unigene61184\_TAEpaeTARAAPEI-5, Unigene61230\_TAEpaeTARAAPEI-5, Unigene61268\_TAEpaeTARAAPEI-5, Unigene61269\_TAEpaeTARAAPEI-5, Unigene61270\_TAEpaeTARAAPEI-5, Unigene61350\_TAEpaeTARAAPEI-5, Unigene61387\_TAEpaeTARAAPEI-5, Unigene61390\_TAEpaeTARAAPEI-5, Unigene61404\_TAEpaeTARAAPEI-5, Unigene61408\_TAEpaeTARAAPEI-5, Unigene61479\_TAEpaeTARAAPEI-5, Unigene61510\_TAEpaeTARAAPEI-5, Unigene6152\_TAEpaeTARAAPEI-5, Unigene61532\_TAEpaeTARAAPEI-5, Unigene61564\_TAEpaeTARAAPEI-5, Unigene6157\_TAEpaeTARAAPEI-5, Unigene61583\_TAEpaeTARAAPEI-5, Unigene61588\_TAEpaeTARAAPEI-5, Unigene61608\_TAEpaeTARAAPEI-5, Unigene61621\_TAEpaeTARAAPEI-5, Unigene61649\_TAEpaeTARAAPEI-5, Unigene61674\_TAEpaeTARAAPEI-5, Unigene61676\_TAEpaeTARAAPEI-5, Unigene61679\_TAEpaeTARAAPEI-5, Unigene61680\_TAEpaeTARAAPEI-5, Unigene61721\_TAEpaeTARAAPEI-5, Unigene61723\_TAEpaeTARAAPEI-5, Unigene61744\_TAEpaeTARAAPEI-5, Unigene61761\_TAEpaeTARAAPEI-5, Unigene61809\_TAEpaeTARAAPEI-5, Unigene61845\_TAEpaeTARAAPEI-5, Unigene61846\_TAEpaeTARAAPEI-5, Unigene61892\_TAEpaeTARAAPEI-5, Unigene6190\_TAEpaeTARAAPEI-5, Unigene6193\_TAEpaeTARAAPEI-5, Unigene61958\_TAEpaeTARAAPEI-5, Unigene61963\_TAEpaeTARAAPEI-5, Unigene61987\_TAEpaeTARAAPEI-5, Unigene62045\_TAEpaeTARAAPEI-5, Unigene62060\_TAEpaeTARAAPEI-5, Unigene62133\_TAEpaeTARAAPEI-5, Unigene62192\_TAEpaeTARAAPEI-5, Unigene62198\_TAEpaeTARAAPEI-5, Unigene62205\_TAEpaeTARAAPEI-5, Unigene62226\_TAEpaeTARAAPEI-5, Unigene62231\_TAEpaeTARAAPEI-5, Unigene62283\_TAEpaeTARAAPEI-5, Unigene62294\_TAEpaeTARAAPEI-5, Unigene62320\_TAEpaeTARAAPEI-5, Unigene62322\_TAEpaeTARAAPEI-5, Unigene6234\_TAEpaeTARAAPEI-5, Unigene62433\_TAEpaeTARAAPEI-5, Unigene62444\_TAEpaeTARAAPEI-5, Unigene6244\_TAEpaeTARAAPEI-5, Unigene62472\_TAEpaeTARAAPEI-5, Unigene62481\_TAEpaeTARAAPEI-5, Unigene62501\_TAEpaeTARAAPEI-5, Unigene62549\_TAEpaeTARAAPEI-5, Unigene62552\_TAEpaeTARAAPEI-5, Unigene62641\_TAEpaeTARAAPEI-5, Unigene62651\_TAEpaeTARAAPEI-5, Unigene62673\_TAEpaeTARAAPEI-5, Unigene62686\_TAEpaeTARAAPEI-5, Unigene62692\_TAEpaeTARAAPEI-5, Unigene62700\_TAEpaeTARAAPEI-5, Unigene62702\_TAEpaeTARAAPEI-5, Unigene62774\_TAEpaeTARAAPEI-5, Unigene62780\_TAEpaeTARAAPEI-5, Unigene62782\_TAEpaeTARAAPEI-5, Unigene62783\_TAEpaeTARAAPEI-5, Unigene62857\_TAEpaeTARAAPEI-5, Unigene62896\_TAEpaeTARAAPEI-5, Unigene62900\_TAEpaeTARAAPEI-5, Unigene62929\_TAEpaeTARAAPEI-5, Unigene62944\_TAEpaeTARAAPEI-5, Unigene6294\_TAEpaeTARAAPEI-5, Unigene62954\_TAEpaeTARAAPEI-5, Unigene62980\_TAEpaeTARAAPEI-5, Unigene62986\_TAEpaeTARAAPEI-5, Unigene62989\_TAEpaeTARAAPEI-5, Unigene62990\_TAEpaeTARAAPEI-5, Unigene63013\_TAEpaeTARAAPEI-5, Unigene63039\_TAEpaeTARAAPEI-5, Unigene63078\_TAEpaeTARAAPEI-5, Unigene63094\_TAEpaeTARAAPEI-5, Unigene63138\_TAEpaeTARAAPEI-5, Unigene63144\_TAEpaeTARAAPEI-5, Unigene63180\_TAEpaeTARAAPEI-5, Unigene63196\_TAEpaeTARAAPEI-5, Unigene63246\_TAEpaeTARAAPEI-5, Unigene63256\_TAEpaeTARAAPEI-5, Unigene63284\_TAEpaeTARAAPEI-5, Unigene63309\_TAEpaeTARAAPEI-5, Unigene63312\_TAEpaeTARAAPEI-5, Unigene63333\_TAEpaeTARAAPEI-5, Unigene6334\_TAEpaeTARAAPEI-5, Unigene6338\_TAEpaeTARAAPEI-5, Unigene63428\_TAEpaeTARAAPEI-5, Unigene63432\_TAEpaeTARAAPEI-5, Unigene63436\_TAEpaeTARAAPEI-5, Unigene63438\_TAEpaeTARAAPEI-5, Unigene63441\_TAEpaeTARAAPEI-5, Unigene63455\_TAEpaeTARAAPEI-5, Unigene63478\_TAEpaeTARAAPEI-5, Unigene63479\_TAEpaeTARAAPEI-5, Unigene63486\_TAEpaeTARAAPEI-5, Unigene63511\_TAEpaeTARAAPEI-5, Unigene63547\_TAEpaeTARAAPEI-5, Unigene63588\_TAEpaeTARAAPEI-5, Unigene6362\_TAEpaeTARAAPEI-5, Unigene63641\_TAEpaeTARAAPEI-5, Unigene63655\_TAEpaeTARAAPEI-5, Unigene63658\_TAEpaeTARAAPEI-5, Unigene6365\_TAEpaeTARAAPEI-5, Unigene63709\_TAEpaeTARAAPEI-5, Unigene63711\_TAEpaeTARAAPEI-5, Unigene63827\_TAEpaeTARAAPEI-5, Unigene63838\_TAEpaeTARAAPEI-5, Unigene63852\_TAEpaeTARAAPEI-5, Unigene63864\_TAEpaeTARAAPEI-5, Unigene63866\_TAEpaeTARAAPEI-5, Unigene63870\_TAEpaeTARAAPEI-5, Unigene63877\_TAEpaeTARAAPEI-5, Unigene63888\_TAEpaeTARAAPEI-5, Unigene6389\_TAEpaeTARAAPEI-5, Unigene6396\_TAEpaeTARAAPEI-5, Unigene63973\_TAEpaeTARAAPEI-5, Unigene63978\_TAEpaeTARAAPEI-5, Unigene64003\_TAEpaeTARAAPEI-5, Unigene64010\_TAEpaeTARAAPEI-5, Unigene64013\_TAEpaeTARAAPEI-5, Unigene64036\_TAEpaeTARAAPEI-5, Unigene64063\_TAEpaeTARAAPEI-5, Unigene64081\_TAEpaeTARAAPEI-5, Unigene64088\_TAEpaeTARAAPEI-5, Unigene64100\_TAEpaeTARAAPEI-5, Unigene64102\_TAEpaeTARAAPEI-5, Unigene6410\_TAEpaeTARAAPEI-5, Unigene64117\_TAEpaeTARAAPEI-5, Unigene64126\_TAEpaeTARAAPEI-5, Unigene64128\_TAEpaeTARAAPEI-5, Unigene6412\_TAEpaeTARAAPEI-5, Unigene64142\_TAEpaeTARAAPEI-5, Unigene64170\_TAEpaeTARAAPEI-5, Unigene6417\_TAEpaeTARAAPEI-5, Unigene64191\_TAEpaeTARAAPEI-5, Unigene64198\_TAEpaeTARAAPEI-5, Unigene64227\_TAEpaeTARAAPEI-5, Unigene64230\_TAEpaeTARAAPEI-5, Unigene64253\_TAEpaeTARAAPEI-5, Unigene64264\_TAEpaeTARAAPEI-5, Unigene64349\_TAEpaeTARAAPEI-5, Unigene6434\_TAEpaeTARAAPEI-5, Unigene64368\_TAEpaeTARAAPEI-5, Unigene64392\_TAEpaeTARAAPEI-5, Unigene64426\_TAEpaeTARAAPEI-5, Unigene64430\_TAEpaeTARAAPEI-5, Unigene64449\_TAEpaeTARAAPEI-5, Unigene64462\_TAEpaeTARAAPEI-5, Unigene64471\_TAEpaeTARAAPEI-5, Unigene64518\_TAEpaeTARAAPEI-5, Unigene64519\_TAEpaeTARAAPEI-5, Unigene64563\_TAEpaeTARAAPEI-5, Unigene64566\_TAEpaeTARAAPEI-5, Unigene64568\_TAEpaeTARAAPEI-5, Unigene64572\_TAEpaeTARAAPEI-5, Unigene64579\_TAEpaeTARAAPEI-5, Unigene64585\_TAEpaeTARAAPEI-5, Unigene64654\_TAEpaeTARAAPEI-5, Unigene64691\_TAEpaeTARAAPEI-5, Unigene64698\_TAEpaeTARAAPEI-5, Unigene64702\_TAEpaeTARAAPEI-5, Unigene64712\_TAEpaeTARAAPEI-5, Unigene64717\_TAEpaeTARAAPEI-5, Unigene64804\_TAEpaeTARAAPEI-5, Unigene64811\_TAEpaeTARAAPEI-5, Unigene64847\_TAEpaeTARAAPEI-5, Unigene64852\_TAEpaeTARAAPEI-5, Unigene64964\_TAEpaeTARAAPEI-5, Unigene64969\_TAEpaeTARAAPEI-5, Unigene64971\_TAEpaeTARAAPEI-5, Unigene64995\_TAEpaeTARAAPEI-5, Unigene65017\_TAEpaeTARAAPEI-5, Unigene65028\_TAEpaeTARAAPEI-5, Unigene65045\_TAEpaeTARAAPEI-5, Unigene65108\_TAEpaeTARAAPEI-5, Unigene65134\_TAEpaeTARAAPEI-5, Unigene65157\_TAEpaeTARAAPEI-5, Unigene65165\_TAEpaeTARAAPEI-5, Unigene65211\_TAEpaeTARAAPEI-5, Unigene65245\_TAEpaeTARAAPEI-5, Unigene65272\_TAEpaeTARAAPEI-5, Unigene65273\_TAEpaeTARAAPEI-5, Unigene65277\_TAEpaeTARAAPEI-5, Unigene65278\_TAEpaeTARAAPEI-5, Unigene65280\_TAEpaeTARAAPEI-5, Unigene65292\_TAEpaeTARAAPEI-5, Unigene65307\_TAEpaeTARAAPEI-5, Unigene65332\_TAEpaeTARAAPEI-5, Unigene65333\_TAEpaeTARAAPEI-5, Unigene65338\_TAEpaeTARAAPEI-5, Unigene65341\_TAEpaeTARAAPEI-5, Unigene65343\_TAEpaeTARAAPEI-5, Unigene65415\_TAEpaeTARAAPEI-5, Unigene65421\_TAEpaeTARAAPEI-5, Unigene65446\_TAEpaeTARAAPEI-5, Unigene65464\_TAEpaeTARAAPEI-5, Unigene65465\_TAEpaeTARAAPEI-5, Unigene65488\_TAEpaeTARAAPEI-5, Unigene65506\_TAEpaeTARAAPEI-5, Unigene65518\_TAEpaeTARAAPEI-5, Unigene6552\_TAEpaeTARAAPEI-5, Unigene65606\_TAEpaeTARAAPEI-5, Unigene65634\_TAEpaeTARAAPEI-5, Unigene65635\_TAEpaeTARAAPEI-5, Unigene65636\_TAEpaeTARAAPEI-5, Unigene65655\_TAEpaeTARAAPEI-5, Unigene65657\_TAEpaeTARAAPEI-5, Unigene65663\_TAEpaeTARAAPEI-5, Unigene65749\_TAEpaeTARAAPEI-5, Unigene65751\_TAEpaeTARAAPEI-5, Unigene65757\_TAEpaeTARAAPEI-5, Unigene65770\_TAEpaeTARAAPEI-5, Unigene65782\_TAEpaeTARAAPEI-5, Unigene65794\_TAEpaeTARAAPEI-5, Unigene65821\_TAEpaeTARAAPEI-5, Unigene65824\_TAEpaeTARAAPEI-5, Unigene65839\_TAEpaeTARAAPEI-5, Unigene65842\_TAEpaeTARAAPEI-5, Unigene65854\_TAEpaeTARAAPEI-5, Unigene65875\_TAEpaeTARAAPEI-5, Unigene65899\_TAEpaeTARAAPEI-5, Unigene65902\_TAEpaeTARAAPEI-5, Unigene65940\_TAEpaeTARAAPEI-5, Unigene65961\_TAEpaeTARAAPEI-5, Unigene65980\_TAEpaeTARAAPEI-5, Unigene65993\_TAEpaeTARAAPEI-5, Unigene65996\_TAEpaeTARAAPEI-5, Unigene65998\_TAEpaeTARAAPEI-5, Unigene66001\_TAEpaeTARAAPEI-5, Unigene66029\_TAEpaeTARAAPEI-5, Unigene66035\_TAEpaeTARAAPEI-5, Unigene66053\_TAEpaeTARAAPEI-5, Unigene6608\_TAEpaeTARAAPEI-5, Unigene66111\_TAEpaeTARAAPEI-5, Unigene66121\_TAEpaeTARAAPEI-5, Unigene66137\_TAEpaeTARAAPEI-5, Unigene66142\_TAEpaeTARAAPEI-5, Unigene6615\_TAEpaeTARAAPEI-5, Unigene66161\_TAEpaeTARAAPEI-5, Unigene66166\_TAEpaeTARAAPEI-5, Unigene66177\_TAEpaeTARAAPEI-5, Unigene66202\_TAEpaeTARAAPEI-5, Unigene66204\_TAEpaeTARAAPEI-5, Unigene66216\_TAEpaeTARAAPEI-5, Unigene6622\_TAEpaeTARAAPEI-5, Unigene66290\_TAEpaeTARAAPEI-5, Unigene66293\_TAEpaeTARAAPEI-5, Unigene66305\_TAEpaeTARAAPEI-5, Unigene66306\_TAEpaeTARAAPEI-5, Unigene66338\_TAEpaeTARAAPEI-5, Unigene66421\_TAEpaeTARAAPEI-5, Unigene66427\_TAEpaeTARAAPEI-5, Unigene66451\_TAEpaeTARAAPEI-5, Unigene66526\_TAEpaeTARAAPEI-5, Unigene66532\_TAEpaeTARAAPEI-5, Unigene66557\_TAEpaeTARAAPEI-5, Unigene66562\_TAEpaeTARAAPEI-5, Unigene66583\_TAEpaeTARAAPEI-5, Unigene66608\_TAEpaeTARAAPEI-5, Unigene66610\_TAEpaeTARAAPEI-5, Unigene66612\_TAEpaeTARAAPEI-5, Unigene66619\_TAEpaeTARAAPEI-5, Unigene66625\_TAEpaeTARAAPEI-5, Unigene6662\_TAEpaeTARAAPEI-5, Unigene6663\_TAEpaeTARAAPEI-5, Unigene66649\_TAEpaeTARAAPEI-5, Unigene6664\_TAEpaeTARAAPEI-5, Unigene66658\_TAEpaeTARAAPEI-5, Unigene66670\_TAEpaeTARAAPEI-5, Unigene66672\_TAEpaeTARAAPEI-5, Unigene66673\_TAEpaeTARAAPEI-5, Unigene66699\_TAEpaeTARAAPEI-5, Unigene66714\_TAEpaeTARAAPEI-5, Unigene66754\_TAEpaeTARAAPEI-5, Unigene66766\_TAEpaeTARAAPEI-5, Unigene66773\_TAEpaeTARAAPEI-5, Unigene66790\_TAEpaeTARAAPEI-5, Unigene66807\_TAEpaeTARAAPEI-5, Unigene66828\_TAEpaeTARAAPEI-5, Unigene66832\_TAEpaeTARAAPEI-5, Unigene66835\_TAEpaeTARAAPEI-5, Unigene66856\_TAEpaeTARAAPEI-5, Unigene66858\_TAEpaeTARAAPEI-5, Unigene66859\_TAEpaeTARAAPEI-5, Unigene66865\_TAEpaeTARAAPEI-5, Unigene66886\_TAEpaeTARAAPEI-5, Unigene66892\_TAEpaeTARAAPEI-5, Unigene66899\_TAEpaeTARAAPEI-5, Unigene66908\_TAEpaeTARAAPEI-5, Unigene66949\_TAEpaeTARAAPEI-5, Unigene66952\_TAEpaeTARAAPEI-5, Unigene66971\_TAEpaeTARAAPEI-5, Unigene66985\_TAEpaeTARAAPEI-5, Unigene67037\_TAEpaeTARAAPEI-5, Unigene67049\_TAEpaeTARAAPEI-5, Unigene67050\_TAEpaeTARAAPEI-5, Unigene67068\_TAEpaeTARAAPEI-5, Unigene67097\_TAEpaeTARAAPEI-5, Unigene67109\_TAEpaeTARAAPEI-5, Unigene67126\_TAEpaeTARAAPEI-5, Unigene67136\_TAEpaeTARAAPEI-5, Unigene67163\_TAEpaeTARAAPEI-5, Unigene67185\_TAEpaeTARAAPEI-5, Unigene6720\_TAEpaeTARAAPEI-5, Unigene67223\_TAEpaeTARAAPEI-5, Unigene67332\_TAEpaeTARAAPEI-5, Unigene67362\_TAEpaeTARAAPEI-5, Unigene67392\_TAEpaeTARAAPEI-5, Unigene67400\_TAEpaeTARAAPEI-5, Unigene67454\_TAEpaeTARAAPEI-5, Unigene67459\_TAEpaeTARAAPEI-5, Unigene67480\_TAEpaeTARAAPEI-5, Unigene67509\_TAEpaeTARAAPEI-5, Unigene67539\_TAEpaeTARAAPEI-5, Unigene67550\_TAEpaeTARAAPEI-5, Unigene67568\_TAEpaeTARAAPEI-5, Unigene67584\_TAEpaeTARAAPEI-5, Unigene67590\_TAEpaeTARAAPEI-5, Unigene67597\_TAEpaeTARAAPEI-5, Unigene67598\_TAEpaeTARAAPEI-5, Unigene67600\_TAEpaeTARAAPEI-5, Unigene67616\_TAEpaeTARAAPEI-5, Unigene67631\_TAEpaeTARAAPEI-5, Unigene67643\_TAEpaeTARAAPEI-5, Unigene67656\_TAEpaeTARAAPEI-5, Unigene67684\_TAEpaeTARAAPEI-5, Unigene67720\_TAEpaeTARAAPEI-5, Unigene67754\_TAEpaeTARAAPEI-5, Unigene67831\_TAEpaeTARAAPEI-5, Unigene67837\_TAEpaeTARAAPEI-5, Unigene67839\_TAEpaeTARAAPEI-5, Unigene67860\_TAEpaeTARAAPEI-5, Unigene67861\_TAEpaeTARAAPEI-5, Unigene67876\_TAEpaeTARAAPEI-5, Unigene67888\_TAEpaeTARAAPEI-5, Unigene67921\_TAEpaeTARAAPEI-5, Unigene67965\_TAEpaeTARAAPEI-5, Unigene67986\_TAEpaeTARAAPEI-5, Unigene67996\_TAEpaeTARAAPEI-5, Unigene68021\_TAEpaeTARAAPEI-5, Unigene68022\_TAEpaeTARAAPEI-5, Unigene68029\_TAEpaeTARAAPEI-5, Unigene68033\_TAEpaeTARAAPEI-5, Unigene68038\_TAEpaeTARAAPEI-5, Unigene68055\_TAEpaeTARAAPEI-5, Unigene68072\_TAEpaeTARAAPEI-5, Unigene68099\_TAEpaeTARAAPEI-5, Unigene68139\_TAEpaeTARAAPEI-5, Unigene68146\_TAEpaeTARAAPEI-5, Unigene68168\_TAEpaeTARAAPEI-5, Unigene68172\_TAEpaeTARAAPEI-5, Unigene68179\_TAEpaeTARAAPEI-5, Unigene68181\_TAEpaeTARAAPEI-5, Unigene68189\_TAEpaeTARAAPEI-5, Unigene68193\_TAEpaeTARAAPEI-5, Unigene68195\_TAEpaeTARAAPEI-5, Unigene68204\_TAEpaeTARAAPEI-5, Unigene68217\_TAEpaeTARAAPEI-5, Unigene68228\_TAEpaeTARAAPEI-5, Unigene68252\_TAEpaeTARAAPEI-5, Unigene68260\_TAEpaeTARAAPEI-5, Unigene68261\_TAEpaeTARAAPEI-5, Unigene68271\_TAEpaeTARAAPEI-5, Unigene68316\_TAEpaeTARAAPEI-5, Unigene68321\_TAEpaeTARAAPEI-5, Unigene68341\_TAEpaeTARAAPEI-5, Unigene68347\_TAEpaeTARAAPEI-5, Unigene68381\_TAEpaeTARAAPEI-5, Unigene68395\_TAEpaeTARAAPEI-5, Unigene68396\_TAEpaeTARAAPEI-5, Unigene68464\_TAEpaeTARAAPEI-5, Unigene68473\_TAEpaeTARAAPEI-5, Unigene68483\_TAEpaeTARAAPEI-5, Unigene68494\_TAEpaeTARAAPEI-5, Unigene68525\_TAEpaeTARAAPEI-5, Unigene68574\_TAEpaeTARAAPEI-5, Unigene68607\_TAEpaeTARAAPEI-5, Unigene68615\_TAEpaeTARAAPEI-5, Unigene68639\_TAEpaeTARAAPEI-5, Unigene68651\_TAEpaeTARAAPEI-5, Unigene68654\_TAEpaeTARAAPEI-5, Unigene6866\_TAEpaeTARAAPEI-5, Unigene68677\_TAEpaeTARAAPEI-5, Unigene68702\_TAEpaeTARAAPEI-5, Unigene68730\_TAEpaeTARAAPEI-5, Unigene68731\_TAEpaeTARAAPEI-5, Unigene68734\_TAEpaeTARAAPEI-5, Unigene68742\_TAEpaeTARAAPEI-5, Unigene68781\_TAEpaeTARAAPEI-5, Unigene68810\_TAEpaeTARAAPEI-5, Unigene68876\_TAEpaeTARAAPEI-5, Unigene68881\_TAEpaeTARAAPEI-5, Unigene68891\_TAEpaeTARAAPEI-5, Unigene68901\_TAEpaeTARAAPEI-5, Unigene68911\_TAEpaeTARAAPEI-5, Unigene68974\_TAEpaeTARAAPEI-5, Unigene69009\_TAEpaeTARAAPEI-5, Unigene69050\_TAEpaeTARAAPEI-5, Unigene69069\_TAEpaeTARAAPEI-5, Unigene69116\_TAEpaeTARAAPEI-5, Unigene69120\_TAEpaeTARAAPEI-5, Unigene69126\_TAEpaeTARAAPEI-5, Unigene69143\_TAEpaeTARAAPEI-5, Unigene69171\_TAEpaeTARAAPEI-5, Unigene69176\_TAEpaeTARAAPEI-5, Unigene69226\_TAEpaeTARAAPEI-5, Unigene69266\_TAEpaeTARAAPEI-5, Unigene69270\_TAEpaeTARAAPEI-5, Unigene69285\_TAEpaeTARAAPEI-5, Unigene69298\_TAEpaeTARAAPEI-5, Unigene69306\_TAEpaeTARAAPEI-5, Unigene69309\_TAEpaeTARAAPEI-5, Unigene69349\_TAEpaeTARAAPEI-5, Unigene69382\_TAEpaeTARAAPEI-5, Unigene69383\_TAEpaeTARAAPEI-5, Unigene69403\_TAEpaeTARAAPEI-5, Unigene69424\_TAEpaeTARAAPEI-5, Unigene69434\_TAEpaeTARAAPEI-5, Unigene69436\_TAEpaeTARAAPEI-5, Unigene6943\_TAEpaeTARAAPEI-5, Unigene69442\_TAEpaeTARAAPEI-5, Unigene69444\_TAEpaeTARAAPEI-5, Unigene69463\_TAEpaeTARAAPEI-5, Unigene69521\_TAEpaeTARAAPEI-5, Unigene69536\_TAEpaeTARAAPEI-5, Unigene69541\_TAEpaeTARAAPEI-5, Unigene69543\_TAEpaeTARAAPEI-5, Unigene69557\_TAEpaeTARAAPEI-5, Unigene69570\_TAEpaeTARAAPEI-5, Unigene69571\_TAEpaeTARAAPEI-5, Unigene6958\_TAEpaeTARAAPEI-5, Unigene69611\_TAEpaeTARAAPEI-5, Unigene69645\_TAEpaeTARAAPEI-5, Unigene69662\_TAEpaeTARAAPEI-5, Unigene69663\_TAEpaeTARAAPEI-5, Unigene69665\_TAEpaeTARAAPEI-5, Unigene69704\_TAEpaeTARAAPEI-5, Unigene69715\_TAEpaeTARAAPEI-5, Unigene69721\_TAEpaeTARAAPEI-5, Unigene69744\_TAEpaeTARAAPEI-5, Unigene69748\_TAEpaeTARAAPEI-5, Unigene69764\_TAEpaeTARAAPEI-5, Unigene69784\_TAEpaeTARAAPEI-5, Unigene69800\_TAEpaeTARAAPEI-5, Unigene69836\_TAEpaeTARAAPEI-5, Unigene69879\_TAEpaeTARAAPEI-5, Unigene69892\_TAEpaeTARAAPEI-5, Unigene69900\_TAEpaeTARAAPEI-5, Unigene69920\_TAEpaeTARAAPEI-5, Unigene69938\_TAEpaeTARAAPEI-5, Unigene69950\_TAEpaeTARAAPEI-5, Unigene69973\_TAEpaeTARAAPEI-5, Unigene69975\_TAEpaeTARAAPEI-5, Unigene70012\_TAEpaeTARAAPEI-5, Unigene70013\_TAEpaeTARAAPEI-5, Unigene70017\_TAEpaeTARAAPEI-5, Unigene70024\_TAEpaeTARAAPEI-5, Unigene70031\_TAEpaeTARAAPEI-5, Unigene70040\_TAEpaeTARAAPEI-5, Unigene70065\_TAEpaeTARAAPEI-5, Unigene70070\_TAEpaeTARAAPEI-5, Unigene70080\_TAEpaeTARAAPEI-5, Unigene70123\_TAEpaeTARAAPEI-5, Unigene7012\_TAEpaeTARAAPEI-5, Unigene70135\_TAEpaeTARAAPEI-5, Unigene70139\_TAEpaeTARAAPEI-5, Unigene70156\_TAEpaeTARAAPEI-5, Unigene70205\_TAEpaeTARAAPEI-5, Unigene70208\_TAEpaeTARAAPEI-5, Unigene70209\_TAEpaeTARAAPEI-5, Unigene70213\_TAEpaeTARAAPEI-5, Unigene7021\_TAEpaeTARAAPEI-5, Unigene70261\_TAEpaeTARAAPEI-5, Unigene70265\_TAEpaeTARAAPEI-5, Unigene70284\_TAEpaeTARAAPEI-5, Unigene70317\_TAEpaeTARAAPEI-5, Unigene70359\_TAEpaeTARAAPEI-5, Unigene70386\_TAEpaeTARAAPEI-5, Unigene70387\_TAEpaeTARAAPEI-5, Unigene70429\_TAEpaeTARAAPEI-5, Unigene70434\_TAEpaeTARAAPEI-5, Unigene70441\_TAEpaeTARAAPEI-5, Unigene70442\_TAEpaeTARAAPEI-5, Unigene70458\_TAEpaeTARAAPEI-5, Unigene70468\_TAEpaeTARAAPEI-5, Unigene70478\_TAEpaeTARAAPEI-5, Unigene70502\_TAEpaeTARAAPEI-5, Unigene70518\_TAEpaeTARAAPEI-5, Unigene70521\_TAEpaeTARAAPEI-5, Unigene70523\_TAEpaeTARAAPEI-5, Unigene70531\_TAEpaeTARAAPEI-5, Unigene70560\_TAEpaeTARAAPEI-5, Unigene7057\_TAEpaeTARAAPEI-5, Unigene70628\_TAEpaeTARAAPEI-5, Unigene70631\_TAEpaeTARAAPEI-5, Unigene70632\_TAEpaeTARAAPEI-5, Unigene70633\_TAEpaeTARAAPEI-5, Unigene70673\_TAEpaeTARAAPEI-5, Unigene70679\_TAEpaeTARAAPEI-5, Unigene70698\_TAEpaeTARAAPEI-5, Unigene70730\_TAEpaeTARAAPEI-5, Unigene70741\_TAEpaeTARAAPEI-5, Unigene70750\_TAEpaeTARAAPEI-5, Unigene70780\_TAEpaeTARAAPEI-5, Unigene70782\_TAEpaeTARAAPEI-5, Unigene70792\_TAEpaeTARAAPEI-5, Unigene70795\_TAEpaeTARAAPEI-5, Unigene70806\_TAEpaeTARAAPEI-5, Unigene70845\_TAEpaeTARAAPEI-5, Unigene70888\_TAEpaeTARAAPEI-5, Unigene70889\_TAEpaeTARAAPEI-5, Unigene70894\_TAEpaeTARAAPEI-5, Unigene70905\_TAEpaeTARAAPEI-5, Unigene70954\_TAEpaeTARAAPEI-5, Unigene70972\_TAEpaeTARAAPEI-5, Unigene70973\_TAEpaeTARAAPEI-5, Unigene70994\_TAEpaeTARAAPEI-5, Unigene70995\_TAEpaeTARAAPEI-5, Unigene70\_TAEpaeTARAAPEI-5, Unigene71017\_TAEpaeTARAAPEI-5, Unigene71052\_TAEpaeTARAAPEI-5, Unigene71062\_TAEpaeTARAAPEI-5, Unigene71091\_TAEpaeTARAAPEI-5, Unigene71130\_TAEpaeTARAAPEI-5, Unigene71138\_TAEpaeTARAAPEI-5, Unigene7114\_TAEpaeTARAAPEI-5, Unigene71157\_TAEpaeTARAAPEI-5, Unigene71167\_TAEpaeTARAAPEI-5, Unigene71205\_TAEpaeTARAAPEI-5, Unigene71217\_TAEpaeTARAAPEI-5, Unigene71245\_TAEpaeTARAAPEI-5, Unigene71251\_TAEpaeTARAAPEI-5, Unigene71278\_TAEpaeTARAAPEI-5, Unigene71292\_TAEpaeTARAAPEI-5, Unigene71315\_TAEpaeTARAAPEI-5, Unigene71332\_TAEpaeTARAAPEI-5, Unigene71351\_TAEpaeTARAAPEI-5, Unigene71355\_TAEpaeTARAAPEI-5, Unigene71365\_TAEpaeTARAAPEI-5, Unigene71402\_TAEpaeTARAAPEI-5, Unigene71403\_TAEpaeTARAAPEI-5, Unigene71407\_TAEpaeTARAAPEI-5, Unigene71424\_TAEpaeTARAAPEI-5, Unigene71429\_TAEpaeTARAAPEI-5, Unigene71438\_TAEpaeTARAAPEI-5, Unigene71461\_TAEpaeTARAAPEI-5, Unigene71498\_TAEpaeTARAAPEI-5, Unigene71520\_TAEpaeTARAAPEI-5, Unigene71529\_TAEpaeTARAAPEI-5, Unigene71550\_TAEpaeTARAAPEI-5, Unigene71565\_TAEpaeTARAAPEI-5, Unigene71571\_TAEpaeTARAAPEI-5, Unigene71587\_TAEpaeTARAAPEI-5, Unigene71602\_TAEpaeTARAAPEI-5, Unigene71636\_TAEpaeTARAAPEI-5, Unigene7164\_TAEpaeTARAAPEI-5, Unigene71676\_TAEpaeTARAAPEI-5, Unigene71682\_TAEpaeTARAAPEI-5, Unigene71687\_TAEpaeTARAAPEI-5, Unigene71700\_TAEpaeTARAAPEI-5, Unigene71713\_TAEpaeTARAAPEI-5, Unigene71730\_TAEpaeTARAAPEI-5, Unigene71774\_TAEpaeTARAAPEI-5, Unigene71797\_TAEpaeTARAAPEI-5, Unigene71799\_TAEpaeTARAAPEI-5, Unigene71820\_TAEpaeTARAAPEI-5, Unigene71822\_TAEpaeTARAAPEI-5, Unigene71837\_TAEpaeTARAAPEI-5, Unigene71840\_TAEpaeTARAAPEI-5, Unigene71842\_TAEpaeTARAAPEI-5, Unigene71850\_TAEpaeTARAAPEI-5, Unigene71857\_TAEpaeTARAAPEI-5, Unigene71876\_TAEpaeTARAAPEI-5, Unigene71878\_TAEpaeTARAAPEI-5, Unigene71881\_TAEpaeTARAAPEI-5, Unigene71889\_TAEpaeTARAAPEI-5, Unigene71896\_TAEpaeTARAAPEI-5, Unigene71904\_TAEpaeTARAAPEI-5, Unigene71918\_TAEpaeTARAAPEI-5, Unigene71929\_TAEpaeTARAAPEI-5, Unigene71934\_TAEpaeTARAAPEI-5, Unigene71935\_TAEpaeTARAAPEI-5, Unigene71945\_TAEpaeTARAAPEI-5, Unigene71957\_TAEpaeTARAAPEI-5, Unigene71989\_TAEpaeTARAAPEI-5, Unigene71993\_TAEpaeTARAAPEI-5, Unigene71998\_TAEpaeTARAAPEI-5, Unigene7199\_TAEpaeTARAAPEI-5, Unigene72009\_TAEpaeTARAAPEI-5, Unigene72014\_TAEpaeTARAAPEI-5, Unigene72086\_TAEpaeTARAAPEI-5, Unigene72099\_TAEpaeTARAAPEI-5, Unigene72104\_TAEpaeTARAAPEI-5, Unigene7211\_TAEpaeTARAAPEI-5, Unigene72172\_TAEpaeTARAAPEI-5, Unigene72189\_TAEpaeTARAAPEI-5, Unigene7219\_TAEpaeTARAAPEI-5, Unigene72235\_TAEpaeTARAAPEI-5, Unigene72237\_TAEpaeTARAAPEI-5, Unigene72240\_TAEpaeTARAAPEI-5, Unigene72250\_TAEpaeTARAAPEI-5, Unigene72256\_TAEpaeTARAAPEI-5, Unigene72263\_TAEpaeTARAAPEI-5, Unigene72285\_TAEpaeTARAAPEI-5, Unigene72310\_TAEpaeTARAAPEI-5, Unigene72316\_TAEpaeTARAAPEI-5, Unigene72334\_TAEpaeTARAAPEI-5, Unigene72340\_TAEpaeTARAAPEI-5, Unigene72374\_TAEpaeTARAAPEI-5, Unigene7237\_TAEpaeTARAAPEI-5, Unigene72393\_TAEpaeTARAAPEI-5, Unigene72399\_TAEpaeTARAAPEI-5, Unigene72404\_TAEpaeTARAAPEI-5, Unigene72412\_TAEpaeTARAAPEI-5, Unigene72444\_TAEpaeTARAAPEI-5, Unigene72453\_TAEpaeTARAAPEI-5, Unigene72458\_TAEpaeTARAAPEI-5, Unigene72462\_TAEpaeTARAAPEI-5, Unigene72472\_TAEpaeTARAAPEI-5, Unigene72490\_TAEpaeTARAAPEI-5, Unigene72500\_TAEpaeTARAAPEI-5, Unigene72520\_TAEpaeTARAAPEI-5, Unigene72521\_TAEpaeTARAAPEI-5, Unigene72528\_TAEpaeTARAAPEI-5, Unigene72531\_TAEpaeTARAAPEI-5, Unigene72574\_TAEpaeTARAAPEI-5, Unigene72576\_TAEpaeTARAAPEI-5, Unigene72598\_TAEpaeTARAAPEI-5, Unigene725\_TAEpaeTARAAPEI-5, Unigene72641\_TAEpaeTARAAPEI-5, Unigene72652\_TAEpaeTARAAPEI-5, Unigene72669\_TAEpaeTARAAPEI-5, Unigene72705\_TAEpaeTARAAPEI-5, Unigene72738\_TAEpaeTARAAPEI-5, Unigene72745\_TAEpaeTARAAPEI-5, Unigene72746\_TAEpaeTARAAPEI-5, Unigene72747\_TAEpaeTARAAPEI-5, Unigene72756\_TAEpaeTARAAPEI-5, Unigene72775\_TAEpaeTARAAPEI-5, Unigene72780\_TAEpaeTARAAPEI-5, Unigene72785\_TAEpaeTARAAPEI-5, Unigene72789\_TAEpaeTARAAPEI-5, Unigene72807\_TAEpaeTARAAPEI-5, Unigene72871\_TAEpaeTARAAPEI-5, Unigene72938\_TAEpaeTARAAPEI-5, Unigene7401\_TAEpaeTARAAPEI-5, Unigene7435\_TAEpaeTARAAPEI-5, Unigene7456\_TAEpaeTARAAPEI-5, Unigene7461\_TAEpaeTARAAPEI-5, Unigene7513\_TAEpaeTARAAPEI-5, Unigene7627\_TAEpaeTARAAPEI-5, Unigene7677\_TAEpaeTARAAPEI-5, Unigene7687\_TAEpaeTARAAPEI-5, Unigene7696\_TAEpaeTARAAPEI-5, Unigene7718\_TAEpaeTARAAPEI-5, Unigene7720\_TAEpaeTARAAPEI-5, Unigene7726\_TAEpaeTARAAPEI-5, Unigene7737\_TAEpaeTARAAPEI-5, Unigene7773\_TAEpaeTARAAPEI-5, Unigene7781\_TAEpaeTARAAPEI-5, Unigene7800\_TAEpaeTARAAPEI-5, Unigene7825\_TAEpaeTARAAPEI-5, Unigene7848\_TAEpaeTARAAPEI-5, Unigene7863\_TAEpaeTARAAPEI-5, Unigene7990\_TAEpaeTARAAPEI-5, Unigene8000\_TAEpaeTARAAPEI-5, Unigene8049\_TAEpaeTARAAPEI-5, Unigene8058\_TAEpaeTARAAPEI-5, Unigene8135\_TAEpaeTARAAPEI-5, Unigene8189\_TAEpaeTARAAPEI-5, Unigene8206\_TAEpaeTARAAPEI-5, Unigene8272\_TAEpaeTARAAPEI-5, Unigene8289\_TAEpaeTARAAPEI-5, Unigene8303\_TAEpaeTARAAPEI-5, Unigene8316\_TAEpaeTARAAPEI-5, Unigene8318\_TAEpaeTARAAPEI-5, Unigene8332\_TAEpaeTARAAPEI-5, Unigene8360\_TAEpaeTARAAPEI-5, Unigene8400\_TAEpaeTARAAPEI-5, Unigene8407\_TAEpaeTARAAPEI-5, Unigene8410\_TAEpaeTARAAPEI-5, Unigene8412\_TAEpaeTARAAPEI-5, Unigene841\_TAEpaeTARAAPEI-5, Unigene8425\_TAEpaeTARAAPEI-5, Unigene8464\_TAEpaeTARAAPEI-5, Unigene8473\_TAEpaeTARAAPEI-5, Unigene8477\_TAEpaeTARAAPEI-5, Unigene8478\_TAEpaeTARAAPEI-5, Unigene8539\_TAEpaeTARAAPEI-5, Unigene8546\_TAEpaeTARAAPEI-5, Unigene8581\_TAEpaeTARAAPEI-5, Unigene858\_TAEpaeTARAAPEI-5, Unigene8602\_TAEpaeTARAAPEI-5, Unigene8605\_TAEpaeTARAAPEI-5, Unigene861\_TAEpaeTARAAPEI-5, Unigene8682\_TAEpaeTARAAPEI-5, Unigene8714\_TAEpaeTARAAPEI-5, Unigene8718\_TAEpaeTARAAPEI-5, Unigene8759\_TAEpaeTARAAPEI-5, Unigene8772\_TAEpaeTARAAPEI-5, Unigene8780\_TAEpaeTARAAPEI-5, Unigene8816\_TAEpaeTARAAPEI-5, Unigene8862\_TAEpaeTARAAPEI-5, Unigene8878\_TAEpaeTARAAPEI-5, Unigene8927\_TAEpaeTARAAPEI-5, Unigene8934\_TAEpaeTARAAPEI-5, Unigene8936\_TAEpaeTARAAPEI-5, Unigene8974\_TAEpaeTARAAPEI-5, Unigene9005\_TAEpaeTARAAPEI-5, Unigene9025\_TAEpaeTARAAPEI-5, Unigene903\_TAEpaeTARAAPEI-5, Unigene907\_TAEpaeTARAAPEI-5, Unigene9118\_TAEpaeTARAAPEI-5, Unigene9120\_TAEpaeTARAAPEI-5, Unigene9152\_TAEpaeTARAAPEI-5, Unigene9216\_TAEpaeTARAAPEI-5, Unigene9272\_TAEpaeTARAAPEI-5, Unigene9294\_TAEpaeTARAAPEI-5, Unigene9320\_TAEpaeTARAAPEI-5, Unigene9393\_TAEpaeTARAAPEI-5, Unigene9423\_TAEpaeTARAAPEI-5, Unigene9430\_TAEpaeTARAAPEI-5, Unigene9582\_TAEpaeTARAAPEI-5, Unigene9584\_TAEpaeTARAAPEI-5, Unigene9589\_TAEpaeTARAAPEI-5, Unigene9603\_TAEpaeTARAAPEI-5, Unigene9626\_TAEpaeTARAAPEI-5, Unigene9648\_TAEpaeTARAAPEI-5, Unigene9651\_TAEpaeTARAAPEI-5, Unigene965\_TAEpaeTARAAPEI-5, Unigene9670\_TAEpaeTARAAPEI-5, Unigene9677\_TAEpaeTARAAPEI-5, Unigene9688\_TAEpaeTARAAPEI-5, Unigene9753\_TAEpaeTARAAPEI-5, Unigene9767\_TAEpaeTARAAPEI-5, Unigene978\_TAEpaeTARAAPEI-5, Unigene9827\_TAEpaeTARAAPEI-5, Unigene9845\_TAEpaeTARAAPEI-5, Unigene9851\_TAEpaeTARAAPEI-5, Unigene98\_TAEpaeTARAAPEI-5, Unigene9900\_TAEpaeTARAAPEI-5, Unigene9910\_TAEpaeTARAAPEI-5, Unigene9961\_TAEpaeTARAAPEI-5, Unigene9975\_TAEpaeTARAAPEI-5, Unigene9986\_TAEpaeTARAAPEI-5 |
| 2 | Spliceosome | Unigene10010\_TAEpaeTARAAPEI-5, Unigene10055\_TAEpaeTARAAPEI-5, Unigene10079\_TAEpaeTARAAPEI-5, Unigene10358\_TAEpaeTARAAPEI-5, Unigene10418\_TAEpaeTARAAPEI-5, Unigene10546\_TAEpaeTARAAPEI-5, Unigene10794\_TAEpaeTARAAPEI-5, Unigene10815\_TAEpaeTARAAPEI-5, Unigene10988\_TAEpaeTARAAPEI-5, Unigene11042\_TAEpaeTARAAPEI-5, Unigene11117\_TAEpaeTARAAPEI-5, Unigene11166\_TAEpaeTARAAPEI-5, Unigene11188\_TAEpaeTARAAPEI-5, Unigene11433\_TAEpaeTARAAPEI-5, Unigene11474\_TAEpaeTARAAPEI-5, Unigene11577\_TAEpaeTARAAPEI-5, Unigene11628\_TAEpaeTARAAPEI-5, Unigene11668\_TAEpaeTARAAPEI-5, Unigene11691\_TAEpaeTARAAPEI-5, Unigene11722\_TAEpaeTARAAPEI-5, Unigene11801\_TAEpaeTARAAPEI-5, Unigene11921\_TAEpaeTARAAPEI-5, Unigene11949\_TAEpaeTARAAPEI-5, Unigene12028\_TAEpaeTARAAPEI-5, Unigene12199\_TAEpaeTARAAPEI-5, Unigene12362\_TAEpaeTARAAPEI-5, Unigene12424\_TAEpaeTARAAPEI-5, Unigene12596\_TAEpaeTARAAPEI-5, Unigene12681\_TAEpaeTARAAPEI-5, Unigene12779\_TAEpaeTARAAPEI-5, Unigene13073\_TAEpaeTARAAPEI-5, Unigene1317\_TAEpaeTARAAPEI-5, Unigene13257\_TAEpaeTARAAPEI-5, Unigene13446\_TAEpaeTARAAPEI-5, Unigene1348\_TAEpaeTARAAPEI-5, Unigene13559\_TAEpaeTARAAPEI-5, Unigene13709\_TAEpaeTARAAPEI-5, Unigene1376\_TAEpaeTARAAPEI-5, Unigene14086\_TAEpaeTARAAPEI-5, Unigene140\_TAEpaeTARAAPEI-5, Unigene14381\_TAEpaeTARAAPEI-5, Unigene14484\_TAEpaeTARAAPEI-5, Unigene14542\_TAEpaeTARAAPEI-5, Unigene14729\_TAEpaeTARAAPEI-5, Unigene14740\_TAEpaeTARAAPEI-5, Unigene14745\_TAEpaeTARAAPEI-5, Unigene14782\_TAEpaeTARAAPEI-5, Unigene14855\_TAEpaeTARAAPEI-5, Unigene14869\_TAEpaeTARAAPEI-5, Unigene14918\_TAEpaeTARAAPEI-5, Unigene14921\_TAEpaeTARAAPEI-5, Unigene14985\_TAEpaeTARAAPEI-5, Unigene14991\_TAEpaeTARAAPEI-5, Unigene15238\_TAEpaeTARAAPEI-5, Unigene15250\_TAEpaeTARAAPEI-5, Unigene15364\_TAEpaeTARAAPEI-5, Unigene15390\_TAEpaeTARAAPEI-5, Unigene15475\_TAEpaeTARAAPEI-5, Unigene1562\_TAEpaeTARAAPEI-5, Unigene15683\_TAEpaeTARAAPEI-5, Unigene15700\_TAEpaeTARAAPEI-5, Unigene15833\_TAEpaeTARAAPEI-5, Unigene16183\_TAEpaeTARAAPEI-5, Unigene16217\_TAEpaeTARAAPEI-5, Unigene1624\_TAEpaeTARAAPEI-5, Unigene16273\_TAEpaeTARAAPEI-5, Unigene16299\_TAEpaeTARAAPEI-5, Unigene16360\_TAEpaeTARAAPEI-5, Unigene16380\_TAEpaeTARAAPEI-5, Unigene16482\_TAEpaeTARAAPEI-5, Unigene1654\_TAEpaeTARAAPEI-5, Unigene16567\_TAEpaeTARAAPEI-5, Unigene16649\_TAEpaeTARAAPEI-5, Unigene16679\_TAEpaeTARAAPEI-5, Unigene16709\_TAEpaeTARAAPEI-5, Unigene16857\_TAEpaeTARAAPEI-5, Unigene16874\_TAEpaeTARAAPEI-5, Unigene16904\_TAEpaeTARAAPEI-5, Unigene17033\_TAEpaeTARAAPEI-5, Unigene17209\_TAEpaeTARAAPEI-5, Unigene17271\_TAEpaeTARAAPEI-5, Unigene17330\_TAEpaeTARAAPEI-5, Unigene17357\_TAEpaeTARAAPEI-5, Unigene17415\_TAEpaeTARAAPEI-5, Unigene17442\_TAEpaeTARAAPEI-5, Unigene17689\_TAEpaeTARAAPEI-5, Unigene17697\_TAEpaeTARAAPEI-5, Unigene17743\_TAEpaeTARAAPEI-5, Unigene17838\_TAEpaeTARAAPEI-5, Unigene17874\_TAEpaeTARAAPEI-5, Unigene17876\_TAEpaeTARAAPEI-5, Unigene18024\_TAEpaeTARAAPEI-5, Unigene18105\_TAEpaeTARAAPEI-5, Unigene18206\_TAEpaeTARAAPEI-5, Unigene18497\_TAEpaeTARAAPEI-5, Unigene18672\_TAEpaeTARAAPEI-5, Unigene1873\_TAEpaeTARAAPEI-5, Unigene18740\_TAEpaeTARAAPEI-5, Unigene18955\_TAEpaeTARAAPEI-5, Unigene19695\_TAEpaeTARAAPEI-5, Unigene2001\_TAEpaeTARAAPEI-5, Unigene20269\_TAEpaeTARAAPEI-5, Unigene20293\_TAEpaeTARAAPEI-5, Unigene20481\_TAEpaeTARAAPEI-5, Unigene20571\_TAEpaeTARAAPEI-5, Unigene20573\_TAEpaeTARAAPEI-5, Unigene20670\_TAEpaeTARAAPEI-5, Unigene2080\_TAEpaeTARAAPEI-5, Unigene20888\_TAEpaeTARAAPEI-5, Unigene21021\_TAEpaeTARAAPEI-5, Unigene21539\_TAEpaeTARAAPEI-5, Unigene21613\_TAEpaeTARAAPEI-5, Unigene21641\_TAEpaeTARAAPEI-5, Unigene21697\_TAEpaeTARAAPEI-5, Unigene21768\_TAEpaeTARAAPEI-5, Unigene21886\_TAEpaeTARAAPEI-5, Unigene21944\_TAEpaeTARAAPEI-5, Unigene22314\_TAEpaeTARAAPEI-5, Unigene2245\_TAEpaeTARAAPEI-5, Unigene22555\_TAEpaeTARAAPEI-5, Unigene22576\_TAEpaeTARAAPEI-5, Unigene22738\_TAEpaeTARAAPEI-5, Unigene22800\_TAEpaeTARAAPEI-5, Unigene22879\_TAEpaeTARAAPEI-5, Unigene22946\_TAEpaeTARAAPEI-5, Unigene23057\_TAEpaeTARAAPEI-5, Unigene23156\_TAEpaeTARAAPEI-5, Unigene23209\_TAEpaeTARAAPEI-5, Unigene23276\_TAEpaeTARAAPEI-5, Unigene23337\_TAEpaeTARAAPEI-5, Unigene23489\_TAEpaeTARAAPEI-5, Unigene23775\_TAEpaeTARAAPEI-5, Unigene24289\_TAEpaeTARAAPEI-5, Unigene24291\_TAEpaeTARAAPEI-5, Unigene24367\_TAEpaeTARAAPEI-5, Unigene24369\_TAEpaeTARAAPEI-5, Unigene24553\_TAEpaeTARAAPEI-5, Unigene24610\_TAEpaeTARAAPEI-5, Unigene24630\_TAEpaeTARAAPEI-5, Unigene24678\_TAEpaeTARAAPEI-5, Unigene24785\_TAEpaeTARAAPEI-5, Unigene24825\_TAEpaeTARAAPEI-5, Unigene24964\_TAEpaeTARAAPEI-5, Unigene25039\_TAEpaeTARAAPEI-5, Unigene25399\_TAEpaeTARAAPEI-5, Unigene25550\_TAEpaeTARAAPEI-5, Unigene25681\_TAEpaeTARAAPEI-5, Unigene25974\_TAEpaeTARAAPEI-5, Unigene26027\_TAEpaeTARAAPEI-5, Unigene26181\_TAEpaeTARAAPEI-5, Unigene2679\_TAEpaeTARAAPEI-5, Unigene26882\_TAEpaeTARAAPEI-5, Unigene27159\_TAEpaeTARAAPEI-5, Unigene27192\_TAEpaeTARAAPEI-5, Unigene27344\_TAEpaeTARAAPEI-5, Unigene27672\_TAEpaeTARAAPEI-5, Unigene27715\_TAEpaeTARAAPEI-5, Unigene27777\_TAEpaeTARAAPEI-5, Unigene27875\_TAEpaeTARAAPEI-5, Unigene28078\_TAEpaeTARAAPEI-5, Unigene2818\_TAEpaeTARAAPEI-5, Unigene28501\_TAEpaeTARAAPEI-5, Unigene28505\_TAEpaeTARAAPEI-5, Unigene28722\_TAEpaeTARAAPEI-5, Unigene28793\_TAEpaeTARAAPEI-5, Unigene28879\_TAEpaeTARAAPEI-5, Unigene28905\_TAEpaeTARAAPEI-5, Unigene2892\_TAEpaeTARAAPEI-5, Unigene29274\_TAEpaeTARAAPEI-5, Unigene2950\_TAEpaeTARAAPEI-5, Unigene29638\_TAEpaeTARAAPEI-5, Unigene29639\_TAEpaeTARAAPEI-5, Unigene29679\_TAEpaeTARAAPEI-5, Unigene2982\_TAEpaeTARAAPEI-5, Unigene29862\_TAEpaeTARAAPEI-5, Unigene30058\_TAEpaeTARAAPEI-5, Unigene30109\_TAEpaeTARAAPEI-5, Unigene30234\_TAEpaeTARAAPEI-5, Unigene3037\_TAEpaeTARAAPEI-5, Unigene30400\_TAEpaeTARAAPEI-5, Unigene30423\_TAEpaeTARAAPEI-5, Unigene3043\_TAEpaeTARAAPEI-5, Unigene30463\_TAEpaeTARAAPEI-5, Unigene30911\_TAEpaeTARAAPEI-5, Unigene31512\_TAEpaeTARAAPEI-5, Unigene31826\_TAEpaeTARAAPEI-5, Unigene3198\_TAEpaeTARAAPEI-5, Unigene32079\_TAEpaeTARAAPEI-5, Unigene32199\_TAEpaeTARAAPEI-5, Unigene32309\_TAEpaeTARAAPEI-5, Unigene32667\_TAEpaeTARAAPEI-5, Unigene33120\_TAEpaeTARAAPEI-5, Unigene33219\_TAEpaeTARAAPEI-5, Unigene33265\_TAEpaeTARAAPEI-5, Unigene33317\_TAEpaeTARAAPEI-5, Unigene33564\_TAEpaeTARAAPEI-5, Unigene33770\_TAEpaeTARAAPEI-5, Unigene33930\_TAEpaeTARAAPEI-5, Unigene34112\_TAEpaeTARAAPEI-5, Unigene34159\_TAEpaeTARAAPEI-5, Unigene342\_TAEpaeTARAAPEI-5, Unigene34301\_TAEpaeTARAAPEI-5, Unigene34447\_TAEpaeTARAAPEI-5, Unigene344\_TAEpaeTARAAPEI-5, Unigene346\_TAEpaeTARAAPEI-5, Unigene35224\_TAEpaeTARAAPEI-5, Unigene35420\_TAEpaeTARAAPEI-5, Unigene35747\_TAEpaeTARAAPEI-5, Unigene36057\_TAEpaeTARAAPEI-5, Unigene36154\_TAEpaeTARAAPEI-5, Unigene36198\_TAEpaeTARAAPEI-5, Unigene36242\_TAEpaeTARAAPEI-5, Unigene36314\_TAEpaeTARAAPEI-5, Unigene36377\_TAEpaeTARAAPEI-5, Unigene36435\_TAEpaeTARAAPEI-5, Unigene36695\_TAEpaeTARAAPEI-5, Unigene36933\_TAEpaeTARAAPEI-5, Unigene36936\_TAEpaeTARAAPEI-5, Unigene37157\_TAEpaeTARAAPEI-5, Unigene37192\_TAEpaeTARAAPEI-5, Unigene37193\_TAEpaeTARAAPEI-5, Unigene37366\_TAEpaeTARAAPEI-5, Unigene37435\_TAEpaeTARAAPEI-5, Unigene37459\_TAEpaeTARAAPEI-5, Unigene37663\_TAEpaeTARAAPEI-5, Unigene37752\_TAEpaeTARAAPEI-5, Unigene37833\_TAEpaeTARAAPEI-5, Unigene38178\_TAEpaeTARAAPEI-5, Unigene38217\_TAEpaeTARAAPEI-5, Unigene38349\_TAEpaeTARAAPEI-5, Unigene38492\_TAEpaeTARAAPEI-5, Unigene38649\_TAEpaeTARAAPEI-5, Unigene38713\_TAEpaeTARAAPEI-5, Unigene38732\_TAEpaeTARAAPEI-5, Unigene3881\_TAEpaeTARAAPEI-5, Unigene39019\_TAEpaeTARAAPEI-5, Unigene39092\_TAEpaeTARAAPEI-5, Unigene39096\_TAEpaeTARAAPEI-5, Unigene39269\_TAEpaeTARAAPEI-5, Unigene39427\_TAEpaeTARAAPEI-5, Unigene39517\_TAEpaeTARAAPEI-5, Unigene39598\_TAEpaeTARAAPEI-5, Unigene39725\_TAEpaeTARAAPEI-5, Unigene3985\_TAEpaeTARAAPEI-5, Unigene39918\_TAEpaeTARAAPEI-5, Unigene39949\_TAEpaeTARAAPEI-5, Unigene4002\_TAEpaeTARAAPEI-5, Unigene40162\_TAEpaeTARAAPEI-5, Unigene40262\_TAEpaeTARAAPEI-5, Unigene40268\_TAEpaeTARAAPEI-5, Unigene40389\_TAEpaeTARAAPEI-5, Unigene4040\_TAEpaeTARAAPEI-5, Unigene40507\_TAEpaeTARAAPEI-5, Unigene40711\_TAEpaeTARAAPEI-5, Unigene40727\_TAEpaeTARAAPEI-5, Unigene40746\_TAEpaeTARAAPEI-5, Unigene4078\_TAEpaeTARAAPEI-5, Unigene40962\_TAEpaeTARAAPEI-5, Unigene40979\_TAEpaeTARAAPEI-5, Unigene41098\_TAEpaeTARAAPEI-5, Unigene41149\_TAEpaeTARAAPEI-5, Unigene41365\_TAEpaeTARAAPEI-5, Unigene41475\_TAEpaeTARAAPEI-5, Unigene41653\_TAEpaeTARAAPEI-5, Unigene41746\_TAEpaeTARAAPEI-5, Unigene41776\_TAEpaeTARAAPEI-5, Unigene41845\_TAEpaeTARAAPEI-5, Unigene41902\_TAEpaeTARAAPEI-5, Unigene42017\_TAEpaeTARAAPEI-5, Unigene42020\_TAEpaeTARAAPEI-5, Unigene42060\_TAEpaeTARAAPEI-5, Unigene42192\_TAEpaeTARAAPEI-5, Unigene42313\_TAEpaeTARAAPEI-5, Unigene42358\_TAEpaeTARAAPEI-5, Unigene42525\_TAEpaeTARAAPEI-5, Unigene42573\_TAEpaeTARAAPEI-5, Unigene42619\_TAEpaeTARAAPEI-5, Unigene42667\_TAEpaeTARAAPEI-5, Unigene42918\_TAEpaeTARAAPEI-5, Unigene42\_TAEpaeTARAAPEI-5, Unigene43485\_TAEpaeTARAAPEI-5, Unigene43491\_TAEpaeTARAAPEI-5, Unigene43563\_TAEpaeTARAAPEI-5, Unigene43918\_TAEpaeTARAAPEI-5, Unigene43951\_TAEpaeTARAAPEI-5, Unigene44118\_TAEpaeTARAAPEI-5, Unigene44575\_TAEpaeTARAAPEI-5, Unigene44652\_TAEpaeTARAAPEI-5, Unigene44678\_TAEpaeTARAAPEI-5, Unigene44726\_TAEpaeTARAAPEI-5, Unigene44809\_TAEpaeTARAAPEI-5, Unigene44911\_TAEpaeTARAAPEI-5, Unigene45281\_TAEpaeTARAAPEI-5, Unigene4539\_TAEpaeTARAAPEI-5, Unigene45455\_TAEpaeTARAAPEI-5, Unigene45520\_TAEpaeTARAAPEI-5, Unigene45552\_TAEpaeTARAAPEI-5, Unigene45562\_TAEpaeTARAAPEI-5, Unigene45716\_TAEpaeTARAAPEI-5, Unigene45811\_TAEpaeTARAAPEI-5, Unigene45867\_TAEpaeTARAAPEI-5, Unigene45939\_TAEpaeTARAAPEI-5, Unigene46052\_TAEpaeTARAAPEI-5, Unigene46245\_TAEpaeTARAAPEI-5, Unigene46343\_TAEpaeTARAAPEI-5, Unigene46468\_TAEpaeTARAAPEI-5, Unigene46498\_TAEpaeTARAAPEI-5, Unigene46507\_TAEpaeTARAAPEI-5, Unigene46600\_TAEpaeTARAAPEI-5, Unigene46722\_TAEpaeTARAAPEI-5, Unigene46735\_TAEpaeTARAAPEI-5, Unigene46756\_TAEpaeTARAAPEI-5, Unigene47002\_TAEpaeTARAAPEI-5, Unigene47105\_TAEpaeTARAAPEI-5, Unigene47255\_TAEpaeTARAAPEI-5, Unigene47623\_TAEpaeTARAAPEI-5, Unigene47653\_TAEpaeTARAAPEI-5, Unigene47832\_TAEpaeTARAAPEI-5, Unigene47984\_TAEpaeTARAAPEI-5, Unigene48127\_TAEpaeTARAAPEI-5, Unigene481\_TAEpaeTARAAPEI-5, Unigene48227\_TAEpaeTARAAPEI-5, Unigene48450\_TAEpaeTARAAPEI-5, Unigene48466\_TAEpaeTARAAPEI-5, Unigene48702\_TAEpaeTARAAPEI-5, Unigene48741\_TAEpaeTARAAPEI-5, Unigene48761\_TAEpaeTARAAPEI-5, Unigene48778\_TAEpaeTARAAPEI-5, Unigene48976\_TAEpaeTARAAPEI-5, Unigene49009\_TAEpaeTARAAPEI-5, Unigene49206\_TAEpaeTARAAPEI-5, Unigene49384\_TAEpaeTARAAPEI-5, Unigene49780\_TAEpaeTARAAPEI-5, Unigene49887\_TAEpaeTARAAPEI-5, Unigene50030\_TAEpaeTARAAPEI-5, Unigene50532\_TAEpaeTARAAPEI-5, Unigene50754\_TAEpaeTARAAPEI-5, Unigene50799\_TAEpaeTARAAPEI-5, Unigene50802\_TAEpaeTARAAPEI-5, Unigene50869\_TAEpaeTARAAPEI-5, Unigene50926\_TAEpaeTARAAPEI-5, Unigene51273\_TAEpaeTARAAPEI-5, Unigene51295\_TAEpaeTARAAPEI-5, Unigene51411\_TAEpaeTARAAPEI-5, Unigene51478\_TAEpaeTARAAPEI-5, Unigene51549\_TAEpaeTARAAPEI-5, Unigene51562\_TAEpaeTARAAPEI-5, Unigene51671\_TAEpaeTARAAPEI-5, Unigene51838\_TAEpaeTARAAPEI-5, Unigene51906\_TAEpaeTARAAPEI-5, Unigene51926\_TAEpaeTARAAPEI-5, Unigene52056\_TAEpaeTARAAPEI-5, Unigene52341\_TAEpaeTARAAPEI-5, Unigene52347\_TAEpaeTARAAPEI-5, Unigene52374\_TAEpaeTARAAPEI-5, Unigene52686\_TAEpaeTARAAPEI-5, Unigene52772\_TAEpaeTARAAPEI-5, Unigene52822\_TAEpaeTARAAPEI-5, Unigene52842\_TAEpaeTARAAPEI-5, Unigene52847\_TAEpaeTARAAPEI-5, Unigene52851\_TAEpaeTARAAPEI-5, Unigene53007\_TAEpaeTARAAPEI-5, Unigene53009\_TAEpaeTARAAPEI-5, Unigene53241\_TAEpaeTARAAPEI-5, Unigene53254\_TAEpaeTARAAPEI-5, Unigene53257\_TAEpaeTARAAPEI-5, Unigene53348\_TAEpaeTARAAPEI-5, Unigene53369\_TAEpaeTARAAPEI-5, Unigene53391\_TAEpaeTARAAPEI-5, Unigene53611\_TAEpaeTARAAPEI-5, Unigene53762\_TAEpaeTARAAPEI-5, Unigene53851\_TAEpaeTARAAPEI-5, Unigene53881\_TAEpaeTARAAPEI-5, Unigene54038\_TAEpaeTARAAPEI-5, Unigene54046\_TAEpaeTARAAPEI-5, Unigene54055\_TAEpaeTARAAPEI-5, Unigene54083\_TAEpaeTARAAPEI-5, Unigene54110\_TAEpaeTARAAPEI-5, Unigene54114\_TAEpaeTARAAPEI-5, Unigene54159\_TAEpaeTARAAPEI-5, Unigene54374\_TAEpaeTARAAPEI-5, Unigene54422\_TAEpaeTARAAPEI-5, Unigene54558\_TAEpaeTARAAPEI-5, Unigene5463\_TAEpaeTARAAPEI-5, Unigene54723\_TAEpaeTARAAPEI-5, Unigene54831\_TAEpaeTARAAPEI-5, Unigene54985\_TAEpaeTARAAPEI-5, Unigene55003\_TAEpaeTARAAPEI-5, Unigene55045\_TAEpaeTARAAPEI-5, Unigene55058\_TAEpaeTARAAPEI-5, Unigene55177\_TAEpaeTARAAPEI-5, Unigene5522\_TAEpaeTARAAPEI-5, Unigene55239\_TAEpaeTARAAPEI-5, Unigene55243\_TAEpaeTARAAPEI-5, Unigene55261\_TAEpaeTARAAPEI-5, Unigene55288\_TAEpaeTARAAPEI-5, Unigene55291\_TAEpaeTARAAPEI-5, Unigene55306\_TAEpaeTARAAPEI-5, Unigene55366\_TAEpaeTARAAPEI-5, Unigene55463\_TAEpaeTARAAPEI-5, Unigene55522\_TAEpaeTARAAPEI-5, Unigene55594\_TAEpaeTARAAPEI-5, Unigene5562\_TAEpaeTARAAPEI-5, Unigene55765\_TAEpaeTARAAPEI-5, Unigene55820\_TAEpaeTARAAPEI-5, Unigene55956\_TAEpaeTARAAPEI-5, Unigene55984\_TAEpaeTARAAPEI-5, Unigene56007\_TAEpaeTARAAPEI-5, Unigene56026\_TAEpaeTARAAPEI-5, Unigene56110\_TAEpaeTARAAPEI-5, Unigene56145\_TAEpaeTARAAPEI-5, Unigene56204\_TAEpaeTARAAPEI-5, Unigene56314\_TAEpaeTARAAPEI-5, Unigene56344\_TAEpaeTARAAPEI-5, Unigene56369\_TAEpaeTARAAPEI-5, Unigene56517\_TAEpaeTARAAPEI-5, Unigene56519\_TAEpaeTARAAPEI-5, Unigene56553\_TAEpaeTARAAPEI-5, Unigene56754\_TAEpaeTARAAPEI-5, Unigene56921\_TAEpaeTARAAPEI-5, Unigene5701\_TAEpaeTARAAPEI-5, Unigene57148\_TAEpaeTARAAPEI-5, Unigene57158\_TAEpaeTARAAPEI-5, Unigene57174\_TAEpaeTARAAPEI-5, Unigene57203\_TAEpaeTARAAPEI-5, Unigene57206\_TAEpaeTARAAPEI-5, Unigene57282\_TAEpaeTARAAPEI-5, Unigene57284\_TAEpaeTARAAPEI-5, Unigene57338\_TAEpaeTARAAPEI-5, Unigene57339\_TAEpaeTARAAPEI-5, Unigene57340\_TAEpaeTARAAPEI-5, Unigene57459\_TAEpaeTARAAPEI-5, Unigene57470\_TAEpaeTARAAPEI-5, Unigene57546\_TAEpaeTARAAPEI-5, Unigene57588\_TAEpaeTARAAPEI-5, Unigene57740\_TAEpaeTARAAPEI-5, Unigene57755\_TAEpaeTARAAPEI-5, Unigene57953\_TAEpaeTARAAPEI-5, Unigene5798\_TAEpaeTARAAPEI-5, Unigene58093\_TAEpaeTARAAPEI-5, Unigene58179\_TAEpaeTARAAPEI-5, Unigene58363\_TAEpaeTARAAPEI-5, Unigene58380\_TAEpaeTARAAPEI-5, Unigene58459\_TAEpaeTARAAPEI-5, Unigene58484\_TAEpaeTARAAPEI-5, Unigene58488\_TAEpaeTARAAPEI-5, Unigene58505\_TAEpaeTARAAPEI-5, Unigene58512\_TAEpaeTARAAPEI-5, Unigene58592\_TAEpaeTARAAPEI-5, Unigene58595\_TAEpaeTARAAPEI-5, Unigene58647\_TAEpaeTARAAPEI-5, Unigene58748\_TAEpaeTARAAPEI-5, Unigene58879\_TAEpaeTARAAPEI-5, Unigene58967\_TAEpaeTARAAPEI-5, Unigene58981\_TAEpaeTARAAPEI-5, Unigene59054\_TAEpaeTARAAPEI-5, Unigene59204\_TAEpaeTARAAPEI-5, Unigene59256\_TAEpaeTARAAPEI-5, Unigene5926\_TAEpaeTARAAPEI-5, Unigene59291\_TAEpaeTARAAPEI-5, Unigene59392\_TAEpaeTARAAPEI-5, Unigene59605\_TAEpaeTARAAPEI-5, Unigene59614\_TAEpaeTARAAPEI-5, Unigene59622\_TAEpaeTARAAPEI-5, Unigene59697\_TAEpaeTARAAPEI-5, Unigene59772\_TAEpaeTARAAPEI-5, Unigene59868\_TAEpaeTARAAPEI-5, Unigene59963\_TAEpaeTARAAPEI-5, Unigene60000\_TAEpaeTARAAPEI-5, Unigene60009\_TAEpaeTARAAPEI-5, Unigene60041\_TAEpaeTARAAPEI-5, Unigene60131\_TAEpaeTARAAPEI-5, Unigene60169\_TAEpaeTARAAPEI-5, Unigene60248\_TAEpaeTARAAPEI-5, Unigene60319\_TAEpaeTARAAPEI-5, Unigene60348\_TAEpaeTARAAPEI-5, Unigene6037\_TAEpaeTARAAPEI-5, Unigene60495\_TAEpaeTARAAPEI-5, Unigene60552\_TAEpaeTARAAPEI-5, Unigene60594\_TAEpaeTARAAPEI-5, Unigene60619\_TAEpaeTARAAPEI-5, Unigene60666\_TAEpaeTARAAPEI-5, Unigene60748\_TAEpaeTARAAPEI-5, Unigene60788\_TAEpaeTARAAPEI-5, Unigene60924\_TAEpaeTARAAPEI-5, Unigene60934\_TAEpaeTARAAPEI-5, Unigene61070\_TAEpaeTARAAPEI-5, Unigene611\_TAEpaeTARAAPEI-5, Unigene61524\_TAEpaeTARAAPEI-5, Unigene61537\_TAEpaeTARAAPEI-5, Unigene61565\_TAEpaeTARAAPEI-5, Unigene61605\_TAEpaeTARAAPEI-5, Unigene61626\_TAEpaeTARAAPEI-5, Unigene61768\_TAEpaeTARAAPEI-5, Unigene61818\_TAEpaeTARAAPEI-5, Unigene61885\_TAEpaeTARAAPEI-5, Unigene61910\_TAEpaeTARAAPEI-5, Unigene61916\_TAEpaeTARAAPEI-5, Unigene61942\_TAEpaeTARAAPEI-5, Unigene61946\_TAEpaeTARAAPEI-5, Unigene61986\_TAEpaeTARAAPEI-5, Unigene62103\_TAEpaeTARAAPEI-5, Unigene62147\_TAEpaeTARAAPEI-5, Unigene62177\_TAEpaeTARAAPEI-5, Unigene62180\_TAEpaeTARAAPEI-5, Unigene62204\_TAEpaeTARAAPEI-5, Unigene62321\_TAEpaeTARAAPEI-5, Unigene62510\_TAEpaeTARAAPEI-5, Unigene62518\_TAEpaeTARAAPEI-5, Unigene62566\_TAEpaeTARAAPEI-5, Unigene62708\_TAEpaeTARAAPEI-5, Unigene62712\_TAEpaeTARAAPEI-5, Unigene62855\_TAEpaeTARAAPEI-5, Unigene62917\_TAEpaeTARAAPEI-5, Unigene62992\_TAEpaeTARAAPEI-5, Unigene63044\_TAEpaeTARAAPEI-5, Unigene63093\_TAEpaeTARAAPEI-5, Unigene63156\_TAEpaeTARAAPEI-5, Unigene63211\_TAEpaeTARAAPEI-5, Unigene63453\_TAEpaeTARAAPEI-5, Unigene63464\_TAEpaeTARAAPEI-5, Unigene63517\_TAEpaeTARAAPEI-5, Unigene63610\_TAEpaeTARAAPEI-5, Unigene63639\_TAEpaeTARAAPEI-5, Unigene63654\_TAEpaeTARAAPEI-5, Unigene63656\_TAEpaeTARAAPEI-5, Unigene63703\_TAEpaeTARAAPEI-5, Unigene63758\_TAEpaeTARAAPEI-5, Unigene63821\_TAEpaeTARAAPEI-5, Unigene63883\_TAEpaeTARAAPEI-5, Unigene63962\_TAEpaeTARAAPEI-5, Unigene64097\_TAEpaeTARAAPEI-5, Unigene64200\_TAEpaeTARAAPEI-5, Unigene64201\_TAEpaeTARAAPEI-5, Unigene64298\_TAEpaeTARAAPEI-5, Unigene64477\_TAEpaeTARAAPEI-5, Unigene64512\_TAEpaeTARAAPEI-5, Unigene64730\_TAEpaeTARAAPEI-5, Unigene64742\_TAEpaeTARAAPEI-5, Unigene64853\_TAEpaeTARAAPEI-5, Unigene64906\_TAEpaeTARAAPEI-5, Unigene64914\_TAEpaeTARAAPEI-5, Unigene64915\_TAEpaeTARAAPEI-5, Unigene64916\_TAEpaeTARAAPEI-5, Unigene64921\_TAEpaeTARAAPEI-5, Unigene64924\_TAEpaeTARAAPEI-5, Unigene65024\_TAEpaeTARAAPEI-5, Unigene65089\_TAEpaeTARAAPEI-5, Unigene65145\_TAEpaeTARAAPEI-5, Unigene65174\_TAEpaeTARAAPEI-5, Unigene65182\_TAEpaeTARAAPEI-5, Unigene65186\_TAEpaeTARAAPEI-5, Unigene65256\_TAEpaeTARAAPEI-5, Unigene65535\_TAEpaeTARAAPEI-5, Unigene65536\_TAEpaeTARAAPEI-5, Unigene65557\_TAEpaeTARAAPEI-5, Unigene65792\_TAEpaeTARAAPEI-5, Unigene65814\_TAEpaeTARAAPEI-5, Unigene65922\_TAEpaeTARAAPEI-5, Unigene65985\_TAEpaeTARAAPEI-5, Unigene66241\_TAEpaeTARAAPEI-5, Unigene66252\_TAEpaeTARAAPEI-5, Unigene66314\_TAEpaeTARAAPEI-5, Unigene66348\_TAEpaeTARAAPEI-5, Unigene66520\_TAEpaeTARAAPEI-5, Unigene66545\_TAEpaeTARAAPEI-5, Unigene66666\_TAEpaeTARAAPEI-5, Unigene66725\_TAEpaeTARAAPEI-5, Unigene6672\_TAEpaeTARAAPEI-5, Unigene66799\_TAEpaeTARAAPEI-5, Unigene6683\_TAEpaeTARAAPEI-5, Unigene66905\_TAEpaeTARAAPEI-5, Unigene6694\_TAEpaeTARAAPEI-5, Unigene66954\_TAEpaeTARAAPEI-5, Unigene66977\_TAEpaeTARAAPEI-5, Unigene66980\_TAEpaeTARAAPEI-5, Unigene6700\_TAEpaeTARAAPEI-5, Unigene67143\_TAEpaeTARAAPEI-5, Unigene67152\_TAEpaeTARAAPEI-5, Unigene67219\_TAEpaeTARAAPEI-5, Unigene67220\_TAEpaeTARAAPEI-5, Unigene6728\_TAEpaeTARAAPEI-5, Unigene67316\_TAEpaeTARAAPEI-5, Unigene67394\_TAEpaeTARAAPEI-5, Unigene67414\_TAEpaeTARAAPEI-5, Unigene67489\_TAEpaeTARAAPEI-5, Unigene67516\_TAEpaeTARAAPEI-5, Unigene67576\_TAEpaeTARAAPEI-5, Unigene6759\_TAEpaeTARAAPEI-5, Unigene67701\_TAEpaeTARAAPEI-5, Unigene67725\_TAEpaeTARAAPEI-5, Unigene67783\_TAEpaeTARAAPEI-5, Unigene67805\_TAEpaeTARAAPEI-5, Unigene67811\_TAEpaeTARAAPEI-5, Unigene67898\_TAEpaeTARAAPEI-5, Unigene67994\_TAEpaeTARAAPEI-5, Unigene68039\_TAEpaeTARAAPEI-5, Unigene68043\_TAEpaeTARAAPEI-5, Unigene68046\_TAEpaeTARAAPEI-5, Unigene68058\_TAEpaeTARAAPEI-5, Unigene68105\_TAEpaeTARAAPEI-5, Unigene68147\_TAEpaeTARAAPEI-5, Unigene6822\_TAEpaeTARAAPEI-5, Unigene68238\_TAEpaeTARAAPEI-5, Unigene68282\_TAEpaeTARAAPEI-5, Unigene6833\_TAEpaeTARAAPEI-5, Unigene68382\_TAEpaeTARAAPEI-5, Unigene68476\_TAEpaeTARAAPEI-5, Unigene68512\_TAEpaeTARAAPEI-5, Unigene68516\_TAEpaeTARAAPEI-5, Unigene68518\_TAEpaeTARAAPEI-5, Unigene68529\_TAEpaeTARAAPEI-5, Unigene68536\_TAEpaeTARAAPEI-5, Unigene68555\_TAEpaeTARAAPEI-5, Unigene68605\_TAEpaeTARAAPEI-5, Unigene68622\_TAEpaeTARAAPEI-5, Unigene68679\_TAEpaeTARAAPEI-5, Unigene68749\_TAEpaeTARAAPEI-5, Unigene68769\_TAEpaeTARAAPEI-5, Unigene68780\_TAEpaeTARAAPEI-5, Unigene68887\_TAEpaeTARAAPEI-5, Unigene68920\_TAEpaeTARAAPEI-5, Unigene68926\_TAEpaeTARAAPEI-5, Unigene6898\_TAEpaeTARAAPEI-5, Unigene69063\_TAEpaeTARAAPEI-5, Unigene6914\_TAEpaeTARAAPEI-5, Unigene69160\_TAEpaeTARAAPEI-5, Unigene6917\_TAEpaeTARAAPEI-5, Unigene69237\_TAEpaeTARAAPEI-5, Unigene69250\_TAEpaeTARAAPEI-5, Unigene69264\_TAEpaeTARAAPEI-5, Unigene69283\_TAEpaeTARAAPEI-5, Unigene69527\_TAEpaeTARAAPEI-5, Unigene69555\_TAEpaeTARAAPEI-5, Unigene69630\_TAEpaeTARAAPEI-5, Unigene69633\_TAEpaeTARAAPEI-5, Unigene69654\_TAEpaeTARAAPEI-5, Unigene6985\_TAEpaeTARAAPEI-5, Unigene69862\_TAEpaeTARAAPEI-5, Unigene69890\_TAEpaeTARAAPEI-5, Unigene70007\_TAEpaeTARAAPEI-5, Unigene70010\_TAEpaeTARAAPEI-5, Unigene70026\_TAEpaeTARAAPEI-5, Unigene70111\_TAEpaeTARAAPEI-5, Unigene70117\_TAEpaeTARAAPEI-5, Unigene70133\_TAEpaeTARAAPEI-5, Unigene70150\_TAEpaeTARAAPEI-5, Unigene70239\_TAEpaeTARAAPEI-5, Unigene70380\_TAEpaeTARAAPEI-5, Unigene70396\_TAEpaeTARAAPEI-5, Unigene704\_TAEpaeTARAAPEI-5, Unigene70604\_TAEpaeTARAAPEI-5, Unigene70709\_TAEpaeTARAAPEI-5, Unigene70712\_TAEpaeTARAAPEI-5, Unigene70778\_TAEpaeTARAAPEI-5, Unigene70813\_TAEpaeTARAAPEI-5, Unigene70830\_TAEpaeTARAAPEI-5, Unigene70831\_TAEpaeTARAAPEI-5, Unigene70874\_TAEpaeTARAAPEI-5, Unigene70878\_TAEpaeTARAAPEI-5, Unigene71012\_TAEpaeTARAAPEI-5, Unigene71030\_TAEpaeTARAAPEI-5, Unigene71056\_TAEpaeTARAAPEI-5, Unigene71077\_TAEpaeTARAAPEI-5, Unigene71119\_TAEpaeTARAAPEI-5, Unigene71186\_TAEpaeTARAAPEI-5, Unigene71315\_TAEpaeTARAAPEI-5, Unigene71325\_TAEpaeTARAAPEI-5, Unigene71387\_TAEpaeTARAAPEI-5, Unigene71479\_TAEpaeTARAAPEI-5, Unigene71490\_TAEpaeTARAAPEI-5, Unigene71493\_TAEpaeTARAAPEI-5, Unigene71564\_TAEpaeTARAAPEI-5, Unigene71612\_TAEpaeTARAAPEI-5, Unigene71625\_TAEpaeTARAAPEI-5, Unigene71643\_TAEpaeTARAAPEI-5, Unigene71660\_TAEpaeTARAAPEI-5, Unigene71706\_TAEpaeTARAAPEI-5, Unigene71727\_TAEpaeTARAAPEI-5, Unigene71754\_TAEpaeTARAAPEI-5, Unigene71786\_TAEpaeTARAAPEI-5, Unigene71791\_TAEpaeTARAAPEI-5, Unigene71805\_TAEpaeTARAAPEI-5, Unigene71838\_TAEpaeTARAAPEI-5, Unigene71845\_TAEpaeTARAAPEI-5, Unigene71858\_TAEpaeTARAAPEI-5, Unigene71940\_TAEpaeTARAAPEI-5, Unigene71974\_TAEpaeTARAAPEI-5, Unigene71999\_TAEpaeTARAAPEI-5, Unigene72049\_TAEpaeTARAAPEI-5, Unigene72056\_TAEpaeTARAAPEI-5, Unigene72080\_TAEpaeTARAAPEI-5, Unigene72103\_TAEpaeTARAAPEI-5, Unigene72112\_TAEpaeTARAAPEI-5, Unigene72139\_TAEpaeTARAAPEI-5, Unigene72168\_TAEpaeTARAAPEI-5, Unigene72176\_TAEpaeTARAAPEI-5, Unigene72197\_TAEpaeTARAAPEI-5, Unigene72212\_TAEpaeTARAAPEI-5, Unigene7227\_TAEpaeTARAAPEI-5, Unigene72342\_TAEpaeTARAAPEI-5, Unigene72360\_TAEpaeTARAAPEI-5, Unigene72424\_TAEpaeTARAAPEI-5, Unigene72489\_TAEpaeTARAAPEI-5, Unigene72516\_TAEpaeTARAAPEI-5, Unigene72525\_TAEpaeTARAAPEI-5, Unigene72560\_TAEpaeTARAAPEI-5, Unigene72564\_TAEpaeTARAAPEI-5, Unigene72628\_TAEpaeTARAAPEI-5, Unigene72636\_TAEpaeTARAAPEI-5, Unigene72653\_TAEpaeTARAAPEI-5, Unigene72673\_TAEpaeTARAAPEI-5, Unigene72695\_TAEpaeTARAAPEI-5, Unigene72774\_TAEpaeTARAAPEI-5, Unigene72782\_TAEpaeTARAAPEI-5, Unigene72793\_TAEpaeTARAAPEI-5, Unigene72899\_TAEpaeTARAAPEI-5, Unigene72932\_TAEpaeTARAAPEI-5, Unigene72940\_TAEpaeTARAAPEI-5, Unigene7304\_TAEpaeTARAAPEI-5, Unigene7341\_TAEpaeTARAAPEI-5, Unigene7354\_TAEpaeTARAAPEI-5, Unigene7375\_TAEpaeTARAAPEI-5, Unigene7738\_TAEpaeTARAAPEI-5, Unigene8269\_TAEpaeTARAAPEI-5, Unigene8274\_TAEpaeTARAAPEI-5, Unigene8297\_TAEpaeTARAAPEI-5, Unigene8395\_TAEpaeTARAAPEI-5, Unigene8409\_TAEpaeTARAAPEI-5, Unigene8414\_TAEpaeTARAAPEI-5, Unigene8481\_TAEpaeTARAAPEI-5, Unigene8571\_TAEpaeTARAAPEI-5, Unigene8618\_TAEpaeTARAAPEI-5, Unigene8633\_TAEpaeTARAAPEI-5, Unigene8836\_TAEpaeTARAAPEI-5, Unigene8843\_TAEpaeTARAAPEI-5, Unigene8904\_TAEpaeTARAAPEI-5, Unigene9164\_TAEpaeTARAAPEI-5, Unigene9167\_TAEpaeTARAAPEI-5, Unigene9233\_TAEpaeTARAAPEI-5, Unigene92\_TAEpaeTARAAPEI-5, Unigene9341\_TAEpaeTARAAPEI-5, Unigene93\_TAEpaeTARAAPEI-5, Unigene9423\_TAEpaeTARAAPEI-5, Unigene9615\_TAEpaeTARAAPEI-5, Unigene9670\_TAEpaeTARAAPEI-5, Unigene9727\_TAEpaeTARAAPEI-5, Unigene9749\_TAEpaeTARAAPEI-5, Unigene9778\_TAEpaeTARAAPEI-5, Unigene9802\_TAEpaeTARAAPEI-5, Unigene9894\_TAEpaeTARAAPEI-5, Unigene9909\_TAEpaeTARAAPEI-5 |
| 3 | Regulation of actin cytoskeleton | Unigene10126\_TAEpaeTARAAPEI-5, Unigene10142\_TAEpaeTARAAPEI-5, Unigene10250\_TAEpaeTARAAPEI-5, Unigene1035\_TAEpaeTARAAPEI-5, Unigene10544\_TAEpaeTARAAPEI-5, Unigene10581\_TAEpaeTARAAPEI-5, Unigene10616\_TAEpaeTARAAPEI-5, Unigene10634\_TAEpaeTARAAPEI-5, Unigene10658\_TAEpaeTARAAPEI-5, Unigene10720\_TAEpaeTARAAPEI-5, Unigene10835\_TAEpaeTARAAPEI-5, Unigene10848\_TAEpaeTARAAPEI-5, Unigene10859\_TAEpaeTARAAPEI-5, Unigene10863\_TAEpaeTARAAPEI-5, Unigene1087\_TAEpaeTARAAPEI-5, Unigene11069\_TAEpaeTARAAPEI-5, Unigene11238\_TAEpaeTARAAPEI-5, Unigene11268\_TAEpaeTARAAPEI-5, Unigene11280\_TAEpaeTARAAPEI-5, Unigene11291\_TAEpaeTARAAPEI-5, Unigene11479\_TAEpaeTARAAPEI-5, Unigene11534\_TAEpaeTARAAPEI-5, Unigene11551\_TAEpaeTARAAPEI-5, Unigene11772\_TAEpaeTARAAPEI-5, Unigene12059\_TAEpaeTARAAPEI-5, Unigene12116\_TAEpaeTARAAPEI-5, Unigene12145\_TAEpaeTARAAPEI-5, Unigene12159\_TAEpaeTARAAPEI-5, Unigene12167\_TAEpaeTARAAPEI-5, Unigene12401\_TAEpaeTARAAPEI-5, Unigene12647\_TAEpaeTARAAPEI-5, Unigene12878\_TAEpaeTARAAPEI-5, Unigene12921\_TAEpaeTARAAPEI-5, Unigene12947\_TAEpaeTARAAPEI-5, Unigene12955\_TAEpaeTARAAPEI-5, Unigene12956\_TAEpaeTARAAPEI-5, Unigene12994\_TAEpaeTARAAPEI-5, Unigene13000\_TAEpaeTARAAPEI-5, Unigene13029\_TAEpaeTARAAPEI-5, Unigene13163\_TAEpaeTARAAPEI-5, Unigene13233\_TAEpaeTARAAPEI-5, Unigene13359\_TAEpaeTARAAPEI-5, Unigene13399\_TAEpaeTARAAPEI-5, Unigene13451\_TAEpaeTARAAPEI-5, Unigene13484\_TAEpaeTARAAPEI-5, Unigene13532\_TAEpaeTARAAPEI-5, Unigene13629\_TAEpaeTARAAPEI-5, Unigene13650\_TAEpaeTARAAPEI-5, Unigene13666\_TAEpaeTARAAPEI-5, Unigene13747\_TAEpaeTARAAPEI-5, Unigene13787\_TAEpaeTARAAPEI-5, Unigene13859\_TAEpaeTARAAPEI-5, Unigene14178\_TAEpaeTARAAPEI-5, Unigene14362\_TAEpaeTARAAPEI-5, Unigene14366\_TAEpaeTARAAPEI-5, Unigene14369\_TAEpaeTARAAPEI-5, Unigene14384\_TAEpaeTARAAPEI-5, Unigene14714\_TAEpaeTARAAPEI-5, Unigene14719\_TAEpaeTARAAPEI-5, Unigene14720\_TAEpaeTARAAPEI-5, Unigene14809\_TAEpaeTARAAPEI-5, Unigene15053\_TAEpaeTARAAPEI-5, Unigene15075\_TAEpaeTARAAPEI-5, Unigene15107\_TAEpaeTARAAPEI-5, Unigene15247\_TAEpaeTARAAPEI-5, Unigene15356\_TAEpaeTARAAPEI-5, Unigene15361\_TAEpaeTARAAPEI-5, Unigene15468\_TAEpaeTARAAPEI-5, Unigene15547\_TAEpaeTARAAPEI-5, Unigene15549\_TAEpaeTARAAPEI-5, Unigene15626\_TAEpaeTARAAPEI-5, Unigene1570\_TAEpaeTARAAPEI-5, Unigene15730\_TAEpaeTARAAPEI-5, Unigene15881\_TAEpaeTARAAPEI-5, Unigene15911\_TAEpaeTARAAPEI-5, Unigene1593\_TAEpaeTARAAPEI-5, Unigene15971\_TAEpaeTARAAPEI-5, Unigene15987\_TAEpaeTARAAPEI-5, Unigene16406\_TAEpaeTARAAPEI-5, Unigene16411\_TAEpaeTARAAPEI-5, Unigene16524\_TAEpaeTARAAPEI-5, Unigene16581\_TAEpaeTARAAPEI-5, Unigene16824\_TAEpaeTARAAPEI-5, Unigene16892\_TAEpaeTARAAPEI-5, Unigene17034\_TAEpaeTARAAPEI-5, Unigene17109\_TAEpaeTARAAPEI-5, Unigene1712\_TAEpaeTARAAPEI-5, Unigene17145\_TAEpaeTARAAPEI-5, Unigene17271\_TAEpaeTARAAPEI-5, Unigene17354\_TAEpaeTARAAPEI-5, Unigene17364\_TAEpaeTARAAPEI-5, Unigene17409\_TAEpaeTARAAPEI-5, Unigene17420\_TAEpaeTARAAPEI-5, Unigene17475\_TAEpaeTARAAPEI-5, Unigene17535\_TAEpaeTARAAPEI-5, Unigene17551\_TAEpaeTARAAPEI-5, Unigene17557\_TAEpaeTARAAPEI-5, Unigene17569\_TAEpaeTARAAPEI-5, Unigene17570\_TAEpaeTARAAPEI-5, Unigene17614\_TAEpaeTARAAPEI-5, Unigene17633\_TAEpaeTARAAPEI-5, Unigene17722\_TAEpaeTARAAPEI-5, Unigene17731\_TAEpaeTARAAPEI-5, Unigene17741\_TAEpaeTARAAPEI-5, Unigene17835\_TAEpaeTARAAPEI-5, Unigene17840\_TAEpaeTARAAPEI-5, Unigene17865\_TAEpaeTARAAPEI-5, Unigene17901\_TAEpaeTARAAPEI-5, Unigene17912\_TAEpaeTARAAPEI-5, Unigene17977\_TAEpaeTARAAPEI-5, Unigene18057\_TAEpaeTARAAPEI-5, Unigene18085\_TAEpaeTARAAPEI-5, Unigene1808\_TAEpaeTARAAPEI-5, Unigene18309\_TAEpaeTARAAPEI-5, Unigene19035\_TAEpaeTARAAPEI-5, Unigene19396\_TAEpaeTARAAPEI-5, Unigene19465\_TAEpaeTARAAPEI-5, Unigene19552\_TAEpaeTARAAPEI-5, Unigene19746\_TAEpaeTARAAPEI-5, Unigene20000\_TAEpaeTARAAPEI-5, Unigene20573\_TAEpaeTARAAPEI-5, Unigene20837\_TAEpaeTARAAPEI-5, Unigene21363\_TAEpaeTARAAPEI-5, Unigene21425\_TAEpaeTARAAPEI-5, Unigene21513\_TAEpaeTARAAPEI-5, Unigene2206\_TAEpaeTARAAPEI-5, Unigene22165\_TAEpaeTARAAPEI-5, Unigene22358\_TAEpaeTARAAPEI-5, Unigene22762\_TAEpaeTARAAPEI-5, Unigene22842\_TAEpaeTARAAPEI-5, Unigene23077\_TAEpaeTARAAPEI-5, Unigene23436\_TAEpaeTARAAPEI-5, Unigene23485\_TAEpaeTARAAPEI-5, Unigene23498\_TAEpaeTARAAPEI-5, Unigene2360\_TAEpaeTARAAPEI-5, Unigene238\_TAEpaeTARAAPEI-5, Unigene24012\_TAEpaeTARAAPEI-5, Unigene2439\_TAEpaeTARAAPEI-5, Unigene24530\_TAEpaeTARAAPEI-5, Unigene24538\_TAEpaeTARAAPEI-5, Unigene24595\_TAEpaeTARAAPEI-5, Unigene24657\_TAEpaeTARAAPEI-5, Unigene2508\_TAEpaeTARAAPEI-5, Unigene25970\_TAEpaeTARAAPEI-5, Unigene26031\_TAEpaeTARAAPEI-5, Unigene26068\_TAEpaeTARAAPEI-5, Unigene26309\_TAEpaeTARAAPEI-5, Unigene26359\_TAEpaeTARAAPEI-5, Unigene26859\_TAEpaeTARAAPEI-5, Unigene27023\_TAEpaeTARAAPEI-5, Unigene27024\_TAEpaeTARAAPEI-5, Unigene27168\_TAEpaeTARAAPEI-5, Unigene2783\_TAEpaeTARAAPEI-5, Unigene2789\_TAEpaeTARAAPEI-5, Unigene27972\_TAEpaeTARAAPEI-5, Unigene28785\_TAEpaeTARAAPEI-5, Unigene2889\_TAEpaeTARAAPEI-5, Unigene28987\_TAEpaeTARAAPEI-5, Unigene29088\_TAEpaeTARAAPEI-5, Unigene29119\_TAEpaeTARAAPEI-5, Unigene29171\_TAEpaeTARAAPEI-5, Unigene29305\_TAEpaeTARAAPEI-5, Unigene29539\_TAEpaeTARAAPEI-5, Unigene2955\_TAEpaeTARAAPEI-5, Unigene29718\_TAEpaeTARAAPEI-5, Unigene29746\_TAEpaeTARAAPEI-5, Unigene29900\_TAEpaeTARAAPEI-5, Unigene30172\_TAEpaeTARAAPEI-5, Unigene30199\_TAEpaeTARAAPEI-5, Unigene301\_TAEpaeTARAAPEI-5, Unigene30361\_TAEpaeTARAAPEI-5, Unigene30758\_TAEpaeTARAAPEI-5, Unigene30803\_TAEpaeTARAAPEI-5, Unigene30885\_TAEpaeTARAAPEI-5, Unigene30916\_TAEpaeTARAAPEI-5, Unigene30997\_TAEpaeTARAAPEI-5, Unigene31043\_TAEpaeTARAAPEI-5, Unigene31137\_TAEpaeTARAAPEI-5, Unigene31209\_TAEpaeTARAAPEI-5, Unigene3145\_TAEpaeTARAAPEI-5, Unigene3158\_TAEpaeTARAAPEI-5, Unigene31705\_TAEpaeTARAAPEI-5, Unigene31750\_TAEpaeTARAAPEI-5, Unigene31877\_TAEpaeTARAAPEI-5, Unigene32081\_TAEpaeTARAAPEI-5, Unigene32104\_TAEpaeTARAAPEI-5, Unigene32212\_TAEpaeTARAAPEI-5, Unigene32215\_TAEpaeTARAAPEI-5, Unigene32294\_TAEpaeTARAAPEI-5, Unigene32545\_TAEpaeTARAAPEI-5, Unigene32748\_TAEpaeTARAAPEI-5, Unigene32782\_TAEpaeTARAAPEI-5, Unigene32844\_TAEpaeTARAAPEI-5, Unigene32999\_TAEpaeTARAAPEI-5, Unigene33030\_TAEpaeTARAAPEI-5, Unigene33057\_TAEpaeTARAAPEI-5, Unigene33089\_TAEpaeTARAAPEI-5, Unigene33125\_TAEpaeTARAAPEI-5, Unigene33174\_TAEpaeTARAAPEI-5, Unigene33609\_TAEpaeTARAAPEI-5, Unigene34165\_TAEpaeTARAAPEI-5, Unigene34195\_TAEpaeTARAAPEI-5, Unigene35171\_TAEpaeTARAAPEI-5, Unigene35225\_TAEpaeTARAAPEI-5, Unigene35478\_TAEpaeTARAAPEI-5, Unigene35733\_TAEpaeTARAAPEI-5, Unigene35882\_TAEpaeTARAAPEI-5, Unigene35934\_TAEpaeTARAAPEI-5, Unigene36128\_TAEpaeTARAAPEI-5, Unigene36447\_TAEpaeTARAAPEI-5, Unigene36648\_TAEpaeTARAAPEI-5, Unigene36858\_TAEpaeTARAAPEI-5, Unigene36984\_TAEpaeTARAAPEI-5, Unigene37038\_TAEpaeTARAAPEI-5, Unigene37217\_TAEpaeTARAAPEI-5, Unigene37286\_TAEpaeTARAAPEI-5, Unigene37328\_TAEpaeTARAAPEI-5, Unigene37834\_TAEpaeTARAAPEI-5, Unigene37986\_TAEpaeTARAAPEI-5, Unigene38242\_TAEpaeTARAAPEI-5, Unigene38258\_TAEpaeTARAAPEI-5, Unigene3828\_TAEpaeTARAAPEI-5, Unigene38667\_TAEpaeTARAAPEI-5, Unigene38738\_TAEpaeTARAAPEI-5, Unigene3888\_TAEpaeTARAAPEI-5, Unigene38949\_TAEpaeTARAAPEI-5, Unigene39193\_TAEpaeTARAAPEI-5, Unigene39224\_TAEpaeTARAAPEI-5, Unigene39227\_TAEpaeTARAAPEI-5, Unigene39254\_TAEpaeTARAAPEI-5, Unigene39328\_TAEpaeTARAAPEI-5, Unigene39623\_TAEpaeTARAAPEI-5, Unigene39638\_TAEpaeTARAAPEI-5, Unigene39872\_TAEpaeTARAAPEI-5, Unigene39874\_TAEpaeTARAAPEI-5, Unigene39926\_TAEpaeTARAAPEI-5, Unigene40146\_TAEpaeTARAAPEI-5, Unigene40169\_TAEpaeTARAAPEI-5, Unigene40566\_TAEpaeTARAAPEI-5, Unigene40763\_TAEpaeTARAAPEI-5, Unigene40775\_TAEpaeTARAAPEI-5, Unigene40803\_TAEpaeTARAAPEI-5, Unigene40826\_TAEpaeTARAAPEI-5, Unigene40999\_TAEpaeTARAAPEI-5, Unigene4154\_TAEpaeTARAAPEI-5, Unigene42790\_TAEpaeTARAAPEI-5, Unigene42882\_TAEpaeTARAAPEI-5, Unigene43003\_TAEpaeTARAAPEI-5, Unigene43158\_TAEpaeTARAAPEI-5, Unigene43198\_TAEpaeTARAAPEI-5, Unigene43207\_TAEpaeTARAAPEI-5, Unigene43310\_TAEpaeTARAAPEI-5, Unigene43326\_TAEpaeTARAAPEI-5, Unigene4380\_TAEpaeTARAAPEI-5, Unigene4423\_TAEpaeTARAAPEI-5, Unigene44324\_TAEpaeTARAAPEI-5, Unigene44401\_TAEpaeTARAAPEI-5, Unigene44633\_TAEpaeTARAAPEI-5, Unigene44643\_TAEpaeTARAAPEI-5, Unigene44963\_TAEpaeTARAAPEI-5, Unigene45049\_TAEpaeTARAAPEI-5, Unigene45121\_TAEpaeTARAAPEI-5, Unigene45410\_TAEpaeTARAAPEI-5, Unigene45651\_TAEpaeTARAAPEI-5, Unigene45779\_TAEpaeTARAAPEI-5, Unigene45803\_TAEpaeTARAAPEI-5, Unigene460\_TAEpaeTARAAPEI-5, Unigene46118\_TAEpaeTARAAPEI-5, Unigene46174\_TAEpaeTARAAPEI-5, Unigene46290\_TAEpaeTARAAPEI-5, Unigene46349\_TAEpaeTARAAPEI-5, Unigene46659\_TAEpaeTARAAPEI-5, Unigene4737\_TAEpaeTARAAPEI-5, Unigene47434\_TAEpaeTARAAPEI-5, Unigene47449\_TAEpaeTARAAPEI-5, Unigene47733\_TAEpaeTARAAPEI-5, Unigene48214\_TAEpaeTARAAPEI-5, Unigene48267\_TAEpaeTARAAPEI-5, Unigene48353\_TAEpaeTARAAPEI-5, Unigene48636\_TAEpaeTARAAPEI-5, Unigene48705\_TAEpaeTARAAPEI-5, Unigene48725\_TAEpaeTARAAPEI-5, Unigene49023\_TAEpaeTARAAPEI-5, Unigene49173\_TAEpaeTARAAPEI-5, Unigene49239\_TAEpaeTARAAPEI-5, Unigene49598\_TAEpaeTARAAPEI-5, Unigene49887\_TAEpaeTARAAPEI-5, Unigene49990\_TAEpaeTARAAPEI-5, Unigene50024\_TAEpaeTARAAPEI-5, Unigene5033\_TAEpaeTARAAPEI-5, Unigene50474\_TAEpaeTARAAPEI-5, Unigene50566\_TAEpaeTARAAPEI-5, Unigene51078\_TAEpaeTARAAPEI-5, Unigene5141\_TAEpaeTARAAPEI-5, Unigene51704\_TAEpaeTARAAPEI-5, Unigene51722\_TAEpaeTARAAPEI-5, Unigene51767\_TAEpaeTARAAPEI-5, Unigene51813\_TAEpaeTARAAPEI-5, Unigene51834\_TAEpaeTARAAPEI-5, Unigene51903\_TAEpaeTARAAPEI-5, Unigene5194\_TAEpaeTARAAPEI-5, Unigene52030\_TAEpaeTARAAPEI-5, Unigene52242\_TAEpaeTARAAPEI-5, Unigene52306\_TAEpaeTARAAPEI-5, Unigene52312\_TAEpaeTARAAPEI-5, Unigene5248\_TAEpaeTARAAPEI-5, Unigene53776\_TAEpaeTARAAPEI-5, Unigene53786\_TAEpaeTARAAPEI-5, Unigene54029\_TAEpaeTARAAPEI-5, Unigene54206\_TAEpaeTARAAPEI-5, Unigene54313\_TAEpaeTARAAPEI-5, Unigene54363\_TAEpaeTARAAPEI-5, Unigene54619\_TAEpaeTARAAPEI-5, Unigene54738\_TAEpaeTARAAPEI-5, Unigene54767\_TAEpaeTARAAPEI-5, Unigene54848\_TAEpaeTARAAPEI-5, Unigene55027\_TAEpaeTARAAPEI-5, Unigene55145\_TAEpaeTARAAPEI-5, Unigene5518\_TAEpaeTARAAPEI-5, Unigene55238\_TAEpaeTARAAPEI-5, Unigene5555\_TAEpaeTARAAPEI-5, Unigene55561\_TAEpaeTARAAPEI-5, Unigene55704\_TAEpaeTARAAPEI-5, Unigene5577\_TAEpaeTARAAPEI-5, Unigene55783\_TAEpaeTARAAPEI-5, Unigene55789\_TAEpaeTARAAPEI-5, Unigene55862\_TAEpaeTARAAPEI-5, Unigene55930\_TAEpaeTARAAPEI-5, Unigene56001\_TAEpaeTARAAPEI-5, Unigene56110\_TAEpaeTARAAPEI-5, Unigene56240\_TAEpaeTARAAPEI-5, Unigene56429\_TAEpaeTARAAPEI-5, Unigene5655\_TAEpaeTARAAPEI-5, Unigene56770\_TAEpaeTARAAPEI-5, Unigene56860\_TAEpaeTARAAPEI-5, Unigene5694\_TAEpaeTARAAPEI-5, Unigene57076\_TAEpaeTARAAPEI-5, Unigene57095\_TAEpaeTARAAPEI-5, Unigene57151\_TAEpaeTARAAPEI-5, Unigene57203\_TAEpaeTARAAPEI-5, Unigene57223\_TAEpaeTARAAPEI-5, Unigene57307\_TAEpaeTARAAPEI-5, Unigene57365\_TAEpaeTARAAPEI-5, Unigene57376\_TAEpaeTARAAPEI-5, Unigene57482\_TAEpaeTARAAPEI-5, Unigene57497\_TAEpaeTARAAPEI-5, Unigene5753\_TAEpaeTARAAPEI-5, Unigene57575\_TAEpaeTARAAPEI-5, Unigene57607\_TAEpaeTARAAPEI-5, Unigene57790\_TAEpaeTARAAPEI-5, Unigene58137\_TAEpaeTARAAPEI-5, Unigene58297\_TAEpaeTARAAPEI-5, Unigene58658\_TAEpaeTARAAPEI-5, Unigene58782\_TAEpaeTARAAPEI-5, Unigene58938\_TAEpaeTARAAPEI-5, Unigene59088\_TAEpaeTARAAPEI-5, Unigene59280\_TAEpaeTARAAPEI-5, Unigene59312\_TAEpaeTARAAPEI-5, Unigene59328\_TAEpaeTARAAPEI-5, Unigene59373\_TAEpaeTARAAPEI-5, Unigene59596\_TAEpaeTARAAPEI-5, Unigene59648\_TAEpaeTARAAPEI-5, Unigene59670\_TAEpaeTARAAPEI-5, Unigene59674\_TAEpaeTARAAPEI-5, Unigene59697\_TAEpaeTARAAPEI-5, Unigene5970\_TAEpaeTARAAPEI-5, Unigene59726\_TAEpaeTARAAPEI-5, Unigene60131\_TAEpaeTARAAPEI-5, Unigene60140\_TAEpaeTARAAPEI-5, Unigene60200\_TAEpaeTARAAPEI-5, Unigene6030\_TAEpaeTARAAPEI-5, Unigene60330\_TAEpaeTARAAPEI-5, Unigene60606\_TAEpaeTARAAPEI-5, Unigene60653\_TAEpaeTARAAPEI-5, Unigene60808\_TAEpaeTARAAPEI-5, Unigene60837\_TAEpaeTARAAPEI-5, Unigene60934\_TAEpaeTARAAPEI-5, Unigene60983\_TAEpaeTARAAPEI-5, Unigene60986\_TAEpaeTARAAPEI-5, Unigene60992\_TAEpaeTARAAPEI-5, Unigene61114\_TAEpaeTARAAPEI-5, Unigene61230\_TAEpaeTARAAPEI-5, Unigene61425\_TAEpaeTARAAPEI-5, Unigene6150\_TAEpaeTARAAPEI-5, Unigene61640\_TAEpaeTARAAPEI-5, Unigene61699\_TAEpaeTARAAPEI-5, Unigene61772\_TAEpaeTARAAPEI-5, Unigene61806\_TAEpaeTARAAPEI-5, Unigene61820\_TAEpaeTARAAPEI-5, Unigene61823\_TAEpaeTARAAPEI-5, Unigene61864\_TAEpaeTARAAPEI-5, Unigene6187\_TAEpaeTARAAPEI-5, Unigene62064\_TAEpaeTARAAPEI-5, Unigene62072\_TAEpaeTARAAPEI-5, Unigene62127\_TAEpaeTARAAPEI-5, Unigene62217\_TAEpaeTARAAPEI-5, Unigene62263\_TAEpaeTARAAPEI-5, Unigene6244\_TAEpaeTARAAPEI-5, Unigene62583\_TAEpaeTARAAPEI-5, Unigene6262\_TAEpaeTARAAPEI-5, Unigene62653\_TAEpaeTARAAPEI-5, Unigene62673\_TAEpaeTARAAPEI-5, Unigene62723\_TAEpaeTARAAPEI-5, Unigene62795\_TAEpaeTARAAPEI-5, Unigene62920\_TAEpaeTARAAPEI-5, Unigene62944\_TAEpaeTARAAPEI-5, Unigene62950\_TAEpaeTARAAPEI-5, Unigene63067\_TAEpaeTARAAPEI-5, Unigene63148\_TAEpaeTARAAPEI-5, Unigene63172\_TAEpaeTARAAPEI-5, Unigene63227\_TAEpaeTARAAPEI-5, Unigene63253\_TAEpaeTARAAPEI-5, Unigene63349\_TAEpaeTARAAPEI-5, Unigene63370\_TAEpaeTARAAPEI-5, Unigene63565\_TAEpaeTARAAPEI-5, Unigene63590\_TAEpaeTARAAPEI-5, Unigene63664\_TAEpaeTARAAPEI-5, Unigene63705\_TAEpaeTARAAPEI-5, Unigene6372\_TAEpaeTARAAPEI-5, Unigene63743\_TAEpaeTARAAPEI-5, Unigene63809\_TAEpaeTARAAPEI-5, Unigene63956\_TAEpaeTARAAPEI-5, Unigene64029\_TAEpaeTARAAPEI-5, Unigene64040\_TAEpaeTARAAPEI-5, Unigene64114\_TAEpaeTARAAPEI-5, Unigene64122\_TAEpaeTARAAPEI-5, Unigene64135\_TAEpaeTARAAPEI-5, Unigene64140\_TAEpaeTARAAPEI-5, Unigene64150\_TAEpaeTARAAPEI-5, Unigene64160\_TAEpaeTARAAPEI-5, Unigene64253\_TAEpaeTARAAPEI-5, Unigene64361\_TAEpaeTARAAPEI-5, Unigene64398\_TAEpaeTARAAPEI-5, Unigene64529\_TAEpaeTARAAPEI-5, Unigene6452\_TAEpaeTARAAPEI-5, Unigene64537\_TAEpaeTARAAPEI-5, Unigene64618\_TAEpaeTARAAPEI-5, Unigene64673\_TAEpaeTARAAPEI-5, Unigene64687\_TAEpaeTARAAPEI-5, Unigene64867\_TAEpaeTARAAPEI-5, Unigene65037\_TAEpaeTARAAPEI-5, Unigene65087\_TAEpaeTARAAPEI-5, Unigene65145\_TAEpaeTARAAPEI-5, Unigene65194\_TAEpaeTARAAPEI-5, Unigene65239\_TAEpaeTARAAPEI-5, Unigene65279\_TAEpaeTARAAPEI-5, Unigene65293\_TAEpaeTARAAPEI-5, Unigene65314\_TAEpaeTARAAPEI-5, Unigene65322\_TAEpaeTARAAPEI-5, Unigene65468\_TAEpaeTARAAPEI-5, Unigene65502\_TAEpaeTARAAPEI-5, Unigene65570\_TAEpaeTARAAPEI-5, Unigene65636\_TAEpaeTARAAPEI-5, Unigene65658\_TAEpaeTARAAPEI-5, Unigene65685\_TAEpaeTARAAPEI-5, Unigene65738\_TAEpaeTARAAPEI-5, Unigene65827\_TAEpaeTARAAPEI-5, Unigene65902\_TAEpaeTARAAPEI-5, Unigene66004\_TAEpaeTARAAPEI-5, Unigene66009\_TAEpaeTARAAPEI-5, Unigene66043\_TAEpaeTARAAPEI-5, Unigene6605\_TAEpaeTARAAPEI-5, Unigene66158\_TAEpaeTARAAPEI-5, Unigene66166\_TAEpaeTARAAPEI-5, Unigene66167\_TAEpaeTARAAPEI-5, Unigene66212\_TAEpaeTARAAPEI-5, Unigene66225\_TAEpaeTARAAPEI-5, Unigene66309\_TAEpaeTARAAPEI-5, Unigene6635\_TAEpaeTARAAPEI-5, Unigene66391\_TAEpaeTARAAPEI-5, Unigene66440\_TAEpaeTARAAPEI-5, Unigene66459\_TAEpaeTARAAPEI-5, Unigene66492\_TAEpaeTARAAPEI-5, Unigene66648\_TAEpaeTARAAPEI-5, Unigene66666\_TAEpaeTARAAPEI-5, Unigene66694\_TAEpaeTARAAPEI-5, Unigene66742\_TAEpaeTARAAPEI-5, Unigene66799\_TAEpaeTARAAPEI-5, Unigene66931\_TAEpaeTARAAPEI-5, Unigene67019\_TAEpaeTARAAPEI-5, Unigene67063\_TAEpaeTARAAPEI-5, Unigene67064\_TAEpaeTARAAPEI-5, Unigene67134\_TAEpaeTARAAPEI-5, Unigene67310\_TAEpaeTARAAPEI-5, Unigene67335\_TAEpaeTARAAPEI-5, Unigene67341\_TAEpaeTARAAPEI-5, Unigene6745\_TAEpaeTARAAPEI-5, Unigene67509\_TAEpaeTARAAPEI-5, Unigene67577\_TAEpaeTARAAPEI-5, Unigene67579\_TAEpaeTARAAPEI-5, Unigene67597\_TAEpaeTARAAPEI-5, Unigene67634\_TAEpaeTARAAPEI-5, Unigene67664\_TAEpaeTARAAPEI-5, Unigene67745\_TAEpaeTARAAPEI-5, Unigene67748\_TAEpaeTARAAPEI-5, Unigene67781\_TAEpaeTARAAPEI-5, Unigene67889\_TAEpaeTARAAPEI-5, Unigene67930\_TAEpaeTARAAPEI-5, Unigene68000\_TAEpaeTARAAPEI-5, Unigene68142\_TAEpaeTARAAPEI-5, Unigene68154\_TAEpaeTARAAPEI-5, Unigene68171\_TAEpaeTARAAPEI-5, Unigene68189\_TAEpaeTARAAPEI-5, Unigene68205\_TAEpaeTARAAPEI-5, Unigene68274\_TAEpaeTARAAPEI-5, Unigene68278\_TAEpaeTARAAPEI-5, Unigene68285\_TAEpaeTARAAPEI-5, Unigene68293\_TAEpaeTARAAPEI-5, Unigene68333\_TAEpaeTARAAPEI-5, Unigene68591\_TAEpaeTARAAPEI-5, Unigene68625\_TAEpaeTARAAPEI-5, Unigene68644\_TAEpaeTARAAPEI-5, Unigene68686\_TAEpaeTARAAPEI-5, Unigene68730\_TAEpaeTARAAPEI-5, Unigene68778\_TAEpaeTARAAPEI-5, Unigene68839\_TAEpaeTARAAPEI-5, Unigene68855\_TAEpaeTARAAPEI-5, Unigene68869\_TAEpaeTARAAPEI-5, Unigene68874\_TAEpaeTARAAPEI-5, Unigene68878\_TAEpaeTARAAPEI-5, Unigene68984\_TAEpaeTARAAPEI-5, Unigene69009\_TAEpaeTARAAPEI-5, Unigene69062\_TAEpaeTARAAPEI-5, Unigene69100\_TAEpaeTARAAPEI-5, Unigene69182\_TAEpaeTARAAPEI-5, Unigene69189\_TAEpaeTARAAPEI-5, Unigene69275\_TAEpaeTARAAPEI-5, Unigene69314\_TAEpaeTARAAPEI-5, Unigene69370\_TAEpaeTARAAPEI-5, Unigene6938\_TAEpaeTARAAPEI-5, Unigene69400\_TAEpaeTARAAPEI-5, Unigene6940\_TAEpaeTARAAPEI-5, Unigene69421\_TAEpaeTARAAPEI-5, Unigene69436\_TAEpaeTARAAPEI-5, Unigene69441\_TAEpaeTARAAPEI-5, Unigene69461\_TAEpaeTARAAPEI-5, Unigene69467\_TAEpaeTARAAPEI-5, Unigene69512\_TAEpaeTARAAPEI-5, Unigene69577\_TAEpaeTARAAPEI-5, Unigene69636\_TAEpaeTARAAPEI-5, Unigene69654\_TAEpaeTARAAPEI-5, Unigene69688\_TAEpaeTARAAPEI-5, Unigene69719\_TAEpaeTARAAPEI-5, Unigene69773\_TAEpaeTARAAPEI-5, Unigene69775\_TAEpaeTARAAPEI-5, Unigene69776\_TAEpaeTARAAPEI-5, Unigene69849\_TAEpaeTARAAPEI-5, Unigene70033\_TAEpaeTARAAPEI-5, Unigene70061\_TAEpaeTARAAPEI-5, Unigene70074\_TAEpaeTARAAPEI-5, Unigene70091\_TAEpaeTARAAPEI-5, Unigene70103\_TAEpaeTARAAPEI-5, Unigene70110\_TAEpaeTARAAPEI-5, Unigene7011\_TAEpaeTARAAPEI-5, Unigene70176\_TAEpaeTARAAPEI-5, Unigene70182\_TAEpaeTARAAPEI-5, Unigene70207\_TAEpaeTARAAPEI-5, Unigene70233\_TAEpaeTARAAPEI-5, Unigene70289\_TAEpaeTARAAPEI-5, Unigene70309\_TAEpaeTARAAPEI-5, Unigene70316\_TAEpaeTARAAPEI-5, Unigene70319\_TAEpaeTARAAPEI-5, Unigene70372\_TAEpaeTARAAPEI-5, Unigene70533\_TAEpaeTARAAPEI-5, Unigene70535\_TAEpaeTARAAPEI-5, Unigene70571\_TAEpaeTARAAPEI-5, Unigene70579\_TAEpaeTARAAPEI-5, Unigene70607\_TAEpaeTARAAPEI-5, Unigene70615\_TAEpaeTARAAPEI-5, Unigene70616\_TAEpaeTARAAPEI-5, Unigene70659\_TAEpaeTARAAPEI-5, Unigene70663\_TAEpaeTARAAPEI-5, Unigene70751\_TAEpaeTARAAPEI-5, Unigene70761\_TAEpaeTARAAPEI-5, Unigene70767\_TAEpaeTARAAPEI-5, Unigene70828\_TAEpaeTARAAPEI-5, Unigene70890\_TAEpaeTARAAPEI-5, Unigene70941\_TAEpaeTARAAPEI-5, Unigene71024\_TAEpaeTARAAPEI-5, Unigene71038\_TAEpaeTARAAPEI-5, Unigene71075\_TAEpaeTARAAPEI-5, Unigene71086\_TAEpaeTARAAPEI-5, Unigene71141\_TAEpaeTARAAPEI-5, Unigene71142\_TAEpaeTARAAPEI-5, Unigene71182\_TAEpaeTARAAPEI-5, Unigene71268\_TAEpaeTARAAPEI-5, Unigene71271\_TAEpaeTARAAPEI-5, Unigene71294\_TAEpaeTARAAPEI-5, Unigene71299\_TAEpaeTARAAPEI-5, Unigene71305\_TAEpaeTARAAPEI-5, Unigene71310\_TAEpaeTARAAPEI-5, Unigene71356\_TAEpaeTARAAPEI-5, Unigene71380\_TAEpaeTARAAPEI-5, Unigene71449\_TAEpaeTARAAPEI-5, Unigene71498\_TAEpaeTARAAPEI-5, Unigene71553\_TAEpaeTARAAPEI-5, Unigene71621\_TAEpaeTARAAPEI-5, Unigene71632\_TAEpaeTARAAPEI-5, Unigene71635\_TAEpaeTARAAPEI-5, Unigene71636\_TAEpaeTARAAPEI-5, Unigene71684\_TAEpaeTARAAPEI-5, Unigene71745\_TAEpaeTARAAPEI-5, Unigene71756\_TAEpaeTARAAPEI-5, Unigene71760\_TAEpaeTARAAPEI-5, Unigene71779\_TAEpaeTARAAPEI-5, Unigene71787\_TAEpaeTARAAPEI-5, Unigene71835\_TAEpaeTARAAPEI-5, Unigene71839\_TAEpaeTARAAPEI-5, Unigene71869\_TAEpaeTARAAPEI-5, Unigene72045\_TAEpaeTARAAPEI-5, Unigene72087\_TAEpaeTARAAPEI-5, Unigene72109\_TAEpaeTARAAPEI-5, Unigene72113\_TAEpaeTARAAPEI-5, Unigene72134\_TAEpaeTARAAPEI-5, Unigene72168\_TAEpaeTARAAPEI-5, Unigene72181\_TAEpaeTARAAPEI-5, Unigene72213\_TAEpaeTARAAPEI-5, Unigene72258\_TAEpaeTARAAPEI-5, Unigene72261\_TAEpaeTARAAPEI-5, Unigene72266\_TAEpaeTARAAPEI-5, Unigene7227\_TAEpaeTARAAPEI-5, Unigene72287\_TAEpaeTARAAPEI-5, Unigene72350\_TAEpaeTARAAPEI-5, Unigene72355\_TAEpaeTARAAPEI-5, Unigene72392\_TAEpaeTARAAPEI-5, Unigene72471\_TAEpaeTARAAPEI-5, Unigene72473\_TAEpaeTARAAPEI-5, Unigene72493\_TAEpaeTARAAPEI-5, Unigene72494\_TAEpaeTARAAPEI-5, Unigene72502\_TAEpaeTARAAPEI-5, Unigene72586\_TAEpaeTARAAPEI-5, Unigene72588\_TAEpaeTARAAPEI-5, Unigene72593\_TAEpaeTARAAPEI-5, Unigene72607\_TAEpaeTARAAPEI-5, Unigene72635\_TAEpaeTARAAPEI-5, Unigene72670\_TAEpaeTARAAPEI-5, Unigene72690\_TAEpaeTARAAPEI-5, Unigene72707\_TAEpaeTARAAPEI-5, Unigene72711\_TAEpaeTARAAPEI-5, Unigene72716\_TAEpaeTARAAPEI-5, Unigene72746\_TAEpaeTARAAPEI-5, Unigene72749\_TAEpaeTARAAPEI-5, Unigene72800\_TAEpaeTARAAPEI-5, Unigene72812\_TAEpaeTARAAPEI-5, Unigene72823\_TAEpaeTARAAPEI-5, Unigene72825\_TAEpaeTARAAPEI-5, Unigene72849\_TAEpaeTARAAPEI-5, Unigene72875\_TAEpaeTARAAPEI-5, Unigene72896\_TAEpaeTARAAPEI-5, Unigene72903\_TAEpaeTARAAPEI-5, Unigene72939\_TAEpaeTARAAPEI-5, Unigene7309\_TAEpaeTARAAPEI-5, Unigene7482\_TAEpaeTARAAPEI-5, Unigene7844\_TAEpaeTARAAPEI-5, Unigene7878\_TAEpaeTARAAPEI-5, Unigene7994\_TAEpaeTARAAPEI-5, Unigene8110\_TAEpaeTARAAPEI-5, Unigene8218\_TAEpaeTARAAPEI-5, Unigene8371\_TAEpaeTARAAPEI-5, Unigene8876\_TAEpaeTARAAPEI-5, Unigene8920\_TAEpaeTARAAPEI-5, Unigene9304\_TAEpaeTARAAPEI-5, Unigene9392\_TAEpaeTARAAPEI-5, Unigene943\_TAEpaeTARAAPEI-5, Unigene9723\_TAEpaeTARAAPEI-5, Unigene9731\_TAEpaeTARAAPEI-5, Unigene9814\_TAEpaeTARAAPEI-5, Unigene9962\_TAEpaeTARAAPEI-5 |
| 4 | Huntington's disease | Unigene10016\_TAEpaeTARAAPEI-5, Unigene10085\_TAEpaeTARAAPEI-5, Unigene10137\_TAEpaeTARAAPEI-5, Unigene10194\_TAEpaeTARAAPEI-5, Unigene10279\_TAEpaeTARAAPEI-5, Unigene10428\_TAEpaeTARAAPEI-5, Unigene10784\_TAEpaeTARAAPEI-5, Unigene10838\_TAEpaeTARAAPEI-5, Unigene10871\_TAEpaeTARAAPEI-5, Unigene11257\_TAEpaeTARAAPEI-5, Unigene11418\_TAEpaeTARAAPEI-5, Unigene11507\_TAEpaeTARAAPEI-5, Unigene11775\_TAEpaeTARAAPEI-5, Unigene11964\_TAEpaeTARAAPEI-5, Unigene12151\_TAEpaeTARAAPEI-5, Unigene12387\_TAEpaeTARAAPEI-5, Unigene12432\_TAEpaeTARAAPEI-5, Unigene12538\_TAEpaeTARAAPEI-5, Unigene12567\_TAEpaeTARAAPEI-5, Unigene12591\_TAEpaeTARAAPEI-5, Unigene12631\_TAEpaeTARAAPEI-5, Unigene12674\_TAEpaeTARAAPEI-5, Unigene12729\_TAEpaeTARAAPEI-5, Unigene1274\_TAEpaeTARAAPEI-5, Unigene12852\_TAEpaeTARAAPEI-5, Unigene13067\_TAEpaeTARAAPEI-5, Unigene13108\_TAEpaeTARAAPEI-5, Unigene13121\_TAEpaeTARAAPEI-5, Unigene13162\_TAEpaeTARAAPEI-5, Unigene13454\_TAEpaeTARAAPEI-5, Unigene13473\_TAEpaeTARAAPEI-5, Unigene13541\_TAEpaeTARAAPEI-5, Unigene13546\_TAEpaeTARAAPEI-5, Unigene13656\_TAEpaeTARAAPEI-5, Unigene13760\_TAEpaeTARAAPEI-5, Unigene13812\_TAEpaeTARAAPEI-5, Unigene13829\_TAEpaeTARAAPEI-5, Unigene13872\_TAEpaeTARAAPEI-5, Unigene1389\_TAEpaeTARAAPEI-5, Unigene13956\_TAEpaeTARAAPEI-5, Unigene14037\_TAEpaeTARAAPEI-5, Unigene14095\_TAEpaeTARAAPEI-5, Unigene14105\_TAEpaeTARAAPEI-5, Unigene14161\_TAEpaeTARAAPEI-5, Unigene14181\_TAEpaeTARAAPEI-5, Unigene14243\_TAEpaeTARAAPEI-5, Unigene14408\_TAEpaeTARAAPEI-5, Unigene14432\_TAEpaeTARAAPEI-5, Unigene14566\_TAEpaeTARAAPEI-5, Unigene14588\_TAEpaeTARAAPEI-5, Unigene14660\_TAEpaeTARAAPEI-5, Unigene1467\_TAEpaeTARAAPEI-5, Unigene14741\_TAEpaeTARAAPEI-5, Unigene14791\_TAEpaeTARAAPEI-5, Unigene14793\_TAEpaeTARAAPEI-5, Unigene14840\_TAEpaeTARAAPEI-5, Unigene14847\_TAEpaeTARAAPEI-5, Unigene14897\_TAEpaeTARAAPEI-5, Unigene14928\_TAEpaeTARAAPEI-5, Unigene14945\_TAEpaeTARAAPEI-5, Unigene14963\_TAEpaeTARAAPEI-5, Unigene15078\_TAEpaeTARAAPEI-5, Unigene15148\_TAEpaeTARAAPEI-5, Unigene15450\_TAEpaeTARAAPEI-5, Unigene15599\_TAEpaeTARAAPEI-5, Unigene15649\_TAEpaeTARAAPEI-5, Unigene15713\_TAEpaeTARAAPEI-5, Unigene15734\_TAEpaeTARAAPEI-5, Unigene15747\_TAEpaeTARAAPEI-5, Unigene15759\_TAEpaeTARAAPEI-5, Unigene157\_TAEpaeTARAAPEI-5, Unigene15853\_TAEpaeTARAAPEI-5, Unigene15905\_TAEpaeTARAAPEI-5, Unigene16034\_TAEpaeTARAAPEI-5, Unigene16140\_TAEpaeTARAAPEI-5, Unigene16213\_TAEpaeTARAAPEI-5, Unigene16250\_TAEpaeTARAAPEI-5, Unigene16311\_TAEpaeTARAAPEI-5, Unigene16387\_TAEpaeTARAAPEI-5, Unigene16399\_TAEpaeTARAAPEI-5, Unigene16457\_TAEpaeTARAAPEI-5, Unigene16476\_TAEpaeTARAAPEI-5, Unigene16515\_TAEpaeTARAAPEI-5, Unigene1657\_TAEpaeTARAAPEI-5, Unigene16599\_TAEpaeTARAAPEI-5, Unigene16682\_TAEpaeTARAAPEI-5, Unigene16749\_TAEpaeTARAAPEI-5, Unigene16789\_TAEpaeTARAAPEI-5, Unigene167\_TAEpaeTARAAPEI-5, Unigene16893\_TAEpaeTARAAPEI-5, Unigene16913\_TAEpaeTARAAPEI-5, Unigene16974\_TAEpaeTARAAPEI-5, Unigene16984\_TAEpaeTARAAPEI-5, Unigene17051\_TAEpaeTARAAPEI-5, Unigene17071\_TAEpaeTARAAPEI-5, Unigene17097\_TAEpaeTARAAPEI-5, Unigene1710\_TAEpaeTARAAPEI-5, Unigene17265\_TAEpaeTARAAPEI-5, Unigene17278\_TAEpaeTARAAPEI-5, Unigene17301\_TAEpaeTARAAPEI-5, Unigene17303\_TAEpaeTARAAPEI-5, Unigene17326\_TAEpaeTARAAPEI-5, Unigene17336\_TAEpaeTARAAPEI-5, Unigene17466\_TAEpaeTARAAPEI-5, Unigene17507\_TAEpaeTARAAPEI-5, Unigene17529\_TAEpaeTARAAPEI-5, Unigene17558\_TAEpaeTARAAPEI-5, Unigene17573\_TAEpaeTARAAPEI-5, Unigene17600\_TAEpaeTARAAPEI-5, Unigene17703\_TAEpaeTARAAPEI-5, Unigene17725\_TAEpaeTARAAPEI-5, Unigene17827\_TAEpaeTARAAPEI-5, Unigene17863\_TAEpaeTARAAPEI-5, Unigene17938\_TAEpaeTARAAPEI-5, Unigene17970\_TAEpaeTARAAPEI-5, Unigene18010\_TAEpaeTARAAPEI-5, Unigene18014\_TAEpaeTARAAPEI-5, Unigene18089\_TAEpaeTARAAPEI-5, Unigene1812\_TAEpaeTARAAPEI-5, Unigene18312\_TAEpaeTARAAPEI-5, Unigene1839\_TAEpaeTARAAPEI-5, Unigene18427\_TAEpaeTARAAPEI-5, Unigene18619\_TAEpaeTARAAPEI-5, Unigene1864\_TAEpaeTARAAPEI-5, Unigene1873\_TAEpaeTARAAPEI-5, Unigene19332\_TAEpaeTARAAPEI-5, Unigene1953\_TAEpaeTARAAPEI-5, Unigene19640\_TAEpaeTARAAPEI-5, Unigene1967\_TAEpaeTARAAPEI-5, Unigene2014\_TAEpaeTARAAPEI-5, Unigene20172\_TAEpaeTARAAPEI-5, Unigene20241\_TAEpaeTARAAPEI-5, Unigene20503\_TAEpaeTARAAPEI-5, Unigene20549\_TAEpaeTARAAPEI-5, Unigene20675\_TAEpaeTARAAPEI-5, Unigene20745\_TAEpaeTARAAPEI-5, Unigene21040\_TAEpaeTARAAPEI-5, Unigene21215\_TAEpaeTARAAPEI-5, Unigene21299\_TAEpaeTARAAPEI-5, Unigene21445\_TAEpaeTARAAPEI-5, Unigene21511\_TAEpaeTARAAPEI-5, Unigene2158\_TAEpaeTARAAPEI-5, Unigene21780\_TAEpaeTARAAPEI-5, Unigene2202\_TAEpaeTARAAPEI-5, Unigene22196\_TAEpaeTARAAPEI-5, Unigene22349\_TAEpaeTARAAPEI-5, Unigene22557\_TAEpaeTARAAPEI-5, Unigene22944\_TAEpaeTARAAPEI-5, Unigene23154\_TAEpaeTARAAPEI-5, Unigene23185\_TAEpaeTARAAPEI-5, Unigene23248\_TAEpaeTARAAPEI-5, Unigene2350\_TAEpaeTARAAPEI-5, Unigene24306\_TAEpaeTARAAPEI-5, Unigene24316\_TAEpaeTARAAPEI-5, Unigene24598\_TAEpaeTARAAPEI-5, Unigene24831\_TAEpaeTARAAPEI-5, Unigene25114\_TAEpaeTARAAPEI-5, Unigene25288\_TAEpaeTARAAPEI-5, Unigene25341\_TAEpaeTARAAPEI-5, Unigene25611\_TAEpaeTARAAPEI-5, Unigene26004\_TAEpaeTARAAPEI-5, Unigene26124\_TAEpaeTARAAPEI-5, Unigene26135\_TAEpaeTARAAPEI-5, Unigene26199\_TAEpaeTARAAPEI-5, Unigene26231\_TAEpaeTARAAPEI-5, Unigene26669\_TAEpaeTARAAPEI-5, Unigene26939\_TAEpaeTARAAPEI-5, Unigene27534\_TAEpaeTARAAPEI-5, Unigene27593\_TAEpaeTARAAPEI-5, Unigene27629\_TAEpaeTARAAPEI-5, Unigene27931\_TAEpaeTARAAPEI-5, Unigene2828\_TAEpaeTARAAPEI-5, Unigene2852\_TAEpaeTARAAPEI-5, Unigene28578\_TAEpaeTARAAPEI-5, Unigene2862\_TAEpaeTARAAPEI-5, Unigene28660\_TAEpaeTARAAPEI-5, Unigene28988\_TAEpaeTARAAPEI-5, Unigene28\_TAEpaeTARAAPEI-5, Unigene29087\_TAEpaeTARAAPEI-5, Unigene29119\_TAEpaeTARAAPEI-5, Unigene29132\_TAEpaeTARAAPEI-5, Unigene29158\_TAEpaeTARAAPEI-5, Unigene29193\_TAEpaeTARAAPEI-5, Unigene2923\_TAEpaeTARAAPEI-5, Unigene29272\_TAEpaeTARAAPEI-5, Unigene29381\_TAEpaeTARAAPEI-5, Unigene293\_TAEpaeTARAAPEI-5, Unigene29460\_TAEpaeTARAAPEI-5, Unigene30015\_TAEpaeTARAAPEI-5, Unigene30101\_TAEpaeTARAAPEI-5, Unigene30135\_TAEpaeTARAAPEI-5, Unigene3027\_TAEpaeTARAAPEI-5, Unigene30744\_TAEpaeTARAAPEI-5, Unigene30861\_TAEpaeTARAAPEI-5, Unigene3089\_TAEpaeTARAAPEI-5, Unigene3091\_TAEpaeTARAAPEI-5, Unigene31040\_TAEpaeTARAAPEI-5, Unigene31394\_TAEpaeTARAAPEI-5, Unigene3144\_TAEpaeTARAAPEI-5, Unigene31709\_TAEpaeTARAAPEI-5, Unigene31846\_TAEpaeTARAAPEI-5, Unigene32338\_TAEpaeTARAAPEI-5, Unigene32425\_TAEpaeTARAAPEI-5, Unigene32934\_TAEpaeTARAAPEI-5, Unigene33014\_TAEpaeTARAAPEI-5, Unigene33391\_TAEpaeTARAAPEI-5, Unigene33549\_TAEpaeTARAAPEI-5, Unigene33859\_TAEpaeTARAAPEI-5, Unigene33881\_TAEpaeTARAAPEI-5, Unigene34000\_TAEpaeTARAAPEI-5, Unigene34024\_TAEpaeTARAAPEI-5, Unigene34043\_TAEpaeTARAAPEI-5, Unigene3417\_TAEpaeTARAAPEI-5, Unigene34285\_TAEpaeTARAAPEI-5, Unigene34547\_TAEpaeTARAAPEI-5, Unigene34897\_TAEpaeTARAAPEI-5, Unigene35153\_TAEpaeTARAAPEI-5, Unigene35164\_TAEpaeTARAAPEI-5, Unigene35612\_TAEpaeTARAAPEI-5, Unigene35653\_TAEpaeTARAAPEI-5, Unigene35948\_TAEpaeTARAAPEI-5, Unigene35956\_TAEpaeTARAAPEI-5, Unigene36036\_TAEpaeTARAAPEI-5, Unigene36116\_TAEpaeTARAAPEI-5, Unigene36211\_TAEpaeTARAAPEI-5, Unigene36701\_TAEpaeTARAAPEI-5, Unigene36803\_TAEpaeTARAAPEI-5, Unigene36880\_TAEpaeTARAAPEI-5, Unigene37156\_TAEpaeTARAAPEI-5, Unigene37280\_TAEpaeTARAAPEI-5, Unigene3729\_TAEpaeTARAAPEI-5, Unigene37331\_TAEpaeTARAAPEI-5, Unigene37367\_TAEpaeTARAAPEI-5, Unigene37487\_TAEpaeTARAAPEI-5, Unigene37539\_TAEpaeTARAAPEI-5, Unigene37828\_TAEpaeTARAAPEI-5, Unigene38062\_TAEpaeTARAAPEI-5, Unigene38127\_TAEpaeTARAAPEI-5, Unigene38306\_TAEpaeTARAAPEI-5, Unigene38618\_TAEpaeTARAAPEI-5, Unigene38636\_TAEpaeTARAAPEI-5, Unigene38671\_TAEpaeTARAAPEI-5, Unigene38716\_TAEpaeTARAAPEI-5, Unigene38775\_TAEpaeTARAAPEI-5, Unigene38851\_TAEpaeTARAAPEI-5, Unigene39041\_TAEpaeTARAAPEI-5, Unigene39051\_TAEpaeTARAAPEI-5, Unigene39274\_TAEpaeTARAAPEI-5, Unigene39301\_TAEpaeTARAAPEI-5, Unigene39328\_TAEpaeTARAAPEI-5, Unigene39396\_TAEpaeTARAAPEI-5, Unigene39411\_TAEpaeTARAAPEI-5, Unigene39522\_TAEpaeTARAAPEI-5, Unigene39680\_TAEpaeTARAAPEI-5, Unigene39855\_TAEpaeTARAAPEI-5, Unigene39867\_TAEpaeTARAAPEI-5, Unigene39976\_TAEpaeTARAAPEI-5, Unigene40221\_TAEpaeTARAAPEI-5, Unigene40782\_TAEpaeTARAAPEI-5, Unigene40806\_TAEpaeTARAAPEI-5, Unigene41208\_TAEpaeTARAAPEI-5, Unigene41388\_TAEpaeTARAAPEI-5, Unigene41473\_TAEpaeTARAAPEI-5, Unigene4164\_TAEpaeTARAAPEI-5, Unigene41799\_TAEpaeTARAAPEI-5, Unigene42217\_TAEpaeTARAAPEI-5, Unigene42360\_TAEpaeTARAAPEI-5, Unigene42657\_TAEpaeTARAAPEI-5, Unigene4275\_TAEpaeTARAAPEI-5, Unigene42986\_TAEpaeTARAAPEI-5, Unigene43099\_TAEpaeTARAAPEI-5, Unigene43137\_TAEpaeTARAAPEI-5, Unigene43623\_TAEpaeTARAAPEI-5, Unigene43947\_TAEpaeTARAAPEI-5, Unigene44034\_TAEpaeTARAAPEI-5, Unigene44258\_TAEpaeTARAAPEI-5, Unigene44522\_TAEpaeTARAAPEI-5, Unigene44725\_TAEpaeTARAAPEI-5, Unigene44777\_TAEpaeTARAAPEI-5, Unigene44905\_TAEpaeTARAAPEI-5, Unigene44928\_TAEpaeTARAAPEI-5, Unigene45351\_TAEpaeTARAAPEI-5, Unigene45433\_TAEpaeTARAAPEI-5, Unigene45455\_TAEpaeTARAAPEI-5, Unigene45493\_TAEpaeTARAAPEI-5, Unigene45577\_TAEpaeTARAAPEI-5, Unigene45636\_TAEpaeTARAAPEI-5, Unigene4594\_TAEpaeTARAAPEI-5, Unigene46328\_TAEpaeTARAAPEI-5, Unigene46530\_TAEpaeTARAAPEI-5, Unigene46682\_TAEpaeTARAAPEI-5, Unigene46911\_TAEpaeTARAAPEI-5, Unigene4692\_TAEpaeTARAAPEI-5, Unigene46962\_TAEpaeTARAAPEI-5, Unigene47214\_TAEpaeTARAAPEI-5, Unigene47239\_TAEpaeTARAAPEI-5, Unigene47372\_TAEpaeTARAAPEI-5, Unigene47525\_TAEpaeTARAAPEI-5, Unigene47608\_TAEpaeTARAAPEI-5, Unigene48176\_TAEpaeTARAAPEI-5, Unigene48287\_TAEpaeTARAAPEI-5, Unigene48445\_TAEpaeTARAAPEI-5, Unigene4845\_TAEpaeTARAAPEI-5, Unigene48734\_TAEpaeTARAAPEI-5, Unigene48744\_TAEpaeTARAAPEI-5, Unigene49141\_TAEpaeTARAAPEI-5, Unigene49181\_TAEpaeTARAAPEI-5, Unigene49211\_TAEpaeTARAAPEI-5, Unigene49219\_TAEpaeTARAAPEI-5, Unigene49327\_TAEpaeTARAAPEI-5, Unigene49826\_TAEpaeTARAAPEI-5, Unigene49867\_TAEpaeTARAAPEI-5, Unigene49952\_TAEpaeTARAAPEI-5, Unigene50088\_TAEpaeTARAAPEI-5, Unigene50104\_TAEpaeTARAAPEI-5, Unigene50742\_TAEpaeTARAAPEI-5, Unigene50753\_TAEpaeTARAAPEI-5, Unigene50809\_TAEpaeTARAAPEI-5, Unigene51029\_TAEpaeTARAAPEI-5, Unigene51150\_TAEpaeTARAAPEI-5, Unigene5121\_TAEpaeTARAAPEI-5, Unigene51223\_TAEpaeTARAAPEI-5, Unigene51311\_TAEpaeTARAAPEI-5, Unigene5143\_TAEpaeTARAAPEI-5, Unigene51561\_TAEpaeTARAAPEI-5, Unigene51772\_TAEpaeTARAAPEI-5, Unigene51901\_TAEpaeTARAAPEI-5, Unigene51907\_TAEpaeTARAAPEI-5, Unigene51977\_TAEpaeTARAAPEI-5, Unigene52012\_TAEpaeTARAAPEI-5, Unigene52429\_TAEpaeTARAAPEI-5, Unigene52457\_TAEpaeTARAAPEI-5, Unigene52492\_TAEpaeTARAAPEI-5, Unigene52535\_TAEpaeTARAAPEI-5, Unigene52548\_TAEpaeTARAAPEI-5, Unigene52566\_TAEpaeTARAAPEI-5, Unigene52781\_TAEpaeTARAAPEI-5, Unigene5284\_TAEpaeTARAAPEI-5, Unigene53004\_TAEpaeTARAAPEI-5, Unigene53081\_TAEpaeTARAAPEI-5, Unigene53161\_TAEpaeTARAAPEI-5, Unigene53182\_TAEpaeTARAAPEI-5, Unigene53263\_TAEpaeTARAAPEI-5, Unigene53512\_TAEpaeTARAAPEI-5, Unigene5364\_TAEpaeTARAAPEI-5, Unigene53667\_TAEpaeTARAAPEI-5, Unigene537\_TAEpaeTARAAPEI-5, Unigene53825\_TAEpaeTARAAPEI-5, Unigene53\_TAEpaeTARAAPEI-5, Unigene54035\_TAEpaeTARAAPEI-5, Unigene54180\_TAEpaeTARAAPEI-5, Unigene54298\_TAEpaeTARAAPEI-5, Unigene54427\_TAEpaeTARAAPEI-5, Unigene54447\_TAEpaeTARAAPEI-5, Unigene54541\_TAEpaeTARAAPEI-5, Unigene54593\_TAEpaeTARAAPEI-5, Unigene54639\_TAEpaeTARAAPEI-5, Unigene5465\_TAEpaeTARAAPEI-5, Unigene54880\_TAEpaeTARAAPEI-5, Unigene55000\_TAEpaeTARAAPEI-5, Unigene55037\_TAEpaeTARAAPEI-5, Unigene55154\_TAEpaeTARAAPEI-5, Unigene55226\_TAEpaeTARAAPEI-5, Unigene55372\_TAEpaeTARAAPEI-5, Unigene55389\_TAEpaeTARAAPEI-5, Unigene55419\_TAEpaeTARAAPEI-5, Unigene55554\_TAEpaeTARAAPEI-5, Unigene55705\_TAEpaeTARAAPEI-5, Unigene55826\_TAEpaeTARAAPEI-5, Unigene55954\_TAEpaeTARAAPEI-5, Unigene55998\_TAEpaeTARAAPEI-5, Unigene559\_TAEpaeTARAAPEI-5, Unigene56014\_TAEpaeTARAAPEI-5, Unigene56039\_TAEpaeTARAAPEI-5, Unigene56061\_TAEpaeTARAAPEI-5, Unigene56160\_TAEpaeTARAAPEI-5, Unigene56173\_TAEpaeTARAAPEI-5, Unigene56256\_TAEpaeTARAAPEI-5, Unigene56286\_TAEpaeTARAAPEI-5, Unigene56349\_TAEpaeTARAAPEI-5, Unigene56378\_TAEpaeTARAAPEI-5, Unigene56398\_TAEpaeTARAAPEI-5, Unigene5650\_TAEpaeTARAAPEI-5, Unigene56585\_TAEpaeTARAAPEI-5, Unigene56618\_TAEpaeTARAAPEI-5, Unigene56811\_TAEpaeTARAAPEI-5, Unigene57253\_TAEpaeTARAAPEI-5, Unigene57338\_TAEpaeTARAAPEI-5, Unigene57734\_TAEpaeTARAAPEI-5, Unigene57736\_TAEpaeTARAAPEI-5, Unigene58508\_TAEpaeTARAAPEI-5, Unigene58763\_TAEpaeTARAAPEI-5, Unigene58850\_TAEpaeTARAAPEI-5, Unigene58941\_TAEpaeTARAAPEI-5, Unigene59006\_TAEpaeTARAAPEI-5, Unigene59210\_TAEpaeTARAAPEI-5, Unigene59244\_TAEpaeTARAAPEI-5, Unigene59380\_TAEpaeTARAAPEI-5, Unigene59429\_TAEpaeTARAAPEI-5, Unigene59851\_TAEpaeTARAAPEI-5, Unigene5991\_TAEpaeTARAAPEI-5, Unigene60059\_TAEpaeTARAAPEI-5, Unigene60123\_TAEpaeTARAAPEI-5, Unigene60383\_TAEpaeTARAAPEI-5, Unigene60468\_TAEpaeTARAAPEI-5, Unigene60473\_TAEpaeTARAAPEI-5, Unigene60543\_TAEpaeTARAAPEI-5, Unigene60579\_TAEpaeTARAAPEI-5, Unigene60585\_TAEpaeTARAAPEI-5, Unigene6059\_TAEpaeTARAAPEI-5, Unigene6062\_TAEpaeTARAAPEI-5, Unigene60638\_TAEpaeTARAAPEI-5, Unigene60755\_TAEpaeTARAAPEI-5, Unigene60894\_TAEpaeTARAAPEI-5, Unigene60925\_TAEpaeTARAAPEI-5, Unigene60946\_TAEpaeTARAAPEI-5, Unigene61092\_TAEpaeTARAAPEI-5, Unigene6116\_TAEpaeTARAAPEI-5, Unigene61268\_TAEpaeTARAAPEI-5, Unigene61544\_TAEpaeTARAAPEI-5, Unigene6160\_TAEpaeTARAAPEI-5, Unigene61621\_TAEpaeTARAAPEI-5, Unigene61680\_TAEpaeTARAAPEI-5, Unigene61690\_TAEpaeTARAAPEI-5, Unigene61791\_TAEpaeTARAAPEI-5, Unigene61892\_TAEpaeTARAAPEI-5, Unigene61957\_TAEpaeTARAAPEI-5, Unigene62166\_TAEpaeTARAAPEI-5, Unigene62274\_TAEpaeTARAAPEI-5, Unigene62508\_TAEpaeTARAAPEI-5, Unigene62549\_TAEpaeTARAAPEI-5, Unigene62593\_TAEpaeTARAAPEI-5, Unigene62702\_TAEpaeTARAAPEI-5, Unigene62793\_TAEpaeTARAAPEI-5, Unigene62824\_TAEpaeTARAAPEI-5, Unigene6294\_TAEpaeTARAAPEI-5, Unigene62952\_TAEpaeTARAAPEI-5, Unigene62980\_TAEpaeTARAAPEI-5, Unigene62989\_TAEpaeTARAAPEI-5, Unigene63001\_TAEpaeTARAAPEI-5, Unigene63017\_TAEpaeTARAAPEI-5, Unigene63073\_TAEpaeTARAAPEI-5, Unigene63078\_TAEpaeTARAAPEI-5, Unigene63283\_TAEpaeTARAAPEI-5, Unigene63431\_TAEpaeTARAAPEI-5, Unigene63544\_TAEpaeTARAAPEI-5, Unigene63642\_TAEpaeTARAAPEI-5, Unigene63745\_TAEpaeTARAAPEI-5, Unigene63868\_TAEpaeTARAAPEI-5, Unigene63875\_TAEpaeTARAAPEI-5, Unigene64039\_TAEpaeTARAAPEI-5, Unigene64086\_TAEpaeTARAAPEI-5, Unigene64099\_TAEpaeTARAAPEI-5, Unigene64102\_TAEpaeTARAAPEI-5, Unigene64126\_TAEpaeTARAAPEI-5, Unigene64165\_TAEpaeTARAAPEI-5, Unigene64297\_TAEpaeTARAAPEI-5, Unigene64320\_TAEpaeTARAAPEI-5, Unigene64364\_TAEpaeTARAAPEI-5, Unigene64449\_TAEpaeTARAAPEI-5, Unigene64777\_TAEpaeTARAAPEI-5, Unigene64811\_TAEpaeTARAAPEI-5, Unigene64852\_TAEpaeTARAAPEI-5, Unigene64995\_TAEpaeTARAAPEI-5, Unigene65061\_TAEpaeTARAAPEI-5, Unigene65165\_TAEpaeTARAAPEI-5, Unigene65170\_TAEpaeTARAAPEI-5, Unigene65292\_TAEpaeTARAAPEI-5, Unigene65324\_TAEpaeTARAAPEI-5, Unigene65343\_TAEpaeTARAAPEI-5, Unigene6545\_TAEpaeTARAAPEI-5, Unigene65518\_TAEpaeTARAAPEI-5, Unigene65634\_TAEpaeTARAAPEI-5, Unigene65657\_TAEpaeTARAAPEI-5, Unigene65685\_TAEpaeTARAAPEI-5, Unigene65757\_TAEpaeTARAAPEI-5, Unigene66071\_TAEpaeTARAAPEI-5, Unigene66161\_TAEpaeTARAAPEI-5, Unigene66201\_TAEpaeTARAAPEI-5, Unigene66206\_TAEpaeTARAAPEI-5, Unigene66235\_TAEpaeTARAAPEI-5, Unigene66271\_TAEpaeTARAAPEI-5, Unigene66307\_TAEpaeTARAAPEI-5, Unigene66336\_TAEpaeTARAAPEI-5, Unigene66427\_TAEpaeTARAAPEI-5, Unigene66562\_TAEpaeTARAAPEI-5, Unigene66625\_TAEpaeTARAAPEI-5, Unigene66807\_TAEpaeTARAAPEI-5, Unigene66855\_TAEpaeTARAAPEI-5, Unigene66858\_TAEpaeTARAAPEI-5, Unigene66864\_TAEpaeTARAAPEI-5, Unigene66877\_TAEpaeTARAAPEI-5, Unigene66902\_TAEpaeTARAAPEI-5, Unigene66929\_TAEpaeTARAAPEI-5, Unigene66934\_TAEpaeTARAAPEI-5, Unigene67068\_TAEpaeTARAAPEI-5, Unigene67159\_TAEpaeTARAAPEI-5, Unigene67192\_TAEpaeTARAAPEI-5, Unigene67280\_TAEpaeTARAAPEI-5, Unigene67346\_TAEpaeTARAAPEI-5, Unigene67376\_TAEpaeTARAAPEI-5, Unigene67459\_TAEpaeTARAAPEI-5, Unigene67480\_TAEpaeTARAAPEI-5, Unigene67550\_TAEpaeTARAAPEI-5, Unigene67597\_TAEpaeTARAAPEI-5, Unigene67733\_TAEpaeTARAAPEI-5, Unigene67835\_TAEpaeTARAAPEI-5, Unigene67861\_TAEpaeTARAAPEI-5, Unigene67903\_TAEpaeTARAAPEI-5, Unigene68018\_TAEpaeTARAAPEI-5, Unigene68029\_TAEpaeTARAAPEI-5, Unigene68033\_TAEpaeTARAAPEI-5, Unigene68076\_TAEpaeTARAAPEI-5, Unigene68125\_TAEpaeTARAAPEI-5, Unigene68139\_TAEpaeTARAAPEI-5, Unigene68156\_TAEpaeTARAAPEI-5, Unigene68189\_TAEpaeTARAAPEI-5, Unigene68207\_TAEpaeTARAAPEI-5, Unigene68242\_TAEpaeTARAAPEI-5, Unigene68316\_TAEpaeTARAAPEI-5, Unigene68346\_TAEpaeTARAAPEI-5, Unigene68390\_TAEpaeTARAAPEI-5, Unigene68502\_TAEpaeTARAAPEI-5, Unigene68564\_TAEpaeTARAAPEI-5, Unigene68619\_TAEpaeTARAAPEI-5, Unigene68739\_TAEpaeTARAAPEI-5, Unigene68903\_TAEpaeTARAAPEI-5, Unigene69044\_TAEpaeTARAAPEI-5, Unigene69086\_TAEpaeTARAAPEI-5, Unigene69146\_TAEpaeTARAAPEI-5, Unigene69179\_TAEpaeTARAAPEI-5, Unigene69199\_TAEpaeTARAAPEI-5, Unigene69227\_TAEpaeTARAAPEI-5, Unigene69285\_TAEpaeTARAAPEI-5, Unigene69309\_TAEpaeTARAAPEI-5, Unigene69316\_TAEpaeTARAAPEI-5, Unigene69403\_TAEpaeTARAAPEI-5, Unigene69494\_TAEpaeTARAAPEI-5, Unigene69562\_TAEpaeTARAAPEI-5, Unigene69607\_TAEpaeTARAAPEI-5, Unigene69681\_TAEpaeTARAAPEI-5, Unigene69792\_TAEpaeTARAAPEI-5, Unigene69819\_TAEpaeTARAAPEI-5, Unigene69914\_TAEpaeTARAAPEI-5, Unigene69975\_TAEpaeTARAAPEI-5, Unigene70112\_TAEpaeTARAAPEI-5, Unigene70156\_TAEpaeTARAAPEI-5, Unigene70317\_TAEpaeTARAAPEI-5, Unigene70390\_TAEpaeTARAAPEI-5, Unigene70399\_TAEpaeTARAAPEI-5, Unigene7045\_TAEpaeTARAAPEI-5, Unigene70495\_TAEpaeTARAAPEI-5, Unigene70510\_TAEpaeTARAAPEI-5, Unigene70521\_TAEpaeTARAAPEI-5, Unigene7057\_TAEpaeTARAAPEI-5, Unigene70598\_TAEpaeTARAAPEI-5, Unigene70664\_TAEpaeTARAAPEI-5, Unigene70756\_TAEpaeTARAAPEI-5, Unigene70792\_TAEpaeTARAAPEI-5, Unigene70795\_TAEpaeTARAAPEI-5, Unigene70832\_TAEpaeTARAAPEI-5, Unigene70863\_TAEpaeTARAAPEI-5, Unigene70909\_TAEpaeTARAAPEI-5, Unigene70979\_TAEpaeTARAAPEI-5, Unigene71062\_TAEpaeTARAAPEI-5, Unigene71078\_TAEpaeTARAAPEI-5, Unigene71091\_TAEpaeTARAAPEI-5, Unigene7114\_TAEpaeTARAAPEI-5, Unigene71162\_TAEpaeTARAAPEI-5, Unigene71180\_TAEpaeTARAAPEI-5, Unigene71205\_TAEpaeTARAAPEI-5, Unigene71206\_TAEpaeTARAAPEI-5, Unigene71214\_TAEpaeTARAAPEI-5, Unigene71230\_TAEpaeTARAAPEI-5, Unigene71238\_TAEpaeTARAAPEI-5, Unigene71340\_TAEpaeTARAAPEI-5, Unigene71426\_TAEpaeTARAAPEI-5, Unigene71461\_TAEpaeTARAAPEI-5, Unigene71498\_TAEpaeTARAAPEI-5, Unigene71516\_TAEpaeTARAAPEI-5, Unigene71535\_TAEpaeTARAAPEI-5, Unigene71565\_TAEpaeTARAAPEI-5, Unigene71587\_TAEpaeTARAAPEI-5, Unigene71592\_TAEpaeTARAAPEI-5, Unigene71606\_TAEpaeTARAAPEI-5, Unigene7163\_TAEpaeTARAAPEI-5, Unigene71641\_TAEpaeTARAAPEI-5, Unigene71674\_TAEpaeTARAAPEI-5, Unigene71682\_TAEpaeTARAAPEI-5, Unigene71691\_TAEpaeTARAAPEI-5, Unigene71773\_TAEpaeTARAAPEI-5, Unigene71788\_TAEpaeTARAAPEI-5, Unigene71797\_TAEpaeTARAAPEI-5, Unigene71799\_TAEpaeTARAAPEI-5, Unigene71822\_TAEpaeTARAAPEI-5, Unigene71857\_TAEpaeTARAAPEI-5, Unigene7199\_TAEpaeTARAAPEI-5, Unigene72140\_TAEpaeTARAAPEI-5, Unigene72149\_TAEpaeTARAAPEI-5, Unigene72323\_TAEpaeTARAAPEI-5, Unigene72340\_TAEpaeTARAAPEI-5, Unigene72347\_TAEpaeTARAAPEI-5, Unigene7237\_TAEpaeTARAAPEI-5, Unigene72399\_TAEpaeTARAAPEI-5, Unigene72401\_TAEpaeTARAAPEI-5, Unigene72458\_TAEpaeTARAAPEI-5, Unigene72472\_TAEpaeTARAAPEI-5, Unigene72500\_TAEpaeTARAAPEI-5, Unigene72520\_TAEpaeTARAAPEI-5, Unigene72541\_TAEpaeTARAAPEI-5, Unigene72576\_TAEpaeTARAAPEI-5, Unigene72599\_TAEpaeTARAAPEI-5, Unigene72675\_TAEpaeTARAAPEI-5, Unigene72705\_TAEpaeTARAAPEI-5, Unigene72709\_TAEpaeTARAAPEI-5, Unigene72786\_TAEpaeTARAAPEI-5, Unigene72816\_TAEpaeTARAAPEI-5, Unigene72900\_TAEpaeTARAAPEI-5, Unigene72926\_TAEpaeTARAAPEI-5, Unigene72938\_TAEpaeTARAAPEI-5, Unigene7418\_TAEpaeTARAAPEI-5, Unigene7456\_TAEpaeTARAAPEI-5, Unigene7574\_TAEpaeTARAAPEI-5, Unigene75\_TAEpaeTARAAPEI-5, Unigene7685\_TAEpaeTARAAPEI-5, Unigene7692\_TAEpaeTARAAPEI-5, Unigene7724\_TAEpaeTARAAPEI-5, Unigene7781\_TAEpaeTARAAPEI-5, Unigene8016\_TAEpaeTARAAPEI-5, Unigene8049\_TAEpaeTARAAPEI-5, Unigene8078\_TAEpaeTARAAPEI-5, Unigene8192\_TAEpaeTARAAPEI-5, Unigene8206\_TAEpaeTARAAPEI-5, Unigene8323\_TAEpaeTARAAPEI-5, Unigene858\_TAEpaeTARAAPEI-5, Unigene8797\_TAEpaeTARAAPEI-5, Unigene8840\_TAEpaeTARAAPEI-5, Unigene8862\_TAEpaeTARAAPEI-5, Unigene8873\_TAEpaeTARAAPEI-5, Unigene8878\_TAEpaeTARAAPEI-5, Unigene8993\_TAEpaeTARAAPEI-5, Unigene9049\_TAEpaeTARAAPEI-5, Unigene907\_TAEpaeTARAAPEI-5, Unigene9113\_TAEpaeTARAAPEI-5, Unigene9181\_TAEpaeTARAAPEI-5, Unigene9451\_TAEpaeTARAAPEI-5, Unigene9554\_TAEpaeTARAAPEI-5, Unigene9738\_TAEpaeTARAAPEI-5, Unigene9767\_TAEpaeTARAAPEI-5, Unigene9794\_TAEpaeTARAAPEI-5, Unigene9941\_TAEpaeTARAAPEI-5 |
| 5 | Focal adhesion | Unigene10142\_TAEpaeTARAAPEI-5, Unigene10169\_TAEpaeTARAAPEI-5, Unigene10250\_TAEpaeTARAAPEI-5, Unigene10441\_TAEpaeTARAAPEI-5, Unigene10474\_TAEpaeTARAAPEI-5, Unigene10547\_TAEpaeTARAAPEI-5, Unigene10616\_TAEpaeTARAAPEI-5, Unigene10716\_TAEpaeTARAAPEI-5, Unigene10720\_TAEpaeTARAAPEI-5, Unigene10848\_TAEpaeTARAAPEI-5, Unigene11238\_TAEpaeTARAAPEI-5, Unigene11268\_TAEpaeTARAAPEI-5, Unigene11397\_TAEpaeTARAAPEI-5, Unigene11534\_TAEpaeTARAAPEI-5, Unigene11551\_TAEpaeTARAAPEI-5, Unigene11610\_TAEpaeTARAAPEI-5, Unigene11645\_TAEpaeTARAAPEI-5, Unigene11874\_TAEpaeTARAAPEI-5, Unigene12059\_TAEpaeTARAAPEI-5, Unigene12159\_TAEpaeTARAAPEI-5, Unigene12165\_TAEpaeTARAAPEI-5, Unigene12167\_TAEpaeTARAAPEI-5, Unigene12947\_TAEpaeTARAAPEI-5, Unigene12955\_TAEpaeTARAAPEI-5, Unigene12956\_TAEpaeTARAAPEI-5, Unigene12994\_TAEpaeTARAAPEI-5, Unigene13091\_TAEpaeTARAAPEI-5, Unigene13451\_TAEpaeTARAAPEI-5, Unigene13629\_TAEpaeTARAAPEI-5, Unigene13650\_TAEpaeTARAAPEI-5, Unigene13666\_TAEpaeTARAAPEI-5, Unigene13747\_TAEpaeTARAAPEI-5, Unigene13880\_TAEpaeTARAAPEI-5, Unigene14178\_TAEpaeTARAAPEI-5, Unigene14270\_TAEpaeTARAAPEI-5, Unigene1433\_TAEpaeTARAAPEI-5, Unigene14366\_TAEpaeTARAAPEI-5, Unigene14369\_TAEpaeTARAAPEI-5, Unigene14384\_TAEpaeTARAAPEI-5, Unigene14714\_TAEpaeTARAAPEI-5, Unigene14809\_TAEpaeTARAAPEI-5, Unigene15053\_TAEpaeTARAAPEI-5, Unigene15107\_TAEpaeTARAAPEI-5, Unigene15247\_TAEpaeTARAAPEI-5, Unigene15468\_TAEpaeTARAAPEI-5, Unigene15730\_TAEpaeTARAAPEI-5, Unigene16204\_TAEpaeTARAAPEI-5, Unigene163\_TAEpaeTARAAPEI-5, Unigene16468\_TAEpaeTARAAPEI-5, Unigene16509\_TAEpaeTARAAPEI-5, Unigene16524\_TAEpaeTARAAPEI-5, Unigene16824\_TAEpaeTARAAPEI-5, Unigene17034\_TAEpaeTARAAPEI-5, Unigene1712\_TAEpaeTARAAPEI-5, Unigene17145\_TAEpaeTARAAPEI-5, Unigene17208\_TAEpaeTARAAPEI-5, Unigene17282\_TAEpaeTARAAPEI-5, Unigene17364\_TAEpaeTARAAPEI-5, Unigene17393\_TAEpaeTARAAPEI-5, Unigene17397\_TAEpaeTARAAPEI-5, Unigene17475\_TAEpaeTARAAPEI-5, Unigene17535\_TAEpaeTARAAPEI-5, Unigene17569\_TAEpaeTARAAPEI-5, Unigene17641\_TAEpaeTARAAPEI-5, Unigene17657\_TAEpaeTARAAPEI-5, Unigene17673\_TAEpaeTARAAPEI-5, Unigene17700\_TAEpaeTARAAPEI-5, Unigene17839\_TAEpaeTARAAPEI-5, Unigene17970\_TAEpaeTARAAPEI-5, Unigene18309\_TAEpaeTARAAPEI-5, Unigene19456\_TAEpaeTARAAPEI-5, Unigene19465\_TAEpaeTARAAPEI-5, Unigene19554\_TAEpaeTARAAPEI-5, Unigene19671\_TAEpaeTARAAPEI-5, Unigene19736\_TAEpaeTARAAPEI-5, Unigene19746\_TAEpaeTARAAPEI-5, Unigene19996\_TAEpaeTARAAPEI-5, Unigene20000\_TAEpaeTARAAPEI-5, Unigene20837\_TAEpaeTARAAPEI-5, Unigene21298\_TAEpaeTARAAPEI-5, Unigene21363\_TAEpaeTARAAPEI-5, Unigene21425\_TAEpaeTARAAPEI-5, Unigene21513\_TAEpaeTARAAPEI-5, Unigene21544\_TAEpaeTARAAPEI-5, Unigene21875\_TAEpaeTARAAPEI-5, Unigene21962\_TAEpaeTARAAPEI-5, Unigene22032\_TAEpaeTARAAPEI-5, Unigene2206\_TAEpaeTARAAPEI-5, Unigene22256\_TAEpaeTARAAPEI-5, Unigene22272\_TAEpaeTARAAPEI-5, Unigene22280\_TAEpaeTARAAPEI-5, Unigene22358\_TAEpaeTARAAPEI-5, Unigene2241\_TAEpaeTARAAPEI-5, Unigene22482\_TAEpaeTARAAPEI-5, Unigene22499\_TAEpaeTARAAPEI-5, Unigene22689\_TAEpaeTARAAPEI-5, Unigene22762\_TAEpaeTARAAPEI-5, Unigene22842\_TAEpaeTARAAPEI-5, Unigene23077\_TAEpaeTARAAPEI-5, Unigene23406\_TAEpaeTARAAPEI-5, Unigene23485\_TAEpaeTARAAPEI-5, Unigene2360\_TAEpaeTARAAPEI-5, Unigene23907\_TAEpaeTARAAPEI-5, Unigene24012\_TAEpaeTARAAPEI-5, Unigene2429\_TAEpaeTARAAPEI-5, Unigene24352\_TAEpaeTARAAPEI-5, Unigene2439\_TAEpaeTARAAPEI-5, Unigene24538\_TAEpaeTARAAPEI-5, Unigene24657\_TAEpaeTARAAPEI-5, Unigene24931\_TAEpaeTARAAPEI-5, Unigene2508\_TAEpaeTARAAPEI-5, Unigene2562\_TAEpaeTARAAPEI-5, Unigene25710\_TAEpaeTARAAPEI-5, Unigene25859\_TAEpaeTARAAPEI-5, Unigene25921\_TAEpaeTARAAPEI-5, Unigene26033\_TAEpaeTARAAPEI-5, Unigene26068\_TAEpaeTARAAPEI-5, Unigene26309\_TAEpaeTARAAPEI-5, Unigene26359\_TAEpaeTARAAPEI-5, Unigene26859\_TAEpaeTARAAPEI-5, Unigene26907\_TAEpaeTARAAPEI-5, Unigene27023\_TAEpaeTARAAPEI-5, Unigene27095\_TAEpaeTARAAPEI-5, Unigene27154\_TAEpaeTARAAPEI-5, Unigene27972\_TAEpaeTARAAPEI-5, Unigene28135\_TAEpaeTARAAPEI-5, Unigene28371\_TAEpaeTARAAPEI-5, Unigene28476\_TAEpaeTARAAPEI-5, Unigene28785\_TAEpaeTARAAPEI-5, Unigene2882\_TAEpaeTARAAPEI-5, Unigene2889\_TAEpaeTARAAPEI-5, Unigene28987\_TAEpaeTARAAPEI-5, Unigene29088\_TAEpaeTARAAPEI-5, Unigene29203\_TAEpaeTARAAPEI-5, Unigene29211\_TAEpaeTARAAPEI-5, Unigene29363\_TAEpaeTARAAPEI-5, Unigene29499\_TAEpaeTARAAPEI-5, Unigene29532\_TAEpaeTARAAPEI-5, Unigene29616\_TAEpaeTARAAPEI-5, Unigene29626\_TAEpaeTARAAPEI-5, Unigene29718\_TAEpaeTARAAPEI-5, Unigene29739\_TAEpaeTARAAPEI-5, Unigene29900\_TAEpaeTARAAPEI-5, Unigene30199\_TAEpaeTARAAPEI-5, Unigene301\_TAEpaeTARAAPEI-5, Unigene30361\_TAEpaeTARAAPEI-5, Unigene30672\_TAEpaeTARAAPEI-5, Unigene30843\_TAEpaeTARAAPEI-5, Unigene308\_TAEpaeTARAAPEI-5, Unigene31043\_TAEpaeTARAAPEI-5, Unigene31109\_TAEpaeTARAAPEI-5, Unigene31209\_TAEpaeTARAAPEI-5, Unigene3145\_TAEpaeTARAAPEI-5, Unigene31705\_TAEpaeTARAAPEI-5, Unigene31877\_TAEpaeTARAAPEI-5, Unigene32040\_TAEpaeTARAAPEI-5, Unigene32081\_TAEpaeTARAAPEI-5, Unigene32104\_TAEpaeTARAAPEI-5, Unigene32212\_TAEpaeTARAAPEI-5, Unigene32215\_TAEpaeTARAAPEI-5, Unigene32680\_TAEpaeTARAAPEI-5, Unigene32844\_TAEpaeTARAAPEI-5, Unigene32999\_TAEpaeTARAAPEI-5, Unigene33030\_TAEpaeTARAAPEI-5, Unigene33057\_TAEpaeTARAAPEI-5, Unigene33125\_TAEpaeTARAAPEI-5, Unigene33174\_TAEpaeTARAAPEI-5, Unigene33204\_TAEpaeTARAAPEI-5, Unigene33255\_TAEpaeTARAAPEI-5, Unigene33301\_TAEpaeTARAAPEI-5, Unigene33609\_TAEpaeTARAAPEI-5, Unigene33696\_TAEpaeTARAAPEI-5, Unigene33986\_TAEpaeTARAAPEI-5, Unigene34195\_TAEpaeTARAAPEI-5, Unigene34303\_TAEpaeTARAAPEI-5, Unigene34344\_TAEpaeTARAAPEI-5, Unigene35102\_TAEpaeTARAAPEI-5, Unigene35733\_TAEpaeTARAAPEI-5, Unigene35839\_TAEpaeTARAAPEI-5, Unigene35934\_TAEpaeTARAAPEI-5, Unigene36128\_TAEpaeTARAAPEI-5, Unigene36136\_TAEpaeTARAAPEI-5, Unigene36447\_TAEpaeTARAAPEI-5, Unigene36453\_TAEpaeTARAAPEI-5, Unigene36648\_TAEpaeTARAAPEI-5, Unigene36847\_TAEpaeTARAAPEI-5, Unigene36984\_TAEpaeTARAAPEI-5, Unigene37038\_TAEpaeTARAAPEI-5, Unigene37112\_TAEpaeTARAAPEI-5, Unigene37318\_TAEpaeTARAAPEI-5, Unigene37328\_TAEpaeTARAAPEI-5, Unigene37368\_TAEpaeTARAAPEI-5, Unigene37500\_TAEpaeTARAAPEI-5, Unigene37692\_TAEpaeTARAAPEI-5, Unigene37980\_TAEpaeTARAAPEI-5, Unigene37986\_TAEpaeTARAAPEI-5, Unigene38129\_TAEpaeTARAAPEI-5, Unigene38160\_TAEpaeTARAAPEI-5, Unigene38258\_TAEpaeTARAAPEI-5, Unigene38545\_TAEpaeTARAAPEI-5, Unigene38660\_TAEpaeTARAAPEI-5, Unigene38949\_TAEpaeTARAAPEI-5, Unigene39224\_TAEpaeTARAAPEI-5, Unigene39227\_TAEpaeTARAAPEI-5, Unigene39295\_TAEpaeTARAAPEI-5, Unigene39378\_TAEpaeTARAAPEI-5, Unigene39407\_TAEpaeTARAAPEI-5, Unigene3949\_TAEpaeTARAAPEI-5, Unigene39874\_TAEpaeTARAAPEI-5, Unigene39926\_TAEpaeTARAAPEI-5, Unigene40344\_TAEpaeTARAAPEI-5, Unigene40566\_TAEpaeTARAAPEI-5, Unigene40763\_TAEpaeTARAAPEI-5, Unigene40775\_TAEpaeTARAAPEI-5, Unigene40826\_TAEpaeTARAAPEI-5, Unigene40856\_TAEpaeTARAAPEI-5, Unigene40999\_TAEpaeTARAAPEI-5, Unigene4154\_TAEpaeTARAAPEI-5, Unigene41673\_TAEpaeTARAAPEI-5, Unigene41794\_TAEpaeTARAAPEI-5, Unigene42195\_TAEpaeTARAAPEI-5, Unigene42882\_TAEpaeTARAAPEI-5, Unigene43003\_TAEpaeTARAAPEI-5, Unigene43026\_TAEpaeTARAAPEI-5, Unigene43076\_TAEpaeTARAAPEI-5, Unigene43158\_TAEpaeTARAAPEI-5, Unigene43310\_TAEpaeTARAAPEI-5, Unigene43326\_TAEpaeTARAAPEI-5, Unigene43451\_TAEpaeTARAAPEI-5, Unigene43696\_TAEpaeTARAAPEI-5, Unigene4371\_TAEpaeTARAAPEI-5, Unigene4380\_TAEpaeTARAAPEI-5, Unigene43842\_TAEpaeTARAAPEI-5, Unigene43850\_TAEpaeTARAAPEI-5, Unigene44056\_TAEpaeTARAAPEI-5, Unigene44210\_TAEpaeTARAAPEI-5, Unigene44287\_TAEpaeTARAAPEI-5, Unigene44401\_TAEpaeTARAAPEI-5, Unigene44491\_TAEpaeTARAAPEI-5, Unigene44523\_TAEpaeTARAAPEI-5, Unigene44643\_TAEpaeTARAAPEI-5, Unigene44682\_TAEpaeTARAAPEI-5, Unigene44963\_TAEpaeTARAAPEI-5, Unigene45049\_TAEpaeTARAAPEI-5, Unigene45180\_TAEpaeTARAAPEI-5, Unigene45449\_TAEpaeTARAAPEI-5, Unigene45468\_TAEpaeTARAAPEI-5, Unigene45534\_TAEpaeTARAAPEI-5, Unigene45602\_TAEpaeTARAAPEI-5, Unigene45803\_TAEpaeTARAAPEI-5, Unigene46118\_TAEpaeTARAAPEI-5, Unigene46174\_TAEpaeTARAAPEI-5, Unigene46290\_TAEpaeTARAAPEI-5, Unigene46349\_TAEpaeTARAAPEI-5, Unigene46659\_TAEpaeTARAAPEI-5, Unigene46947\_TAEpaeTARAAPEI-5, Unigene46978\_TAEpaeTARAAPEI-5, Unigene46995\_TAEpaeTARAAPEI-5, Unigene47023\_TAEpaeTARAAPEI-5, Unigene47314\_TAEpaeTARAAPEI-5, Unigene47449\_TAEpaeTARAAPEI-5, Unigene47575\_TAEpaeTARAAPEI-5, Unigene47595\_TAEpaeTARAAPEI-5, Unigene47898\_TAEpaeTARAAPEI-5, Unigene4800\_TAEpaeTARAAPEI-5, Unigene48018\_TAEpaeTARAAPEI-5, Unigene480\_TAEpaeTARAAPEI-5, Unigene48256\_TAEpaeTARAAPEI-5, Unigene48267\_TAEpaeTARAAPEI-5, Unigene48353\_TAEpaeTARAAPEI-5, Unigene48377\_TAEpaeTARAAPEI-5, Unigene48394\_TAEpaeTARAAPEI-5, Unigene48467\_TAEpaeTARAAPEI-5, Unigene48636\_TAEpaeTARAAPEI-5, Unigene48699\_TAEpaeTARAAPEI-5, Unigene48705\_TAEpaeTARAAPEI-5, Unigene48725\_TAEpaeTARAAPEI-5, Unigene48762\_TAEpaeTARAAPEI-5, Unigene48845\_TAEpaeTARAAPEI-5, Unigene49023\_TAEpaeTARAAPEI-5, Unigene49085\_TAEpaeTARAAPEI-5, Unigene49089\_TAEpaeTARAAPEI-5, Unigene49090\_TAEpaeTARAAPEI-5, Unigene49173\_TAEpaeTARAAPEI-5, Unigene49598\_TAEpaeTARAAPEI-5, Unigene49657\_TAEpaeTARAAPEI-5, Unigene49990\_TAEpaeTARAAPEI-5, Unigene50336\_TAEpaeTARAAPEI-5, Unigene50359\_TAEpaeTARAAPEI-5, Unigene50455\_TAEpaeTARAAPEI-5, Unigene50474\_TAEpaeTARAAPEI-5, Unigene50477\_TAEpaeTARAAPEI-5, Unigene50512\_TAEpaeTARAAPEI-5, Unigene50566\_TAEpaeTARAAPEI-5, Unigene50589\_TAEpaeTARAAPEI-5, Unigene50937\_TAEpaeTARAAPEI-5, Unigene5107\_TAEpaeTARAAPEI-5, Unigene51205\_TAEpaeTARAAPEI-5, Unigene51214\_TAEpaeTARAAPEI-5, Unigene51234\_TAEpaeTARAAPEI-5, Unigene51253\_TAEpaeTARAAPEI-5, Unigene5141\_TAEpaeTARAAPEI-5, Unigene51601\_TAEpaeTARAAPEI-5, Unigene51659\_TAEpaeTARAAPEI-5, Unigene51722\_TAEpaeTARAAPEI-5, Unigene5194\_TAEpaeTARAAPEI-5, Unigene52160\_TAEpaeTARAAPEI-5, Unigene52306\_TAEpaeTARAAPEI-5, Unigene52312\_TAEpaeTARAAPEI-5, Unigene52327\_TAEpaeTARAAPEI-5, Unigene52351\_TAEpaeTARAAPEI-5, Unigene52394\_TAEpaeTARAAPEI-5, Unigene5248\_TAEpaeTARAAPEI-5, Unigene52813\_TAEpaeTARAAPEI-5, Unigene52844\_TAEpaeTARAAPEI-5, Unigene52901\_TAEpaeTARAAPEI-5, Unigene53074\_TAEpaeTARAAPEI-5, Unigene53131\_TAEpaeTARAAPEI-5, Unigene53203\_TAEpaeTARAAPEI-5, Unigene53319\_TAEpaeTARAAPEI-5, Unigene53501\_TAEpaeTARAAPEI-5, Unigene53773\_TAEpaeTARAAPEI-5, Unigene53786\_TAEpaeTARAAPEI-5, Unigene53790\_TAEpaeTARAAPEI-5, Unigene53877\_TAEpaeTARAAPEI-5, Unigene53911\_TAEpaeTARAAPEI-5, Unigene54005\_TAEpaeTARAAPEI-5, Unigene54029\_TAEpaeTARAAPEI-5, Unigene54161\_TAEpaeTARAAPEI-5, Unigene54206\_TAEpaeTARAAPEI-5, Unigene54359\_TAEpaeTARAAPEI-5, Unigene54363\_TAEpaeTARAAPEI-5, Unigene54425\_TAEpaeTARAAPEI-5, Unigene54441\_TAEpaeTARAAPEI-5, Unigene54495\_TAEpaeTARAAPEI-5, Unigene54630\_TAEpaeTARAAPEI-5, Unigene54767\_TAEpaeTARAAPEI-5, Unigene54927\_TAEpaeTARAAPEI-5, Unigene55111\_TAEpaeTARAAPEI-5, Unigene55704\_TAEpaeTARAAPEI-5, Unigene55802\_TAEpaeTARAAPEI-5, Unigene55862\_TAEpaeTARAAPEI-5, Unigene55946\_TAEpaeTARAAPEI-5, Unigene56001\_TAEpaeTARAAPEI-5, Unigene56020\_TAEpaeTARAAPEI-5, Unigene56240\_TAEpaeTARAAPEI-5, Unigene56332\_TAEpaeTARAAPEI-5, Unigene56444\_TAEpaeTARAAPEI-5, Unigene56860\_TAEpaeTARAAPEI-5, Unigene56925\_TAEpaeTARAAPEI-5, Unigene5694\_TAEpaeTARAAPEI-5, Unigene56953\_TAEpaeTARAAPEI-5, Unigene57076\_TAEpaeTARAAPEI-5, Unigene57095\_TAEpaeTARAAPEI-5, Unigene57151\_TAEpaeTARAAPEI-5, Unigene57161\_TAEpaeTARAAPEI-5, Unigene57318\_TAEpaeTARAAPEI-5, Unigene57482\_TAEpaeTARAAPEI-5, Unigene57497\_TAEpaeTARAAPEI-5, Unigene57522\_TAEpaeTARAAPEI-5, Unigene57536\_TAEpaeTARAAPEI-5, Unigene5753\_TAEpaeTARAAPEI-5, Unigene57575\_TAEpaeTARAAPEI-5, Unigene57607\_TAEpaeTARAAPEI-5, Unigene57610\_TAEpaeTARAAPEI-5, Unigene57683\_TAEpaeTARAAPEI-5, Unigene57790\_TAEpaeTARAAPEI-5, Unigene57969\_TAEpaeTARAAPEI-5, Unigene57975\_TAEpaeTARAAPEI-5, Unigene58052\_TAEpaeTARAAPEI-5, Unigene58098\_TAEpaeTARAAPEI-5, Unigene58215\_TAEpaeTARAAPEI-5, Unigene58332\_TAEpaeTARAAPEI-5, Unigene58514\_TAEpaeTARAAPEI-5, Unigene58782\_TAEpaeTARAAPEI-5, Unigene58848\_TAEpaeTARAAPEI-5, Unigene58938\_TAEpaeTARAAPEI-5, Unigene59185\_TAEpaeTARAAPEI-5, Unigene59280\_TAEpaeTARAAPEI-5, Unigene59325\_TAEpaeTARAAPEI-5, Unigene59373\_TAEpaeTARAAPEI-5, Unigene59578\_TAEpaeTARAAPEI-5, Unigene59596\_TAEpaeTARAAPEI-5, Unigene59648\_TAEpaeTARAAPEI-5, Unigene5970\_TAEpaeTARAAPEI-5, Unigene60011\_TAEpaeTARAAPEI-5, Unigene60200\_TAEpaeTARAAPEI-5, Unigene60345\_TAEpaeTARAAPEI-5, Unigene60360\_TAEpaeTARAAPEI-5, Unigene60374\_TAEpaeTARAAPEI-5, Unigene60561\_TAEpaeTARAAPEI-5, Unigene60686\_TAEpaeTARAAPEI-5, Unigene60983\_TAEpaeTARAAPEI-5, Unigene61029\_TAEpaeTARAAPEI-5, Unigene61115\_TAEpaeTARAAPEI-5, Unigene61230\_TAEpaeTARAAPEI-5, Unigene61381\_TAEpaeTARAAPEI-5, Unigene61561\_TAEpaeTARAAPEI-5, Unigene61902\_TAEpaeTARAAPEI-5, Unigene62072\_TAEpaeTARAAPEI-5, Unigene62127\_TAEpaeTARAAPEI-5, Unigene62275\_TAEpaeTARAAPEI-5, Unigene62347\_TAEpaeTARAAPEI-5, Unigene6244\_TAEpaeTARAAPEI-5, Unigene6262\_TAEpaeTARAAPEI-5, Unigene62653\_TAEpaeTARAAPEI-5, Unigene62673\_TAEpaeTARAAPEI-5, Unigene62723\_TAEpaeTARAAPEI-5, Unigene62920\_TAEpaeTARAAPEI-5, Unigene62924\_TAEpaeTARAAPEI-5, Unigene62944\_TAEpaeTARAAPEI-5, Unigene62962\_TAEpaeTARAAPEI-5, Unigene63067\_TAEpaeTARAAPEI-5, Unigene63105\_TAEpaeTARAAPEI-5, Unigene63172\_TAEpaeTARAAPEI-5, Unigene63227\_TAEpaeTARAAPEI-5, Unigene63241\_TAEpaeTARAAPEI-5, Unigene63253\_TAEpaeTARAAPEI-5, Unigene63323\_TAEpaeTARAAPEI-5, Unigene63370\_TAEpaeTARAAPEI-5, Unigene63468\_TAEpaeTARAAPEI-5, Unigene63705\_TAEpaeTARAAPEI-5, Unigene6372\_TAEpaeTARAAPEI-5, Unigene63733\_TAEpaeTARAAPEI-5, Unigene63743\_TAEpaeTARAAPEI-5, Unigene63809\_TAEpaeTARAAPEI-5, Unigene63960\_TAEpaeTARAAPEI-5, Unigene63980\_TAEpaeTARAAPEI-5, Unigene64074\_TAEpaeTARAAPEI-5, Unigene64122\_TAEpaeTARAAPEI-5, Unigene64150\_TAEpaeTARAAPEI-5, Unigene64253\_TAEpaeTARAAPEI-5, Unigene64293\_TAEpaeTARAAPEI-5, Unigene64398\_TAEpaeTARAAPEI-5, Unigene6452\_TAEpaeTARAAPEI-5, Unigene64580\_TAEpaeTARAAPEI-5, Unigene64618\_TAEpaeTARAAPEI-5, Unigene64658\_TAEpaeTARAAPEI-5, Unigene64687\_TAEpaeTARAAPEI-5, Unigene65043\_TAEpaeTARAAPEI-5, Unigene65153\_TAEpaeTARAAPEI-5, Unigene65293\_TAEpaeTARAAPEI-5, Unigene65296\_TAEpaeTARAAPEI-5, Unigene65468\_TAEpaeTARAAPEI-5, Unigene65502\_TAEpaeTARAAPEI-5, Unigene65717\_TAEpaeTARAAPEI-5, Unigene65902\_TAEpaeTARAAPEI-5, Unigene65983\_TAEpaeTARAAPEI-5, Unigene66009\_TAEpaeTARAAPEI-5, Unigene66025\_TAEpaeTARAAPEI-5, Unigene66166\_TAEpaeTARAAPEI-5, Unigene66212\_TAEpaeTARAAPEI-5, Unigene66225\_TAEpaeTARAAPEI-5, Unigene66237\_TAEpaeTARAAPEI-5, Unigene66262\_TAEpaeTARAAPEI-5, Unigene66291\_TAEpaeTARAAPEI-5, Unigene6635\_TAEpaeTARAAPEI-5, Unigene66382\_TAEpaeTARAAPEI-5, Unigene66459\_TAEpaeTARAAPEI-5, Unigene66492\_TAEpaeTARAAPEI-5, Unigene66511\_TAEpaeTARAAPEI-5, Unigene66578\_TAEpaeTARAAPEI-5, Unigene66648\_TAEpaeTARAAPEI-5, Unigene66729\_TAEpaeTARAAPEI-5, Unigene66742\_TAEpaeTARAAPEI-5, Unigene66799\_TAEpaeTARAAPEI-5, Unigene66834\_TAEpaeTARAAPEI-5, Unigene66951\_TAEpaeTARAAPEI-5, Unigene66956\_TAEpaeTARAAPEI-5, Unigene67071\_TAEpaeTARAAPEI-5, Unigene67082\_TAEpaeTARAAPEI-5, Unigene67134\_TAEpaeTARAAPEI-5, Unigene67135\_TAEpaeTARAAPEI-5, Unigene67228\_TAEpaeTARAAPEI-5, Unigene67294\_TAEpaeTARAAPEI-5, Unigene67310\_TAEpaeTARAAPEI-5, Unigene67341\_TAEpaeTARAAPEI-5, Unigene67418\_TAEpaeTARAAPEI-5, Unigene6745\_TAEpaeTARAAPEI-5, Unigene67509\_TAEpaeTARAAPEI-5, Unigene67524\_TAEpaeTARAAPEI-5, Unigene67577\_TAEpaeTARAAPEI-5, Unigene67578\_TAEpaeTARAAPEI-5, Unigene67579\_TAEpaeTARAAPEI-5, Unigene67634\_TAEpaeTARAAPEI-5, Unigene67716\_TAEpaeTARAAPEI-5, Unigene67719\_TAEpaeTARAAPEI-5, Unigene67729\_TAEpaeTARAAPEI-5, Unigene67748\_TAEpaeTARAAPEI-5, Unigene67781\_TAEpaeTARAAPEI-5, Unigene67889\_TAEpaeTARAAPEI-5, Unigene68026\_TAEpaeTARAAPEI-5, Unigene68103\_TAEpaeTARAAPEI-5, Unigene68111\_TAEpaeTARAAPEI-5, Unigene68142\_TAEpaeTARAAPEI-5, Unigene68148\_TAEpaeTARAAPEI-5, Unigene68154\_TAEpaeTARAAPEI-5, Unigene68171\_TAEpaeTARAAPEI-5, Unigene68205\_TAEpaeTARAAPEI-5, Unigene68274\_TAEpaeTARAAPEI-5, Unigene68278\_TAEpaeTARAAPEI-5, Unigene68289\_TAEpaeTARAAPEI-5, Unigene68293\_TAEpaeTARAAPEI-5, Unigene68333\_TAEpaeTARAAPEI-5, Unigene68530\_TAEpaeTARAAPEI-5, Unigene68624\_TAEpaeTARAAPEI-5, Unigene68644\_TAEpaeTARAAPEI-5, Unigene68686\_TAEpaeTARAAPEI-5, Unigene68730\_TAEpaeTARAAPEI-5, Unigene68778\_TAEpaeTARAAPEI-5, Unigene68839\_TAEpaeTARAAPEI-5, Unigene68855\_TAEpaeTARAAPEI-5, Unigene68869\_TAEpaeTARAAPEI-5, Unigene68878\_TAEpaeTARAAPEI-5, Unigene68886\_TAEpaeTARAAPEI-5, Unigene68917\_TAEpaeTARAAPEI-5, Unigene68998\_TAEpaeTARAAPEI-5, Unigene69189\_TAEpaeTARAAPEI-5, Unigene69370\_TAEpaeTARAAPEI-5, Unigene6938\_TAEpaeTARAAPEI-5, Unigene69400\_TAEpaeTARAAPEI-5, Unigene6940\_TAEpaeTARAAPEI-5, Unigene69436\_TAEpaeTARAAPEI-5, Unigene69461\_TAEpaeTARAAPEI-5, Unigene69467\_TAEpaeTARAAPEI-5, Unigene69512\_TAEpaeTARAAPEI-5, Unigene69654\_TAEpaeTARAAPEI-5, Unigene69666\_TAEpaeTARAAPEI-5, Unigene69688\_TAEpaeTARAAPEI-5, Unigene69716\_TAEpaeTARAAPEI-5, Unigene69773\_TAEpaeTARAAPEI-5, Unigene69839\_TAEpaeTARAAPEI-5, Unigene70061\_TAEpaeTARAAPEI-5, Unigene70074\_TAEpaeTARAAPEI-5, Unigene70091\_TAEpaeTARAAPEI-5, Unigene70104\_TAEpaeTARAAPEI-5, Unigene70182\_TAEpaeTARAAPEI-5, Unigene70207\_TAEpaeTARAAPEI-5, Unigene70224\_TAEpaeTARAAPEI-5, Unigene70233\_TAEpaeTARAAPEI-5, Unigene70255\_TAEpaeTARAAPEI-5, Unigene70297\_TAEpaeTARAAPEI-5, Unigene70309\_TAEpaeTARAAPEI-5, Unigene70316\_TAEpaeTARAAPEI-5, Unigene70483\_TAEpaeTARAAPEI-5, Unigene70533\_TAEpaeTARAAPEI-5, Unigene70571\_TAEpaeTARAAPEI-5, Unigene70579\_TAEpaeTARAAPEI-5, Unigene70585\_TAEpaeTARAAPEI-5, Unigene70615\_TAEpaeTARAAPEI-5, Unigene70635\_TAEpaeTARAAPEI-5, Unigene70704\_TAEpaeTARAAPEI-5, Unigene70751\_TAEpaeTARAAPEI-5, Unigene70767\_TAEpaeTARAAPEI-5, Unigene70848\_TAEpaeTARAAPEI-5, Unigene70885\_TAEpaeTARAAPEI-5, Unigene70890\_TAEpaeTARAAPEI-5, Unigene70918\_TAEpaeTARAAPEI-5, Unigene70941\_TAEpaeTARAAPEI-5, Unigene71038\_TAEpaeTARAAPEI-5, Unigene71075\_TAEpaeTARAAPEI-5, Unigene71102\_TAEpaeTARAAPEI-5, Unigene71112\_TAEpaeTARAAPEI-5, Unigene71141\_TAEpaeTARAAPEI-5, Unigene71356\_TAEpaeTARAAPEI-5, Unigene71380\_TAEpaeTARAAPEI-5, Unigene71440\_TAEpaeTARAAPEI-5, Unigene71494\_TAEpaeTARAAPEI-5, Unigene71539\_TAEpaeTARAAPEI-5, Unigene71553\_TAEpaeTARAAPEI-5, Unigene71617\_TAEpaeTARAAPEI-5, Unigene71621\_TAEpaeTARAAPEI-5, Unigene71632\_TAEpaeTARAAPEI-5, Unigene71636\_TAEpaeTARAAPEI-5, Unigene71677\_TAEpaeTARAAPEI-5, Unigene71684\_TAEpaeTARAAPEI-5, Unigene71740\_TAEpaeTARAAPEI-5, Unigene71745\_TAEpaeTARAAPEI-5, Unigene71756\_TAEpaeTARAAPEI-5, Unigene71779\_TAEpaeTARAAPEI-5, Unigene71787\_TAEpaeTARAAPEI-5, Unigene71839\_TAEpaeTARAAPEI-5, Unigene71841\_TAEpaeTARAAPEI-5, Unigene71906\_TAEpaeTARAAPEI-5, Unigene72000\_TAEpaeTARAAPEI-5, Unigene72015\_TAEpaeTARAAPEI-5, Unigene72045\_TAEpaeTARAAPEI-5, Unigene72058\_TAEpaeTARAAPEI-5, Unigene72109\_TAEpaeTARAAPEI-5, Unigene72258\_TAEpaeTARAAPEI-5, Unigene72304\_TAEpaeTARAAPEI-5, Unigene72315\_TAEpaeTARAAPEI-5, Unigene72322\_TAEpaeTARAAPEI-5, Unigene72337\_TAEpaeTARAAPEI-5, Unigene72340\_TAEpaeTARAAPEI-5, Unigene72350\_TAEpaeTARAAPEI-5, Unigene72448\_TAEpaeTARAAPEI-5, Unigene72449\_TAEpaeTARAAPEI-5, Unigene72471\_TAEpaeTARAAPEI-5, Unigene72473\_TAEpaeTARAAPEI-5, Unigene72479\_TAEpaeTARAAPEI-5, Unigene72494\_TAEpaeTARAAPEI-5, Unigene72540\_TAEpaeTARAAPEI-5, Unigene72586\_TAEpaeTARAAPEI-5, Unigene72593\_TAEpaeTARAAPEI-5, Unigene72607\_TAEpaeTARAAPEI-5, Unigene72643\_TAEpaeTARAAPEI-5, Unigene72656\_TAEpaeTARAAPEI-5, Unigene72670\_TAEpaeTARAAPEI-5, Unigene72691\_TAEpaeTARAAPEI-5, Unigene72697\_TAEpaeTARAAPEI-5, Unigene72711\_TAEpaeTARAAPEI-5, Unigene72744\_TAEpaeTARAAPEI-5, Unigene72746\_TAEpaeTARAAPEI-5, Unigene72749\_TAEpaeTARAAPEI-5, Unigene72800\_TAEpaeTARAAPEI-5, Unigene72812\_TAEpaeTARAAPEI-5, Unigene72825\_TAEpaeTARAAPEI-5, Unigene72842\_TAEpaeTARAAPEI-5, Unigene72875\_TAEpaeTARAAPEI-5, Unigene72876\_TAEpaeTARAAPEI-5, Unigene72884\_TAEpaeTARAAPEI-5, Unigene72939\_TAEpaeTARAAPEI-5, Unigene7482\_TAEpaeTARAAPEI-5, Unigene7731\_TAEpaeTARAAPEI-5, Unigene7878\_TAEpaeTARAAPEI-5, Unigene8218\_TAEpaeTARAAPEI-5, Unigene8505\_TAEpaeTARAAPEI-5, Unigene8876\_TAEpaeTARAAPEI-5, Unigene8920\_TAEpaeTARAAPEI-5, Unigene907\_TAEpaeTARAAPEI-5, Unigene9304\_TAEpaeTARAAPEI-5, Unigene9577\_TAEpaeTARAAPEI-5, Unigene9723\_TAEpaeTARAAPEI-5, Unigene9731\_TAEpaeTARAAPEI-5, Unigene98\_TAEpaeTARAAPEI-5, Unigene9962\_TAEpaeTARAAPEI-5 |
| 6 | Pathways in cancer | Unigene10085\_TAEpaeTARAAPEI-5, Unigene10156\_TAEpaeTARAAPEI-5, Unigene10305\_TAEpaeTARAAPEI-5, Unigene10547\_TAEpaeTARAAPEI-5, Unigene10616\_TAEpaeTARAAPEI-5, Unigene10688\_TAEpaeTARAAPEI-5, Unigene10707\_TAEpaeTARAAPEI-5, Unigene10716\_TAEpaeTARAAPEI-5, Unigene10720\_TAEpaeTARAAPEI-5, Unigene11008\_TAEpaeTARAAPEI-5, Unigene11123\_TAEpaeTARAAPEI-5, Unigene11205\_TAEpaeTARAAPEI-5, Unigene11257\_TAEpaeTARAAPEI-5, Unigene11397\_TAEpaeTARAAPEI-5, Unigene11507\_TAEpaeTARAAPEI-5, Unigene11610\_TAEpaeTARAAPEI-5, Unigene11645\_TAEpaeTARAAPEI-5, Unigene11829\_TAEpaeTARAAPEI-5, Unigene12152\_TAEpaeTARAAPEI-5, Unigene12159\_TAEpaeTARAAPEI-5, Unigene12165\_TAEpaeTARAAPEI-5, Unigene12167\_TAEpaeTARAAPEI-5, Unigene12203\_TAEpaeTARAAPEI-5, Unigene12758\_TAEpaeTARAAPEI-5, Unigene12895\_TAEpaeTARAAPEI-5, Unigene12956\_TAEpaeTARAAPEI-5, Unigene13000\_TAEpaeTARAAPEI-5, Unigene13473\_TAEpaeTARAAPEI-5, Unigene13629\_TAEpaeTARAAPEI-5, Unigene13656\_TAEpaeTARAAPEI-5, Unigene13747\_TAEpaeTARAAPEI-5, Unigene13910\_TAEpaeTARAAPEI-5, Unigene13919\_TAEpaeTARAAPEI-5, Unigene14027\_TAEpaeTARAAPEI-5, Unigene14092\_TAEpaeTARAAPEI-5, Unigene14114\_TAEpaeTARAAPEI-5, Unigene14133\_TAEpaeTARAAPEI-5, Unigene14135\_TAEpaeTARAAPEI-5, Unigene14151\_TAEpaeTARAAPEI-5, Unigene14178\_TAEpaeTARAAPEI-5, Unigene14192\_TAEpaeTARAAPEI-5, Unigene14226\_TAEpaeTARAAPEI-5, Unigene14270\_TAEpaeTARAAPEI-5, Unigene14628\_TAEpaeTARAAPEI-5, Unigene14714\_TAEpaeTARAAPEI-5, Unigene14763\_TAEpaeTARAAPEI-5, Unigene14804\_TAEpaeTARAAPEI-5, Unigene14945\_TAEpaeTARAAPEI-5, Unigene15053\_TAEpaeTARAAPEI-5, Unigene15075\_TAEpaeTARAAPEI-5, Unigene15107\_TAEpaeTARAAPEI-5, Unigene15135\_TAEpaeTARAAPEI-5, Unigene15194\_TAEpaeTARAAPEI-5, Unigene15247\_TAEpaeTARAAPEI-5, Unigene15269\_TAEpaeTARAAPEI-5, Unigene15450\_TAEpaeTARAAPEI-5, Unigene15610\_TAEpaeTARAAPEI-5, Unigene15620\_TAEpaeTARAAPEI-5, Unigene15628\_TAEpaeTARAAPEI-5, Unigene15730\_TAEpaeTARAAPEI-5, Unigene15756\_TAEpaeTARAAPEI-5, Unigene15768\_TAEpaeTARAAPEI-5, Unigene15784\_TAEpaeTARAAPEI-5, Unigene15975\_TAEpaeTARAAPEI-5, Unigene15987\_TAEpaeTARAAPEI-5, Unigene16008\_TAEpaeTARAAPEI-5, Unigene16103\_TAEpaeTARAAPEI-5, Unigene16189\_TAEpaeTARAAPEI-5, Unigene16230\_TAEpaeTARAAPEI-5, Unigene16282\_TAEpaeTARAAPEI-5, Unigene16371\_TAEpaeTARAAPEI-5, Unigene16454\_TAEpaeTARAAPEI-5, Unigene16468\_TAEpaeTARAAPEI-5, Unigene16483\_TAEpaeTARAAPEI-5, Unigene16518\_TAEpaeTARAAPEI-5, Unigene16800\_TAEpaeTARAAPEI-5, Unigene16815\_TAEpaeTARAAPEI-5, Unigene16817\_TAEpaeTARAAPEI-5, Unigene16821\_TAEpaeTARAAPEI-5, Unigene16824\_TAEpaeTARAAPEI-5, Unigene17126\_TAEpaeTARAAPEI-5, Unigene17145\_TAEpaeTARAAPEI-5, Unigene17167\_TAEpaeTARAAPEI-5, Unigene17221\_TAEpaeTARAAPEI-5, Unigene17338\_TAEpaeTARAAPEI-5, Unigene17364\_TAEpaeTARAAPEI-5, Unigene17397\_TAEpaeTARAAPEI-5, Unigene17450\_TAEpaeTARAAPEI-5, Unigene17474\_TAEpaeTARAAPEI-5, Unigene17475\_TAEpaeTARAAPEI-5, Unigene17673\_TAEpaeTARAAPEI-5, Unigene17700\_TAEpaeTARAAPEI-5, Unigene17706\_TAEpaeTARAAPEI-5, Unigene17833\_TAEpaeTARAAPEI-5, Unigene17839\_TAEpaeTARAAPEI-5, Unigene17926\_TAEpaeTARAAPEI-5, Unigene17932\_TAEpaeTARAAPEI-5, Unigene17946\_TAEpaeTARAAPEI-5, Unigene17947\_TAEpaeTARAAPEI-5, Unigene17969\_TAEpaeTARAAPEI-5, Unigene17970\_TAEpaeTARAAPEI-5, Unigene18102\_TAEpaeTARAAPEI-5, Unigene18151\_TAEpaeTARAAPEI-5, Unigene19554\_TAEpaeTARAAPEI-5, Unigene19640\_TAEpaeTARAAPEI-5, Unigene19736\_TAEpaeTARAAPEI-5, Unigene20359\_TAEpaeTARAAPEI-5, Unigene20549\_TAEpaeTARAAPEI-5, Unigene20934\_TAEpaeTARAAPEI-5, Unigene21363\_TAEpaeTARAAPEI-5, Unigene2158\_TAEpaeTARAAPEI-5, Unigene21846\_TAEpaeTARAAPEI-5, Unigene21875\_TAEpaeTARAAPEI-5, Unigene21960\_TAEpaeTARAAPEI-5, Unigene2206\_TAEpaeTARAAPEI-5, Unigene22121\_TAEpaeTARAAPEI-5, Unigene22256\_TAEpaeTARAAPEI-5, Unigene22264\_TAEpaeTARAAPEI-5, Unigene22265\_TAEpaeTARAAPEI-5, Unigene22299\_TAEpaeTARAAPEI-5, Unigene2241\_TAEpaeTARAAPEI-5, Unigene22526\_TAEpaeTARAAPEI-5, Unigene2291\_TAEpaeTARAAPEI-5, Unigene23040\_TAEpaeTARAAPEI-5, Unigene23210\_TAEpaeTARAAPEI-5, Unigene23237\_TAEpaeTARAAPEI-5, Unigene23520\_TAEpaeTARAAPEI-5, Unigene23919\_TAEpaeTARAAPEI-5, Unigene2429\_TAEpaeTARAAPEI-5, Unigene24488\_TAEpaeTARAAPEI-5, Unigene24595\_TAEpaeTARAAPEI-5, Unigene24688\_TAEpaeTARAAPEI-5, Unigene25003\_TAEpaeTARAAPEI-5, Unigene25231\_TAEpaeTARAAPEI-5, Unigene25334\_TAEpaeTARAAPEI-5, Unigene25877\_TAEpaeTARAAPEI-5, Unigene25921\_TAEpaeTARAAPEI-5, Unigene25960\_TAEpaeTARAAPEI-5, Unigene26033\_TAEpaeTARAAPEI-5, Unigene26194\_TAEpaeTARAAPEI-5, Unigene26355\_TAEpaeTARAAPEI-5, Unigene26859\_TAEpaeTARAAPEI-5, Unigene26907\_TAEpaeTARAAPEI-5, Unigene27477\_TAEpaeTARAAPEI-5, Unigene27748\_TAEpaeTARAAPEI-5, Unigene28135\_TAEpaeTARAAPEI-5, Unigene28371\_TAEpaeTARAAPEI-5, Unigene28415\_TAEpaeTARAAPEI-5, Unigene28476\_TAEpaeTARAAPEI-5, Unigene2882\_TAEpaeTARAAPEI-5, Unigene28987\_TAEpaeTARAAPEI-5, Unigene29077\_TAEpaeTARAAPEI-5, Unigene29903\_TAEpaeTARAAPEI-5, Unigene29929\_TAEpaeTARAAPEI-5, Unigene30199\_TAEpaeTARAAPEI-5, Unigene30316\_TAEpaeTARAAPEI-5, Unigene30460\_TAEpaeTARAAPEI-5, Unigene30803\_TAEpaeTARAAPEI-5, Unigene31513\_TAEpaeTARAAPEI-5, Unigene31877\_TAEpaeTARAAPEI-5, Unigene32040\_TAEpaeTARAAPEI-5, Unigene32081\_TAEpaeTARAAPEI-5, Unigene32182\_TAEpaeTARAAPEI-5, Unigene32212\_TAEpaeTARAAPEI-5, Unigene3258\_TAEpaeTARAAPEI-5, Unigene32814\_TAEpaeTARAAPEI-5, Unigene33089\_TAEpaeTARAAPEI-5, Unigene33399\_TAEpaeTARAAPEI-5, Unigene33487\_TAEpaeTARAAPEI-5, Unigene33986\_TAEpaeTARAAPEI-5, Unigene34144\_TAEpaeTARAAPEI-5, Unigene34178\_TAEpaeTARAAPEI-5, Unigene34259\_TAEpaeTARAAPEI-5, Unigene34287\_TAEpaeTARAAPEI-5, Unigene34344\_TAEpaeTARAAPEI-5, Unigene345\_TAEpaeTARAAPEI-5, Unigene34845\_TAEpaeTARAAPEI-5, Unigene34957\_TAEpaeTARAAPEI-5, Unigene34972\_TAEpaeTARAAPEI-5, Unigene35102\_TAEpaeTARAAPEI-5, Unigene35454\_TAEpaeTARAAPEI-5, Unigene3559\_TAEpaeTARAAPEI-5, Unigene35733\_TAEpaeTARAAPEI-5, Unigene3582\_TAEpaeTARAAPEI-5, Unigene35839\_TAEpaeTARAAPEI-5, Unigene35973\_TAEpaeTARAAPEI-5, Unigene36256\_TAEpaeTARAAPEI-5, Unigene36447\_TAEpaeTARAAPEI-5, Unigene36580\_TAEpaeTARAAPEI-5, Unigene37038\_TAEpaeTARAAPEI-5, Unigene37112\_TAEpaeTARAAPEI-5, Unigene37247\_TAEpaeTARAAPEI-5, Unigene37318\_TAEpaeTARAAPEI-5, Unigene3737\_TAEpaeTARAAPEI-5, Unigene37500\_TAEpaeTARAAPEI-5, Unigene37692\_TAEpaeTARAAPEI-5, Unigene37724\_TAEpaeTARAAPEI-5, Unigene3788\_TAEpaeTARAAPEI-5, Unigene37919\_TAEpaeTARAAPEI-5, Unigene37937\_TAEpaeTARAAPEI-5, Unigene37980\_TAEpaeTARAAPEI-5, Unigene38035\_TAEpaeTARAAPEI-5, Unigene38037\_TAEpaeTARAAPEI-5, Unigene38062\_TAEpaeTARAAPEI-5, Unigene38160\_TAEpaeTARAAPEI-5, Unigene38413\_TAEpaeTARAAPEI-5, Unigene38441\_TAEpaeTARAAPEI-5, Unigene38551\_TAEpaeTARAAPEI-5, Unigene38671\_TAEpaeTARAAPEI-5, Unigene38851\_TAEpaeTARAAPEI-5, Unigene3888\_TAEpaeTARAAPEI-5, Unigene39051\_TAEpaeTARAAPEI-5, Unigene39180\_TAEpaeTARAAPEI-5, Unigene39407\_TAEpaeTARAAPEI-5, Unigene39696\_TAEpaeTARAAPEI-5, Unigene39861\_TAEpaeTARAAPEI-5, Unigene40344\_TAEpaeTARAAPEI-5, Unigene40826\_TAEpaeTARAAPEI-5, Unigene40999\_TAEpaeTARAAPEI-5, Unigene41176\_TAEpaeTARAAPEI-5, Unigene41673\_TAEpaeTARAAPEI-5, Unigene41799\_TAEpaeTARAAPEI-5, Unigene41827\_TAEpaeTARAAPEI-5, Unigene41855\_TAEpaeTARAAPEI-5, Unigene42098\_TAEpaeTARAAPEI-5, Unigene43003\_TAEpaeTARAAPEI-5, Unigene43076\_TAEpaeTARAAPEI-5, Unigene43135\_TAEpaeTARAAPEI-5, Unigene43193\_TAEpaeTARAAPEI-5, Unigene43195\_TAEpaeTARAAPEI-5, Unigene43198\_TAEpaeTARAAPEI-5, Unigene43303\_TAEpaeTARAAPEI-5, Unigene4371\_TAEpaeTARAAPEI-5, Unigene4380\_TAEpaeTARAAPEI-5, Unigene43842\_TAEpaeTARAAPEI-5, Unigene4384\_TAEpaeTARAAPEI-5, Unigene44202\_TAEpaeTARAAPEI-5, Unigene4425\_TAEpaeTARAAPEI-5, Unigene44287\_TAEpaeTARAAPEI-5, Unigene44401\_TAEpaeTARAAPEI-5, Unigene4451\_TAEpaeTARAAPEI-5, Unigene44580\_TAEpaeTARAAPEI-5, Unigene44633\_TAEpaeTARAAPEI-5, Unigene44643\_TAEpaeTARAAPEI-5, Unigene44755\_TAEpaeTARAAPEI-5, Unigene44881\_TAEpaeTARAAPEI-5, Unigene44955\_TAEpaeTARAAPEI-5, Unigene45180\_TAEpaeTARAAPEI-5, Unigene45433\_TAEpaeTARAAPEI-5, Unigene45602\_TAEpaeTARAAPEI-5, Unigene45712\_TAEpaeTARAAPEI-5, Unigene46290\_TAEpaeTARAAPEI-5, Unigene46298\_TAEpaeTARAAPEI-5, Unigene46657\_TAEpaeTARAAPEI-5, Unigene46788\_TAEpaeTARAAPEI-5, Unigene46844\_TAEpaeTARAAPEI-5, Unigene46853\_TAEpaeTARAAPEI-5, Unigene46962\_TAEpaeTARAAPEI-5, Unigene47023\_TAEpaeTARAAPEI-5, Unigene47047\_TAEpaeTARAAPEI-5, Unigene4716\_TAEpaeTARAAPEI-5, Unigene47218\_TAEpaeTARAAPEI-5, Unigene47595\_TAEpaeTARAAPEI-5, Unigene47753\_TAEpaeTARAAPEI-5, Unigene47898\_TAEpaeTARAAPEI-5, Unigene4790\_TAEpaeTARAAPEI-5, Unigene48002\_TAEpaeTARAAPEI-5, Unigene480\_TAEpaeTARAAPEI-5, Unigene48102\_TAEpaeTARAAPEI-5, Unigene48247\_TAEpaeTARAAPEI-5, Unigene48256\_TAEpaeTARAAPEI-5, Unigene48377\_TAEpaeTARAAPEI-5, Unigene48394\_TAEpaeTARAAPEI-5, Unigene48575\_TAEpaeTARAAPEI-5, Unigene48580\_TAEpaeTARAAPEI-5, Unigene48649\_TAEpaeTARAAPEI-5, Unigene48725\_TAEpaeTARAAPEI-5, Unigene4901\_TAEpaeTARAAPEI-5, Unigene49023\_TAEpaeTARAAPEI-5, Unigene49047\_TAEpaeTARAAPEI-5, Unigene49085\_TAEpaeTARAAPEI-5, Unigene49108\_TAEpaeTARAAPEI-5, Unigene49135\_TAEpaeTARAAPEI-5, Unigene49173\_TAEpaeTARAAPEI-5, Unigene49351\_TAEpaeTARAAPEI-5, Unigene49598\_TAEpaeTARAAPEI-5, Unigene49657\_TAEpaeTARAAPEI-5, Unigene49990\_TAEpaeTARAAPEI-5, Unigene50359\_TAEpaeTARAAPEI-5, Unigene50455\_TAEpaeTARAAPEI-5, Unigene50474\_TAEpaeTARAAPEI-5, Unigene50566\_TAEpaeTARAAPEI-5, Unigene50761\_TAEpaeTARAAPEI-5, Unigene51205\_TAEpaeTARAAPEI-5, Unigene51253\_TAEpaeTARAAPEI-5, Unigene5158\_TAEpaeTARAAPEI-5, Unigene51601\_TAEpaeTARAAPEI-5, Unigene51611\_TAEpaeTARAAPEI-5, Unigene51681\_TAEpaeTARAAPEI-5, Unigene51722\_TAEpaeTARAAPEI-5, Unigene5194\_TAEpaeTARAAPEI-5, Unigene51974\_TAEpaeTARAAPEI-5, Unigene52160\_TAEpaeTARAAPEI-5, Unigene52306\_TAEpaeTARAAPEI-5, Unigene52308\_TAEpaeTARAAPEI-5, Unigene52351\_TAEpaeTARAAPEI-5, Unigene52452\_TAEpaeTARAAPEI-5, Unigene5248\_TAEpaeTARAAPEI-5, Unigene52611\_TAEpaeTARAAPEI-5, Unigene53028\_TAEpaeTARAAPEI-5, Unigene53203\_TAEpaeTARAAPEI-5, Unigene53542\_TAEpaeTARAAPEI-5, Unigene53786\_TAEpaeTARAAPEI-5, Unigene53911\_TAEpaeTARAAPEI-5, Unigene54063\_TAEpaeTARAAPEI-5, Unigene54065\_TAEpaeTARAAPEI-5, Unigene54113\_TAEpaeTARAAPEI-5, Unigene5411\_TAEpaeTARAAPEI-5, Unigene54218\_TAEpaeTARAAPEI-5, Unigene54359\_TAEpaeTARAAPEI-5, Unigene54363\_TAEpaeTARAAPEI-5, Unigene54441\_TAEpaeTARAAPEI-5, Unigene54962\_TAEpaeTARAAPEI-5, Unigene55024\_TAEpaeTARAAPEI-5, Unigene55027\_TAEpaeTARAAPEI-5, Unigene55111\_TAEpaeTARAAPEI-5, Unigene55117\_TAEpaeTARAAPEI-5, Unigene55226\_TAEpaeTARAAPEI-5, Unigene55341\_TAEpaeTARAAPEI-5, Unigene55494\_TAEpaeTARAAPEI-5, Unigene55569\_TAEpaeTARAAPEI-5, Unigene55612\_TAEpaeTARAAPEI-5, Unigene55954\_TAEpaeTARAAPEI-5, Unigene56110\_TAEpaeTARAAPEI-5, Unigene56444\_TAEpaeTARAAPEI-5, Unigene56445\_TAEpaeTARAAPEI-5, Unigene5655\_TAEpaeTARAAPEI-5, Unigene56816\_TAEpaeTARAAPEI-5, Unigene56915\_TAEpaeTARAAPEI-5, Unigene5694\_TAEpaeTARAAPEI-5, Unigene57083\_TAEpaeTARAAPEI-5, Unigene57095\_TAEpaeTARAAPEI-5, Unigene57151\_TAEpaeTARAAPEI-5, Unigene57292\_TAEpaeTARAAPEI-5, Unigene57329\_TAEpaeTARAAPEI-5, Unigene57458\_TAEpaeTARAAPEI-5, Unigene57482\_TAEpaeTARAAPEI-5, Unigene57492\_TAEpaeTARAAPEI-5, Unigene57536\_TAEpaeTARAAPEI-5, Unigene57575\_TAEpaeTARAAPEI-5, Unigene57576\_TAEpaeTARAAPEI-5, Unigene57610\_TAEpaeTARAAPEI-5, Unigene57670\_TAEpaeTARAAPEI-5, Unigene57969\_TAEpaeTARAAPEI-5, Unigene57974\_TAEpaeTARAAPEI-5, Unigene58098\_TAEpaeTARAAPEI-5, Unigene58191\_TAEpaeTARAAPEI-5, Unigene58278\_TAEpaeTARAAPEI-5, Unigene58427\_TAEpaeTARAAPEI-5, Unigene58848\_TAEpaeTARAAPEI-5, Unigene58863\_TAEpaeTARAAPEI-5, Unigene59005\_TAEpaeTARAAPEI-5, Unigene59048\_TAEpaeTARAAPEI-5, Unigene59099\_TAEpaeTARAAPEI-5, Unigene59282\_TAEpaeTARAAPEI-5, Unigene5934\_TAEpaeTARAAPEI-5, Unigene59442\_TAEpaeTARAAPEI-5, Unigene59624\_TAEpaeTARAAPEI-5, Unigene5970\_TAEpaeTARAAPEI-5, Unigene59754\_TAEpaeTARAAPEI-5, Unigene59927\_TAEpaeTARAAPEI-5, Unigene60091\_TAEpaeTARAAPEI-5, Unigene60110\_TAEpaeTARAAPEI-5, Unigene60216\_TAEpaeTARAAPEI-5, Unigene60254\_TAEpaeTARAAPEI-5, Unigene60339\_TAEpaeTARAAPEI-5, Unigene60345\_TAEpaeTARAAPEI-5, Unigene60383\_TAEpaeTARAAPEI-5, Unigene60472\_TAEpaeTARAAPEI-5, Unigene60743\_TAEpaeTARAAPEI-5, Unigene61029\_TAEpaeTARAAPEI-5, Unigene6116\_TAEpaeTARAAPEI-5, Unigene61511\_TAEpaeTARAAPEI-5, Unigene62020\_TAEpaeTARAAPEI-5, Unigene62275\_TAEpaeTARAAPEI-5, Unigene62300\_TAEpaeTARAAPEI-5, Unigene62502\_TAEpaeTARAAPEI-5, Unigene6260\_TAEpaeTARAAPEI-5, Unigene62705\_TAEpaeTARAAPEI-5, Unigene62780\_TAEpaeTARAAPEI-5, Unigene62782\_TAEpaeTARAAPEI-5, Unigene62793\_TAEpaeTARAAPEI-5, Unigene62962\_TAEpaeTARAAPEI-5, Unigene63051\_TAEpaeTARAAPEI-5, Unigene63225\_TAEpaeTARAAPEI-5, Unigene63365\_TAEpaeTARAAPEI-5, Unigene63523\_TAEpaeTARAAPEI-5, Unigene63586\_TAEpaeTARAAPEI-5, Unigene63743\_TAEpaeTARAAPEI-5, Unigene63745\_TAEpaeTARAAPEI-5, Unigene63877\_TAEpaeTARAAPEI-5, Unigene63980\_TAEpaeTARAAPEI-5, Unigene64264\_TAEpaeTARAAPEI-5, Unigene64279\_TAEpaeTARAAPEI-5, Unigene64293\_TAEpaeTARAAPEI-5, Unigene64297\_TAEpaeTARAAPEI-5, Unigene64426\_TAEpaeTARAAPEI-5, Unigene64687\_TAEpaeTARAAPEI-5, Unigene64757\_TAEpaeTARAAPEI-5, Unigene64814\_TAEpaeTARAAPEI-5, Unigene64901\_TAEpaeTARAAPEI-5, Unigene65279\_TAEpaeTARAAPEI-5, Unigene65342\_TAEpaeTARAAPEI-5, Unigene65502\_TAEpaeTARAAPEI-5, Unigene65658\_TAEpaeTARAAPEI-5, Unigene65703\_TAEpaeTARAAPEI-5, Unigene65717\_TAEpaeTARAAPEI-5, Unigene65789\_TAEpaeTARAAPEI-5, Unigene65827\_TAEpaeTARAAPEI-5, Unigene65845\_TAEpaeTARAAPEI-5, Unigene65920\_TAEpaeTARAAPEI-5, Unigene66025\_TAEpaeTARAAPEI-5, Unigene66211\_TAEpaeTARAAPEI-5, Unigene66291\_TAEpaeTARAAPEI-5, Unigene66307\_TAEpaeTARAAPEI-5, Unigene66492\_TAEpaeTARAAPEI-5, Unigene66511\_TAEpaeTARAAPEI-5, Unigene66529\_TAEpaeTARAAPEI-5, Unigene66587\_TAEpaeTARAAPEI-5, Unigene66651\_TAEpaeTARAAPEI-5, Unigene66714\_TAEpaeTARAAPEI-5, Unigene66742\_TAEpaeTARAAPEI-5, Unigene66829\_TAEpaeTARAAPEI-5, Unigene66834\_TAEpaeTARAAPEI-5, Unigene66916\_TAEpaeTARAAPEI-5, Unigene66993\_TAEpaeTARAAPEI-5, Unigene67163\_TAEpaeTARAAPEI-5, Unigene6726\_TAEpaeTARAAPEI-5, Unigene67341\_TAEpaeTARAAPEI-5, Unigene67524\_TAEpaeTARAAPEI-5, Unigene67546\_TAEpaeTARAAPEI-5, Unigene67579\_TAEpaeTARAAPEI-5, Unigene67634\_TAEpaeTARAAPEI-5, Unigene67719\_TAEpaeTARAAPEI-5, Unigene67781\_TAEpaeTARAAPEI-5, Unigene67822\_TAEpaeTARAAPEI-5, Unigene67823\_TAEpaeTARAAPEI-5, Unigene67865\_TAEpaeTARAAPEI-5, Unigene68001\_TAEpaeTARAAPEI-5, Unigene68026\_TAEpaeTARAAPEI-5, Unigene68088\_TAEpaeTARAAPEI-5, Unigene68103\_TAEpaeTARAAPEI-5, Unigene68109\_TAEpaeTARAAPEI-5, Unigene68137\_TAEpaeTARAAPEI-5, Unigene68142\_TAEpaeTARAAPEI-5, Unigene68148\_TAEpaeTARAAPEI-5, Unigene68197\_TAEpaeTARAAPEI-5, Unigene68202\_TAEpaeTARAAPEI-5, Unigene68223\_TAEpaeTARAAPEI-5, Unigene68278\_TAEpaeTARAAPEI-5, Unigene68317\_TAEpaeTARAAPEI-5, Unigene68333\_TAEpaeTARAAPEI-5, Unigene68341\_TAEpaeTARAAPEI-5, Unigene68491\_TAEpaeTARAAPEI-5, Unigene68530\_TAEpaeTARAAPEI-5, Unigene68651\_TAEpaeTARAAPEI-5, Unigene6868\_TAEpaeTARAAPEI-5, Unigene68734\_TAEpaeTARAAPEI-5, Unigene68739\_TAEpaeTARAAPEI-5, Unigene68840\_TAEpaeTARAAPEI-5, Unigene68855\_TAEpaeTARAAPEI-5, Unigene68869\_TAEpaeTARAAPEI-5, Unigene68878\_TAEpaeTARAAPEI-5, Unigene68903\_TAEpaeTARAAPEI-5, Unigene68937\_TAEpaeTARAAPEI-5, Unigene68944\_TAEpaeTARAAPEI-5, Unigene68998\_TAEpaeTARAAPEI-5, Unigene69044\_TAEpaeTARAAPEI-5, Unigene69095\_TAEpaeTARAAPEI-5, Unigene69130\_TAEpaeTARAAPEI-5, Unigene69174\_TAEpaeTARAAPEI-5, Unigene69219\_TAEpaeTARAAPEI-5, Unigene69231\_TAEpaeTARAAPEI-5, Unigene69400\_TAEpaeTARAAPEI-5, Unigene69407\_TAEpaeTARAAPEI-5, Unigene69439\_TAEpaeTARAAPEI-5, Unigene69488\_TAEpaeTARAAPEI-5, Unigene69512\_TAEpaeTARAAPEI-5, Unigene69577\_TAEpaeTARAAPEI-5, Unigene69580\_TAEpaeTARAAPEI-5, Unigene69607\_TAEpaeTARAAPEI-5, Unigene69632\_TAEpaeTARAAPEI-5, Unigene69644\_TAEpaeTARAAPEI-5, Unigene69651\_TAEpaeTARAAPEI-5, Unigene69666\_TAEpaeTARAAPEI-5, Unigene69688\_TAEpaeTARAAPEI-5, Unigene69896\_TAEpaeTARAAPEI-5, Unigene69978\_TAEpaeTARAAPEI-5, Unigene70027\_TAEpaeTARAAPEI-5, Unigene7002\_TAEpaeTARAAPEI-5, Unigene70033\_TAEpaeTARAAPEI-5, Unigene70072\_TAEpaeTARAAPEI-5, Unigene70089\_TAEpaeTARAAPEI-5, Unigene70091\_TAEpaeTARAAPEI-5, Unigene70104\_TAEpaeTARAAPEI-5, Unigene70182\_TAEpaeTARAAPEI-5, Unigene70207\_TAEpaeTARAAPEI-5, Unigene70224\_TAEpaeTARAAPEI-5, Unigene70233\_TAEpaeTARAAPEI-5, Unigene70337\_TAEpaeTARAAPEI-5, Unigene70392\_TAEpaeTARAAPEI-5, Unigene70424\_TAEpaeTARAAPEI-5, Unigene70495\_TAEpaeTARAAPEI-5, Unigene70535\_TAEpaeTARAAPEI-5, Unigene70546\_TAEpaeTARAAPEI-5, Unigene70549\_TAEpaeTARAAPEI-5, Unigene70585\_TAEpaeTARAAPEI-5, Unigene70596\_TAEpaeTARAAPEI-5, Unigene70650\_TAEpaeTARAAPEI-5, Unigene70756\_TAEpaeTARAAPEI-5, Unigene70767\_TAEpaeTARAAPEI-5, Unigene70893\_TAEpaeTARAAPEI-5, Unigene70900\_TAEpaeTARAAPEI-5, Unigene70903\_TAEpaeTARAAPEI-5, Unigene70968\_TAEpaeTARAAPEI-5, Unigene70974\_TAEpaeTARAAPEI-5, Unigene71075\_TAEpaeTARAAPEI-5, Unigene71102\_TAEpaeTARAAPEI-5, Unigene71107\_TAEpaeTARAAPEI-5, Unigene71238\_TAEpaeTARAAPEI-5, Unigene71380\_TAEpaeTARAAPEI-5, Unigene71438\_TAEpaeTARAAPEI-5, Unigene71440\_TAEpaeTARAAPEI-5, Unigene71471\_TAEpaeTARAAPEI-5, Unigene71513\_TAEpaeTARAAPEI-5, Unigene71617\_TAEpaeTARAAPEI-5, Unigene71635\_TAEpaeTARAAPEI-5, Unigene71641\_TAEpaeTARAAPEI-5, Unigene71677\_TAEpaeTARAAPEI-5, Unigene71684\_TAEpaeTARAAPEI-5, Unigene71703\_TAEpaeTARAAPEI-5, Unigene71723\_TAEpaeTARAAPEI-5, Unigene71756\_TAEpaeTARAAPEI-5, Unigene71762\_TAEpaeTARAAPEI-5, Unigene71779\_TAEpaeTARAAPEI-5, Unigene71820\_TAEpaeTARAAPEI-5, Unigene71822\_TAEpaeTARAAPEI-5, Unigene71828\_TAEpaeTARAAPEI-5, Unigene71841\_TAEpaeTARAAPEI-5, Unigene71906\_TAEpaeTARAAPEI-5, Unigene72033\_TAEpaeTARAAPEI-5, Unigene72044\_TAEpaeTARAAPEI-5, Unigene72045\_TAEpaeTARAAPEI-5, Unigene72058\_TAEpaeTARAAPEI-5, Unigene72068\_TAEpaeTARAAPEI-5, Unigene72109\_TAEpaeTARAAPEI-5, Unigene72115\_TAEpaeTARAAPEI-5, Unigene72155\_TAEpaeTARAAPEI-5, Unigene72164\_TAEpaeTARAAPEI-5, Unigene72168\_TAEpaeTARAAPEI-5, Unigene72199\_TAEpaeTARAAPEI-5, Unigene72222\_TAEpaeTARAAPEI-5, Unigene72258\_TAEpaeTARAAPEI-5, Unigene72261\_TAEpaeTARAAPEI-5, Unigene72280\_TAEpaeTARAAPEI-5, Unigene72281\_TAEpaeTARAAPEI-5, Unigene72300\_TAEpaeTARAAPEI-5, Unigene72315\_TAEpaeTARAAPEI-5, Unigene72322\_TAEpaeTARAAPEI-5, Unigene72323\_TAEpaeTARAAPEI-5, Unigene72337\_TAEpaeTARAAPEI-5, Unigene72340\_TAEpaeTARAAPEI-5, Unigene72350\_TAEpaeTARAAPEI-5, Unigene72448\_TAEpaeTARAAPEI-5, Unigene72449\_TAEpaeTARAAPEI-5, Unigene72494\_TAEpaeTARAAPEI-5, Unigene72540\_TAEpaeTARAAPEI-5, Unigene72635\_TAEpaeTARAAPEI-5, Unigene72643\_TAEpaeTARAAPEI-5, Unigene72655\_TAEpaeTARAAPEI-5, Unigene72675\_TAEpaeTARAAPEI-5, Unigene72691\_TAEpaeTARAAPEI-5, Unigene72697\_TAEpaeTARAAPEI-5, Unigene72744\_TAEpaeTARAAPEI-5, Unigene72786\_TAEpaeTARAAPEI-5, Unigene72821\_TAEpaeTARAAPEI-5, Unigene72822\_TAEpaeTARAAPEI-5, Unigene72825\_TAEpaeTARAAPEI-5, Unigene72842\_TAEpaeTARAAPEI-5, Unigene72853\_TAEpaeTARAAPEI-5, Unigene72875\_TAEpaeTARAAPEI-5, Unigene72883\_TAEpaeTARAAPEI-5, Unigene72926\_TAEpaeTARAAPEI-5, Unigene7406\_TAEpaeTARAAPEI-5, Unigene7482\_TAEpaeTARAAPEI-5, Unigene7487\_TAEpaeTARAAPEI-5, Unigene7574\_TAEpaeTARAAPEI-5, Unigene8114\_TAEpaeTARAAPEI-5, Unigene8228\_TAEpaeTARAAPEI-5, Unigene8360\_TAEpaeTARAAPEI-5, Unigene8737\_TAEpaeTARAAPEI-5, Unigene8842\_TAEpaeTARAAPEI-5, Unigene8873\_TAEpaeTARAAPEI-5, Unigene8920\_TAEpaeTARAAPEI-5, Unigene8933\_TAEpaeTARAAPEI-5, Unigene9076\_TAEpaeTARAAPEI-5, Unigene907\_TAEpaeTARAAPEI-5, Unigene9339\_TAEpaeTARAAPEI-5, Unigene9376\_TAEpaeTARAAPEI-5, Unigene9443\_TAEpaeTARAAPEI-5, Unigene9481\_TAEpaeTARAAPEI-5, Unigene9577\_TAEpaeTARAAPEI-5 |
| 7 | Tight junction | Unigene10067\_TAEpaeTARAAPEI-5, Unigene10177\_TAEpaeTARAAPEI-5, Unigene10250\_TAEpaeTARAAPEI-5, Unigene10425\_TAEpaeTARAAPEI-5, Unigene10444\_TAEpaeTARAAPEI-5, Unigene10544\_TAEpaeTARAAPEI-5, Unigene10547\_TAEpaeTARAAPEI-5, Unigene10658\_TAEpaeTARAAPEI-5, Unigene10787\_TAEpaeTARAAPEI-5, Unigene10835\_TAEpaeTARAAPEI-5, Unigene10863\_TAEpaeTARAAPEI-5, Unigene1087\_TAEpaeTARAAPEI-5, Unigene10907\_TAEpaeTARAAPEI-5, Unigene11056\_TAEpaeTARAAPEI-5, Unigene11280\_TAEpaeTARAAPEI-5, Unigene11328\_TAEpaeTARAAPEI-5, Unigene11341\_TAEpaeTARAAPEI-5, Unigene11417\_TAEpaeTARAAPEI-5, Unigene11482\_TAEpaeTARAAPEI-5, Unigene11534\_TAEpaeTARAAPEI-5, Unigene11585\_TAEpaeTARAAPEI-5, Unigene11787\_TAEpaeTARAAPEI-5, Unigene12145\_TAEpaeTARAAPEI-5, Unigene12175\_TAEpaeTARAAPEI-5, Unigene12275\_TAEpaeTARAAPEI-5, Unigene12401\_TAEpaeTARAAPEI-5, Unigene12478\_TAEpaeTARAAPEI-5, Unigene12489\_TAEpaeTARAAPEI-5, Unigene12723\_TAEpaeTARAAPEI-5, Unigene12878\_TAEpaeTARAAPEI-5, Unigene12888\_TAEpaeTARAAPEI-5, Unigene12921\_TAEpaeTARAAPEI-5, Unigene12947\_TAEpaeTARAAPEI-5, Unigene13000\_TAEpaeTARAAPEI-5, Unigene13055\_TAEpaeTARAAPEI-5, Unigene13082\_TAEpaeTARAAPEI-5, Unigene13115\_TAEpaeTARAAPEI-5, Unigene13163\_TAEpaeTARAAPEI-5, Unigene13168\_TAEpaeTARAAPEI-5, Unigene13361\_TAEpaeTARAAPEI-5, Unigene13484\_TAEpaeTARAAPEI-5, Unigene13532\_TAEpaeTARAAPEI-5, Unigene13629\_TAEpaeTARAAPEI-5, Unigene13637\_TAEpaeTARAAPEI-5, Unigene13666\_TAEpaeTARAAPEI-5, Unigene13858\_TAEpaeTARAAPEI-5, Unigene13968\_TAEpaeTARAAPEI-5, Unigene14180\_TAEpaeTARAAPEI-5, Unigene14270\_TAEpaeTARAAPEI-5, Unigene14362\_TAEpaeTARAAPEI-5, Unigene14915\_TAEpaeTARAAPEI-5, Unigene15120\_TAEpaeTARAAPEI-5, Unigene15361\_TAEpaeTARAAPEI-5, Unigene15484\_TAEpaeTARAAPEI-5, Unigene15549\_TAEpaeTARAAPEI-5, Unigene15615\_TAEpaeTARAAPEI-5, Unigene15620\_TAEpaeTARAAPEI-5, Unigene15835\_TAEpaeTARAAPEI-5, Unigene15881\_TAEpaeTARAAPEI-5, Unigene1593\_TAEpaeTARAAPEI-5, Unigene15971\_TAEpaeTARAAPEI-5, Unigene16002\_TAEpaeTARAAPEI-5, Unigene16244\_TAEpaeTARAAPEI-5, Unigene16406\_TAEpaeTARAAPEI-5, Unigene16411\_TAEpaeTARAAPEI-5, Unigene16420\_TAEpaeTARAAPEI-5, Unigene16468\_TAEpaeTARAAPEI-5, Unigene16712\_TAEpaeTARAAPEI-5, Unigene17012\_TAEpaeTARAAPEI-5, Unigene17109\_TAEpaeTARAAPEI-5, Unigene17114\_TAEpaeTARAAPEI-5, Unigene1712\_TAEpaeTARAAPEI-5, Unigene17145\_TAEpaeTARAAPEI-5, Unigene17354\_TAEpaeTARAAPEI-5, Unigene17420\_TAEpaeTARAAPEI-5, Unigene17425\_TAEpaeTARAAPEI-5, Unigene17551\_TAEpaeTARAAPEI-5, Unigene17673\_TAEpaeTARAAPEI-5, Unigene17687\_TAEpaeTARAAPEI-5, Unigene17700\_TAEpaeTARAAPEI-5, Unigene17731\_TAEpaeTARAAPEI-5, Unigene17741\_TAEpaeTARAAPEI-5, Unigene17819\_TAEpaeTARAAPEI-5, Unigene17832\_TAEpaeTARAAPEI-5, Unigene17844\_TAEpaeTARAAPEI-5, Unigene17865\_TAEpaeTARAAPEI-5, Unigene1786\_TAEpaeTARAAPEI-5, Unigene17901\_TAEpaeTARAAPEI-5, Unigene17934\_TAEpaeTARAAPEI-5, Unigene17977\_TAEpaeTARAAPEI-5, Unigene17999\_TAEpaeTARAAPEI-5, Unigene18027\_TAEpaeTARAAPEI-5, Unigene18309\_TAEpaeTARAAPEI-5, Unigene19248\_TAEpaeTARAAPEI-5, Unigene19396\_TAEpaeTARAAPEI-5, Unigene19465\_TAEpaeTARAAPEI-5, Unigene19746\_TAEpaeTARAAPEI-5, Unigene19798\_TAEpaeTARAAPEI-5, Unigene19985\_TAEpaeTARAAPEI-5, Unigene20000\_TAEpaeTARAAPEI-5, Unigene20093\_TAEpaeTARAAPEI-5, Unigene20955\_TAEpaeTARAAPEI-5, Unigene21335\_TAEpaeTARAAPEI-5, Unigene21513\_TAEpaeTARAAPEI-5, Unigene21538\_TAEpaeTARAAPEI-5, Unigene21878\_TAEpaeTARAAPEI-5, Unigene21891\_TAEpaeTARAAPEI-5, Unigene2206\_TAEpaeTARAAPEI-5, Unigene22358\_TAEpaeTARAAPEI-5, Unigene22554\_TAEpaeTARAAPEI-5, Unigene22839\_TAEpaeTARAAPEI-5, Unigene2340\_TAEpaeTARAAPEI-5, Unigene23436\_TAEpaeTARAAPEI-5, Unigene23485\_TAEpaeTARAAPEI-5, Unigene2429\_TAEpaeTARAAPEI-5, Unigene2439\_TAEpaeTARAAPEI-5, Unigene24538\_TAEpaeTARAAPEI-5, Unigene24741\_TAEpaeTARAAPEI-5, Unigene25334\_TAEpaeTARAAPEI-5, Unigene253\_TAEpaeTARAAPEI-5, Unigene25970\_TAEpaeTARAAPEI-5, Unigene26031\_TAEpaeTARAAPEI-5, Unigene26250\_TAEpaeTARAAPEI-5, Unigene26309\_TAEpaeTARAAPEI-5, Unigene26359\_TAEpaeTARAAPEI-5, Unigene26365\_TAEpaeTARAAPEI-5, Unigene26514\_TAEpaeTARAAPEI-5, Unigene27882\_TAEpaeTARAAPEI-5, Unigene27972\_TAEpaeTARAAPEI-5, Unigene28721\_TAEpaeTARAAPEI-5, Unigene28826\_TAEpaeTARAAPEI-5, Unigene2889\_TAEpaeTARAAPEI-5, Unigene29171\_TAEpaeTARAAPEI-5, Unigene29466\_TAEpaeTARAAPEI-5, Unigene29748\_TAEpaeTARAAPEI-5, Unigene29887\_TAEpaeTARAAPEI-5, Unigene29929\_TAEpaeTARAAPEI-5, Unigene3004\_TAEpaeTARAAPEI-5, Unigene30172\_TAEpaeTARAAPEI-5, Unigene30199\_TAEpaeTARAAPEI-5, Unigene30758\_TAEpaeTARAAPEI-5, Unigene30803\_TAEpaeTARAAPEI-5, Unigene30885\_TAEpaeTARAAPEI-5, Unigene30916\_TAEpaeTARAAPEI-5, Unigene31547\_TAEpaeTARAAPEI-5, Unigene3158\_TAEpaeTARAAPEI-5, Unigene31750\_TAEpaeTARAAPEI-5, Unigene32104\_TAEpaeTARAAPEI-5, Unigene32294\_TAEpaeTARAAPEI-5, Unigene32639\_TAEpaeTARAAPEI-5, Unigene32711\_TAEpaeTARAAPEI-5, Unigene32748\_TAEpaeTARAAPEI-5, Unigene32782\_TAEpaeTARAAPEI-5, Unigene32999\_TAEpaeTARAAPEI-5, Unigene33030\_TAEpaeTARAAPEI-5, Unigene33057\_TAEpaeTARAAPEI-5, Unigene33174\_TAEpaeTARAAPEI-5, Unigene33321\_TAEpaeTARAAPEI-5, Unigene33880\_TAEpaeTARAAPEI-5, Unigene34144\_TAEpaeTARAAPEI-5, Unigene34267\_TAEpaeTARAAPEI-5, Unigene34331\_TAEpaeTARAAPEI-5, Unigene34523\_TAEpaeTARAAPEI-5, Unigene34847\_TAEpaeTARAAPEI-5, Unigene35030\_TAEpaeTARAAPEI-5, Unigene35102\_TAEpaeTARAAPEI-5, Unigene35688\_TAEpaeTARAAPEI-5, Unigene3582\_TAEpaeTARAAPEI-5, Unigene35882\_TAEpaeTARAAPEI-5, Unigene35973\_TAEpaeTARAAPEI-5, Unigene36194\_TAEpaeTARAAPEI-5, Unigene36424\_TAEpaeTARAAPEI-5, Unigene36648\_TAEpaeTARAAPEI-5, Unigene36858\_TAEpaeTARAAPEI-5, Unigene37038\_TAEpaeTARAAPEI-5, Unigene37058\_TAEpaeTARAAPEI-5, Unigene3737\_TAEpaeTARAAPEI-5, Unigene37724\_TAEpaeTARAAPEI-5, Unigene37855\_TAEpaeTARAAPEI-5, Unigene37980\_TAEpaeTARAAPEI-5, Unigene37986\_TAEpaeTARAAPEI-5, Unigene38228\_TAEpaeTARAAPEI-5, Unigene38258\_TAEpaeTARAAPEI-5, Unigene3828\_TAEpaeTARAAPEI-5, Unigene38485\_TAEpaeTARAAPEI-5, Unigene38667\_TAEpaeTARAAPEI-5, Unigene38738\_TAEpaeTARAAPEI-5, Unigene38891\_TAEpaeTARAAPEI-5, Unigene38997\_TAEpaeTARAAPEI-5, Unigene39227\_TAEpaeTARAAPEI-5, Unigene39254\_TAEpaeTARAAPEI-5, Unigene39376\_TAEpaeTARAAPEI-5, Unigene39638\_TAEpaeTARAAPEI-5, Unigene39705\_TAEpaeTARAAPEI-5, Unigene39872\_TAEpaeTARAAPEI-5, Unigene39874\_TAEpaeTARAAPEI-5, Unigene39926\_TAEpaeTARAAPEI-5, Unigene39939\_TAEpaeTARAAPEI-5, Unigene40279\_TAEpaeTARAAPEI-5, Unigene40763\_TAEpaeTARAAPEI-5, Unigene40775\_TAEpaeTARAAPEI-5, Unigene40826\_TAEpaeTARAAPEI-5, Unigene40931\_TAEpaeTARAAPEI-5, Unigene40999\_TAEpaeTARAAPEI-5, Unigene41157\_TAEpaeTARAAPEI-5, Unigene41673\_TAEpaeTARAAPEI-5, Unigene41868\_TAEpaeTARAAPEI-5, Unigene42325\_TAEpaeTARAAPEI-5, Unigene42414\_TAEpaeTARAAPEI-5, Unigene42424\_TAEpaeTARAAPEI-5, Unigene42443\_TAEpaeTARAAPEI-5, Unigene42725\_TAEpaeTARAAPEI-5, Unigene43076\_TAEpaeTARAAPEI-5, Unigene43326\_TAEpaeTARAAPEI-5, Unigene4380\_TAEpaeTARAAPEI-5, Unigene43926\_TAEpaeTARAAPEI-5, Unigene44127\_TAEpaeTARAAPEI-5, Unigene44209\_TAEpaeTARAAPEI-5, Unigene4423\_TAEpaeTARAAPEI-5, Unigene44324\_TAEpaeTARAAPEI-5, Unigene44401\_TAEpaeTARAAPEI-5, Unigene44598\_TAEpaeTARAAPEI-5, Unigene44963\_TAEpaeTARAAPEI-5, Unigene45049\_TAEpaeTARAAPEI-5, Unigene45239\_TAEpaeTARAAPEI-5, Unigene45410\_TAEpaeTARAAPEI-5, Unigene45435\_TAEpaeTARAAPEI-5, Unigene45451\_TAEpaeTARAAPEI-5, Unigene45510\_TAEpaeTARAAPEI-5, Unigene45602\_TAEpaeTARAAPEI-5, Unigene45651\_TAEpaeTARAAPEI-5, Unigene45682\_TAEpaeTARAAPEI-5, Unigene45779\_TAEpaeTARAAPEI-5, Unigene45838\_TAEpaeTARAAPEI-5, Unigene45969\_TAEpaeTARAAPEI-5, Unigene4605\_TAEpaeTARAAPEI-5, Unigene46118\_TAEpaeTARAAPEI-5, Unigene46461\_TAEpaeTARAAPEI-5, Unigene46807\_TAEpaeTARAAPEI-5, Unigene46978\_TAEpaeTARAAPEI-5, Unigene47023\_TAEpaeTARAAPEI-5, Unigene47386\_TAEpaeTARAAPEI-5, Unigene47434\_TAEpaeTARAAPEI-5, Unigene47449\_TAEpaeTARAAPEI-5, Unigene47595\_TAEpaeTARAAPEI-5, Unigene48038\_TAEpaeTARAAPEI-5, Unigene48238\_TAEpaeTARAAPEI-5, Unigene48332\_TAEpaeTARAAPEI-5, Unigene48335\_TAEpaeTARAAPEI-5, Unigene48356\_TAEpaeTARAAPEI-5, Unigene48394\_TAEpaeTARAAPEI-5, Unigene48636\_TAEpaeTARAAPEI-5, Unigene48689\_TAEpaeTARAAPEI-5, Unigene48705\_TAEpaeTARAAPEI-5, Unigene48840\_TAEpaeTARAAPEI-5, Unigene4903\_TAEpaeTARAAPEI-5, Unigene49085\_TAEpaeTARAAPEI-5, Unigene49114\_TAEpaeTARAAPEI-5, Unigene49966\_TAEpaeTARAAPEI-5, Unigene49981\_TAEpaeTARAAPEI-5, Unigene50851\_TAEpaeTARAAPEI-5, Unigene51260\_TAEpaeTARAAPEI-5, Unigene5145\_TAEpaeTARAAPEI-5, Unigene51640\_TAEpaeTARAAPEI-5, Unigene51664\_TAEpaeTARAAPEI-5, Unigene51722\_TAEpaeTARAAPEI-5, Unigene51767\_TAEpaeTARAAPEI-5, Unigene51791\_TAEpaeTARAAPEI-5, Unigene51813\_TAEpaeTARAAPEI-5, Unigene51987\_TAEpaeTARAAPEI-5, Unigene5224\_TAEpaeTARAAPEI-5, Unigene5248\_TAEpaeTARAAPEI-5, Unigene52546\_TAEpaeTARAAPEI-5, Unigene52867\_TAEpaeTARAAPEI-5, Unigene53062\_TAEpaeTARAAPEI-5, Unigene53203\_TAEpaeTARAAPEI-5, Unigene53446\_TAEpaeTARAAPEI-5, Unigene53786\_TAEpaeTARAAPEI-5, Unigene54313\_TAEpaeTARAAPEI-5, Unigene54363\_TAEpaeTARAAPEI-5, Unigene54526\_TAEpaeTARAAPEI-5, Unigene54702\_TAEpaeTARAAPEI-5, Unigene54858\_TAEpaeTARAAPEI-5, Unigene55238\_TAEpaeTARAAPEI-5, Unigene55256\_TAEpaeTARAAPEI-5, Unigene55396\_TAEpaeTARAAPEI-5, Unigene5555\_TAEpaeTARAAPEI-5, Unigene55602\_TAEpaeTARAAPEI-5, Unigene55789\_TAEpaeTARAAPEI-5, Unigene55845\_TAEpaeTARAAPEI-5, Unigene56185\_TAEpaeTARAAPEI-5, Unigene56402\_TAEpaeTARAAPEI-5, Unigene56490\_TAEpaeTARAAPEI-5, Unigene5655\_TAEpaeTARAAPEI-5, Unigene56576\_TAEpaeTARAAPEI-5, Unigene56725\_TAEpaeTARAAPEI-5, Unigene56770\_TAEpaeTARAAPEI-5, Unigene5683\_TAEpaeTARAAPEI-5, Unigene56915\_TAEpaeTARAAPEI-5, Unigene57076\_TAEpaeTARAAPEI-5, Unigene57161\_TAEpaeTARAAPEI-5, Unigene57224\_TAEpaeTARAAPEI-5, Unigene57376\_TAEpaeTARAAPEI-5, Unigene57542\_TAEpaeTARAAPEI-5, Unigene57610\_TAEpaeTARAAPEI-5, Unigene57935\_TAEpaeTARAAPEI-5, Unigene57969\_TAEpaeTARAAPEI-5, Unigene58297\_TAEpaeTARAAPEI-5, Unigene58436\_TAEpaeTARAAPEI-5, Unigene5855\_TAEpaeTARAAPEI-5, Unigene58626\_TAEpaeTARAAPEI-5, Unigene58630\_TAEpaeTARAAPEI-5, Unigene5872\_TAEpaeTARAAPEI-5, Unigene58807\_TAEpaeTARAAPEI-5, Unigene58808\_TAEpaeTARAAPEI-5, Unigene58848\_TAEpaeTARAAPEI-5, Unigene59053\_TAEpaeTARAAPEI-5, Unigene59452\_TAEpaeTARAAPEI-5, Unigene59571\_TAEpaeTARAAPEI-5, Unigene59597\_TAEpaeTARAAPEI-5, Unigene59670\_TAEpaeTARAAPEI-5, Unigene59674\_TAEpaeTARAAPEI-5, Unigene6030\_TAEpaeTARAAPEI-5, Unigene60330\_TAEpaeTARAAPEI-5, Unigene60344\_TAEpaeTARAAPEI-5, Unigene60374\_TAEpaeTARAAPEI-5, Unigene60489\_TAEpaeTARAAPEI-5, Unigene60986\_TAEpaeTARAAPEI-5, Unigene61029\_TAEpaeTARAAPEI-5, Unigene61114\_TAEpaeTARAAPEI-5, Unigene61147\_TAEpaeTARAAPEI-5, Unigene61351\_TAEpaeTARAAPEI-5, Unigene61367\_TAEpaeTARAAPEI-5, Unigene6150\_TAEpaeTARAAPEI-5, Unigene61561\_TAEpaeTARAAPEI-5, Unigene61806\_TAEpaeTARAAPEI-5, Unigene61820\_TAEpaeTARAAPEI-5, Unigene61823\_TAEpaeTARAAPEI-5, Unigene62056\_TAEpaeTARAAPEI-5, Unigene62064\_TAEpaeTARAAPEI-5, Unigene62217\_TAEpaeTARAAPEI-5, Unigene62275\_TAEpaeTARAAPEI-5, Unigene62363\_TAEpaeTARAAPEI-5, Unigene6262\_TAEpaeTARAAPEI-5, Unigene62658\_TAEpaeTARAAPEI-5, Unigene62742\_TAEpaeTARAAPEI-5, Unigene62920\_TAEpaeTARAAPEI-5, Unigene62962\_TAEpaeTARAAPEI-5, Unigene63015\_TAEpaeTARAAPEI-5, Unigene63027\_TAEpaeTARAAPEI-5, Unigene63056\_TAEpaeTARAAPEI-5, Unigene63066\_TAEpaeTARAAPEI-5, Unigene63077\_TAEpaeTARAAPEI-5, Unigene63148\_TAEpaeTARAAPEI-5, Unigene63280\_TAEpaeTARAAPEI-5, Unigene63311\_TAEpaeTARAAPEI-5, Unigene63425\_TAEpaeTARAAPEI-5, Unigene63468\_TAEpaeTARAAPEI-5, Unigene63499\_TAEpaeTARAAPEI-5, Unigene63522\_TAEpaeTARAAPEI-5, Unigene63532\_TAEpaeTARAAPEI-5, Unigene63705\_TAEpaeTARAAPEI-5, Unigene63960\_TAEpaeTARAAPEI-5, Unigene63980\_TAEpaeTARAAPEI-5, Unigene64029\_TAEpaeTARAAPEI-5, Unigene64040\_TAEpaeTARAAPEI-5, Unigene64293\_TAEpaeTARAAPEI-5, Unigene64361\_TAEpaeTARAAPEI-5, Unigene64373\_TAEpaeTARAAPEI-5, Unigene64401\_TAEpaeTARAAPEI-5, Unigene64455\_TAEpaeTARAAPEI-5, Unigene64468\_TAEpaeTARAAPEI-5, Unigene64529\_TAEpaeTARAAPEI-5, Unigene64687\_TAEpaeTARAAPEI-5, Unigene64787\_TAEpaeTARAAPEI-5, Unigene64824\_TAEpaeTARAAPEI-5, Unigene65011\_TAEpaeTARAAPEI-5, Unigene65037\_TAEpaeTARAAPEI-5, Unigene65047\_TAEpaeTARAAPEI-5, Unigene65314\_TAEpaeTARAAPEI-5, Unigene65318\_TAEpaeTARAAPEI-5, Unigene65322\_TAEpaeTARAAPEI-5, Unigene65517\_TAEpaeTARAAPEI-5, Unigene65523\_TAEpaeTARAAPEI-5, Unigene65530\_TAEpaeTARAAPEI-5, Unigene65570\_TAEpaeTARAAPEI-5, Unigene65593\_TAEpaeTARAAPEI-5, Unigene65685\_TAEpaeTARAAPEI-5, Unigene65820\_TAEpaeTARAAPEI-5, Unigene65885\_TAEpaeTARAAPEI-5, Unigene65960\_TAEpaeTARAAPEI-5, Unigene65964\_TAEpaeTARAAPEI-5, Unigene66043\_TAEpaeTARAAPEI-5, Unigene66058\_TAEpaeTARAAPEI-5, Unigene6605\_TAEpaeTARAAPEI-5, Unigene66212\_TAEpaeTARAAPEI-5, Unigene66288\_TAEpaeTARAAPEI-5, Unigene66291\_TAEpaeTARAAPEI-5, Unigene66352\_TAEpaeTARAAPEI-5, Unigene66459\_TAEpaeTARAAPEI-5, Unigene66683\_TAEpaeTARAAPEI-5, Unigene66694\_TAEpaeTARAAPEI-5, Unigene66800\_TAEpaeTARAAPEI-5, Unigene66931\_TAEpaeTARAAPEI-5, Unigene66965\_TAEpaeTARAAPEI-5, Unigene67006\_TAEpaeTARAAPEI-5, Unigene67063\_TAEpaeTARAAPEI-5, Unigene67065\_TAEpaeTARAAPEI-5, Unigene67066\_TAEpaeTARAAPEI-5, Unigene67165\_TAEpaeTARAAPEI-5, Unigene67196\_TAEpaeTARAAPEI-5, Unigene67288\_TAEpaeTARAAPEI-5, Unigene67341\_TAEpaeTARAAPEI-5, Unigene67524\_TAEpaeTARAAPEI-5, Unigene67660\_TAEpaeTARAAPEI-5, Unigene67663\_TAEpaeTARAAPEI-5, Unigene67745\_TAEpaeTARAAPEI-5, Unigene67748\_TAEpaeTARAAPEI-5, Unigene67781\_TAEpaeTARAAPEI-5, Unigene67786\_TAEpaeTARAAPEI-5, Unigene67829\_TAEpaeTARAAPEI-5, Unigene67884\_TAEpaeTARAAPEI-5, Unigene67889\_TAEpaeTARAAPEI-5, Unigene67982\_TAEpaeTARAAPEI-5, Unigene68001\_TAEpaeTARAAPEI-5, Unigene68071\_TAEpaeTARAAPEI-5, Unigene68103\_TAEpaeTARAAPEI-5, Unigene68184\_TAEpaeTARAAPEI-5, Unigene68189\_TAEpaeTARAAPEI-5, Unigene68258\_TAEpaeTARAAPEI-5, Unigene68274\_TAEpaeTARAAPEI-5, Unigene68507\_TAEpaeTARAAPEI-5, Unigene68625\_TAEpaeTARAAPEI-5, Unigene68643\_TAEpaeTARAAPEI-5, Unigene68686\_TAEpaeTARAAPEI-5, Unigene68694\_TAEpaeTARAAPEI-5, Unigene68710\_TAEpaeTARAAPEI-5, Unigene68845\_TAEpaeTARAAPEI-5, Unigene68874\_TAEpaeTARAAPEI-5, Unigene68984\_TAEpaeTARAAPEI-5, Unigene68998\_TAEpaeTARAAPEI-5, Unigene69030\_TAEpaeTARAAPEI-5, Unigene69033\_TAEpaeTARAAPEI-5, Unigene69062\_TAEpaeTARAAPEI-5, Unigene69164\_TAEpaeTARAAPEI-5, Unigene69189\_TAEpaeTARAAPEI-5, Unigene69220\_TAEpaeTARAAPEI-5, Unigene69275\_TAEpaeTARAAPEI-5, Unigene69311\_TAEpaeTARAAPEI-5, Unigene69370\_TAEpaeTARAAPEI-5, Unigene69372\_TAEpaeTARAAPEI-5, Unigene6938\_TAEpaeTARAAPEI-5, Unigene6940\_TAEpaeTARAAPEI-5, Unigene69421\_TAEpaeTARAAPEI-5, Unigene69441\_TAEpaeTARAAPEI-5, Unigene69489\_TAEpaeTARAAPEI-5, Unigene69492\_TAEpaeTARAAPEI-5, Unigene69552\_TAEpaeTARAAPEI-5, Unigene69558\_TAEpaeTARAAPEI-5, Unigene69575\_TAEpaeTARAAPEI-5, Unigene69579\_TAEpaeTARAAPEI-5, Unigene69670\_TAEpaeTARAAPEI-5, Unigene69674\_TAEpaeTARAAPEI-5, Unigene69700\_TAEpaeTARAAPEI-5, Unigene69705\_TAEpaeTARAAPEI-5, Unigene69730\_TAEpaeTARAAPEI-5, Unigene69773\_TAEpaeTARAAPEI-5, Unigene69776\_TAEpaeTARAAPEI-5, Unigene69849\_TAEpaeTARAAPEI-5, Unigene69900\_TAEpaeTARAAPEI-5, Unigene69964\_TAEpaeTARAAPEI-5, Unigene69987\_TAEpaeTARAAPEI-5, Unigene70033\_TAEpaeTARAAPEI-5, Unigene70046\_TAEpaeTARAAPEI-5, Unigene70061\_TAEpaeTARAAPEI-5, Unigene70074\_TAEpaeTARAAPEI-5, Unigene70103\_TAEpaeTARAAPEI-5, Unigene70104\_TAEpaeTARAAPEI-5, Unigene7011\_TAEpaeTARAAPEI-5, Unigene70176\_TAEpaeTARAAPEI-5, Unigene70200\_TAEpaeTARAAPEI-5, Unigene70303\_TAEpaeTARAAPEI-5, Unigene70316\_TAEpaeTARAAPEI-5, Unigene70372\_TAEpaeTARAAPEI-5, Unigene70434\_TAEpaeTARAAPEI-5, Unigene7046\_TAEpaeTARAAPEI-5, Unigene70477\_TAEpaeTARAAPEI-5, Unigene70533\_TAEpaeTARAAPEI-5, Unigene70579\_TAEpaeTARAAPEI-5, Unigene70607\_TAEpaeTARAAPEI-5, Unigene70615\_TAEpaeTARAAPEI-5, Unigene70711\_TAEpaeTARAAPEI-5, Unigene70761\_TAEpaeTARAAPEI-5, Unigene70825\_TAEpaeTARAAPEI-5, Unigene70941\_TAEpaeTARAAPEI-5, Unigene70966\_TAEpaeTARAAPEI-5, Unigene70977\_TAEpaeTARAAPEI-5, Unigene71182\_TAEpaeTARAAPEI-5, Unigene71305\_TAEpaeTARAAPEI-5, Unigene71310\_TAEpaeTARAAPEI-5, Unigene71460\_TAEpaeTARAAPEI-5, Unigene71608\_TAEpaeTARAAPEI-5, Unigene71617\_TAEpaeTARAAPEI-5, Unigene71621\_TAEpaeTARAAPEI-5, Unigene71696\_TAEpaeTARAAPEI-5, Unigene71719\_TAEpaeTARAAPEI-5, Unigene71752\_TAEpaeTARAAPEI-5, Unigene71835\_TAEpaeTARAAPEI-5, Unigene72087\_TAEpaeTARAAPEI-5, Unigene72092\_TAEpaeTARAAPEI-5, Unigene72181\_TAEpaeTARAAPEI-5, Unigene72213\_TAEpaeTARAAPEI-5, Unigene72266\_TAEpaeTARAAPEI-5, Unigene72319\_TAEpaeTARAAPEI-5, Unigene72322\_TAEpaeTARAAPEI-5, Unigene72337\_TAEpaeTARAAPEI-5, Unigene72340\_TAEpaeTARAAPEI-5, Unigene72355\_TAEpaeTARAAPEI-5, Unigene72392\_TAEpaeTARAAPEI-5, Unigene72414\_TAEpaeTARAAPEI-5, Unigene72473\_TAEpaeTARAAPEI-5, Unigene72501\_TAEpaeTARAAPEI-5, Unigene72540\_TAEpaeTARAAPEI-5, Unigene72588\_TAEpaeTARAAPEI-5, Unigene72635\_TAEpaeTARAAPEI-5, Unigene72665\_TAEpaeTARAAPEI-5, Unigene72687\_TAEpaeTARAAPEI-5, Unigene72690\_TAEpaeTARAAPEI-5, Unigene72707\_TAEpaeTARAAPEI-5, Unigene72743\_TAEpaeTARAAPEI-5, Unigene72768\_TAEpaeTARAAPEI-5, Unigene72823\_TAEpaeTARAAPEI-5, Unigene72826\_TAEpaeTARAAPEI-5, Unigene72849\_TAEpaeTARAAPEI-5, Unigene72896\_TAEpaeTARAAPEI-5, Unigene72897\_TAEpaeTARAAPEI-5, Unigene72903\_TAEpaeTARAAPEI-5, Unigene7328\_TAEpaeTARAAPEI-5, Unigene7562\_TAEpaeTARAAPEI-5, Unigene7656\_TAEpaeTARAAPEI-5, Unigene8100\_TAEpaeTARAAPEI-5, Unigene8507\_TAEpaeTARAAPEI-5, Unigene8562\_TAEpaeTARAAPEI-5, Unigene8666\_TAEpaeTARAAPEI-5, Unigene8876\_TAEpaeTARAAPEI-5, Unigene9072\_TAEpaeTARAAPEI-5, Unigene907\_TAEpaeTARAAPEI-5, Unigene9135\_TAEpaeTARAAPEI-5, Unigene9304\_TAEpaeTARAAPEI-5, Unigene9723\_TAEpaeTARAAPEI-5, Unigene9731\_TAEpaeTARAAPEI-5, Unigene9945\_TAEpaeTARAAPEI-5 |
| 8 | Endocytosis | Unigene10010\_TAEpaeTARAAPEI-5, Unigene10068\_TAEpaeTARAAPEI-5, Unigene10177\_TAEpaeTARAAPEI-5, Unigene10294\_TAEpaeTARAAPEI-5, Unigene10320\_TAEpaeTARAAPEI-5, Unigene10358\_TAEpaeTARAAPEI-5, Unigene10539\_TAEpaeTARAAPEI-5, Unigene10694\_TAEpaeTARAAPEI-5, Unigene10716\_TAEpaeTARAAPEI-5, Unigene10794\_TAEpaeTARAAPEI-5, Unigene10869\_TAEpaeTARAAPEI-5, Unigene11020\_TAEpaeTARAAPEI-5, Unigene11123\_TAEpaeTARAAPEI-5, Unigene11482\_TAEpaeTARAAPEI-5, Unigene11841\_TAEpaeTARAAPEI-5, Unigene11984\_TAEpaeTARAAPEI-5, Unigene12075\_TAEpaeTARAAPEI-5, Unigene12361\_TAEpaeTARAAPEI-5, Unigene12432\_TAEpaeTARAAPEI-5, Unigene1244\_TAEpaeTARAAPEI-5, Unigene1274\_TAEpaeTARAAPEI-5, Unigene13090\_TAEpaeTARAAPEI-5, Unigene13121\_TAEpaeTARAAPEI-5, Unigene13163\_TAEpaeTARAAPEI-5, Unigene13228\_TAEpaeTARAAPEI-5, Unigene13238\_TAEpaeTARAAPEI-5, Unigene1327\_TAEpaeTARAAPEI-5, Unigene13361\_TAEpaeTARAAPEI-5, Unigene13487\_TAEpaeTARAAPEI-5, Unigene13498\_TAEpaeTARAAPEI-5, Unigene13901\_TAEpaeTARAAPEI-5, Unigene13975\_TAEpaeTARAAPEI-5, Unigene14069\_TAEpaeTARAAPEI-5, Unigene14180\_TAEpaeTARAAPEI-5, Unigene14226\_TAEpaeTARAAPEI-5, Unigene14481\_TAEpaeTARAAPEI-5, Unigene14566\_TAEpaeTARAAPEI-5, Unigene14791\_TAEpaeTARAAPEI-5, Unigene14855\_TAEpaeTARAAPEI-5, Unigene14935\_TAEpaeTARAAPEI-5, Unigene14943\_TAEpaeTARAAPEI-5, Unigene15075\_TAEpaeTARAAPEI-5, Unigene15334\_TAEpaeTARAAPEI-5, Unigene15486\_TAEpaeTARAAPEI-5, Unigene15610\_TAEpaeTARAAPEI-5, Unigene15654\_TAEpaeTARAAPEI-5, Unigene15785\_TAEpaeTARAAPEI-5, Unigene1579\_TAEpaeTARAAPEI-5, Unigene15832\_TAEpaeTARAAPEI-5, Unigene15833\_TAEpaeTARAAPEI-5, Unigene15865\_TAEpaeTARAAPEI-5, Unigene15945\_TAEpaeTARAAPEI-5, Unigene15987\_TAEpaeTARAAPEI-5, Unigene16002\_TAEpaeTARAAPEI-5, Unigene16311\_TAEpaeTARAAPEI-5, Unigene16406\_TAEpaeTARAAPEI-5, Unigene16411\_TAEpaeTARAAPEI-5, Unigene16515\_TAEpaeTARAAPEI-5, Unigene16842\_TAEpaeTARAAPEI-5, Unigene16950\_TAEpaeTARAAPEI-5, Unigene16986\_TAEpaeTARAAPEI-5, Unigene17250\_TAEpaeTARAAPEI-5, Unigene17364\_TAEpaeTARAAPEI-5, Unigene17475\_TAEpaeTARAAPEI-5, Unigene17585\_TAEpaeTARAAPEI-5, Unigene17607\_TAEpaeTARAAPEI-5, Unigene17680\_TAEpaeTARAAPEI-5, Unigene17689\_TAEpaeTARAAPEI-5, Unigene17795\_TAEpaeTARAAPEI-5, Unigene17833\_TAEpaeTARAAPEI-5, Unigene17868\_TAEpaeTARAAPEI-5, Unigene17901\_TAEpaeTARAAPEI-5, Unigene17984\_TAEpaeTARAAPEI-5, Unigene18030\_TAEpaeTARAAPEI-5, Unigene18082\_TAEpaeTARAAPEI-5, Unigene18085\_TAEpaeTARAAPEI-5, Unigene180\_TAEpaeTARAAPEI-5, Unigene1844\_TAEpaeTARAAPEI-5, Unigene18797\_TAEpaeTARAAPEI-5, Unigene19248\_TAEpaeTARAAPEI-5, Unigene19332\_TAEpaeTARAAPEI-5, Unigene19798\_TAEpaeTARAAPEI-5, Unigene19823\_TAEpaeTARAAPEI-5, Unigene20146\_TAEpaeTARAAPEI-5, Unigene20248\_TAEpaeTARAAPEI-5, Unigene2034\_TAEpaeTARAAPEI-5, Unigene21780\_TAEpaeTARAAPEI-5, Unigene21846\_TAEpaeTARAAPEI-5, Unigene21960\_TAEpaeTARAAPEI-5, Unigene2206\_TAEpaeTARAAPEI-5, Unigene22264\_TAEpaeTARAAPEI-5, Unigene22273\_TAEpaeTARAAPEI-5, Unigene22526\_TAEpaeTARAAPEI-5, Unigene22898\_TAEpaeTARAAPEI-5, Unigene23004\_TAEpaeTARAAPEI-5, Unigene23209\_TAEpaeTARAAPEI-5, Unigene23210\_TAEpaeTARAAPEI-5, Unigene23318\_TAEpaeTARAAPEI-5, Unigene23365\_TAEpaeTARAAPEI-5, Unigene23386\_TAEpaeTARAAPEI-5, Unigene24306\_TAEpaeTARAAPEI-5, Unigene24316\_TAEpaeTARAAPEI-5, Unigene2476\_TAEpaeTARAAPEI-5, Unigene24825\_TAEpaeTARAAPEI-5, Unigene25039\_TAEpaeTARAAPEI-5, Unigene2508\_TAEpaeTARAAPEI-5, Unigene25405\_TAEpaeTARAAPEI-5, Unigene25487\_TAEpaeTARAAPEI-5, Unigene25681\_TAEpaeTARAAPEI-5, Unigene25831\_TAEpaeTARAAPEI-5, Unigene26108\_TAEpaeTARAAPEI-5, Unigene26882\_TAEpaeTARAAPEI-5, Unigene27023\_TAEpaeTARAAPEI-5, Unigene27205\_TAEpaeTARAAPEI-5, Unigene27408\_TAEpaeTARAAPEI-5, Unigene27477\_TAEpaeTARAAPEI-5, Unigene27704\_TAEpaeTARAAPEI-5, Unigene27715\_TAEpaeTARAAPEI-5, Unigene28269\_TAEpaeTARAAPEI-5, Unigene28526\_TAEpaeTARAAPEI-5, Unigene28828\_TAEpaeTARAAPEI-5, Unigene2897\_TAEpaeTARAAPEI-5, Unigene28\_TAEpaeTARAAPEI-5, Unigene29077\_TAEpaeTARAAPEI-5, Unigene29123\_TAEpaeTARAAPEI-5, Unigene29593\_TAEpaeTARAAPEI-5, Unigene29638\_TAEpaeTARAAPEI-5, Unigene29718\_TAEpaeTARAAPEI-5, Unigene29814\_TAEpaeTARAAPEI-5, Unigene29944\_TAEpaeTARAAPEI-5, Unigene3004\_TAEpaeTARAAPEI-5, Unigene31040\_TAEpaeTARAAPEI-5, Unigene31348\_TAEpaeTARAAPEI-5, Unigene31512\_TAEpaeTARAAPEI-5, Unigene31572\_TAEpaeTARAAPEI-5, Unigene31615\_TAEpaeTARAAPEI-5, Unigene31802\_TAEpaeTARAAPEI-5, Unigene31854\_TAEpaeTARAAPEI-5, Unigene31877\_TAEpaeTARAAPEI-5, Unigene32311\_TAEpaeTARAAPEI-5, Unigene32410\_TAEpaeTARAAPEI-5, Unigene32874\_TAEpaeTARAAPEI-5, Unigene33009\_TAEpaeTARAAPEI-5, Unigene33089\_TAEpaeTARAAPEI-5, Unigene33150\_TAEpaeTARAAPEI-5, Unigene33283\_TAEpaeTARAAPEI-5, Unigene33321\_TAEpaeTARAAPEI-5, Unigene33508\_TAEpaeTARAAPEI-5, Unigene33556\_TAEpaeTARAAPEI-5, Unigene33609\_TAEpaeTARAAPEI-5, Unigene33930\_TAEpaeTARAAPEI-5, Unigene34159\_TAEpaeTARAAPEI-5, Unigene34447\_TAEpaeTARAAPEI-5, Unigene34972\_TAEpaeTARAAPEI-5, Unigene35420\_TAEpaeTARAAPEI-5, Unigene35482\_TAEpaeTARAAPEI-5, Unigene35566\_TAEpaeTARAAPEI-5, Unigene36036\_TAEpaeTARAAPEI-5, Unigene36256\_TAEpaeTARAAPEI-5, Unigene36424\_TAEpaeTARAAPEI-5, Unigene36435\_TAEpaeTARAAPEI-5, Unigene36451\_TAEpaeTARAAPEI-5, Unigene36689\_TAEpaeTARAAPEI-5, Unigene36695\_TAEpaeTARAAPEI-5, Unigene37038\_TAEpaeTARAAPEI-5, Unigene370\_TAEpaeTARAAPEI-5, Unigene37192\_TAEpaeTARAAPEI-5, Unigene37667\_TAEpaeTARAAPEI-5, Unigene37708\_TAEpaeTARAAPEI-5, Unigene37735\_TAEpaeTARAAPEI-5, Unigene37856\_TAEpaeTARAAPEI-5, Unigene37893\_TAEpaeTARAAPEI-5, Unigene38225\_TAEpaeTARAAPEI-5, Unigene38287\_TAEpaeTARAAPEI-5, Unigene38433\_TAEpaeTARAAPEI-5, Unigene38444\_TAEpaeTARAAPEI-5, Unigene38744\_TAEpaeTARAAPEI-5, Unigene38891\_TAEpaeTARAAPEI-5, Unigene39221\_TAEpaeTARAAPEI-5, Unigene39224\_TAEpaeTARAAPEI-5, Unigene39287\_TAEpaeTARAAPEI-5, Unigene39344\_TAEpaeTARAAPEI-5, Unigene39640\_TAEpaeTARAAPEI-5, Unigene40262\_TAEpaeTARAAPEI-5, Unigene40363\_TAEpaeTARAAPEI-5, Unigene40499\_TAEpaeTARAAPEI-5, Unigene40921\_TAEpaeTARAAPEI-5, Unigene4095\_TAEpaeTARAAPEI-5, Unigene40962\_TAEpaeTARAAPEI-5, Unigene40999\_TAEpaeTARAAPEI-5, Unigene41114\_TAEpaeTARAAPEI-5, Unigene41393\_TAEpaeTARAAPEI-5, Unigene41475\_TAEpaeTARAAPEI-5, Unigene42317\_TAEpaeTARAAPEI-5, Unigene4231\_TAEpaeTARAAPEI-5, Unigene42443\_TAEpaeTARAAPEI-5, Unigene42589\_TAEpaeTARAAPEI-5, Unigene42612\_TAEpaeTARAAPEI-5, Unigene42835\_TAEpaeTARAAPEI-5, Unigene43051\_TAEpaeTARAAPEI-5, Unigene43070\_TAEpaeTARAAPEI-5, Unigene43137\_TAEpaeTARAAPEI-5, Unigene43451\_TAEpaeTARAAPEI-5, Unigene43703\_TAEpaeTARAAPEI-5, Unigene43926\_TAEpaeTARAAPEI-5, Unigene4396\_TAEpaeTARAAPEI-5, Unigene44127\_TAEpaeTARAAPEI-5, Unigene44209\_TAEpaeTARAAPEI-5, Unigene44258\_TAEpaeTARAAPEI-5, Unigene44328\_TAEpaeTARAAPEI-5, Unigene44434\_TAEpaeTARAAPEI-5, Unigene44445\_TAEpaeTARAAPEI-5, Unigene44456\_TAEpaeTARAAPEI-5, Unigene44633\_TAEpaeTARAAPEI-5, Unigene44641\_TAEpaeTARAAPEI-5, Unigene45046\_TAEpaeTARAAPEI-5, Unigene45562\_TAEpaeTARAAPEI-5, Unigene45758\_TAEpaeTARAAPEI-5, Unigene46343\_TAEpaeTARAAPEI-5, Unigene4660\_TAEpaeTARAAPEI-5, Unigene46978\_TAEpaeTARAAPEI-5, Unigene47105\_TAEpaeTARAAPEI-5, Unigene47312\_TAEpaeTARAAPEI-5, Unigene480\_TAEpaeTARAAPEI-5, Unigene48360\_TAEpaeTARAAPEI-5, Unigene48528\_TAEpaeTARAAPEI-5, Unigene4876\_TAEpaeTARAAPEI-5, Unigene48778\_TAEpaeTARAAPEI-5, Unigene48997\_TAEpaeTARAAPEI-5, Unigene49108\_TAEpaeTARAAPEI-5, Unigene49181\_TAEpaeTARAAPEI-5, Unigene49351\_TAEpaeTARAAPEI-5, Unigene49523\_TAEpaeTARAAPEI-5, Unigene49657\_TAEpaeTARAAPEI-5, Unigene49826\_TAEpaeTARAAPEI-5, Unigene50000\_TAEpaeTARAAPEI-5, Unigene5002\_TAEpaeTARAAPEI-5, Unigene50329\_TAEpaeTARAAPEI-5, Unigene50400\_TAEpaeTARAAPEI-5, Unigene50663\_TAEpaeTARAAPEI-5, Unigene51008\_TAEpaeTARAAPEI-5, Unigene51027\_TAEpaeTARAAPEI-5, Unigene5107\_TAEpaeTARAAPEI-5, Unigene51193\_TAEpaeTARAAPEI-5, Unigene51722\_TAEpaeTARAAPEI-5, Unigene51926\_TAEpaeTARAAPEI-5, Unigene51974\_TAEpaeTARAAPEI-5, Unigene52030\_TAEpaeTARAAPEI-5, Unigene52413\_TAEpaeTARAAPEI-5, Unigene52457\_TAEpaeTARAAPEI-5, Unigene5248\_TAEpaeTARAAPEI-5, Unigene52590\_TAEpaeTARAAPEI-5, Unigene52642\_TAEpaeTARAAPEI-5, Unigene52686\_TAEpaeTARAAPEI-5, Unigene52838\_TAEpaeTARAAPEI-5, Unigene52867\_TAEpaeTARAAPEI-5, Unigene53241\_TAEpaeTARAAPEI-5, Unigene53462\_TAEpaeTARAAPEI-5, Unigene53505\_TAEpaeTARAAPEI-5, Unigene53611\_TAEpaeTARAAPEI-5, Unigene54211\_TAEpaeTARAAPEI-5, Unigene54363\_TAEpaeTARAAPEI-5, Unigene54652\_TAEpaeTARAAPEI-5, Unigene54702\_TAEpaeTARAAPEI-5, Unigene54767\_TAEpaeTARAAPEI-5, Unigene55041\_TAEpaeTARAAPEI-5, Unigene55065\_TAEpaeTARAAPEI-5, Unigene55117\_TAEpaeTARAAPEI-5, Unigene55138\_TAEpaeTARAAPEI-5, Unigene5523\_TAEpaeTARAAPEI-5, Unigene55243\_TAEpaeTARAAPEI-5, Unigene55261\_TAEpaeTARAAPEI-5, Unigene55282\_TAEpaeTARAAPEI-5, Unigene55298\_TAEpaeTARAAPEI-5, Unigene55456\_TAEpaeTARAAPEI-5, Unigene55594\_TAEpaeTARAAPEI-5, Unigene55711\_TAEpaeTARAAPEI-5, Unigene5577\_TAEpaeTARAAPEI-5, Unigene55845\_TAEpaeTARAAPEI-5, Unigene55861\_TAEpaeTARAAPEI-5, Unigene55898\_TAEpaeTARAAPEI-5, Unigene55930\_TAEpaeTARAAPEI-5, Unigene55953\_TAEpaeTARAAPEI-5, Unigene56160\_TAEpaeTARAAPEI-5, Unigene56240\_TAEpaeTARAAPEI-5, Unigene56256\_TAEpaeTARAAPEI-5, Unigene56259\_TAEpaeTARAAPEI-5, Unigene56429\_TAEpaeTARAAPEI-5, Unigene56663\_TAEpaeTARAAPEI-5, Unigene56725\_TAEpaeTARAAPEI-5, Unigene56921\_TAEpaeTARAAPEI-5, Unigene5694\_TAEpaeTARAAPEI-5, Unigene57059\_TAEpaeTARAAPEI-5, Unigene5706\_TAEpaeTARAAPEI-5, Unigene57095\_TAEpaeTARAAPEI-5, Unigene57161\_TAEpaeTARAAPEI-5, Unigene5726\_TAEpaeTARAAPEI-5, Unigene57416\_TAEpaeTARAAPEI-5, Unigene57497\_TAEpaeTARAAPEI-5, Unigene57703\_TAEpaeTARAAPEI-5, Unigene57726\_TAEpaeTARAAPEI-5, Unigene57845\_TAEpaeTARAAPEI-5, Unigene58217\_TAEpaeTARAAPEI-5, Unigene58289\_TAEpaeTARAAPEI-5, Unigene58463\_TAEpaeTARAAPEI-5, Unigene58464\_TAEpaeTARAAPEI-5, Unigene58505\_TAEpaeTARAAPEI-5, Unigene58711\_TAEpaeTARAAPEI-5, Unigene58722\_TAEpaeTARAAPEI-5, Unigene59005\_TAEpaeTARAAPEI-5, Unigene59006\_TAEpaeTARAAPEI-5, Unigene59060\_TAEpaeTARAAPEI-5, Unigene59197\_TAEpaeTARAAPEI-5, Unigene59204\_TAEpaeTARAAPEI-5, Unigene59440\_TAEpaeTARAAPEI-5, Unigene59541\_TAEpaeTARAAPEI-5, Unigene59597\_TAEpaeTARAAPEI-5, Unigene59624\_TAEpaeTARAAPEI-5, Unigene59648\_TAEpaeTARAAPEI-5, Unigene59785\_TAEpaeTARAAPEI-5, Unigene59796\_TAEpaeTARAAPEI-5, Unigene59896\_TAEpaeTARAAPEI-5, Unigene60067\_TAEpaeTARAAPEI-5, Unigene60143\_TAEpaeTARAAPEI-5, Unigene60169\_TAEpaeTARAAPEI-5, Unigene60374\_TAEpaeTARAAPEI-5, Unigene60379\_TAEpaeTARAAPEI-5, Unigene60459\_TAEpaeTARAAPEI-5, Unigene60472\_TAEpaeTARAAPEI-5, Unigene60646\_TAEpaeTARAAPEI-5, Unigene60971\_TAEpaeTARAAPEI-5, Unigene61114\_TAEpaeTARAAPEI-5, Unigene61230\_TAEpaeTARAAPEI-5, Unigene61439\_TAEpaeTARAAPEI-5, Unigene61507\_TAEpaeTARAAPEI-5, Unigene61561\_TAEpaeTARAAPEI-5, Unigene61690\_TAEpaeTARAAPEI-5, Unigene61820\_TAEpaeTARAAPEI-5, Unigene61838\_TAEpaeTARAAPEI-5, Unigene62038\_TAEpaeTARAAPEI-5, Unigene62180\_TAEpaeTARAAPEI-5, Unigene62204\_TAEpaeTARAAPEI-5, Unigene62219\_TAEpaeTARAAPEI-5, Unigene6244\_TAEpaeTARAAPEI-5, Unigene62458\_TAEpaeTARAAPEI-5, Unigene6256\_TAEpaeTARAAPEI-5, Unigene62593\_TAEpaeTARAAPEI-5, Unigene62644\_TAEpaeTARAAPEI-5, Unigene62650\_TAEpaeTARAAPEI-5, Unigene62655\_TAEpaeTARAAPEI-5, Unigene62673\_TAEpaeTARAAPEI-5, Unigene62782\_TAEpaeTARAAPEI-5, Unigene62835\_TAEpaeTARAAPEI-5, Unigene62944\_TAEpaeTARAAPEI-5, Unigene63027\_TAEpaeTARAAPEI-5, Unigene63051\_TAEpaeTARAAPEI-5, Unigene63306\_TAEpaeTARAAPEI-5, Unigene6335\_TAEpaeTARAAPEI-5, Unigene63370\_TAEpaeTARAAPEI-5, Unigene63453\_TAEpaeTARAAPEI-5, Unigene63468\_TAEpaeTARAAPEI-5, Unigene63471\_TAEpaeTARAAPEI-5, Unigene63544\_TAEpaeTARAAPEI-5, Unigene63960\_TAEpaeTARAAPEI-5, Unigene64116\_TAEpaeTARAAPEI-5, Unigene64181\_TAEpaeTARAAPEI-5, Unigene64201\_TAEpaeTARAAPEI-5, Unigene64204\_TAEpaeTARAAPEI-5, Unigene64253\_TAEpaeTARAAPEI-5, Unigene64265\_TAEpaeTARAAPEI-5, Unigene64320\_TAEpaeTARAAPEI-5, Unigene64416\_TAEpaeTARAAPEI-5, Unigene64463\_TAEpaeTARAAPEI-5, Unigene6459\_TAEpaeTARAAPEI-5, Unigene64622\_TAEpaeTARAAPEI-5, Unigene64756\_TAEpaeTARAAPEI-5, Unigene64769\_TAEpaeTARAAPEI-5, Unigene64860\_TAEpaeTARAAPEI-5, Unigene64951\_TAEpaeTARAAPEI-5, Unigene64999\_TAEpaeTARAAPEI-5, Unigene65030\_TAEpaeTARAAPEI-5, Unigene65047\_TAEpaeTARAAPEI-5, Unigene65182\_TAEpaeTARAAPEI-5, Unigene65190\_TAEpaeTARAAPEI-5, Unigene65238\_TAEpaeTARAAPEI-5, Unigene65314\_TAEpaeTARAAPEI-5, Unigene65326\_TAEpaeTARAAPEI-5, Unigene65502\_TAEpaeTARAAPEI-5, Unigene65513\_TAEpaeTARAAPEI-5, Unigene65593\_TAEpaeTARAAPEI-5, Unigene65706\_TAEpaeTARAAPEI-5, Unigene65717\_TAEpaeTARAAPEI-5, Unigene65833\_TAEpaeTARAAPEI-5, Unigene65902\_TAEpaeTARAAPEI-5, Unigene65964\_TAEpaeTARAAPEI-5, Unigene66162\_TAEpaeTARAAPEI-5, Unigene66166\_TAEpaeTARAAPEI-5, Unigene66235\_TAEpaeTARAAPEI-5, Unigene66257\_TAEpaeTARAAPEI-5, Unigene66412\_TAEpaeTARAAPEI-5, Unigene66475\_TAEpaeTARAAPEI-5, Unigene66492\_TAEpaeTARAAPEI-5, Unigene66605\_TAEpaeTARAAPEI-5, Unigene66645\_TAEpaeTARAAPEI-5, Unigene66725\_TAEpaeTARAAPEI-5, Unigene66745\_TAEpaeTARAAPEI-5, Unigene66796\_TAEpaeTARAAPEI-5, Unigene66877\_TAEpaeTARAAPEI-5, Unigene66902\_TAEpaeTARAAPEI-5, Unigene66940\_TAEpaeTARAAPEI-5, Unigene66977\_TAEpaeTARAAPEI-5, Unigene67141\_TAEpaeTARAAPEI-5, Unigene67192\_TAEpaeTARAAPEI-5, Unigene67268\_TAEpaeTARAAPEI-5, Unigene67323\_TAEpaeTARAAPEI-5, Unigene67346\_TAEpaeTARAAPEI-5, Unigene67355\_TAEpaeTARAAPEI-5, Unigene67402\_TAEpaeTARAAPEI-5, Unigene67509\_TAEpaeTARAAPEI-5, Unigene67560\_TAEpaeTARAAPEI-5, Unigene67582\_TAEpaeTARAAPEI-5, Unigene67634\_TAEpaeTARAAPEI-5, Unigene67645\_TAEpaeTARAAPEI-5, Unigene67754\_TAEpaeTARAAPEI-5, Unigene67772\_TAEpaeTARAAPEI-5, Unigene67781\_TAEpaeTARAAPEI-5, Unigene67829\_TAEpaeTARAAPEI-5, Unigene67840\_TAEpaeTARAAPEI-5, Unigene67878\_TAEpaeTARAAPEI-5, Unigene67884\_TAEpaeTARAAPEI-5, Unigene67957\_TAEpaeTARAAPEI-5, Unigene68041\_TAEpaeTARAAPEI-5, Unigene68082\_TAEpaeTARAAPEI-5, Unigene68084\_TAEpaeTARAAPEI-5, Unigene68197\_TAEpaeTARAAPEI-5, Unigene68333\_TAEpaeTARAAPEI-5, Unigene68341\_TAEpaeTARAAPEI-5, Unigene68454\_TAEpaeTARAAPEI-5, Unigene68676\_TAEpaeTARAAPEI-5, Unigene68686\_TAEpaeTARAAPEI-5, Unigene68730\_TAEpaeTARAAPEI-5, Unigene68734\_TAEpaeTARAAPEI-5, Unigene69062\_TAEpaeTARAAPEI-5, Unigene69086\_TAEpaeTARAAPEI-5, Unigene69091\_TAEpaeTARAAPEI-5, Unigene69095\_TAEpaeTARAAPEI-5, Unigene69115\_TAEpaeTARAAPEI-5, Unigene69130\_TAEpaeTARAAPEI-5, Unigene69164\_TAEpaeTARAAPEI-5, Unigene69179\_TAEpaeTARAAPEI-5, Unigene69227\_TAEpaeTARAAPEI-5, Unigene69231\_TAEpaeTARAAPEI-5, Unigene69284\_TAEpaeTARAAPEI-5, Unigene69289\_TAEpaeTARAAPEI-5, Unigene69407\_TAEpaeTARAAPEI-5, Unigene69436\_TAEpaeTARAAPEI-5, Unigene69451\_TAEpaeTARAAPEI-5, Unigene69512\_TAEpaeTARAAPEI-5, Unigene69522\_TAEpaeTARAAPEI-5, Unigene69562\_TAEpaeTARAAPEI-5, Unigene69569\_TAEpaeTARAAPEI-5, Unigene69610\_TAEpaeTARAAPEI-5, Unigene69666\_TAEpaeTARAAPEI-5, Unigene69987\_TAEpaeTARAAPEI-5, Unigene70096\_TAEpaeTARAAPEI-5, Unigene70112\_TAEpaeTARAAPEI-5, Unigene70211\_TAEpaeTARAAPEI-5, Unigene70229\_TAEpaeTARAAPEI-5, Unigene70233\_TAEpaeTARAAPEI-5, Unigene70298\_TAEpaeTARAAPEI-5, Unigene70337\_TAEpaeTARAAPEI-5, Unigene70390\_TAEpaeTARAAPEI-5, Unigene70392\_TAEpaeTARAAPEI-5, Unigene70465\_TAEpaeTARAAPEI-5, Unigene70515\_TAEpaeTARAAPEI-5, Unigene70576\_TAEpaeTARAAPEI-5, Unigene70592\_TAEpaeTARAAPEI-5, Unigene70612\_TAEpaeTARAAPEI-5, Unigene70615\_TAEpaeTARAAPEI-5, Unigene70742\_TAEpaeTARAAPEI-5, Unigene70874\_TAEpaeTARAAPEI-5, Unigene70900\_TAEpaeTARAAPEI-5, Unigene71012\_TAEpaeTARAAPEI-5, Unigene71177\_TAEpaeTARAAPEI-5, Unigene71206\_TAEpaeTARAAPEI-5, Unigene71221\_TAEpaeTARAAPEI-5, Unigene712\_TAEpaeTARAAPEI-5, Unigene71366\_TAEpaeTARAAPEI-5, Unigene71438\_TAEpaeTARAAPEI-5, Unigene71460\_TAEpaeTARAAPEI-5, Unigene71535\_TAEpaeTARAAPEI-5, Unigene71551\_TAEpaeTARAAPEI-5, Unigene71592\_TAEpaeTARAAPEI-5, Unigene71606\_TAEpaeTARAAPEI-5, Unigene71635\_TAEpaeTARAAPEI-5, Unigene71636\_TAEpaeTARAAPEI-5, Unigene71658\_TAEpaeTARAAPEI-5, Unigene71675\_TAEpaeTARAAPEI-5, Unigene71776\_TAEpaeTARAAPEI-5, Unigene71820\_TAEpaeTARAAPEI-5, Unigene71839\_TAEpaeTARAAPEI-5, Unigene71906\_TAEpaeTARAAPEI-5, Unigene72002\_TAEpaeTARAAPEI-5, Unigene7201\_TAEpaeTARAAPEI-5, Unigene72057\_TAEpaeTARAAPEI-5, Unigene72084\_TAEpaeTARAAPEI-5, Unigene72087\_TAEpaeTARAAPEI-5, Unigene72108\_TAEpaeTARAAPEI-5, Unigene72155\_TAEpaeTARAAPEI-5, Unigene72177\_TAEpaeTARAAPEI-5, Unigene72350\_TAEpaeTARAAPEI-5, Unigene72392\_TAEpaeTARAAPEI-5, Unigene72429\_TAEpaeTARAAPEI-5, Unigene72481\_TAEpaeTARAAPEI-5, Unigene72579\_TAEpaeTARAAPEI-5, Unigene72595\_TAEpaeTARAAPEI-5, Unigene72624\_TAEpaeTARAAPEI-5, Unigene72630\_TAEpaeTARAAPEI-5, Unigene72643\_TAEpaeTARAAPEI-5, Unigene72658\_TAEpaeTARAAPEI-5, Unigene72673\_TAEpaeTARAAPEI-5, Unigene72720\_TAEpaeTARAAPEI-5, Unigene72739\_TAEpaeTARAAPEI-5, Unigene72746\_TAEpaeTARAAPEI-5, Unigene72782\_TAEpaeTARAAPEI-5, Unigene72809\_TAEpaeTARAAPEI-5, Unigene72825\_TAEpaeTARAAPEI-5, Unigene72828\_TAEpaeTARAAPEI-5, Unigene72844\_TAEpaeTARAAPEI-5, Unigene72896\_TAEpaeTARAAPEI-5, Unigene72944\_TAEpaeTARAAPEI-5, Unigene7294\_TAEpaeTARAAPEI-5, Unigene7375\_TAEpaeTARAAPEI-5, Unigene7421\_TAEpaeTARAAPEI-5, Unigene7482\_TAEpaeTARAAPEI-5, Unigene75\_TAEpaeTARAAPEI-5, Unigene7692\_TAEpaeTARAAPEI-5, Unigene7724\_TAEpaeTARAAPEI-5, Unigene7806\_TAEpaeTARAAPEI-5, Unigene8210\_TAEpaeTARAAPEI-5, Unigene8297\_TAEpaeTARAAPEI-5, Unigene8470\_TAEpaeTARAAPEI-5, Unigene8704\_TAEpaeTARAAPEI-5, Unigene8796\_TAEpaeTARAAPEI-5, Unigene8797\_TAEpaeTARAAPEI-5, Unigene8883\_TAEpaeTARAAPEI-5, Unigene9193\_TAEpaeTARAAPEI-5, Unigene98\_TAEpaeTARAAPEI-5 |
| 9 | Purine metabolism | Unigene1005\_TAEpaeTARAAPEI-5, Unigene10245\_TAEpaeTARAAPEI-5, Unigene10265\_TAEpaeTARAAPEI-5, Unigene10279\_TAEpaeTARAAPEI-5, Unigene10293\_TAEpaeTARAAPEI-5, Unigene10327\_TAEpaeTARAAPEI-5, Unigene10348\_TAEpaeTARAAPEI-5, Unigene10430\_TAEpaeTARAAPEI-5, Unigene10487\_TAEpaeTARAAPEI-5, Unigene10567\_TAEpaeTARAAPEI-5, Unigene10630\_TAEpaeTARAAPEI-5, Unigene10879\_TAEpaeTARAAPEI-5, Unigene11396\_TAEpaeTARAAPEI-5, Unigene11662\_TAEpaeTARAAPEI-5, Unigene11964\_TAEpaeTARAAPEI-5, Unigene1226\_TAEpaeTARAAPEI-5, Unigene12277\_TAEpaeTARAAPEI-5, Unigene12301\_TAEpaeTARAAPEI-5, Unigene12408\_TAEpaeTARAAPEI-5, Unigene12657\_TAEpaeTARAAPEI-5, Unigene12765\_TAEpaeTARAAPEI-5, Unigene13058\_TAEpaeTARAAPEI-5, Unigene13153\_TAEpaeTARAAPEI-5, Unigene13246\_TAEpaeTARAAPEI-5, Unigene13250\_TAEpaeTARAAPEI-5, Unigene13485\_TAEpaeTARAAPEI-5, Unigene13500\_TAEpaeTARAAPEI-5, Unigene13508\_TAEpaeTARAAPEI-5, Unigene13540\_TAEpaeTARAAPEI-5, Unigene13717\_TAEpaeTARAAPEI-5, Unigene14086\_TAEpaeTARAAPEI-5, Unigene14226\_TAEpaeTARAAPEI-5, Unigene14388\_TAEpaeTARAAPEI-5, Unigene14717\_TAEpaeTARAAPEI-5, Unigene14741\_TAEpaeTARAAPEI-5, Unigene14745\_TAEpaeTARAAPEI-5, Unigene14762\_TAEpaeTARAAPEI-5, Unigene15120\_TAEpaeTARAAPEI-5, Unigene15232\_TAEpaeTARAAPEI-5, Unigene15295\_TAEpaeTARAAPEI-5, Unigene15574\_TAEpaeTARAAPEI-5, Unigene15595\_TAEpaeTARAAPEI-5, Unigene1606\_TAEpaeTARAAPEI-5, Unigene16070\_TAEpaeTARAAPEI-5, Unigene16097\_TAEpaeTARAAPEI-5, Unigene16124\_TAEpaeTARAAPEI-5, Unigene16243\_TAEpaeTARAAPEI-5, Unigene16250\_TAEpaeTARAAPEI-5, Unigene16356\_TAEpaeTARAAPEI-5, Unigene16412\_TAEpaeTARAAPEI-5, Unigene16550\_TAEpaeTARAAPEI-5, Unigene16625\_TAEpaeTARAAPEI-5, Unigene16780\_TAEpaeTARAAPEI-5, Unigene16878\_TAEpaeTARAAPEI-5, Unigene16893\_TAEpaeTARAAPEI-5, Unigene17009\_TAEpaeTARAAPEI-5, Unigene17097\_TAEpaeTARAAPEI-5, Unigene17214\_TAEpaeTARAAPEI-5, Unigene17252\_TAEpaeTARAAPEI-5, Unigene17316\_TAEpaeTARAAPEI-5, Unigene17345\_TAEpaeTARAAPEI-5, Unigene17374\_TAEpaeTARAAPEI-5, Unigene17388\_TAEpaeTARAAPEI-5, Unigene17402\_TAEpaeTARAAPEI-5, Unigene17414\_TAEpaeTARAAPEI-5, Unigene17439\_TAEpaeTARAAPEI-5, Unigene17564\_TAEpaeTARAAPEI-5, Unigene17827\_TAEpaeTARAAPEI-5, Unigene17938\_TAEpaeTARAAPEI-5, Unigene18091\_TAEpaeTARAAPEI-5, Unigene1812\_TAEpaeTARAAPEI-5, Unigene18175\_TAEpaeTARAAPEI-5, Unigene18276\_TAEpaeTARAAPEI-5, Unigene18306\_TAEpaeTARAAPEI-5, Unigene18652\_TAEpaeTARAAPEI-5, Unigene19223\_TAEpaeTARAAPEI-5, Unigene19367\_TAEpaeTARAAPEI-5, Unigene19430\_TAEpaeTARAAPEI-5, Unigene19861\_TAEpaeTARAAPEI-5, Unigene20050\_TAEpaeTARAAPEI-5, Unigene20172\_TAEpaeTARAAPEI-5, Unigene20369\_TAEpaeTARAAPEI-5, Unigene20379\_TAEpaeTARAAPEI-5, Unigene20745\_TAEpaeTARAAPEI-5, Unigene20813\_TAEpaeTARAAPEI-5, Unigene20858\_TAEpaeTARAAPEI-5, Unigene21150\_TAEpaeTARAAPEI-5, Unigene21208\_TAEpaeTARAAPEI-5, Unigene21278\_TAEpaeTARAAPEI-5, Unigene21309\_TAEpaeTARAAPEI-5, Unigene22657\_TAEpaeTARAAPEI-5, Unigene23183\_TAEpaeTARAAPEI-5, Unigene2342\_TAEpaeTARAAPEI-5, Unigene23770\_TAEpaeTARAAPEI-5, Unigene23829\_TAEpaeTARAAPEI-5, Unigene24489\_TAEpaeTARAAPEI-5, Unigene2453\_TAEpaeTARAAPEI-5, Unigene24684\_TAEpaeTARAAPEI-5, Unigene2488\_TAEpaeTARAAPEI-5, Unigene25215\_TAEpaeTARAAPEI-5, Unigene25278\_TAEpaeTARAAPEI-5, Unigene2536\_TAEpaeTARAAPEI-5, Unigene25456\_TAEpaeTARAAPEI-5, Unigene25481\_TAEpaeTARAAPEI-5, Unigene25886\_TAEpaeTARAAPEI-5, Unigene25889\_TAEpaeTARAAPEI-5, Unigene26322\_TAEpaeTARAAPEI-5, Unigene2639\_TAEpaeTARAAPEI-5, Unigene2657\_TAEpaeTARAAPEI-5, Unigene26669\_TAEpaeTARAAPEI-5, Unigene27267\_TAEpaeTARAAPEI-5, Unigene27281\_TAEpaeTARAAPEI-5, Unigene27386\_TAEpaeTARAAPEI-5, Unigene27541\_TAEpaeTARAAPEI-5, Unigene27931\_TAEpaeTARAAPEI-5, Unigene2812\_TAEpaeTARAAPEI-5, Unigene28263\_TAEpaeTARAAPEI-5, Unigene28589\_TAEpaeTARAAPEI-5, Unigene28613\_TAEpaeTARAAPEI-5, Unigene29097\_TAEpaeTARAAPEI-5, Unigene29158\_TAEpaeTARAAPEI-5, Unigene29272\_TAEpaeTARAAPEI-5, Unigene29470\_TAEpaeTARAAPEI-5, Unigene29570\_TAEpaeTARAAPEI-5, Unigene30040\_TAEpaeTARAAPEI-5, Unigene30097\_TAEpaeTARAAPEI-5, Unigene30102\_TAEpaeTARAAPEI-5, Unigene3019\_TAEpaeTARAAPEI-5, Unigene30381\_TAEpaeTARAAPEI-5, Unigene3048\_TAEpaeTARAAPEI-5, Unigene30664\_TAEpaeTARAAPEI-5, Unigene30810\_TAEpaeTARAAPEI-5, Unigene30861\_TAEpaeTARAAPEI-5, Unigene3089\_TAEpaeTARAAPEI-5, Unigene31093\_TAEpaeTARAAPEI-5, Unigene31114\_TAEpaeTARAAPEI-5, Unigene3116\_TAEpaeTARAAPEI-5, Unigene31314\_TAEpaeTARAAPEI-5, Unigene31525\_TAEpaeTARAAPEI-5, Unigene31560\_TAEpaeTARAAPEI-5, Unigene32050\_TAEpaeTARAAPEI-5, Unigene32142\_TAEpaeTARAAPEI-5, Unigene32377\_TAEpaeTARAAPEI-5, Unigene32384\_TAEpaeTARAAPEI-5, Unigene3261\_TAEpaeTARAAPEI-5, Unigene32625\_TAEpaeTARAAPEI-5, Unigene32729\_TAEpaeTARAAPEI-5, Unigene33074\_TAEpaeTARAAPEI-5, Unigene33092\_TAEpaeTARAAPEI-5, Unigene3309\_TAEpaeTARAAPEI-5, Unigene33280\_TAEpaeTARAAPEI-5, Unigene33391\_TAEpaeTARAAPEI-5, Unigene33583\_TAEpaeTARAAPEI-5, Unigene33860\_TAEpaeTARAAPEI-5, Unigene33973\_TAEpaeTARAAPEI-5, Unigene34046\_TAEpaeTARAAPEI-5, Unigene34171\_TAEpaeTARAAPEI-5, Unigene34381\_TAEpaeTARAAPEI-5, Unigene34694\_TAEpaeTARAAPEI-5, Unigene35001\_TAEpaeTARAAPEI-5, Unigene35164\_TAEpaeTARAAPEI-5, Unigene35198\_TAEpaeTARAAPEI-5, Unigene35393\_TAEpaeTARAAPEI-5, Unigene35466\_TAEpaeTARAAPEI-5, Unigene35553\_TAEpaeTARAAPEI-5, Unigene35577\_TAEpaeTARAAPEI-5, Unigene35653\_TAEpaeTARAAPEI-5, Unigene35793\_TAEpaeTARAAPEI-5, Unigene35948\_TAEpaeTARAAPEI-5, Unigene36056\_TAEpaeTARAAPEI-5, Unigene36284\_TAEpaeTARAAPEI-5, Unigene36413\_TAEpaeTARAAPEI-5, Unigene36608\_TAEpaeTARAAPEI-5, Unigene3667\_TAEpaeTARAAPEI-5, Unigene37031\_TAEpaeTARAAPEI-5, Unigene37141\_TAEpaeTARAAPEI-5, Unigene3729\_TAEpaeTARAAPEI-5, Unigene37426\_TAEpaeTARAAPEI-5, Unigene37539\_TAEpaeTARAAPEI-5, Unigene37568\_TAEpaeTARAAPEI-5, Unigene37652\_TAEpaeTARAAPEI-5, Unigene37878\_TAEpaeTARAAPEI-5, Unigene37925\_TAEpaeTARAAPEI-5, Unigene38047\_TAEpaeTARAAPEI-5, Unigene38295\_TAEpaeTARAAPEI-5, Unigene38720\_TAEpaeTARAAPEI-5, Unigene38863\_TAEpaeTARAAPEI-5, Unigene39391\_TAEpaeTARAAPEI-5, Unigene39531\_TAEpaeTARAAPEI-5, Unigene39716\_TAEpaeTARAAPEI-5, Unigene39805\_TAEpaeTARAAPEI-5, Unigene39841\_TAEpaeTARAAPEI-5, Unigene39914\_TAEpaeTARAAPEI-5, Unigene39973\_TAEpaeTARAAPEI-5, Unigene3\_TAEpaeTARAAPEI-5, Unigene4030\_TAEpaeTARAAPEI-5, Unigene40780\_TAEpaeTARAAPEI-5, Unigene40941\_TAEpaeTARAAPEI-5, Unigene41375\_TAEpaeTARAAPEI-5, Unigene41399\_TAEpaeTARAAPEI-5, Unigene41473\_TAEpaeTARAAPEI-5, Unigene41552\_TAEpaeTARAAPEI-5, Unigene42238\_TAEpaeTARAAPEI-5, Unigene42243\_TAEpaeTARAAPEI-5, Unigene42391\_TAEpaeTARAAPEI-5, Unigene42456\_TAEpaeTARAAPEI-5, Unigene4249\_TAEpaeTARAAPEI-5, Unigene42776\_TAEpaeTARAAPEI-5, Unigene42868\_TAEpaeTARAAPEI-5, Unigene4331\_TAEpaeTARAAPEI-5, Unigene43601\_TAEpaeTARAAPEI-5, Unigene43690\_TAEpaeTARAAPEI-5, Unigene43812\_TAEpaeTARAAPEI-5, Unigene43880\_TAEpaeTARAAPEI-5, Unigene44255\_TAEpaeTARAAPEI-5, Unigene4472\_TAEpaeTARAAPEI-5, Unigene45348\_TAEpaeTARAAPEI-5, Unigene45349\_TAEpaeTARAAPEI-5, Unigene45351\_TAEpaeTARAAPEI-5, Unigene45439\_TAEpaeTARAAPEI-5, Unigene45444\_TAEpaeTARAAPEI-5, Unigene45489\_TAEpaeTARAAPEI-5, Unigene45494\_TAEpaeTARAAPEI-5, Unigene45538\_TAEpaeTARAAPEI-5, Unigene45599\_TAEpaeTARAAPEI-5, Unigene4595\_TAEpaeTARAAPEI-5, Unigene46115\_TAEpaeTARAAPEI-5, Unigene4612\_TAEpaeTARAAPEI-5, Unigene46483\_TAEpaeTARAAPEI-5, Unigene46581\_TAEpaeTARAAPEI-5, Unigene46682\_TAEpaeTARAAPEI-5, Unigene46730\_TAEpaeTARAAPEI-5, Unigene46813\_TAEpaeTARAAPEI-5, Unigene46826\_TAEpaeTARAAPEI-5, Unigene4687\_TAEpaeTARAAPEI-5, Unigene46898\_TAEpaeTARAAPEI-5, Unigene47001\_TAEpaeTARAAPEI-5, Unigene47109\_TAEpaeTARAAPEI-5, Unigene47262\_TAEpaeTARAAPEI-5, Unigene47372\_TAEpaeTARAAPEI-5, Unigene4758\_TAEpaeTARAAPEI-5, Unigene47608\_TAEpaeTARAAPEI-5, Unigene47683\_TAEpaeTARAAPEI-5, Unigene47700\_TAEpaeTARAAPEI-5, Unigene47757\_TAEpaeTARAAPEI-5, Unigene47847\_TAEpaeTARAAPEI-5, Unigene47982\_TAEpaeTARAAPEI-5, Unigene48287\_TAEpaeTARAAPEI-5, Unigene48344\_TAEpaeTARAAPEI-5, Unigene48347\_TAEpaeTARAAPEI-5, Unigene48447\_TAEpaeTARAAPEI-5, Unigene48539\_TAEpaeTARAAPEI-5, Unigene4891\_TAEpaeTARAAPEI-5, Unigene49038\_TAEpaeTARAAPEI-5, Unigene49439\_TAEpaeTARAAPEI-5, Unigene49479\_TAEpaeTARAAPEI-5, Unigene49540\_TAEpaeTARAAPEI-5, Unigene49597\_TAEpaeTARAAPEI-5, Unigene49613\_TAEpaeTARAAPEI-5, Unigene49799\_TAEpaeTARAAPEI-5, Unigene49933\_TAEpaeTARAAPEI-5, Unigene49952\_TAEpaeTARAAPEI-5, Unigene50069\_TAEpaeTARAAPEI-5, Unigene50548\_TAEpaeTARAAPEI-5, Unigene50604\_TAEpaeTARAAPEI-5, Unigene50809\_TAEpaeTARAAPEI-5, Unigene50979\_TAEpaeTARAAPEI-5, Unigene51055\_TAEpaeTARAAPEI-5, Unigene51150\_TAEpaeTARAAPEI-5, Unigene5121\_TAEpaeTARAAPEI-5, Unigene51262\_TAEpaeTARAAPEI-5, Unigene51523\_TAEpaeTARAAPEI-5, Unigene51530\_TAEpaeTARAAPEI-5, Unigene51708\_TAEpaeTARAAPEI-5, Unigene51822\_TAEpaeTARAAPEI-5, Unigene51843\_TAEpaeTARAAPEI-5, Unigene51890\_TAEpaeTARAAPEI-5, Unigene51901\_TAEpaeTARAAPEI-5, Unigene51920\_TAEpaeTARAAPEI-5, Unigene51955\_TAEpaeTARAAPEI-5, Unigene52168\_TAEpaeTARAAPEI-5, Unigene5240\_TAEpaeTARAAPEI-5, Unigene52602\_TAEpaeTARAAPEI-5, Unigene52797\_TAEpaeTARAAPEI-5, Unigene5284\_TAEpaeTARAAPEI-5, Unigene53081\_TAEpaeTARAAPEI-5, Unigene53085\_TAEpaeTARAAPEI-5, Unigene53355\_TAEpaeTARAAPEI-5, Unigene53584\_TAEpaeTARAAPEI-5, Unigene53644\_TAEpaeTARAAPEI-5, Unigene53758\_TAEpaeTARAAPEI-5, Unigene537\_TAEpaeTARAAPEI-5, Unigene5380\_TAEpaeTARAAPEI-5, Unigene53926\_TAEpaeTARAAPEI-5, Unigene5398\_TAEpaeTARAAPEI-5, Unigene54298\_TAEpaeTARAAPEI-5, Unigene54447\_TAEpaeTARAAPEI-5, Unigene54534\_TAEpaeTARAAPEI-5, Unigene54541\_TAEpaeTARAAPEI-5, Unigene54669\_TAEpaeTARAAPEI-5, Unigene55060\_TAEpaeTARAAPEI-5, Unigene55265\_TAEpaeTARAAPEI-5, Unigene55437\_TAEpaeTARAAPEI-5, Unigene55510\_TAEpaeTARAAPEI-5, Unigene55601\_TAEpaeTARAAPEI-5, Unigene55636\_TAEpaeTARAAPEI-5, Unigene55649\_TAEpaeTARAAPEI-5, Unigene55656\_TAEpaeTARAAPEI-5, Unigene55682\_TAEpaeTARAAPEI-5, Unigene55767\_TAEpaeTARAAPEI-5, Unigene55790\_TAEpaeTARAAPEI-5, Unigene56003\_TAEpaeTARAAPEI-5, Unigene56061\_TAEpaeTARAAPEI-5, Unigene56173\_TAEpaeTARAAPEI-5, Unigene56282\_TAEpaeTARAAPEI-5, Unigene56500\_TAEpaeTARAAPEI-5, Unigene56512\_TAEpaeTARAAPEI-5, Unigene56585\_TAEpaeTARAAPEI-5, Unigene56934\_TAEpaeTARAAPEI-5, Unigene57157\_TAEpaeTARAAPEI-5, Unigene57177\_TAEpaeTARAAPEI-5, Unigene57224\_TAEpaeTARAAPEI-5, Unigene57338\_TAEpaeTARAAPEI-5, Unigene57373\_TAEpaeTARAAPEI-5, Unigene57587\_TAEpaeTARAAPEI-5, Unigene57599\_TAEpaeTARAAPEI-5, Unigene57601\_TAEpaeTARAAPEI-5, Unigene57622\_TAEpaeTARAAPEI-5, Unigene57627\_TAEpaeTARAAPEI-5, Unigene57688\_TAEpaeTARAAPEI-5, Unigene58250\_TAEpaeTARAAPEI-5, Unigene58303\_TAEpaeTARAAPEI-5, Unigene5841\_TAEpaeTARAAPEI-5, Unigene58525\_TAEpaeTARAAPEI-5, Unigene58716\_TAEpaeTARAAPEI-5, Unigene58734\_TAEpaeTARAAPEI-5, Unigene58794\_TAEpaeTARAAPEI-5, Unigene59015\_TAEpaeTARAAPEI-5, Unigene59053\_TAEpaeTARAAPEI-5, Unigene59066\_TAEpaeTARAAPEI-5, Unigene59226\_TAEpaeTARAAPEI-5, Unigene59250\_TAEpaeTARAAPEI-5, Unigene59303\_TAEpaeTARAAPEI-5, Unigene59346\_TAEpaeTARAAPEI-5, Unigene59370\_TAEpaeTARAAPEI-5, Unigene59424\_TAEpaeTARAAPEI-5, Unigene59529\_TAEpaeTARAAPEI-5, Unigene59886\_TAEpaeTARAAPEI-5, Unigene59916\_TAEpaeTARAAPEI-5, Unigene60059\_TAEpaeTARAAPEI-5, Unigene60152\_TAEpaeTARAAPEI-5, Unigene60222\_TAEpaeTARAAPEI-5, Unigene60487\_TAEpaeTARAAPEI-5, Unigene60491\_TAEpaeTARAAPEI-5, Unigene60585\_TAEpaeTARAAPEI-5, Unigene60695\_TAEpaeTARAAPEI-5, Unigene60814\_TAEpaeTARAAPEI-5, Unigene6092\_TAEpaeTARAAPEI-5, Unigene61092\_TAEpaeTARAAPEI-5, Unigene61126\_TAEpaeTARAAPEI-5, Unigene61179\_TAEpaeTARAAPEI-5, Unigene61184\_TAEpaeTARAAPEI-5, Unigene61337\_TAEpaeTARAAPEI-5, Unigene61387\_TAEpaeTARAAPEI-5, Unigene61390\_TAEpaeTARAAPEI-5, Unigene61408\_TAEpaeTARAAPEI-5, Unigene61583\_TAEpaeTARAAPEI-5, Unigene61626\_TAEpaeTARAAPEI-5, Unigene61920\_TAEpaeTARAAPEI-5, Unigene62192\_TAEpaeTARAAPEI-5, Unigene62205\_TAEpaeTARAAPEI-5, Unigene62444\_TAEpaeTARAAPEI-5, Unigene62494\_TAEpaeTARAAPEI-5, Unigene62507\_TAEpaeTARAAPEI-5, Unigene62686\_TAEpaeTARAAPEI-5, Unigene62783\_TAEpaeTARAAPEI-5, Unigene62959\_TAEpaeTARAAPEI-5, Unigene62980\_TAEpaeTARAAPEI-5, Unigene63078\_TAEpaeTARAAPEI-5, Unigene63196\_TAEpaeTARAAPEI-5, Unigene63298\_TAEpaeTARAAPEI-5, Unigene63308\_TAEpaeTARAAPEI-5, Unigene63333\_TAEpaeTARAAPEI-5, Unigene63432\_TAEpaeTARAAPEI-5, Unigene63556\_TAEpaeTARAAPEI-5, Unigene63658\_TAEpaeTARAAPEI-5, Unigene63709\_TAEpaeTARAAPEI-5, Unigene63870\_TAEpaeTARAAPEI-5, Unigene63994\_TAEpaeTARAAPEI-5, Unigene64102\_TAEpaeTARAAPEI-5, Unigene6410\_TAEpaeTARAAPEI-5, Unigene64121\_TAEpaeTARAAPEI-5, Unigene64142\_TAEpaeTARAAPEI-5, Unigene64165\_TAEpaeTARAAPEI-5, Unigene6417\_TAEpaeTARAAPEI-5, Unigene64198\_TAEpaeTARAAPEI-5, Unigene6434\_TAEpaeTARAAPEI-5, Unigene64379\_TAEpaeTARAAPEI-5, Unigene64406\_TAEpaeTARAAPEI-5, Unigene64430\_TAEpaeTARAAPEI-5, Unigene64439\_TAEpaeTARAAPEI-5, Unigene6449\_TAEpaeTARAAPEI-5, Unigene64566\_TAEpaeTARAAPEI-5, Unigene64568\_TAEpaeTARAAPEI-5, Unigene64739\_TAEpaeTARAAPEI-5, Unigene65165\_TAEpaeTARAAPEI-5, Unigene65273\_TAEpaeTARAAPEI-5, Unigene65333\_TAEpaeTARAAPEI-5, Unigene65341\_TAEpaeTARAAPEI-5, Unigene65415\_TAEpaeTARAAPEI-5, Unigene65488\_TAEpaeTARAAPEI-5, Unigene65518\_TAEpaeTARAAPEI-5, Unigene65528\_TAEpaeTARAAPEI-5, Unigene65572\_TAEpaeTARAAPEI-5, Unigene65634\_TAEpaeTARAAPEI-5, Unigene65657\_TAEpaeTARAAPEI-5, Unigene65794\_TAEpaeTARAAPEI-5, Unigene66029\_TAEpaeTARAAPEI-5, Unigene66070\_TAEpaeTARAAPEI-5, Unigene66177\_TAEpaeTARAAPEI-5, Unigene6662\_TAEpaeTARAAPEI-5, Unigene66673\_TAEpaeTARAAPEI-5, Unigene66807\_TAEpaeTARAAPEI-5, Unigene66971\_TAEpaeTARAAPEI-5, Unigene67146\_TAEpaeTARAAPEI-5, Unigene67324\_TAEpaeTARAAPEI-5, Unigene67454\_TAEpaeTARAAPEI-5, Unigene67489\_TAEpaeTARAAPEI-5, Unigene67539\_TAEpaeTARAAPEI-5, Unigene67590\_TAEpaeTARAAPEI-5, Unigene67598\_TAEpaeTARAAPEI-5, Unigene67860\_TAEpaeTARAAPEI-5, Unigene67876\_TAEpaeTARAAPEI-5, Unigene67977\_TAEpaeTARAAPEI-5, Unigene68022\_TAEpaeTARAAPEI-5, Unigene68038\_TAEpaeTARAAPEI-5, Unigene68189\_TAEpaeTARAAPEI-5, Unigene68239\_TAEpaeTARAAPEI-5, Unigene68316\_TAEpaeTARAAPEI-5, Unigene68347\_TAEpaeTARAAPEI-5, Unigene68525\_TAEpaeTARAAPEI-5, Unigene68574\_TAEpaeTARAAPEI-5, Unigene68576\_TAEpaeTARAAPEI-5, Unigene68639\_TAEpaeTARAAPEI-5, Unigene68858\_TAEpaeTARAAPEI-5, Unigene68999\_TAEpaeTARAAPEI-5, Unigene69234\_TAEpaeTARAAPEI-5, Unigene69309\_TAEpaeTARAAPEI-5, Unigene69322\_TAEpaeTARAAPEI-5, Unigene69434\_TAEpaeTARAAPEI-5, Unigene69442\_TAEpaeTARAAPEI-5, Unigene69617\_TAEpaeTARAAPEI-5, Unigene69734\_TAEpaeTARAAPEI-5, Unigene69748\_TAEpaeTARAAPEI-5, Unigene69836\_TAEpaeTARAAPEI-5, Unigene6987\_TAEpaeTARAAPEI-5, Unigene69900\_TAEpaeTARAAPEI-5, Unigene69975\_TAEpaeTARAAPEI-5, Unigene70019\_TAEpaeTARAAPEI-5, Unigene70131\_TAEpaeTARAAPEI-5, Unigene70145\_TAEpaeTARAAPEI-5, Unigene70156\_TAEpaeTARAAPEI-5, Unigene70213\_TAEpaeTARAAPEI-5, Unigene70317\_TAEpaeTARAAPEI-5, Unigene70359\_TAEpaeTARAAPEI-5, Unigene70404\_TAEpaeTARAAPEI-5, Unigene70434\_TAEpaeTARAAPEI-5, Unigene70446\_TAEpaeTARAAPEI-5, Unigene70454\_TAEpaeTARAAPEI-5, Unigene70468\_TAEpaeTARAAPEI-5, Unigene70593\_TAEpaeTARAAPEI-5, Unigene70633\_TAEpaeTARAAPEI-5, Unigene70730\_TAEpaeTARAAPEI-5, Unigene70795\_TAEpaeTARAAPEI-5, Unigene70810\_TAEpaeTARAAPEI-5, Unigene70830\_TAEpaeTARAAPEI-5, Unigene70863\_TAEpaeTARAAPEI-5, Unigene70926\_TAEpaeTARAAPEI-5, Unigene70995\_TAEpaeTARAAPEI-5, Unigene71062\_TAEpaeTARAAPEI-5, Unigene71078\_TAEpaeTARAAPEI-5, Unigene71091\_TAEpaeTARAAPEI-5, Unigene71109\_TAEpaeTARAAPEI-5, Unigene71120\_TAEpaeTARAAPEI-5, Unigene7114\_TAEpaeTARAAPEI-5, Unigene71175\_TAEpaeTARAAPEI-5, Unigene71205\_TAEpaeTARAAPEI-5, Unigene71217\_TAEpaeTARAAPEI-5, Unigene71245\_TAEpaeTARAAPEI-5, Unigene71269\_TAEpaeTARAAPEI-5, Unigene71359\_TAEpaeTARAAPEI-5, Unigene71498\_TAEpaeTARAAPEI-5, Unigene71565\_TAEpaeTARAAPEI-5, Unigene71676\_TAEpaeTARAAPEI-5, Unigene71687\_TAEpaeTARAAPEI-5, Unigene71799\_TAEpaeTARAAPEI-5, Unigene71822\_TAEpaeTARAAPEI-5, Unigene71840\_TAEpaeTARAAPEI-5, Unigene71857\_TAEpaeTARAAPEI-5, Unigene71875\_TAEpaeTARAAPEI-5, Unigene71876\_TAEpaeTARAAPEI-5, Unigene71993\_TAEpaeTARAAPEI-5, Unigene71998\_TAEpaeTARAAPEI-5, Unigene7199\_TAEpaeTARAAPEI-5, Unigene72081\_TAEpaeTARAAPEI-5, Unigene72237\_TAEpaeTARAAPEI-5, Unigene72240\_TAEpaeTARAAPEI-5, Unigene72256\_TAEpaeTARAAPEI-5, Unigene72340\_TAEpaeTARAAPEI-5, Unigene72360\_TAEpaeTARAAPEI-5, Unigene72374\_TAEpaeTARAAPEI-5, Unigene72500\_TAEpaeTARAAPEI-5, Unigene72531\_TAEpaeTARAAPEI-5, Unigene72569\_TAEpaeTARAAPEI-5, Unigene72576\_TAEpaeTARAAPEI-5, Unigene72585\_TAEpaeTARAAPEI-5, Unigene72702\_TAEpaeTARAAPEI-5, Unigene72705\_TAEpaeTARAAPEI-5, Unigene72775\_TAEpaeTARAAPEI-5, Unigene72780\_TAEpaeTARAAPEI-5, Unigene72816\_TAEpaeTARAAPEI-5, Unigene72843\_TAEpaeTARAAPEI-5, Unigene72938\_TAEpaeTARAAPEI-5, Unigene7456\_TAEpaeTARAAPEI-5, Unigene7718\_TAEpaeTARAAPEI-5, Unigene7772\_TAEpaeTARAAPEI-5, Unigene7943\_TAEpaeTARAAPEI-5, Unigene8272\_TAEpaeTARAAPEI-5, Unigene8605\_TAEpaeTARAAPEI-5, Unigene8654\_TAEpaeTARAAPEI-5, Unigene8816\_TAEpaeTARAAPEI-5, Unigene8954\_TAEpaeTARAAPEI-5, Unigene903\_TAEpaeTARAAPEI-5, Unigene907\_TAEpaeTARAAPEI-5, Unigene9118\_TAEpaeTARAAPEI-5, Unigene9271\_TAEpaeTARAAPEI-5, Unigene9415\_TAEpaeTARAAPEI-5, Unigene9468\_TAEpaeTARAAPEI-5, Unigene9582\_TAEpaeTARAAPEI-5, Unigene9626\_TAEpaeTARAAPEI-5, Unigene9651\_TAEpaeTARAAPEI-5, Unigene9767\_TAEpaeTARAAPEI-5, Unigene9851\_TAEpaeTARAAPEI-5 |
| 10 | MAPK signaling pathway | Unigene10010\_TAEpaeTARAAPEI-5, Unigene10302\_TAEpaeTARAAPEI-5, Unigene10358\_TAEpaeTARAAPEI-5, Unigene10454\_TAEpaeTARAAPEI-5, Unigene10547\_TAEpaeTARAAPEI-5, Unigene10689\_TAEpaeTARAAPEI-5, Unigene10794\_TAEpaeTARAAPEI-5, Unigene10870\_TAEpaeTARAAPEI-5, Unigene10957\_TAEpaeTARAAPEI-5, Unigene11019\_TAEpaeTARAAPEI-5, Unigene11062\_TAEpaeTARAAPEI-5, Unigene11115\_TAEpaeTARAAPEI-5, Unigene11170\_TAEpaeTARAAPEI-5, Unigene11355\_TAEpaeTARAAPEI-5, Unigene11575\_TAEpaeTARAAPEI-5, Unigene11603\_TAEpaeTARAAPEI-5, Unigene11818\_TAEpaeTARAAPEI-5, Unigene11971\_TAEpaeTARAAPEI-5, Unigene12204\_TAEpaeTARAAPEI-5, Unigene12247\_TAEpaeTARAAPEI-5, Unigene12264\_TAEpaeTARAAPEI-5, Unigene12285\_TAEpaeTARAAPEI-5, Unigene12495\_TAEpaeTARAAPEI-5, Unigene12956\_TAEpaeTARAAPEI-5, Unigene13046\_TAEpaeTARAAPEI-5, Unigene13227\_TAEpaeTARAAPEI-5, Unigene13231\_TAEpaeTARAAPEI-5, Unigene13439\_TAEpaeTARAAPEI-5, Unigene13555\_TAEpaeTARAAPEI-5, Unigene13656\_TAEpaeTARAAPEI-5, Unigene13681\_TAEpaeTARAAPEI-5, Unigene13723\_TAEpaeTARAAPEI-5, Unigene13747\_TAEpaeTARAAPEI-5, Unigene14178\_TAEpaeTARAAPEI-5, Unigene14270\_TAEpaeTARAAPEI-5, Unigene14330\_TAEpaeTARAAPEI-5, Unigene14369\_TAEpaeTARAAPEI-5, Unigene14371\_TAEpaeTARAAPEI-5, Unigene14855\_TAEpaeTARAAPEI-5, Unigene14940\_TAEpaeTARAAPEI-5, Unigene15075\_TAEpaeTARAAPEI-5, Unigene15247\_TAEpaeTARAAPEI-5, Unigene15331\_TAEpaeTARAAPEI-5, Unigene15730\_TAEpaeTARAAPEI-5, Unigene15756\_TAEpaeTARAAPEI-5, Unigene15808\_TAEpaeTARAAPEI-5, Unigene15833\_TAEpaeTARAAPEI-5, Unigene15867\_TAEpaeTARAAPEI-5, Unigene15987\_TAEpaeTARAAPEI-5, Unigene16134\_TAEpaeTARAAPEI-5, Unigene16218\_TAEpaeTARAAPEI-5, Unigene16468\_TAEpaeTARAAPEI-5, Unigene16509\_TAEpaeTARAAPEI-5, Unigene16529\_TAEpaeTARAAPEI-5, Unigene16691\_TAEpaeTARAAPEI-5, Unigene16824\_TAEpaeTARAAPEI-5, Unigene16948\_TAEpaeTARAAPEI-5, Unigene17130\_TAEpaeTARAAPEI-5, Unigene17167\_TAEpaeTARAAPEI-5, Unigene17246\_TAEpaeTARAAPEI-5, Unigene17352\_TAEpaeTARAAPEI-5, Unigene17364\_TAEpaeTARAAPEI-5, Unigene17397\_TAEpaeTARAAPEI-5, Unigene17403\_TAEpaeTARAAPEI-5, Unigene17673\_TAEpaeTARAAPEI-5, Unigene17689\_TAEpaeTARAAPEI-5, Unigene17700\_TAEpaeTARAAPEI-5, Unigene18070\_TAEpaeTARAAPEI-5, Unigene18420\_TAEpaeTARAAPEI-5, Unigene1943\_TAEpaeTARAAPEI-5, Unigene19456\_TAEpaeTARAAPEI-5, Unigene19640\_TAEpaeTARAAPEI-5, Unigene19937\_TAEpaeTARAAPEI-5, Unigene20195\_TAEpaeTARAAPEI-5, Unigene20403\_TAEpaeTARAAPEI-5, Unigene20863\_TAEpaeTARAAPEI-5, Unigene20962\_TAEpaeTARAAPEI-5, Unigene21363\_TAEpaeTARAAPEI-5, Unigene21544\_TAEpaeTARAAPEI-5, Unigene2171\_TAEpaeTARAAPEI-5, Unigene2172\_TAEpaeTARAAPEI-5, Unigene21943\_TAEpaeTARAAPEI-5, Unigene2206\_TAEpaeTARAAPEI-5, Unigene22264\_TAEpaeTARAAPEI-5, Unigene22482\_TAEpaeTARAAPEI-5, Unigene22762\_TAEpaeTARAAPEI-5, Unigene22934\_TAEpaeTARAAPEI-5, Unigene23068\_TAEpaeTARAAPEI-5, Unigene23209\_TAEpaeTARAAPEI-5, Unigene23406\_TAEpaeTARAAPEI-5, Unigene23452\_TAEpaeTARAAPEI-5, Unigene23792\_TAEpaeTARAAPEI-5, Unigene23848\_TAEpaeTARAAPEI-5, Unigene2429\_TAEpaeTARAAPEI-5, Unigene24420\_TAEpaeTARAAPEI-5, Unigene24595\_TAEpaeTARAAPEI-5, Unigene24825\_TAEpaeTARAAPEI-5, Unigene24931\_TAEpaeTARAAPEI-5, Unigene25039\_TAEpaeTARAAPEI-5, Unigene2562\_TAEpaeTARAAPEI-5, Unigene25681\_TAEpaeTARAAPEI-5, Unigene25684\_TAEpaeTARAAPEI-5, Unigene25710\_TAEpaeTARAAPEI-5, Unigene25859\_TAEpaeTARAAPEI-5, Unigene25921\_TAEpaeTARAAPEI-5, Unigene26068\_TAEpaeTARAAPEI-5, Unigene26111\_TAEpaeTARAAPEI-5, Unigene26859\_TAEpaeTARAAPEI-5, Unigene26882\_TAEpaeTARAAPEI-5, Unigene2701\_TAEpaeTARAAPEI-5, Unigene2722\_TAEpaeTARAAPEI-5, Unigene27376\_TAEpaeTARAAPEI-5, Unigene2754\_TAEpaeTARAAPEI-5, Unigene27715\_TAEpaeTARAAPEI-5, Unigene27896\_TAEpaeTARAAPEI-5, Unigene28047\_TAEpaeTARAAPEI-5, Unigene28464\_TAEpaeTARAAPEI-5, Unigene2882\_TAEpaeTARAAPEI-5, Unigene29157\_TAEpaeTARAAPEI-5, Unigene29203\_TAEpaeTARAAPEI-5, Unigene29376\_TAEpaeTARAAPEI-5, Unigene29499\_TAEpaeTARAAPEI-5, Unigene29638\_TAEpaeTARAAPEI-5, Unigene30672\_TAEpaeTARAAPEI-5, Unigene30803\_TAEpaeTARAAPEI-5, Unigene31014\_TAEpaeTARAAPEI-5, Unigene3109\_TAEpaeTARAAPEI-5, Unigene31474\_TAEpaeTARAAPEI-5, Unigene31512\_TAEpaeTARAAPEI-5, Unigene31615\_TAEpaeTARAAPEI-5, Unigene32081\_TAEpaeTARAAPEI-5, Unigene32215\_TAEpaeTARAAPEI-5, Unigene32274\_TAEpaeTARAAPEI-5, Unigene32643\_TAEpaeTARAAPEI-5, Unigene32680\_TAEpaeTARAAPEI-5, Unigene32748\_TAEpaeTARAAPEI-5, Unigene32826\_TAEpaeTARAAPEI-5, Unigene33089\_TAEpaeTARAAPEI-5, Unigene33497\_TAEpaeTARAAPEI-5, Unigene33696\_TAEpaeTARAAPEI-5, Unigene33849\_TAEpaeTARAAPEI-5, Unigene33930\_TAEpaeTARAAPEI-5, Unigene33932\_TAEpaeTARAAPEI-5, Unigene34049\_TAEpaeTARAAPEI-5, Unigene34159\_TAEpaeTARAAPEI-5, Unigene34344\_TAEpaeTARAAPEI-5, Unigene34447\_TAEpaeTARAAPEI-5, Unigene34593\_TAEpaeTARAAPEI-5, Unigene3481\_TAEpaeTARAAPEI-5, Unigene35003\_TAEpaeTARAAPEI-5, Unigene35102\_TAEpaeTARAAPEI-5, Unigene35420\_TAEpaeTARAAPEI-5, Unigene35440\_TAEpaeTARAAPEI-5, Unigene35498\_TAEpaeTARAAPEI-5, Unigene35733\_TAEpaeTARAAPEI-5, Unigene35839\_TAEpaeTARAAPEI-5, Unigene36256\_TAEpaeTARAAPEI-5, Unigene36358\_TAEpaeTARAAPEI-5, Unigene36435\_TAEpaeTARAAPEI-5, Unigene36586\_TAEpaeTARAAPEI-5, Unigene36628\_TAEpaeTARAAPEI-5, Unigene36695\_TAEpaeTARAAPEI-5, Unigene37038\_TAEpaeTARAAPEI-5, Unigene37059\_TAEpaeTARAAPEI-5, Unigene37192\_TAEpaeTARAAPEI-5, Unigene37236\_TAEpaeTARAAPEI-5, Unigene37267\_TAEpaeTARAAPEI-5, Unigene3739\_TAEpaeTARAAPEI-5, Unigene37455\_TAEpaeTARAAPEI-5, Unigene37519\_TAEpaeTARAAPEI-5, Unigene3766\_TAEpaeTARAAPEI-5, Unigene37692\_TAEpaeTARAAPEI-5, Unigene38044\_TAEpaeTARAAPEI-5, Unigene38160\_TAEpaeTARAAPEI-5, Unigene38182\_TAEpaeTARAAPEI-5, Unigene38545\_TAEpaeTARAAPEI-5, Unigene38660\_TAEpaeTARAAPEI-5, Unigene38738\_TAEpaeTARAAPEI-5, Unigene38782\_TAEpaeTARAAPEI-5, Unigene39101\_TAEpaeTARAAPEI-5, Unigene39200\_TAEpaeTARAAPEI-5, Unigene392\_TAEpaeTARAAPEI-5, Unigene39344\_TAEpaeTARAAPEI-5, Unigene39378\_TAEpaeTARAAPEI-5, Unigene40262\_TAEpaeTARAAPEI-5, Unigene40423\_TAEpaeTARAAPEI-5, Unigene40962\_TAEpaeTARAAPEI-5, Unigene40999\_TAEpaeTARAAPEI-5, Unigene41451\_TAEpaeTARAAPEI-5, Unigene41475\_TAEpaeTARAAPEI-5, Unigene41541\_TAEpaeTARAAPEI-5, Unigene41673\_TAEpaeTARAAPEI-5, Unigene41794\_TAEpaeTARAAPEI-5, Unigene42195\_TAEpaeTARAAPEI-5, Unigene43328\_TAEpaeTARAAPEI-5, Unigene4371\_TAEpaeTARAAPEI-5, Unigene4398\_TAEpaeTARAAPEI-5, Unigene4405\_TAEpaeTARAAPEI-5, Unigene44633\_TAEpaeTARAAPEI-5, Unigene44643\_TAEpaeTARAAPEI-5, Unigene44648\_TAEpaeTARAAPEI-5, Unigene44755\_TAEpaeTARAAPEI-5, Unigene44921\_TAEpaeTARAAPEI-5, Unigene45534\_TAEpaeTARAAPEI-5, Unigene45562\_TAEpaeTARAAPEI-5, Unigene45602\_TAEpaeTARAAPEI-5, Unigene46158\_TAEpaeTARAAPEI-5, Unigene46169\_TAEpaeTARAAPEI-5, Unigene46343\_TAEpaeTARAAPEI-5, Unigene46349\_TAEpaeTARAAPEI-5, Unigene46636\_TAEpaeTARAAPEI-5, Unigene46946\_TAEpaeTARAAPEI-5, Unigene4696\_TAEpaeTARAAPEI-5, Unigene46995\_TAEpaeTARAAPEI-5, Unigene47023\_TAEpaeTARAAPEI-5, Unigene47105\_TAEpaeTARAAPEI-5, Unigene47136\_TAEpaeTARAAPEI-5, Unigene47512\_TAEpaeTARAAPEI-5, Unigene4763\_TAEpaeTARAAPEI-5, Unigene47710\_TAEpaeTARAAPEI-5, Unigene47803\_TAEpaeTARAAPEI-5, Unigene47887\_TAEpaeTARAAPEI-5, Unigene47945\_TAEpaeTARAAPEI-5, Unigene48247\_TAEpaeTARAAPEI-5, Unigene48394\_TAEpaeTARAAPEI-5, Unigene48699\_TAEpaeTARAAPEI-5, Unigene48725\_TAEpaeTARAAPEI-5, Unigene48732\_TAEpaeTARAAPEI-5, Unigene48778\_TAEpaeTARAAPEI-5, Unigene48907\_TAEpaeTARAAPEI-5, Unigene49023\_TAEpaeTARAAPEI-5, Unigene49085\_TAEpaeTARAAPEI-5, Unigene49412\_TAEpaeTARAAPEI-5, Unigene49544\_TAEpaeTARAAPEI-5, Unigene49657\_TAEpaeTARAAPEI-5, Unigene49761\_TAEpaeTARAAPEI-5, Unigene49772\_TAEpaeTARAAPEI-5, Unigene49990\_TAEpaeTARAAPEI-5, Unigene50336\_TAEpaeTARAAPEI-5, Unigene50566\_TAEpaeTARAAPEI-5, Unigene50571\_TAEpaeTARAAPEI-5, Unigene50589\_TAEpaeTARAAPEI-5, Unigene50609\_TAEpaeTARAAPEI-5, Unigene50663\_TAEpaeTARAAPEI-5, Unigene51008\_TAEpaeTARAAPEI-5, Unigene51253\_TAEpaeTARAAPEI-5, Unigene51357\_TAEpaeTARAAPEI-5, Unigene51588\_TAEpaeTARAAPEI-5, Unigene51681\_TAEpaeTARAAPEI-5, Unigene51722\_TAEpaeTARAAPEI-5, Unigene51740\_TAEpaeTARAAPEI-5, Unigene51777\_TAEpaeTARAAPEI-5, Unigene51926\_TAEpaeTARAAPEI-5, Unigene5194\_TAEpaeTARAAPEI-5, Unigene52269\_TAEpaeTARAAPEI-5, Unigene5248\_TAEpaeTARAAPEI-5, Unigene52619\_TAEpaeTARAAPEI-5, Unigene52686\_TAEpaeTARAAPEI-5, Unigene52813\_TAEpaeTARAAPEI-5, Unigene52835\_TAEpaeTARAAPEI-5, Unigene52847\_TAEpaeTARAAPEI-5, Unigene53074\_TAEpaeTARAAPEI-5, Unigene53100\_TAEpaeTARAAPEI-5, Unigene53106\_TAEpaeTARAAPEI-5, Unigene53203\_TAEpaeTARAAPEI-5, Unigene53241\_TAEpaeTARAAPEI-5, Unigene5324\_TAEpaeTARAAPEI-5, Unigene53319\_TAEpaeTARAAPEI-5, Unigene53501\_TAEpaeTARAAPEI-5, Unigene53530\_TAEpaeTARAAPEI-5, Unigene53611\_TAEpaeTARAAPEI-5, Unigene53911\_TAEpaeTARAAPEI-5, Unigene54002\_TAEpaeTARAAPEI-5, Unigene54005\_TAEpaeTARAAPEI-5, Unigene54139\_TAEpaeTARAAPEI-5, Unigene54211\_TAEpaeTARAAPEI-5, Unigene54363\_TAEpaeTARAAPEI-5, Unigene54495\_TAEpaeTARAAPEI-5, Unigene54896\_TAEpaeTARAAPEI-5, Unigene55063\_TAEpaeTARAAPEI-5, Unigene55216\_TAEpaeTARAAPEI-5, Unigene55238\_TAEpaeTARAAPEI-5, Unigene55243\_TAEpaeTARAAPEI-5, Unigene55261\_TAEpaeTARAAPEI-5, Unigene55594\_TAEpaeTARAAPEI-5, Unigene55704\_TAEpaeTARAAPEI-5, Unigene55934\_TAEpaeTARAAPEI-5, Unigene56106\_TAEpaeTARAAPEI-5, Unigene5655\_TAEpaeTARAAPEI-5, Unigene56843\_TAEpaeTARAAPEI-5, Unigene56921\_TAEpaeTARAAPEI-5, Unigene56929\_TAEpaeTARAAPEI-5, Unigene5694\_TAEpaeTARAAPEI-5, Unigene57095\_TAEpaeTARAAPEI-5, Unigene57151\_TAEpaeTARAAPEI-5, Unigene57371\_TAEpaeTARAAPEI-5, Unigene57392\_TAEpaeTARAAPEI-5, Unigene57397\_TAEpaeTARAAPEI-5, Unigene57414\_TAEpaeTARAAPEI-5, Unigene57611\_TAEpaeTARAAPEI-5, Unigene57742\_TAEpaeTARAAPEI-5, Unigene57857\_TAEpaeTARAAPEI-5, Unigene57904\_TAEpaeTARAAPEI-5, Unigene57969\_TAEpaeTARAAPEI-5, Unigene58229\_TAEpaeTARAAPEI-5, Unigene58311\_TAEpaeTARAAPEI-5, Unigene58505\_TAEpaeTARAAPEI-5, Unigene58818\_TAEpaeTARAAPEI-5, Unigene58848\_TAEpaeTARAAPEI-5, Unigene59005\_TAEpaeTARAAPEI-5, Unigene59041\_TAEpaeTARAAPEI-5, Unigene59185\_TAEpaeTARAAPEI-5, Unigene59204\_TAEpaeTARAAPEI-5, Unigene59211\_TAEpaeTARAAPEI-5, Unigene59471\_TAEpaeTARAAPEI-5, Unigene59593\_TAEpaeTARAAPEI-5, Unigene5970\_TAEpaeTARAAPEI-5, Unigene59850\_TAEpaeTARAAPEI-5, Unigene59866\_TAEpaeTARAAPEI-5, Unigene59947\_TAEpaeTARAAPEI-5, Unigene60103\_TAEpaeTARAAPEI-5, Unigene60126\_TAEpaeTARAAPEI-5, Unigene60169\_TAEpaeTARAAPEI-5, Unigene60200\_TAEpaeTARAAPEI-5, Unigene60208\_TAEpaeTARAAPEI-5, Unigene60471\_TAEpaeTARAAPEI-5, Unigene60486\_TAEpaeTARAAPEI-5, Unigene60561\_TAEpaeTARAAPEI-5, Unigene60761\_TAEpaeTARAAPEI-5, Unigene60877\_TAEpaeTARAAPEI-5, Unigene60982\_TAEpaeTARAAPEI-5, Unigene60983\_TAEpaeTARAAPEI-5, Unigene60992\_TAEpaeTARAAPEI-5, Unigene61115\_TAEpaeTARAAPEI-5, Unigene61454\_TAEpaeTARAAPEI-5, Unigene61489\_TAEpaeTARAAPEI-5, Unigene61657\_TAEpaeTARAAPEI-5, Unigene62078\_TAEpaeTARAAPEI-5, Unigene62180\_TAEpaeTARAAPEI-5, Unigene62204\_TAEpaeTARAAPEI-5, Unigene62275\_TAEpaeTARAAPEI-5, Unigene62404\_TAEpaeTARAAPEI-5, Unigene62485\_TAEpaeTARAAPEI-5, Unigene62687\_TAEpaeTARAAPEI-5, Unigene62729\_TAEpaeTARAAPEI-5, Unigene62924\_TAEpaeTARAAPEI-5, Unigene62962\_TAEpaeTARAAPEI-5, Unigene62975\_TAEpaeTARAAPEI-5, Unigene63069\_TAEpaeTARAAPEI-5, Unigene63227\_TAEpaeTARAAPEI-5, Unigene63340\_TAEpaeTARAAPEI-5, Unigene63365\_TAEpaeTARAAPEI-5, Unigene63453\_TAEpaeTARAAPEI-5, Unigene63474\_TAEpaeTARAAPEI-5, Unigene63833\_TAEpaeTARAAPEI-5, Unigene63980\_TAEpaeTARAAPEI-5, Unigene64074\_TAEpaeTARAAPEI-5, Unigene64201\_TAEpaeTARAAPEI-5, Unigene64204\_TAEpaeTARAAPEI-5, Unigene64293\_TAEpaeTARAAPEI-5, Unigene64299\_TAEpaeTARAAPEI-5, Unigene64658\_TAEpaeTARAAPEI-5, Unigene64673\_TAEpaeTARAAPEI-5, Unigene64856\_TAEpaeTARAAPEI-5, Unigene64905\_TAEpaeTARAAPEI-5, Unigene65153\_TAEpaeTARAAPEI-5, Unigene6517\_TAEpaeTARAAPEI-5, Unigene65293\_TAEpaeTARAAPEI-5, Unigene65296\_TAEpaeTARAAPEI-5, Unigene65326\_TAEpaeTARAAPEI-5, Unigene65502\_TAEpaeTARAAPEI-5, Unigene65717\_TAEpaeTARAAPEI-5, Unigene65789\_TAEpaeTARAAPEI-5, Unigene66066\_TAEpaeTARAAPEI-5, Unigene66262\_TAEpaeTARAAPEI-5, Unigene66323\_TAEpaeTARAAPEI-5, Unigene66330\_TAEpaeTARAAPEI-5, Unigene66492\_TAEpaeTARAAPEI-5, Unigene66690\_TAEpaeTARAAPEI-5, Unigene66725\_TAEpaeTARAAPEI-5, Unigene66904\_TAEpaeTARAAPEI-5, Unigene66922\_TAEpaeTARAAPEI-5, Unigene67074\_TAEpaeTARAAPEI-5, Unigene67524\_TAEpaeTARAAPEI-5, Unigene67579\_TAEpaeTARAAPEI-5, Unigene67634\_TAEpaeTARAAPEI-5, Unigene67722\_TAEpaeTARAAPEI-5, Unigene67781\_TAEpaeTARAAPEI-5, Unigene67953\_TAEpaeTARAAPEI-5, Unigene68025\_TAEpaeTARAAPEI-5, Unigene68026\_TAEpaeTARAAPEI-5, Unigene68106\_TAEpaeTARAAPEI-5, Unigene68142\_TAEpaeTARAAPEI-5, Unigene68333\_TAEpaeTARAAPEI-5, Unigene68406\_TAEpaeTARAAPEI-5, Unigene68616\_TAEpaeTARAAPEI-5, Unigene68644\_TAEpaeTARAAPEI-5, Unigene68851\_TAEpaeTARAAPEI-5, Unigene68869\_TAEpaeTARAAPEI-5, Unigene68878\_TAEpaeTARAAPEI-5, Unigene68886\_TAEpaeTARAAPEI-5, Unigene68945\_TAEpaeTARAAPEI-5, Unigene68964\_TAEpaeTARAAPEI-5, Unigene68994\_TAEpaeTARAAPEI-5, Unigene69095\_TAEpaeTARAAPEI-5, Unigene69130\_TAEpaeTARAAPEI-5, Unigene69154\_TAEpaeTARAAPEI-5, Unigene69210\_TAEpaeTARAAPEI-5, Unigene69400\_TAEpaeTARAAPEI-5, Unigene69407\_TAEpaeTARAAPEI-5, Unigene69560\_TAEpaeTARAAPEI-5, Unigene69607\_TAEpaeTARAAPEI-5, Unigene69688\_TAEpaeTARAAPEI-5, Unigene69771\_TAEpaeTARAAPEI-5, Unigene69859\_TAEpaeTARAAPEI-5, Unigene69870\_TAEpaeTARAAPEI-5, Unigene69891\_TAEpaeTARAAPEI-5, Unigene69930\_TAEpaeTARAAPEI-5, Unigene69952\_TAEpaeTARAAPEI-5, Unigene70104\_TAEpaeTARAAPEI-5, Unigene70113\_TAEpaeTARAAPEI-5, Unigene70233\_TAEpaeTARAAPEI-5, Unigene70461\_TAEpaeTARAAPEI-5, Unigene70839\_TAEpaeTARAAPEI-5, Unigene70848\_TAEpaeTARAAPEI-5, Unigene70874\_TAEpaeTARAAPEI-5, Unigene70890\_TAEpaeTARAAPEI-5, Unigene70900\_TAEpaeTARAAPEI-5, Unigene70947\_TAEpaeTARAAPEI-5, Unigene71012\_TAEpaeTARAAPEI-5, Unigene71075\_TAEpaeTARAAPEI-5, Unigene71107\_TAEpaeTARAAPEI-5, Unigene71224\_TAEpaeTARAAPEI-5, Unigene71242\_TAEpaeTARAAPEI-5, Unigene71366\_TAEpaeTARAAPEI-5, Unigene71368\_TAEpaeTARAAPEI-5, Unigene71417\_TAEpaeTARAAPEI-5, Unigene71446\_TAEpaeTARAAPEI-5, Unigene71472\_TAEpaeTARAAPEI-5, Unigene71684\_TAEpaeTARAAPEI-5, Unigene71775\_TAEpaeTARAAPEI-5, Unigene71816\_TAEpaeTARAAPEI-5, Unigene71852\_TAEpaeTARAAPEI-5, Unigene71912\_TAEpaeTARAAPEI-5, Unigene71995\_TAEpaeTARAAPEI-5, Unigene72032\_TAEpaeTARAAPEI-5, Unigene72068\_TAEpaeTARAAPEI-5, Unigene72105\_TAEpaeTARAAPEI-5, Unigene72109\_TAEpaeTARAAPEI-5, Unigene72178\_TAEpaeTARAAPEI-5, Unigene72258\_TAEpaeTARAAPEI-5, Unigene72315\_TAEpaeTARAAPEI-5, Unigene72340\_TAEpaeTARAAPEI-5, Unigene72350\_TAEpaeTARAAPEI-5, Unigene72387\_TAEpaeTARAAPEI-5, Unigene72430\_TAEpaeTARAAPEI-5, Unigene72494\_TAEpaeTARAAPEI-5, Unigene72540\_TAEpaeTARAAPEI-5, Unigene72566\_TAEpaeTARAAPEI-5, Unigene72624\_TAEpaeTARAAPEI-5, Unigene72673\_TAEpaeTARAAPEI-5, Unigene72782\_TAEpaeTARAAPEI-5, Unigene72802\_TAEpaeTARAAPEI-5, Unigene72812\_TAEpaeTARAAPEI-5, Unigene72825\_TAEpaeTARAAPEI-5, Unigene72844\_TAEpaeTARAAPEI-5, Unigene72947\_TAEpaeTARAAPEI-5, Unigene7375\_TAEpaeTARAAPEI-5, Unigene7463\_TAEpaeTARAAPEI-5, Unigene7482\_TAEpaeTARAAPEI-5, Unigene7652\_TAEpaeTARAAPEI-5, Unigene8297\_TAEpaeTARAAPEI-5, Unigene8411\_TAEpaeTARAAPEI-5, Unigene8505\_TAEpaeTARAAPEI-5, Unigene8770\_TAEpaeTARAAPEI-5, Unigene8833\_TAEpaeTARAAPEI-5, Unigene907\_TAEpaeTARAAPEI-5, Unigene9202\_TAEpaeTARAAPEI-5, Unigene9376\_TAEpaeTARAAPEI-5, Unigene9501\_TAEpaeTARAAPEI-5, Unigene9577\_TAEpaeTARAAPEI-5, Unigene9640\_TAEpaeTARAAPEI-5, Unigene9705\_TAEpaeTARAAPEI-5, Unigene9827\_TAEpaeTARAAPEI-5, Unigene9962\_TAEpaeTARAAPEI-5 |
| 11 | Adherens junction | Unigene10085\_TAEpaeTARAAPEI-5, Unigene100\_TAEpaeTARAAPEI-5, Unigene10177\_TAEpaeTARAAPEI-5, Unigene10250\_TAEpaeTARAAPEI-5, Unigene1035\_TAEpaeTARAAPEI-5, Unigene10716\_TAEpaeTARAAPEI-5, Unigene10729\_TAEpaeTARAAPEI-5, Unigene10896\_TAEpaeTARAAPEI-5, Unigene11062\_TAEpaeTARAAPEI-5, Unigene11257\_TAEpaeTARAAPEI-5, Unigene11315\_TAEpaeTARAAPEI-5, Unigene11482\_TAEpaeTARAAPEI-5, Unigene11507\_TAEpaeTARAAPEI-5, Unigene11534\_TAEpaeTARAAPEI-5, Unigene11743\_TAEpaeTARAAPEI-5, Unigene11964\_TAEpaeTARAAPEI-5, Unigene11990\_TAEpaeTARAAPEI-5, Unigene12059\_TAEpaeTARAAPEI-5, Unigene12311\_TAEpaeTARAAPEI-5, Unigene12647\_TAEpaeTARAAPEI-5, Unigene12664\_TAEpaeTARAAPEI-5, Unigene12935\_TAEpaeTARAAPEI-5, Unigene12947\_TAEpaeTARAAPEI-5, Unigene12956\_TAEpaeTARAAPEI-5, Unigene12994\_TAEpaeTARAAPEI-5, Unigene13055\_TAEpaeTARAAPEI-5, Unigene13082\_TAEpaeTARAAPEI-5, Unigene13091\_TAEpaeTARAAPEI-5, Unigene13110\_TAEpaeTARAAPEI-5, Unigene13439\_TAEpaeTARAAPEI-5, Unigene13473\_TAEpaeTARAAPEI-5, Unigene13629\_TAEpaeTARAAPEI-5, Unigene13747\_TAEpaeTARAAPEI-5, Unigene1375\_TAEpaeTARAAPEI-5, Unigene13859\_TAEpaeTARAAPEI-5, Unigene13968\_TAEpaeTARAAPEI-5, Unigene14027\_TAEpaeTARAAPEI-5, Unigene14178\_TAEpaeTARAAPEI-5, Unigene14664\_TAEpaeTARAAPEI-5, Unigene14676\_TAEpaeTARAAPEI-5, Unigene14719\_TAEpaeTARAAPEI-5, Unigene14945\_TAEpaeTARAAPEI-5, Unigene15247\_TAEpaeTARAAPEI-5, Unigene15248\_TAEpaeTARAAPEI-5, Unigene15450\_TAEpaeTARAAPEI-5, Unigene15547\_TAEpaeTARAAPEI-5, Unigene15620\_TAEpaeTARAAPEI-5, Unigene15774\_TAEpaeTARAAPEI-5, Unigene15789\_TAEpaeTARAAPEI-5, Unigene15835\_TAEpaeTARAAPEI-5, Unigene15921\_TAEpaeTARAAPEI-5, Unigene15987\_TAEpaeTARAAPEI-5, Unigene16002\_TAEpaeTARAAPEI-5, Unigene16023\_TAEpaeTARAAPEI-5, Unigene1603\_TAEpaeTARAAPEI-5, Unigene16040\_TAEpaeTARAAPEI-5, Unigene16244\_TAEpaeTARAAPEI-5, Unigene16500\_TAEpaeTARAAPEI-5, Unigene16561\_TAEpaeTARAAPEI-5, Unigene1658\_TAEpaeTARAAPEI-5, Unigene16753\_TAEpaeTARAAPEI-5, Unigene16919\_TAEpaeTARAAPEI-5, Unigene17034\_TAEpaeTARAAPEI-5, Unigene1712\_TAEpaeTARAAPEI-5, Unigene17163\_TAEpaeTARAAPEI-5, Unigene17167\_TAEpaeTARAAPEI-5, Unigene17271\_TAEpaeTARAAPEI-5, Unigene17364\_TAEpaeTARAAPEI-5, Unigene17614\_TAEpaeTARAAPEI-5, Unigene17633\_TAEpaeTARAAPEI-5, Unigene17657\_TAEpaeTARAAPEI-5, Unigene17819\_TAEpaeTARAAPEI-5, Unigene17840\_TAEpaeTARAAPEI-5, Unigene17912\_TAEpaeTARAAPEI-5, Unigene17970\_TAEpaeTARAAPEI-5, Unigene18053\_TAEpaeTARAAPEI-5, Unigene18106\_TAEpaeTARAAPEI-5, Unigene18156\_TAEpaeTARAAPEI-5, Unigene18309\_TAEpaeTARAAPEI-5, Unigene18420\_TAEpaeTARAAPEI-5, Unigene19079\_TAEpaeTARAAPEI-5, Unigene19465\_TAEpaeTARAAPEI-5, Unigene19746\_TAEpaeTARAAPEI-5, Unigene20000\_TAEpaeTARAAPEI-5, Unigene20573\_TAEpaeTARAAPEI-5, Unigene20955\_TAEpaeTARAAPEI-5, Unigene21363\_TAEpaeTARAAPEI-5, Unigene21513\_TAEpaeTARAAPEI-5, Unigene2206\_TAEpaeTARAAPEI-5, Unigene22165\_TAEpaeTARAAPEI-5, Unigene22264\_TAEpaeTARAAPEI-5, Unigene22358\_TAEpaeTARAAPEI-5, Unigene23040\_TAEpaeTARAAPEI-5, Unigene23237\_TAEpaeTARAAPEI-5, Unigene2340\_TAEpaeTARAAPEI-5, Unigene23485\_TAEpaeTARAAPEI-5, Unigene2360\_TAEpaeTARAAPEI-5, Unigene238\_TAEpaeTARAAPEI-5, Unigene2439\_TAEpaeTARAAPEI-5, Unigene24488\_TAEpaeTARAAPEI-5, Unigene24530\_TAEpaeTARAAPEI-5, Unigene24538\_TAEpaeTARAAPEI-5, Unigene24595\_TAEpaeTARAAPEI-5, Unigene25334\_TAEpaeTARAAPEI-5, Unigene253\_TAEpaeTARAAPEI-5, Unigene26309\_TAEpaeTARAAPEI-5, Unigene26359\_TAEpaeTARAAPEI-5, Unigene26365\_TAEpaeTARAAPEI-5, Unigene27154\_TAEpaeTARAAPEI-5, Unigene27972\_TAEpaeTARAAPEI-5, Unigene28237\_TAEpaeTARAAPEI-5, Unigene28349\_TAEpaeTARAAPEI-5, Unigene28785\_TAEpaeTARAAPEI-5, Unigene2889\_TAEpaeTARAAPEI-5, Unigene29539\_TAEpaeTARAAPEI-5, Unigene29746\_TAEpaeTARAAPEI-5, Unigene30199\_TAEpaeTARAAPEI-5, Unigene30624\_TAEpaeTARAAPEI-5, Unigene31877\_TAEpaeTARAAPEI-5, Unigene32104\_TAEpaeTARAAPEI-5, Unigene32545\_TAEpaeTARAAPEI-5, Unigene32643\_TAEpaeTARAAPEI-5, Unigene32999\_TAEpaeTARAAPEI-5, Unigene33030\_TAEpaeTARAAPEI-5, Unigene33057\_TAEpaeTARAAPEI-5, Unigene33174\_TAEpaeTARAAPEI-5, Unigene3346\_TAEpaeTARAAPEI-5, Unigene33473\_TAEpaeTARAAPEI-5, Unigene3355\_TAEpaeTARAAPEI-5, Unigene34144\_TAEpaeTARAAPEI-5, Unigene34394\_TAEpaeTARAAPEI-5, Unigene35487\_TAEpaeTARAAPEI-5, Unigene35498\_TAEpaeTARAAPEI-5, Unigene35733\_TAEpaeTARAAPEI-5, Unigene3582\_TAEpaeTARAAPEI-5, Unigene35973\_TAEpaeTARAAPEI-5, Unigene36256\_TAEpaeTARAAPEI-5, Unigene36424\_TAEpaeTARAAPEI-5, Unigene36648\_TAEpaeTARAAPEI-5, Unigene36848\_TAEpaeTARAAPEI-5, Unigene37038\_TAEpaeTARAAPEI-5, Unigene37286\_TAEpaeTARAAPEI-5, Unigene37689\_TAEpaeTARAAPEI-5, Unigene37834\_TAEpaeTARAAPEI-5, Unigene37980\_TAEpaeTARAAPEI-5, Unigene37986\_TAEpaeTARAAPEI-5, Unigene38258\_TAEpaeTARAAPEI-5, Unigene3888\_TAEpaeTARAAPEI-5, Unigene38891\_TAEpaeTARAAPEI-5, Unigene39051\_TAEpaeTARAAPEI-5, Unigene39227\_TAEpaeTARAAPEI-5, Unigene39328\_TAEpaeTARAAPEI-5, Unigene39926\_TAEpaeTARAAPEI-5, Unigene40169\_TAEpaeTARAAPEI-5, Unigene40763\_TAEpaeTARAAPEI-5, Unigene40775\_TAEpaeTARAAPEI-5, Unigene40826\_TAEpaeTARAAPEI-5, Unigene41393\_TAEpaeTARAAPEI-5, Unigene41449\_TAEpaeTARAAPEI-5, Unigene41530\_TAEpaeTARAAPEI-5, Unigene4154\_TAEpaeTARAAPEI-5, Unigene41799\_TAEpaeTARAAPEI-5, Unigene42245\_TAEpaeTARAAPEI-5, Unigene42790\_TAEpaeTARAAPEI-5, Unigene43076\_TAEpaeTARAAPEI-5, Unigene43326\_TAEpaeTARAAPEI-5, Unigene43328\_TAEpaeTARAAPEI-5, Unigene43451\_TAEpaeTARAAPEI-5, Unigene43710\_TAEpaeTARAAPEI-5, Unigene4380\_TAEpaeTARAAPEI-5, Unigene43824\_TAEpaeTARAAPEI-5, Unigene43862\_TAEpaeTARAAPEI-5, Unigene43895\_TAEpaeTARAAPEI-5, Unigene43931\_TAEpaeTARAAPEI-5, Unigene44202\_TAEpaeTARAAPEI-5, Unigene44209\_TAEpaeTARAAPEI-5, Unigene44401\_TAEpaeTARAAPEI-5, Unigene44598\_TAEpaeTARAAPEI-5, Unigene44633\_TAEpaeTARAAPEI-5, Unigene4486\_TAEpaeTARAAPEI-5, Unigene44963\_TAEpaeTARAAPEI-5, Unigene45049\_TAEpaeTARAAPEI-5, Unigene45192\_TAEpaeTARAAPEI-5, Unigene45239\_TAEpaeTARAAPEI-5, Unigene45877\_TAEpaeTARAAPEI-5, Unigene46657\_TAEpaeTARAAPEI-5, Unigene46978\_TAEpaeTARAAPEI-5, Unigene47107\_TAEpaeTARAAPEI-5, Unigene47136\_TAEpaeTARAAPEI-5, Unigene47218\_TAEpaeTARAAPEI-5, Unigene47449\_TAEpaeTARAAPEI-5, Unigene47595\_TAEpaeTARAAPEI-5, Unigene47945\_TAEpaeTARAAPEI-5, Unigene480\_TAEpaeTARAAPEI-5, Unigene48353\_TAEpaeTARAAPEI-5, Unigene48636\_TAEpaeTARAAPEI-5, Unigene48705\_TAEpaeTARAAPEI-5, Unigene48725\_TAEpaeTARAAPEI-5, Unigene49074\_TAEpaeTARAAPEI-5, Unigene49088\_TAEpaeTARAAPEI-5, Unigene49096\_TAEpaeTARAAPEI-5, Unigene49516\_TAEpaeTARAAPEI-5, Unigene49589\_TAEpaeTARAAPEI-5, Unigene49657\_TAEpaeTARAAPEI-5, Unigene49966\_TAEpaeTARAAPEI-5, Unigene49990\_TAEpaeTARAAPEI-5, Unigene50024\_TAEpaeTARAAPEI-5, Unigene50216\_TAEpaeTARAAPEI-5, Unigene5033\_TAEpaeTARAAPEI-5, Unigene50566\_TAEpaeTARAAPEI-5, Unigene51640\_TAEpaeTARAAPEI-5, Unigene51722\_TAEpaeTARAAPEI-5, Unigene51903\_TAEpaeTARAAPEI-5, Unigene5194\_TAEpaeTARAAPEI-5, Unigene51987\_TAEpaeTARAAPEI-5, Unigene5198\_TAEpaeTARAAPEI-5, Unigene52030\_TAEpaeTARAAPEI-5, Unigene52138\_TAEpaeTARAAPEI-5, Unigene52312\_TAEpaeTARAAPEI-5, Unigene52394\_TAEpaeTARAAPEI-5, Unigene5248\_TAEpaeTARAAPEI-5, Unigene52519\_TAEpaeTARAAPEI-5, Unigene52835\_TAEpaeTARAAPEI-5, Unigene52867\_TAEpaeTARAAPEI-5, Unigene53446\_TAEpaeTARAAPEI-5, Unigene53471\_TAEpaeTARAAPEI-5, Unigene53552\_TAEpaeTARAAPEI-5, Unigene53786\_TAEpaeTARAAPEI-5, Unigene54161\_TAEpaeTARAAPEI-5, Unigene54363\_TAEpaeTARAAPEI-5, Unigene5523\_TAEpaeTARAAPEI-5, Unigene55349\_TAEpaeTARAAPEI-5, Unigene55494\_TAEpaeTARAAPEI-5, Unigene55579\_TAEpaeTARAAPEI-5, Unigene55783\_TAEpaeTARAAPEI-5, Unigene55954\_TAEpaeTARAAPEI-5, Unigene56110\_TAEpaeTARAAPEI-5, Unigene56276\_TAEpaeTARAAPEI-5, Unigene56332\_TAEpaeTARAAPEI-5, Unigene56511\_TAEpaeTARAAPEI-5, Unigene56725\_TAEpaeTARAAPEI-5, Unigene5683\_TAEpaeTARAAPEI-5, Unigene56915\_TAEpaeTARAAPEI-5, Unigene5694\_TAEpaeTARAAPEI-5, Unigene57076\_TAEpaeTARAAPEI-5, Unigene57095\_TAEpaeTARAAPEI-5, Unigene57161\_TAEpaeTARAAPEI-5, Unigene57203\_TAEpaeTARAAPEI-5, Unigene57223\_TAEpaeTARAAPEI-5, Unigene57392\_TAEpaeTARAAPEI-5, Unigene57972\_TAEpaeTARAAPEI-5, Unigene57974\_TAEpaeTARAAPEI-5, Unigene58027\_TAEpaeTARAAPEI-5, Unigene58407\_TAEpaeTARAAPEI-5, Unigene58768\_TAEpaeTARAAPEI-5, Unigene59211\_TAEpaeTARAAPEI-5, Unigene59282\_TAEpaeTARAAPEI-5, Unigene59436\_TAEpaeTARAAPEI-5, Unigene59593\_TAEpaeTARAAPEI-5, Unigene59800\_TAEpaeTARAAPEI-5, Unigene59850\_TAEpaeTARAAPEI-5, Unigene60126\_TAEpaeTARAAPEI-5, Unigene60131\_TAEpaeTARAAPEI-5, Unigene60196\_TAEpaeTARAAPEI-5, Unigene60254\_TAEpaeTARAAPEI-5, Unigene60344\_TAEpaeTARAAPEI-5, Unigene60374\_TAEpaeTARAAPEI-5, Unigene60606\_TAEpaeTARAAPEI-5, Unigene60634\_TAEpaeTARAAPEI-5, Unigene60808\_TAEpaeTARAAPEI-5, Unigene60837\_TAEpaeTARAAPEI-5, Unigene60934\_TAEpaeTARAAPEI-5, Unigene61029\_TAEpaeTARAAPEI-5, Unigene61141\_TAEpaeTARAAPEI-5, Unigene6116\_TAEpaeTARAAPEI-5, Unigene61507\_TAEpaeTARAAPEI-5, Unigene61561\_TAEpaeTARAAPEI-5, Unigene62275\_TAEpaeTARAAPEI-5, Unigene6262\_TAEpaeTARAAPEI-5, Unigene62742\_TAEpaeTARAAPEI-5, Unigene62793\_TAEpaeTARAAPEI-5, Unigene62920\_TAEpaeTARAAPEI-5, Unigene62999\_TAEpaeTARAAPEI-5, Unigene63040\_TAEpaeTARAAPEI-5, Unigene63468\_TAEpaeTARAAPEI-5, Unigene63565\_TAEpaeTARAAPEI-5, Unigene63616\_TAEpaeTARAAPEI-5, Unigene63745\_TAEpaeTARAAPEI-5, Unigene63960\_TAEpaeTARAAPEI-5, Unigene63998\_TAEpaeTARAAPEI-5, Unigene64089\_TAEpaeTARAAPEI-5, Unigene64114\_TAEpaeTARAAPEI-5, Unigene64122\_TAEpaeTARAAPEI-5, Unigene64140\_TAEpaeTARAAPEI-5, Unigene64160\_TAEpaeTARAAPEI-5, Unigene64293\_TAEpaeTARAAPEI-5, Unigene64398\_TAEpaeTARAAPEI-5, Unigene64468\_TAEpaeTARAAPEI-5, Unigene64687\_TAEpaeTARAAPEI-5, Unigene64689\_TAEpaeTARAAPEI-5, Unigene65145\_TAEpaeTARAAPEI-5, Unigene65212\_TAEpaeTARAAPEI-5, Unigene65502\_TAEpaeTARAAPEI-5, Unigene65514\_TAEpaeTARAAPEI-5, Unigene65636\_TAEpaeTARAAPEI-5, Unigene65717\_TAEpaeTARAAPEI-5, Unigene65738\_TAEpaeTARAAPEI-5, Unigene65864\_TAEpaeTARAAPEI-5, Unigene65920\_TAEpaeTARAAPEI-5, Unigene66063\_TAEpaeTARAAPEI-5, Unigene66080\_TAEpaeTARAAPEI-5, Unigene66106\_TAEpaeTARAAPEI-5, Unigene66127\_TAEpaeTARAAPEI-5, Unigene66141\_TAEpaeTARAAPEI-5, Unigene66158\_TAEpaeTARAAPEI-5, Unigene66225\_TAEpaeTARAAPEI-5, Unigene66291\_TAEpaeTARAAPEI-5, Unigene66309\_TAEpaeTARAAPEI-5, Unigene66324\_TAEpaeTARAAPEI-5, Unigene663\_TAEpaeTARAAPEI-5, Unigene66459\_TAEpaeTARAAPEI-5, Unigene66492\_TAEpaeTARAAPEI-5, Unigene66666\_TAEpaeTARAAPEI-5, Unigene66728\_TAEpaeTARAAPEI-5, Unigene66904\_TAEpaeTARAAPEI-5, Unigene67006\_TAEpaeTARAAPEI-5, Unigene67066\_TAEpaeTARAAPEI-5, Unigene67310\_TAEpaeTARAAPEI-5, Unigene67341\_TAEpaeTARAAPEI-5, Unigene67509\_TAEpaeTARAAPEI-5, Unigene67524\_TAEpaeTARAAPEI-5, Unigene67634\_TAEpaeTARAAPEI-5, Unigene67645\_TAEpaeTARAAPEI-5, Unigene67748\_TAEpaeTARAAPEI-5, Unigene67772\_TAEpaeTARAAPEI-5, Unigene67781\_TAEpaeTARAAPEI-5, Unigene67825\_TAEpaeTARAAPEI-5, Unigene67829\_TAEpaeTARAAPEI-5, Unigene67889\_TAEpaeTARAAPEI-5, Unigene67982\_TAEpaeTARAAPEI-5, Unigene68001\_TAEpaeTARAAPEI-5, Unigene68071\_TAEpaeTARAAPEI-5, Unigene68103\_TAEpaeTARAAPEI-5, Unigene68121\_TAEpaeTARAAPEI-5, Unigene68124\_TAEpaeTARAAPEI-5, Unigene68142\_TAEpaeTARAAPEI-5, Unigene68189\_TAEpaeTARAAPEI-5, Unigene68274\_TAEpaeTARAAPEI-5, Unigene68333\_TAEpaeTARAAPEI-5, Unigene68509\_TAEpaeTARAAPEI-5, Unigene68686\_TAEpaeTARAAPEI-5, Unigene68739\_TAEpaeTARAAPEI-5, Unigene68796\_TAEpaeTARAAPEI-5, Unigene68878\_TAEpaeTARAAPEI-5, Unigene68903\_TAEpaeTARAAPEI-5, Unigene68964\_TAEpaeTARAAPEI-5, Unigene69009\_TAEpaeTARAAPEI-5, Unigene69033\_TAEpaeTARAAPEI-5, Unigene69044\_TAEpaeTARAAPEI-5, Unigene69130\_TAEpaeTARAAPEI-5, Unigene69164\_TAEpaeTARAAPEI-5, Unigene69182\_TAEpaeTARAAPEI-5, Unigene69189\_TAEpaeTARAAPEI-5, Unigene69370\_TAEpaeTARAAPEI-5, Unigene69407\_TAEpaeTARAAPEI-5, Unigene6940\_TAEpaeTARAAPEI-5, Unigene69426\_TAEpaeTARAAPEI-5, Unigene69439\_TAEpaeTARAAPEI-5, Unigene6949\_TAEpaeTARAAPEI-5, Unigene69512\_TAEpaeTARAAPEI-5, Unigene69636\_TAEpaeTARAAPEI-5, Unigene69654\_TAEpaeTARAAPEI-5, Unigene69666\_TAEpaeTARAAPEI-5, Unigene69688\_TAEpaeTARAAPEI-5, Unigene69724\_TAEpaeTARAAPEI-5, Unigene69731\_TAEpaeTARAAPEI-5, Unigene69760\_TAEpaeTARAAPEI-5, Unigene69773\_TAEpaeTARAAPEI-5, Unigene69775\_TAEpaeTARAAPEI-5, Unigene69879\_TAEpaeTARAAPEI-5, Unigene69987\_TAEpaeTARAAPEI-5, Unigene70061\_TAEpaeTARAAPEI-5, Unigene70074\_TAEpaeTARAAPEI-5, Unigene70316\_TAEpaeTARAAPEI-5, Unigene70433\_TAEpaeTARAAPEI-5, Unigene70495\_TAEpaeTARAAPEI-5, Unigene70533\_TAEpaeTARAAPEI-5, Unigene70546\_TAEpaeTARAAPEI-5, Unigene70579\_TAEpaeTARAAPEI-5, Unigene70659\_TAEpaeTARAAPEI-5, Unigene70663\_TAEpaeTARAAPEI-5, Unigene70677\_TAEpaeTARAAPEI-5, Unigene70751\_TAEpaeTARAAPEI-5, Unigene70795\_TAEpaeTARAAPEI-5, Unigene70825\_TAEpaeTARAAPEI-5, Unigene70893\_TAEpaeTARAAPEI-5, Unigene70900\_TAEpaeTARAAPEI-5, Unigene70910\_TAEpaeTARAAPEI-5, Unigene70941\_TAEpaeTARAAPEI-5, Unigene71024\_TAEpaeTARAAPEI-5, Unigene71086\_TAEpaeTARAAPEI-5, Unigene71142\_TAEpaeTARAAPEI-5, Unigene71238\_TAEpaeTARAAPEI-5, Unigene71271\_TAEpaeTARAAPEI-5, Unigene71294\_TAEpaeTARAAPEI-5, Unigene71460\_TAEpaeTARAAPEI-5, Unigene71617\_TAEpaeTARAAPEI-5, Unigene71621\_TAEpaeTARAAPEI-5, Unigene71635\_TAEpaeTARAAPEI-5, Unigene71641\_TAEpaeTARAAPEI-5, Unigene71699\_TAEpaeTARAAPEI-5, Unigene71822\_TAEpaeTARAAPEI-5, Unigene71869\_TAEpaeTARAAPEI-5, Unigene71906\_TAEpaeTARAAPEI-5, Unigene71934\_TAEpaeTARAAPEI-5, Unigene71985\_TAEpaeTARAAPEI-5, Unigene72034\_TAEpaeTARAAPEI-5, Unigene72185\_TAEpaeTARAAPEI-5, Unigene72189\_TAEpaeTARAAPEI-5, Unigene7227\_TAEpaeTARAAPEI-5, Unigene72287\_TAEpaeTARAAPEI-5, Unigene72320\_TAEpaeTARAAPEI-5, Unigene72322\_TAEpaeTARAAPEI-5, Unigene72323\_TAEpaeTARAAPEI-5, Unigene72337\_TAEpaeTARAAPEI-5, Unigene72340\_TAEpaeTARAAPEI-5, Unigene72348\_TAEpaeTARAAPEI-5, Unigene72350\_TAEpaeTARAAPEI-5, Unigene72379\_TAEpaeTARAAPEI-5, Unigene72517\_TAEpaeTARAAPEI-5, Unigene72576\_TAEpaeTARAAPEI-5, Unigene72660\_TAEpaeTARAAPEI-5, Unigene72675\_TAEpaeTARAAPEI-5, Unigene72716\_TAEpaeTARAAPEI-5, Unigene72746\_TAEpaeTARAAPEI-5, Unigene72747\_TAEpaeTARAAPEI-5, Unigene72786\_TAEpaeTARAAPEI-5, Unigene72825\_TAEpaeTARAAPEI-5, Unigene72926\_TAEpaeTARAAPEI-5, Unigene7482\_TAEpaeTARAAPEI-5, Unigene7571\_TAEpaeTARAAPEI-5, Unigene7574\_TAEpaeTARAAPEI-5, Unigene7581\_TAEpaeTARAAPEI-5, Unigene7994\_TAEpaeTARAAPEI-5, Unigene8042\_TAEpaeTARAAPEI-5, Unigene8100\_TAEpaeTARAAPEI-5, Unigene8371\_TAEpaeTARAAPEI-5, Unigene8400\_TAEpaeTARAAPEI-5, Unigene8436\_TAEpaeTARAAPEI-5, Unigene8583\_TAEpaeTARAAPEI-5, Unigene8873\_TAEpaeTARAAPEI-5, Unigene8876\_TAEpaeTARAAPEI-5, Unigene9002\_TAEpaeTARAAPEI-5, Unigene9304\_TAEpaeTARAAPEI-5, Unigene9723\_TAEpaeTARAAPEI-5, Unigene9731\_TAEpaeTARAAPEI-5 |
| 12 | Cell cycle | Unigene10024\_TAEpaeTARAAPEI-5, Unigene10085\_TAEpaeTARAAPEI-5, Unigene10474\_TAEpaeTARAAPEI-5, Unigene10537\_TAEpaeTARAAPEI-5, Unigene10688\_TAEpaeTARAAPEI-5, Unigene10707\_TAEpaeTARAAPEI-5, Unigene11062\_TAEpaeTARAAPEI-5, Unigene11132\_TAEpaeTARAAPEI-5, Unigene11143\_TAEpaeTARAAPEI-5, Unigene11172\_TAEpaeTARAAPEI-5, Unigene11205\_TAEpaeTARAAPEI-5, Unigene11257\_TAEpaeTARAAPEI-5, Unigene11397\_TAEpaeTARAAPEI-5, Unigene11507\_TAEpaeTARAAPEI-5, Unigene11608\_TAEpaeTARAAPEI-5, Unigene11727\_TAEpaeTARAAPEI-5, Unigene11735\_TAEpaeTARAAPEI-5, Unigene12057\_TAEpaeTARAAPEI-5, Unigene120\_TAEpaeTARAAPEI-5, Unigene12381\_TAEpaeTARAAPEI-5, Unigene12404\_TAEpaeTARAAPEI-5, Unigene12421\_TAEpaeTARAAPEI-5, Unigene12583\_TAEpaeTARAAPEI-5, Unigene12835\_TAEpaeTARAAPEI-5, Unigene12940\_TAEpaeTARAAPEI-5, Unigene12945\_TAEpaeTARAAPEI-5, Unigene12970\_TAEpaeTARAAPEI-5, Unigene1304\_TAEpaeTARAAPEI-5, Unigene13149\_TAEpaeTARAAPEI-5, Unigene13473\_TAEpaeTARAAPEI-5, Unigene13659\_TAEpaeTARAAPEI-5, Unigene13814\_TAEpaeTARAAPEI-5, Unigene13864\_TAEpaeTARAAPEI-5, Unigene14133\_TAEpaeTARAAPEI-5, Unigene14213\_TAEpaeTARAAPEI-5, Unigene14481\_TAEpaeTARAAPEI-5, Unigene14664\_TAEpaeTARAAPEI-5, Unigene14744\_TAEpaeTARAAPEI-5, Unigene14871\_TAEpaeTARAAPEI-5, Unigene14913\_TAEpaeTARAAPEI-5, Unigene14945\_TAEpaeTARAAPEI-5, Unigene15056\_TAEpaeTARAAPEI-5, Unigene15060\_TAEpaeTARAAPEI-5, Unigene15181\_TAEpaeTARAAPEI-5, Unigene151\_TAEpaeTARAAPEI-5, Unigene15277\_TAEpaeTARAAPEI-5, Unigene15450\_TAEpaeTARAAPEI-5, Unigene15526\_TAEpaeTARAAPEI-5, Unigene16103\_TAEpaeTARAAPEI-5, Unigene16196\_TAEpaeTARAAPEI-5, Unigene16365\_TAEpaeTARAAPEI-5, Unigene16454\_TAEpaeTARAAPEI-5, Unigene16639\_TAEpaeTARAAPEI-5, Unigene16671\_TAEpaeTARAAPEI-5, Unigene16797\_TAEpaeTARAAPEI-5, Unigene16803\_TAEpaeTARAAPEI-5, Unigene16986\_TAEpaeTARAAPEI-5, Unigene17157\_TAEpaeTARAAPEI-5, Unigene17183\_TAEpaeTARAAPEI-5, Unigene17338\_TAEpaeTARAAPEI-5, Unigene17358\_TAEpaeTARAAPEI-5, Unigene17582\_TAEpaeTARAAPEI-5, Unigene17601\_TAEpaeTARAAPEI-5, Unigene17746\_TAEpaeTARAAPEI-5, Unigene17826\_TAEpaeTARAAPEI-5, Unigene17967\_TAEpaeTARAAPEI-5, Unigene17970\_TAEpaeTARAAPEI-5, Unigene18028\_TAEpaeTARAAPEI-5, Unigene18056\_TAEpaeTARAAPEI-5, Unigene18073\_TAEpaeTARAAPEI-5, Unigene18085\_TAEpaeTARAAPEI-5, Unigene18097\_TAEpaeTARAAPEI-5, Unigene18099\_TAEpaeTARAAPEI-5, Unigene1840\_TAEpaeTARAAPEI-5, Unigene18535\_TAEpaeTARAAPEI-5, Unigene186\_TAEpaeTARAAPEI-5, Unigene19155\_TAEpaeTARAAPEI-5, Unigene19864\_TAEpaeTARAAPEI-5, Unigene19866\_TAEpaeTARAAPEI-5, Unigene20120\_TAEpaeTARAAPEI-5, Unigene20277\_TAEpaeTARAAPEI-5, Unigene20349\_TAEpaeTARAAPEI-5, Unigene20549\_TAEpaeTARAAPEI-5, Unigene20709\_TAEpaeTARAAPEI-5, Unigene2140\_TAEpaeTARAAPEI-5, Unigene21546\_TAEpaeTARAAPEI-5, Unigene2158\_TAEpaeTARAAPEI-5, Unigene21976\_TAEpaeTARAAPEI-5, Unigene22299\_TAEpaeTARAAPEI-5, Unigene22648\_TAEpaeTARAAPEI-5, Unigene22759\_TAEpaeTARAAPEI-5, Unigene22821\_TAEpaeTARAAPEI-5, Unigene23040\_TAEpaeTARAAPEI-5, Unigene23119\_TAEpaeTARAAPEI-5, Unigene23237\_TAEpaeTARAAPEI-5, Unigene24057\_TAEpaeTARAAPEI-5, Unigene24488\_TAEpaeTARAAPEI-5, Unigene2459\_TAEpaeTARAAPEI-5, Unigene24718\_TAEpaeTARAAPEI-5, Unigene25630\_TAEpaeTARAAPEI-5, Unigene25983\_TAEpaeTARAAPEI-5, Unigene26117\_TAEpaeTARAAPEI-5, Unigene2653\_TAEpaeTARAAPEI-5, Unigene26702\_TAEpaeTARAAPEI-5, Unigene26719\_TAEpaeTARAAPEI-5, Unigene26759\_TAEpaeTARAAPEI-5, Unigene26761\_TAEpaeTARAAPEI-5, Unigene27470\_TAEpaeTARAAPEI-5, Unigene28001\_TAEpaeTARAAPEI-5, Unigene28557\_TAEpaeTARAAPEI-5, Unigene2898\_TAEpaeTARAAPEI-5, Unigene2899\_TAEpaeTARAAPEI-5, Unigene29929\_TAEpaeTARAAPEI-5, Unigene29946\_TAEpaeTARAAPEI-5, Unigene30332\_TAEpaeTARAAPEI-5, Unigene30552\_TAEpaeTARAAPEI-5, Unigene30895\_TAEpaeTARAAPEI-5, Unigene31332\_TAEpaeTARAAPEI-5, Unigene3154\_TAEpaeTARAAPEI-5, Unigene31613\_TAEpaeTARAAPEI-5, Unigene31957\_TAEpaeTARAAPEI-5, Unigene32180\_TAEpaeTARAAPEI-5, Unigene32643\_TAEpaeTARAAPEI-5, Unigene32650\_TAEpaeTARAAPEI-5, Unigene33029\_TAEpaeTARAAPEI-5, Unigene33033\_TAEpaeTARAAPEI-5, Unigene33061\_TAEpaeTARAAPEI-5, Unigene33125\_TAEpaeTARAAPEI-5, Unigene33316\_TAEpaeTARAAPEI-5, Unigene33387\_TAEpaeTARAAPEI-5, Unigene33628\_TAEpaeTARAAPEI-5, Unigene34084\_TAEpaeTARAAPEI-5, Unigene34203\_TAEpaeTARAAPEI-5, Unigene34405\_TAEpaeTARAAPEI-5, Unigene35640\_TAEpaeTARAAPEI-5, Unigene36008\_TAEpaeTARAAPEI-5, Unigene361\_TAEpaeTARAAPEI-5, Unigene36475\_TAEpaeTARAAPEI-5, Unigene36546\_TAEpaeTARAAPEI-5, Unigene3678\_TAEpaeTARAAPEI-5, Unigene37020\_TAEpaeTARAAPEI-5, Unigene370\_TAEpaeTARAAPEI-5, Unigene37318\_TAEpaeTARAAPEI-5, Unigene3737\_TAEpaeTARAAPEI-5, Unigene37420\_TAEpaeTARAAPEI-5, Unigene37488\_TAEpaeTARAAPEI-5, Unigene37724\_TAEpaeTARAAPEI-5, Unigene38035\_TAEpaeTARAAPEI-5, Unigene38062\_TAEpaeTARAAPEI-5, Unigene38063\_TAEpaeTARAAPEI-5, Unigene38388\_TAEpaeTARAAPEI-5, Unigene38472\_TAEpaeTARAAPEI-5, Unigene38671\_TAEpaeTARAAPEI-5, Unigene38851\_TAEpaeTARAAPEI-5, Unigene38\_TAEpaeTARAAPEI-5, Unigene39051\_TAEpaeTARAAPEI-5, Unigene39087\_TAEpaeTARAAPEI-5, Unigene39289\_TAEpaeTARAAPEI-5, Unigene40003\_TAEpaeTARAAPEI-5, Unigene40564\_TAEpaeTARAAPEI-5, Unigene40639\_TAEpaeTARAAPEI-5, Unigene41014\_TAEpaeTARAAPEI-5, Unigene41183\_TAEpaeTARAAPEI-5, Unigene41306\_TAEpaeTARAAPEI-5, Unigene41472\_TAEpaeTARAAPEI-5, Unigene41713\_TAEpaeTARAAPEI-5, Unigene41799\_TAEpaeTARAAPEI-5, Unigene41810\_TAEpaeTARAAPEI-5, Unigene41864\_TAEpaeTARAAPEI-5, Unigene4227\_TAEpaeTARAAPEI-5, Unigene42380\_TAEpaeTARAAPEI-5, Unigene42427\_TAEpaeTARAAPEI-5, Unigene42706\_TAEpaeTARAAPEI-5, Unigene42765\_TAEpaeTARAAPEI-5, Unigene43016\_TAEpaeTARAAPEI-5, Unigene43043\_TAEpaeTARAAPEI-5, Unigene43195\_TAEpaeTARAAPEI-5, Unigene43276\_TAEpaeTARAAPEI-5, Unigene43588\_TAEpaeTARAAPEI-5, Unigene4398\_TAEpaeTARAAPEI-5, Unigene44202\_TAEpaeTARAAPEI-5, Unigene4425\_TAEpaeTARAAPEI-5, Unigene4451\_TAEpaeTARAAPEI-5, Unigene44965\_TAEpaeTARAAPEI-5, Unigene45433\_TAEpaeTARAAPEI-5, Unigene45468\_TAEpaeTARAAPEI-5, Unigene46038\_TAEpaeTARAAPEI-5, Unigene46117\_TAEpaeTARAAPEI-5, Unigene46244\_TAEpaeTARAAPEI-5, Unigene46657\_TAEpaeTARAAPEI-5, Unigene47136\_TAEpaeTARAAPEI-5, Unigene4716\_TAEpaeTARAAPEI-5, Unigene47218\_TAEpaeTARAAPEI-5, Unigene47666\_TAEpaeTARAAPEI-5, Unigene47930\_TAEpaeTARAAPEI-5, Unigene48002\_TAEpaeTARAAPEI-5, Unigene48174\_TAEpaeTARAAPEI-5, Unigene48575\_TAEpaeTARAAPEI-5, Unigene48954\_TAEpaeTARAAPEI-5, Unigene49034\_TAEpaeTARAAPEI-5, Unigene49197\_TAEpaeTARAAPEI-5, Unigene49321\_TAEpaeTARAAPEI-5, Unigene49387\_TAEpaeTARAAPEI-5, Unigene50138\_TAEpaeTARAAPEI-5, Unigene50237\_TAEpaeTARAAPEI-5, Unigene50324\_TAEpaeTARAAPEI-5, Unigene50407\_TAEpaeTARAAPEI-5, Unigene50682\_TAEpaeTARAAPEI-5, Unigene50727\_TAEpaeTARAAPEI-5, Unigene50737\_TAEpaeTARAAPEI-5, Unigene5107\_TAEpaeTARAAPEI-5, Unigene51117\_TAEpaeTARAAPEI-5, Unigene51247\_TAEpaeTARAAPEI-5, Unigene51377\_TAEpaeTARAAPEI-5, Unigene51423\_TAEpaeTARAAPEI-5, Unigene51454\_TAEpaeTARAAPEI-5, Unigene51547\_TAEpaeTARAAPEI-5, Unigene51579\_TAEpaeTARAAPEI-5, Unigene51653\_TAEpaeTARAAPEI-5, Unigene51982\_TAEpaeTARAAPEI-5, Unigene52041\_TAEpaeTARAAPEI-5, Unigene52308\_TAEpaeTARAAPEI-5, Unigene52448\_TAEpaeTARAAPEI-5, Unigene53380\_TAEpaeTARAAPEI-5, Unigene53844\_TAEpaeTARAAPEI-5, Unigene54294\_TAEpaeTARAAPEI-5, Unigene5430\_TAEpaeTARAAPEI-5, Unigene54346\_TAEpaeTARAAPEI-5, Unigene54488\_TAEpaeTARAAPEI-5, Unigene54548\_TAEpaeTARAAPEI-5, Unigene55098\_TAEpaeTARAAPEI-5, Unigene55226\_TAEpaeTARAAPEI-5, Unigene55349\_TAEpaeTARAAPEI-5, Unigene55494\_TAEpaeTARAAPEI-5, Unigene55562\_TAEpaeTARAAPEI-5, Unigene55951\_TAEpaeTARAAPEI-5, Unigene55954\_TAEpaeTARAAPEI-5, Unigene55\_TAEpaeTARAAPEI-5, Unigene5618\_TAEpaeTARAAPEI-5, Unigene56524\_TAEpaeTARAAPEI-5, Unigene56833\_TAEpaeTARAAPEI-5, Unigene56908\_TAEpaeTARAAPEI-5, Unigene56939\_TAEpaeTARAAPEI-5, Unigene56984\_TAEpaeTARAAPEI-5, Unigene57492\_TAEpaeTARAAPEI-5, Unigene57531\_TAEpaeTARAAPEI-5, Unigene57659\_TAEpaeTARAAPEI-5, Unigene57670\_TAEpaeTARAAPEI-5, Unigene57820\_TAEpaeTARAAPEI-5, Unigene57850\_TAEpaeTARAAPEI-5, Unigene58000\_TAEpaeTARAAPEI-5, Unigene58208\_TAEpaeTARAAPEI-5, Unigene58278\_TAEpaeTARAAPEI-5, Unigene58427\_TAEpaeTARAAPEI-5, Unigene58582\_TAEpaeTARAAPEI-5, Unigene58837\_TAEpaeTARAAPEI-5, Unigene58904\_TAEpaeTARAAPEI-5, Unigene59098\_TAEpaeTARAAPEI-5, Unigene59178\_TAEpaeTARAAPEI-5, Unigene59245\_TAEpaeTARAAPEI-5, Unigene59282\_TAEpaeTARAAPEI-5, Unigene60125\_TAEpaeTARAAPEI-5, Unigene60184\_TAEpaeTARAAPEI-5, Unigene60215\_TAEpaeTARAAPEI-5, Unigene60254\_TAEpaeTARAAPEI-5, Unigene60258\_TAEpaeTARAAPEI-5, Unigene60383\_TAEpaeTARAAPEI-5, Unigene60456\_TAEpaeTARAAPEI-5, Unigene60601\_TAEpaeTARAAPEI-5, Unigene60680\_TAEpaeTARAAPEI-5, Unigene60868\_TAEpaeTARAAPEI-5, Unigene60907\_TAEpaeTARAAPEI-5, Unigene61006\_TAEpaeTARAAPEI-5, Unigene61064\_TAEpaeTARAAPEI-5, Unigene6107\_TAEpaeTARAAPEI-5, Unigene61141\_TAEpaeTARAAPEI-5, Unigene6116\_TAEpaeTARAAPEI-5, Unigene61175\_TAEpaeTARAAPEI-5, Unigene61459\_TAEpaeTARAAPEI-5, Unigene61481\_TAEpaeTARAAPEI-5, Unigene61603\_TAEpaeTARAAPEI-5, Unigene61696\_TAEpaeTARAAPEI-5, Unigene6170\_TAEpaeTARAAPEI-5, Unigene61873\_TAEpaeTARAAPEI-5, Unigene61989\_TAEpaeTARAAPEI-5, Unigene62049\_TAEpaeTARAAPEI-5, Unigene62160\_TAEpaeTARAAPEI-5, Unigene62249\_TAEpaeTARAAPEI-5, Unigene62338\_TAEpaeTARAAPEI-5, Unigene62366\_TAEpaeTARAAPEI-5, Unigene62413\_TAEpaeTARAAPEI-5, Unigene62539\_TAEpaeTARAAPEI-5, Unigene6260\_TAEpaeTARAAPEI-5, Unigene62793\_TAEpaeTARAAPEI-5, Unigene62874\_TAEpaeTARAAPEI-5, Unigene62979\_TAEpaeTARAAPEI-5, Unigene62995\_TAEpaeTARAAPEI-5, Unigene63097\_TAEpaeTARAAPEI-5, Unigene63110\_TAEpaeTARAAPEI-5, Unigene63171\_TAEpaeTARAAPEI-5, Unigene63384\_TAEpaeTARAAPEI-5, Unigene63495\_TAEpaeTARAAPEI-5, Unigene6353\_TAEpaeTARAAPEI-5, Unigene63682\_TAEpaeTARAAPEI-5, Unigene63745\_TAEpaeTARAAPEI-5, Unigene63794\_TAEpaeTARAAPEI-5, Unigene63848\_TAEpaeTARAAPEI-5, Unigene63900\_TAEpaeTARAAPEI-5, Unigene63998\_TAEpaeTARAAPEI-5, Unigene64152\_TAEpaeTARAAPEI-5, Unigene64186\_TAEpaeTARAAPEI-5, Unigene64372\_TAEpaeTARAAPEI-5, Unigene64385\_TAEpaeTARAAPEI-5, Unigene64437\_TAEpaeTARAAPEI-5, Unigene64602\_TAEpaeTARAAPEI-5, Unigene64655\_TAEpaeTARAAPEI-5, Unigene64671\_TAEpaeTARAAPEI-5, Unigene64724\_TAEpaeTARAAPEI-5, Unigene65507\_TAEpaeTARAAPEI-5, Unigene65617\_TAEpaeTARAAPEI-5, Unigene65658\_TAEpaeTARAAPEI-5, Unigene65735\_TAEpaeTARAAPEI-5, Unigene65753\_TAEpaeTARAAPEI-5, Unigene65811\_TAEpaeTARAAPEI-5, Unigene65916\_TAEpaeTARAAPEI-5, Unigene65920\_TAEpaeTARAAPEI-5, Unigene65977\_TAEpaeTARAAPEI-5, Unigene66047\_TAEpaeTARAAPEI-5, Unigene66066\_TAEpaeTARAAPEI-5, Unigene66183\_TAEpaeTARAAPEI-5, Unigene66195\_TAEpaeTARAAPEI-5, Unigene66307\_TAEpaeTARAAPEI-5, Unigene66825\_TAEpaeTARAAPEI-5, Unigene66850\_TAEpaeTARAAPEI-5, Unigene67033\_TAEpaeTARAAPEI-5, Unigene67176\_TAEpaeTARAAPEI-5, Unigene67183\_TAEpaeTARAAPEI-5, Unigene67245\_TAEpaeTARAAPEI-5, Unigene67305\_TAEpaeTARAAPEI-5, Unigene67307\_TAEpaeTARAAPEI-5, Unigene6733\_TAEpaeTARAAPEI-5, Unigene67474\_TAEpaeTARAAPEI-5, Unigene67546\_TAEpaeTARAAPEI-5, Unigene67591\_TAEpaeTARAAPEI-5, Unigene67675\_TAEpaeTARAAPEI-5, Unigene67734\_TAEpaeTARAAPEI-5, Unigene67768\_TAEpaeTARAAPEI-5, Unigene67858\_TAEpaeTARAAPEI-5, Unigene67968\_TAEpaeTARAAPEI-5, Unigene68059\_TAEpaeTARAAPEI-5, Unigene68088\_TAEpaeTARAAPEI-5, Unigene68104\_TAEpaeTARAAPEI-5, Unigene68510\_TAEpaeTARAAPEI-5, Unigene68528\_TAEpaeTARAAPEI-5, Unigene68554\_TAEpaeTARAAPEI-5, Unigene68658\_TAEpaeTARAAPEI-5, Unigene68726\_TAEpaeTARAAPEI-5, Unigene68739\_TAEpaeTARAAPEI-5, Unigene68767\_TAEpaeTARAAPEI-5, Unigene68903\_TAEpaeTARAAPEI-5, Unigene68\_TAEpaeTARAAPEI-5, Unigene69008\_TAEpaeTARAAPEI-5, Unigene69044\_TAEpaeTARAAPEI-5, Unigene69174\_TAEpaeTARAAPEI-5, Unigene69217\_TAEpaeTARAAPEI-5, Unigene69310\_TAEpaeTARAAPEI-5, Unigene69439\_TAEpaeTARAAPEI-5, Unigene69515\_TAEpaeTARAAPEI-5, Unigene6966\_TAEpaeTARAAPEI-5, Unigene69821\_TAEpaeTARAAPEI-5, Unigene70089\_TAEpaeTARAAPEI-5, Unigene70162\_TAEpaeTARAAPEI-5, Unigene70177\_TAEpaeTARAAPEI-5, Unigene70203\_TAEpaeTARAAPEI-5, Unigene70224\_TAEpaeTARAAPEI-5, Unigene70384\_TAEpaeTARAAPEI-5, Unigene70454\_TAEpaeTARAAPEI-5, Unigene70495\_TAEpaeTARAAPEI-5, Unigene70546\_TAEpaeTARAAPEI-5, Unigene70567\_TAEpaeTARAAPEI-5, Unigene70571\_TAEpaeTARAAPEI-5, Unigene70669\_TAEpaeTARAAPEI-5, Unigene70681\_TAEpaeTARAAPEI-5, Unigene70756\_TAEpaeTARAAPEI-5, Unigene70855\_TAEpaeTARAAPEI-5, Unigene70906\_TAEpaeTARAAPEI-5, Unigene7120\_TAEpaeTARAAPEI-5, Unigene71238\_TAEpaeTARAAPEI-5, Unigene71301\_TAEpaeTARAAPEI-5, Unigene71312\_TAEpaeTARAAPEI-5, Unigene71433\_TAEpaeTARAAPEI-5, Unigene71440\_TAEpaeTARAAPEI-5, Unigene71601\_TAEpaeTARAAPEI-5, Unigene71641\_TAEpaeTARAAPEI-5, Unigene71670\_TAEpaeTARAAPEI-5, Unigene71677\_TAEpaeTARAAPEI-5, Unigene71723\_TAEpaeTARAAPEI-5, Unigene71749\_TAEpaeTARAAPEI-5, Unigene71762\_TAEpaeTARAAPEI-5, Unigene71822\_TAEpaeTARAAPEI-5, Unigene71841\_TAEpaeTARAAPEI-5, Unigene71842\_TAEpaeTARAAPEI-5, Unigene72022\_TAEpaeTARAAPEI-5, Unigene72023\_TAEpaeTARAAPEI-5, Unigene72055\_TAEpaeTARAAPEI-5, Unigene72058\_TAEpaeTARAAPEI-5, Unigene72067\_TAEpaeTARAAPEI-5, Unigene72298\_TAEpaeTARAAPEI-5, Unigene72323\_TAEpaeTARAAPEI-5, Unigene72348\_TAEpaeTARAAPEI-5, Unigene72358\_TAEpaeTARAAPEI-5, Unigene72392\_TAEpaeTARAAPEI-5, Unigene72403\_TAEpaeTARAAPEI-5, Unigene72455\_TAEpaeTARAAPEI-5, Unigene7246\_TAEpaeTARAAPEI-5, Unigene72655\_TAEpaeTARAAPEI-5, Unigene72661\_TAEpaeTARAAPEI-5, Unigene72675\_TAEpaeTARAAPEI-5, Unigene72691\_TAEpaeTARAAPEI-5, Unigene72786\_TAEpaeTARAAPEI-5, Unigene72863\_TAEpaeTARAAPEI-5, Unigene72882\_TAEpaeTARAAPEI-5, Unigene72887\_TAEpaeTARAAPEI-5, Unigene72926\_TAEpaeTARAAPEI-5, Unigene7445\_TAEpaeTARAAPEI-5, Unigene7574\_TAEpaeTARAAPEI-5, Unigene8086\_TAEpaeTARAAPEI-5, Unigene8235\_TAEpaeTARAAPEI-5, Unigene8364\_TAEpaeTARAAPEI-5, Unigene8441\_TAEpaeTARAAPEI-5, Unigene8466\_TAEpaeTARAAPEI-5, Unigene8488\_TAEpaeTARAAPEI-5, Unigene8628\_TAEpaeTARAAPEI-5, Unigene8755\_TAEpaeTARAAPEI-5, Unigene8873\_TAEpaeTARAAPEI-5, Unigene9234\_TAEpaeTARAAPEI-5, Unigene9412\_TAEpaeTARAAPEI-5, Unigene9443\_TAEpaeTARAAPEI-5, Unigene9785\_TAEpaeTARAAPEI-5, Unigene9810\_TAEpaeTARAAPEI-5, Unigene98\_TAEpaeTARAAPEI-5 |
| 13 | Insulin signaling pathway | Unigene10441\_TAEpaeTARAAPEI-5, Unigene10716\_TAEpaeTARAAPEI-5, Unigene10848\_TAEpaeTARAAPEI-5, Unigene10896\_TAEpaeTARAAPEI-5, Unigene11123\_TAEpaeTARAAPEI-5, Unigene11389\_TAEpaeTARAAPEI-5, Unigene11397\_TAEpaeTARAAPEI-5, Unigene11990\_TAEpaeTARAAPEI-5, Unigene12285\_TAEpaeTARAAPEI-5, Unigene12758\_TAEpaeTARAAPEI-5, Unigene12956\_TAEpaeTARAAPEI-5, Unigene13066\_TAEpaeTARAAPEI-5, Unigene13361\_TAEpaeTARAAPEI-5, Unigene13747\_TAEpaeTARAAPEI-5, Unigene13763\_TAEpaeTARAAPEI-5, Unigene13890\_TAEpaeTARAAPEI-5, Unigene13910\_TAEpaeTARAAPEI-5, Unigene14270\_TAEpaeTARAAPEI-5, Unigene14714\_TAEpaeTARAAPEI-5, Unigene14735\_TAEpaeTARAAPEI-5, Unigene15247\_TAEpaeTARAAPEI-5, Unigene15248\_TAEpaeTARAAPEI-5, Unigene15278\_TAEpaeTARAAPEI-5, Unigene15730\_TAEpaeTARAAPEI-5, Unigene15784\_TAEpaeTARAAPEI-5, Unigene15789\_TAEpaeTARAAPEI-5, Unigene15962\_TAEpaeTARAAPEI-5, Unigene16040\_TAEpaeTARAAPEI-5, Unigene16518\_TAEpaeTARAAPEI-5, Unigene16523\_TAEpaeTARAAPEI-5, Unigene1658\_TAEpaeTARAAPEI-5, Unigene16661\_TAEpaeTARAAPEI-5, Unigene16662\_TAEpaeTARAAPEI-5, Unigene16728\_TAEpaeTARAAPEI-5, Unigene16824\_TAEpaeTARAAPEI-5, Unigene17037\_TAEpaeTARAAPEI-5, Unigene17163\_TAEpaeTARAAPEI-5, Unigene17201\_TAEpaeTARAAPEI-5, Unigene17202\_TAEpaeTARAAPEI-5, Unigene17397\_TAEpaeTARAAPEI-5, Unigene17560\_TAEpaeTARAAPEI-5, Unigene17565\_TAEpaeTARAAPEI-5, Unigene17569\_TAEpaeTARAAPEI-5, Unigene17633\_TAEpaeTARAAPEI-5, Unigene17641\_TAEpaeTARAAPEI-5, Unigene17653\_TAEpaeTARAAPEI-5, Unigene17675\_TAEpaeTARAAPEI-5, Unigene17920\_TAEpaeTARAAPEI-5, Unigene17981\_TAEpaeTARAAPEI-5, Unigene18053\_TAEpaeTARAAPEI-5, Unigene19079\_TAEpaeTARAAPEI-5, Unigene19248\_TAEpaeTARAAPEI-5, Unigene19448\_TAEpaeTARAAPEI-5, Unigene19671\_TAEpaeTARAAPEI-5, Unigene19753\_TAEpaeTARAAPEI-5, Unigene19798\_TAEpaeTARAAPEI-5, Unigene19937\_TAEpaeTARAAPEI-5, Unigene20057\_TAEpaeTARAAPEI-5, Unigene2016\_TAEpaeTARAAPEI-5, Unigene20403\_TAEpaeTARAAPEI-5, Unigene20426\_TAEpaeTARAAPEI-5, Unigene21363\_TAEpaeTARAAPEI-5, Unigene21545\_TAEpaeTARAAPEI-5, Unigene22841\_TAEpaeTARAAPEI-5, Unigene22864\_TAEpaeTARAAPEI-5, Unigene23619\_TAEpaeTARAAPEI-5, Unigene24613\_TAEpaeTARAAPEI-5, Unigene24657\_TAEpaeTARAAPEI-5, Unigene24959\_TAEpaeTARAAPEI-5, Unigene25063\_TAEpaeTARAAPEI-5, Unigene25430\_TAEpaeTARAAPEI-5, Unigene25921\_TAEpaeTARAAPEI-5, Unigene25956\_TAEpaeTARAAPEI-5, Unigene26095\_TAEpaeTARAAPEI-5, Unigene26175\_TAEpaeTARAAPEI-5, Unigene26662\_TAEpaeTARAAPEI-5, Unigene26859\_TAEpaeTARAAPEI-5, Unigene26973\_TAEpaeTARAAPEI-5, Unigene27011\_TAEpaeTARAAPEI-5, Unigene27094\_TAEpaeTARAAPEI-5, Unigene27180\_TAEpaeTARAAPEI-5, Unigene27477\_TAEpaeTARAAPEI-5, Unigene27981\_TAEpaeTARAAPEI-5, Unigene28361\_TAEpaeTARAAPEI-5, Unigene2882\_TAEpaeTARAAPEI-5, Unigene28853\_TAEpaeTARAAPEI-5, Unigene29077\_TAEpaeTARAAPEI-5, Unigene29783\_TAEpaeTARAAPEI-5, Unigene301\_TAEpaeTARAAPEI-5, Unigene30229\_TAEpaeTARAAPEI-5, Unigene30279\_TAEpaeTARAAPEI-5, Unigene30803\_TAEpaeTARAAPEI-5, Unigene30828\_TAEpaeTARAAPEI-5, Unigene308\_TAEpaeTARAAPEI-5, Unigene31007\_TAEpaeTARAAPEI-5, Unigene31043\_TAEpaeTARAAPEI-5, Unigene31045\_TAEpaeTARAAPEI-5, Unigene31109\_TAEpaeTARAAPEI-5, Unigene3145\_TAEpaeTARAAPEI-5, Unigene31474\_TAEpaeTARAAPEI-5, Unigene31785\_TAEpaeTARAAPEI-5, Unigene31877\_TAEpaeTARAAPEI-5, Unigene31947\_TAEpaeTARAAPEI-5, Unigene32081\_TAEpaeTARAAPEI-5, Unigene3213\_TAEpaeTARAAPEI-5, Unigene32776\_TAEpaeTARAAPEI-5, Unigene33125\_TAEpaeTARAAPEI-5, Unigene33204\_TAEpaeTARAAPEI-5, Unigene3331\_TAEpaeTARAAPEI-5, Unigene34195\_TAEpaeTARAAPEI-5, Unigene34287\_TAEpaeTARAAPEI-5, Unigene34394\_TAEpaeTARAAPEI-5, Unigene34532\_TAEpaeTARAAPEI-5, Unigene34902\_TAEpaeTARAAPEI-5, Unigene35102\_TAEpaeTARAAPEI-5, Unigene3514\_TAEpaeTARAAPEI-5, Unigene35487\_TAEpaeTARAAPEI-5, Unigene35733\_TAEpaeTARAAPEI-5, Unigene35839\_TAEpaeTARAAPEI-5, Unigene36129\_TAEpaeTARAAPEI-5, Unigene36174\_TAEpaeTARAAPEI-5, Unigene36362\_TAEpaeTARAAPEI-5, Unigene36447\_TAEpaeTARAAPEI-5, Unigene37196\_TAEpaeTARAAPEI-5, Unigene37207\_TAEpaeTARAAPEI-5, Unigene37318\_TAEpaeTARAAPEI-5, Unigene37692\_TAEpaeTARAAPEI-5, Unigene37844\_TAEpaeTARAAPEI-5, Unigene37912\_TAEpaeTARAAPEI-5, Unigene38221\_TAEpaeTARAAPEI-5, Unigene38413\_TAEpaeTARAAPEI-5, Unigene39154\_TAEpaeTARAAPEI-5, Unigene39247\_TAEpaeTARAAPEI-5, Unigene40082\_TAEpaeTARAAPEI-5, Unigene40789\_TAEpaeTARAAPEI-5, Unigene40824\_TAEpaeTARAAPEI-5, Unigene4087\_TAEpaeTARAAPEI-5, Unigene40999\_TAEpaeTARAAPEI-5, Unigene41033\_TAEpaeTARAAPEI-5, Unigene41176\_TAEpaeTARAAPEI-5, Unigene41368\_TAEpaeTARAAPEI-5, Unigene41440\_TAEpaeTARAAPEI-5, Unigene41488\_TAEpaeTARAAPEI-5, Unigene41855\_TAEpaeTARAAPEI-5, Unigene42252\_TAEpaeTARAAPEI-5, Unigene42391\_TAEpaeTARAAPEI-5, Unigene42517\_TAEpaeTARAAPEI-5, Unigene42533\_TAEpaeTARAAPEI-5, Unigene42875\_TAEpaeTARAAPEI-5, Unigene4288\_TAEpaeTARAAPEI-5, Unigene43026\_TAEpaeTARAAPEI-5, Unigene43303\_TAEpaeTARAAPEI-5, Unigene43310\_TAEpaeTARAAPEI-5, Unigene43510\_TAEpaeTARAAPEI-5, Unigene43710\_TAEpaeTARAAPEI-5, Unigene4371\_TAEpaeTARAAPEI-5, Unigene43931\_TAEpaeTARAAPEI-5, Unigene44022\_TAEpaeTARAAPEI-5, Unigene44136\_TAEpaeTARAAPEI-5, Unigene44317\_TAEpaeTARAAPEI-5, Unigene44370\_TAEpaeTARAAPEI-5, Unigene44643\_TAEpaeTARAAPEI-5, Unigene44881\_TAEpaeTARAAPEI-5, Unigene44926\_TAEpaeTARAAPEI-5, Unigene45298\_TAEpaeTARAAPEI-5, Unigene45338\_TAEpaeTARAAPEI-5, Unigene45602\_TAEpaeTARAAPEI-5, Unigene45704\_TAEpaeTARAAPEI-5, Unigene45874\_TAEpaeTARAAPEI-5, Unigene46290\_TAEpaeTARAAPEI-5, Unigene46298\_TAEpaeTARAAPEI-5, Unigene46859\_TAEpaeTARAAPEI-5, Unigene47047\_TAEpaeTARAAPEI-5, Unigene47436\_TAEpaeTARAAPEI-5, Unigene47685\_TAEpaeTARAAPEI-5, Unigene48002\_TAEpaeTARAAPEI-5, Unigene480\_TAEpaeTARAAPEI-5, Unigene48237\_TAEpaeTARAAPEI-5, Unigene48432\_TAEpaeTARAAPEI-5, Unigene48472\_TAEpaeTARAAPEI-5, Unigene48580\_TAEpaeTARAAPEI-5, Unigene48589\_TAEpaeTARAAPEI-5, Unigene48711\_TAEpaeTARAAPEI-5, Unigene49023\_TAEpaeTARAAPEI-5, Unigene49045\_TAEpaeTARAAPEI-5, Unigene49085\_TAEpaeTARAAPEI-5, Unigene49173\_TAEpaeTARAAPEI-5, Unigene49966\_TAEpaeTARAAPEI-5, Unigene49990\_TAEpaeTARAAPEI-5, Unigene50283\_TAEpaeTARAAPEI-5, Unigene50473\_TAEpaeTARAAPEI-5, Unigene50609\_TAEpaeTARAAPEI-5, Unigene50648\_TAEpaeTARAAPEI-5, Unigene50937\_TAEpaeTARAAPEI-5, Unigene51056\_TAEpaeTARAAPEI-5, Unigene51253\_TAEpaeTARAAPEI-5, Unigene51368\_TAEpaeTARAAPEI-5, Unigene51697\_TAEpaeTARAAPEI-5, Unigene51974\_TAEpaeTARAAPEI-5, Unigene5198\_TAEpaeTARAAPEI-5, Unigene52138\_TAEpaeTARAAPEI-5, Unigene52308\_TAEpaeTARAAPEI-5, Unigene52312\_TAEpaeTARAAPEI-5, Unigene52325\_TAEpaeTARAAPEI-5, Unigene52350\_TAEpaeTARAAPEI-5, Unigene52394\_TAEpaeTARAAPEI-5, Unigene52891\_TAEpaeTARAAPEI-5, Unigene53106\_TAEpaeTARAAPEI-5, Unigene53203\_TAEpaeTARAAPEI-5, Unigene53341\_TAEpaeTARAAPEI-5, Unigene53426\_TAEpaeTARAAPEI-5, Unigene53911\_TAEpaeTARAAPEI-5, Unigene54058\_TAEpaeTARAAPEI-5, Unigene54066\_TAEpaeTARAAPEI-5, Unigene54261\_TAEpaeTARAAPEI-5, Unigene54490\_TAEpaeTARAAPEI-5, Unigene54725\_TAEpaeTARAAPEI-5, Unigene55063\_TAEpaeTARAAPEI-5, Unigene55108\_TAEpaeTARAAPEI-5, Unigene55277\_TAEpaeTARAAPEI-5, Unigene55578\_TAEpaeTARAAPEI-5, Unigene55786\_TAEpaeTARAAPEI-5, Unigene55811\_TAEpaeTARAAPEI-5, Unigene55958\_TAEpaeTARAAPEI-5, Unigene56010\_TAEpaeTARAAPEI-5, Unigene56207\_TAEpaeTARAAPEI-5, Unigene5655\_TAEpaeTARAAPEI-5, Unigene56822\_TAEpaeTARAAPEI-5, Unigene56860\_TAEpaeTARAAPEI-5, Unigene56874\_TAEpaeTARAAPEI-5, Unigene56925\_TAEpaeTARAAPEI-5, Unigene56955\_TAEpaeTARAAPEI-5, Unigene57151\_TAEpaeTARAAPEI-5, Unigene57371\_TAEpaeTARAAPEI-5, Unigene57397\_TAEpaeTARAAPEI-5, Unigene57696\_TAEpaeTARAAPEI-5, Unigene57718\_TAEpaeTARAAPEI-5, Unigene57728\_TAEpaeTARAAPEI-5, Unigene57831\_TAEpaeTARAAPEI-5, Unigene57902\_TAEpaeTARAAPEI-5, Unigene57972\_TAEpaeTARAAPEI-5, Unigene57983\_TAEpaeTARAAPEI-5, Unigene58027\_TAEpaeTARAAPEI-5, Unigene58223\_TAEpaeTARAAPEI-5, Unigene58245\_TAEpaeTARAAPEI-5, Unigene58386\_TAEpaeTARAAPEI-5, Unigene58481\_TAEpaeTARAAPEI-5, Unigene5848\_TAEpaeTARAAPEI-5, Unigene58533\_TAEpaeTARAAPEI-5, Unigene58660\_TAEpaeTARAAPEI-5, Unigene58703\_TAEpaeTARAAPEI-5, Unigene58782\_TAEpaeTARAAPEI-5, Unigene58791\_TAEpaeTARAAPEI-5, Unigene58848\_TAEpaeTARAAPEI-5, Unigene58991\_TAEpaeTARAAPEI-5, Unigene59181\_TAEpaeTARAAPEI-5, Unigene59280\_TAEpaeTARAAPEI-5, Unigene5934\_TAEpaeTARAAPEI-5, Unigene59615\_TAEpaeTARAAPEI-5, Unigene5970\_TAEpaeTARAAPEI-5, Unigene59922\_TAEpaeTARAAPEI-5, Unigene60091\_TAEpaeTARAAPEI-5, Unigene60271\_TAEpaeTARAAPEI-5, Unigene6047\_TAEpaeTARAAPEI-5, Unigene60761\_TAEpaeTARAAPEI-5, Unigene60831\_TAEpaeTARAAPEI-5, Unigene60891\_TAEpaeTARAAPEI-5, Unigene61376\_TAEpaeTARAAPEI-5, Unigene6152\_TAEpaeTARAAPEI-5, Unigene61532\_TAEpaeTARAAPEI-5, Unigene61552\_TAEpaeTARAAPEI-5, Unigene61894\_TAEpaeTARAAPEI-5, Unigene62281\_TAEpaeTARAAPEI-5, Unigene62417\_TAEpaeTARAAPEI-5, Unigene62439\_TAEpaeTARAAPEI-5, Unigene62472\_TAEpaeTARAAPEI-5, Unigene62641\_TAEpaeTARAAPEI-5, Unigene62962\_TAEpaeTARAAPEI-5, Unigene62978\_TAEpaeTARAAPEI-5, Unigene63027\_TAEpaeTARAAPEI-5, Unigene63051\_TAEpaeTARAAPEI-5, Unigene63172\_TAEpaeTARAAPEI-5, Unigene63253\_TAEpaeTARAAPEI-5, Unigene6330\_TAEpaeTARAAPEI-5, Unigene63328\_TAEpaeTARAAPEI-5, Unigene63387\_TAEpaeTARAAPEI-5, Unigene63582\_TAEpaeTARAAPEI-5, Unigene63809\_TAEpaeTARAAPEI-5, Unigene63980\_TAEpaeTARAAPEI-5, Unigene64009\_TAEpaeTARAAPEI-5, Unigene64012\_TAEpaeTARAAPEI-5, Unigene64088\_TAEpaeTARAAPEI-5, Unigene6422\_TAEpaeTARAAPEI-5, Unigene6452\_TAEpaeTARAAPEI-5, Unigene64931\_TAEpaeTARAAPEI-5, Unigene64972\_TAEpaeTARAAPEI-5, Unigene65457\_TAEpaeTARAAPEI-5, Unigene65468\_TAEpaeTARAAPEI-5, Unigene65593\_TAEpaeTARAAPEI-5, Unigene65663\_TAEpaeTARAAPEI-5, Unigene65717\_TAEpaeTARAAPEI-5, Unigene65964\_TAEpaeTARAAPEI-5, Unigene66324\_TAEpaeTARAAPEI-5, Unigene66382\_TAEpaeTARAAPEI-5, Unigene663\_TAEpaeTARAAPEI-5, Unigene66490\_TAEpaeTARAAPEI-5, Unigene66526\_TAEpaeTARAAPEI-5, Unigene66532\_TAEpaeTARAAPEI-5, Unigene66648\_TAEpaeTARAAPEI-5, Unigene66742\_TAEpaeTARAAPEI-5, Unigene67043\_TAEpaeTARAAPEI-5, Unigene67172\_TAEpaeTARAAPEI-5, Unigene67359\_TAEpaeTARAAPEI-5, Unigene67392\_TAEpaeTARAAPEI-5, Unigene67445\_TAEpaeTARAAPEI-5, Unigene67579\_TAEpaeTARAAPEI-5, Unigene67884\_TAEpaeTARAAPEI-5, Unigene68026\_TAEpaeTARAAPEI-5, Unigene68121\_TAEpaeTARAAPEI-5, Unigene68146\_TAEpaeTARAAPEI-5, Unigene68284\_TAEpaeTARAAPEI-5, Unigene68350\_TAEpaeTARAAPEI-5, Unigene68458\_TAEpaeTARAAPEI-5, Unigene68702\_TAEpaeTARAAPEI-5, Unigene68807\_TAEpaeTARAAPEI-5, Unigene68869\_TAEpaeTARAAPEI-5, Unigene68878\_TAEpaeTARAAPEI-5, Unigene69043\_TAEpaeTARAAPEI-5, Unigene69072\_TAEpaeTARAAPEI-5, Unigene69153\_TAEpaeTARAAPEI-5, Unigene69180\_TAEpaeTARAAPEI-5, Unigene69210\_TAEpaeTARAAPEI-5, Unigene69231\_TAEpaeTARAAPEI-5, Unigene69257\_TAEpaeTARAAPEI-5, Unigene69400\_TAEpaeTARAAPEI-5, Unigene69441\_TAEpaeTARAAPEI-5, Unigene69461\_TAEpaeTARAAPEI-5, Unigene69512\_TAEpaeTARAAPEI-5, Unigene69517\_TAEpaeTARAAPEI-5, Unigene69638\_TAEpaeTARAAPEI-5, Unigene69646\_TAEpaeTARAAPEI-5, Unigene69666\_TAEpaeTARAAPEI-5, Unigene69688\_TAEpaeTARAAPEI-5, Unigene69764\_TAEpaeTARAAPEI-5, Unigene70091\_TAEpaeTARAAPEI-5, Unigene70161\_TAEpaeTARAAPEI-5, Unigene70207\_TAEpaeTARAAPEI-5, Unigene70224\_TAEpaeTARAAPEI-5, Unigene70337\_TAEpaeTARAAPEI-5, Unigene70392\_TAEpaeTARAAPEI-5, Unigene70543\_TAEpaeTARAAPEI-5, Unigene70604\_TAEpaeTARAAPEI-5, Unigene70626\_TAEpaeTARAAPEI-5, Unigene70836\_TAEpaeTARAAPEI-5, Unigene70968\_TAEpaeTARAAPEI-5, Unigene70994\_TAEpaeTARAAPEI-5, Unigene71050\_TAEpaeTARAAPEI-5, Unigene71075\_TAEpaeTARAAPEI-5, Unigene71141\_TAEpaeTARAAPEI-5, Unigene71213\_TAEpaeTARAAPEI-5, Unigene71223\_TAEpaeTARAAPEI-5, Unigene71278\_TAEpaeTARAAPEI-5, Unigene71379\_TAEpaeTARAAPEI-5, Unigene71440\_TAEpaeTARAAPEI-5, Unigene71472\_TAEpaeTARAAPEI-5, Unigene71632\_TAEpaeTARAAPEI-5, Unigene71635\_TAEpaeTARAAPEI-5, Unigene71684\_TAEpaeTARAAPEI-5, Unigene71690\_TAEpaeTARAAPEI-5, Unigene71756\_TAEpaeTARAAPEI-5, Unigene71774\_TAEpaeTARAAPEI-5, Unigene71779\_TAEpaeTARAAPEI-5, Unigene71841\_TAEpaeTARAAPEI-5, Unigene71868\_TAEpaeTARAAPEI-5, Unigene71893\_TAEpaeTARAAPEI-5, Unigene71906\_TAEpaeTARAAPEI-5, Unigene71915\_TAEpaeTARAAPEI-5, Unigene71945\_TAEpaeTARAAPEI-5, Unigene71948\_TAEpaeTARAAPEI-5, Unigene71969\_TAEpaeTARAAPEI-5, Unigene72000\_TAEpaeTARAAPEI-5, Unigene72105\_TAEpaeTARAAPEI-5, Unigene72109\_TAEpaeTARAAPEI-5, Unigene72155\_TAEpaeTARAAPEI-5, Unigene72213\_TAEpaeTARAAPEI-5, Unigene72258\_TAEpaeTARAAPEI-5, Unigene72263\_TAEpaeTARAAPEI-5, Unigene72304\_TAEpaeTARAAPEI-5, Unigene72331\_TAEpaeTARAAPEI-5, Unigene72479\_TAEpaeTARAAPEI-5, Unigene72494\_TAEpaeTARAAPEI-5, Unigene72588\_TAEpaeTARAAPEI-5, Unigene72691\_TAEpaeTARAAPEI-5, Unigene72785\_TAEpaeTARAAPEI-5, Unigene72821\_TAEpaeTARAAPEI-5, Unigene7627\_TAEpaeTARAAPEI-5, Unigene7662\_TAEpaeTARAAPEI-5, Unigene7739\_TAEpaeTARAAPEI-5, Unigene8505\_TAEpaeTARAAPEI-5, Unigene8920\_TAEpaeTARAAPEI-5, Unigene9120\_TAEpaeTARAAPEI-5, Unigene9332\_TAEpaeTARAAPEI-5, Unigene9339\_TAEpaeTARAAPEI-5, Unigene9577\_TAEpaeTARAAPEI-5, Unigene9701\_TAEpaeTARAAPEI-5, Unigene9705\_TAEpaeTARAAPEI-5 |
| 14 | Vascular smooth muscle contraction | Unigene10142\_TAEpaeTARAAPEI-5, Unigene10245\_TAEpaeTARAAPEI-5, Unigene10302\_TAEpaeTARAAPEI-5, Unigene10544\_TAEpaeTARAAPEI-5, Unigene10547\_TAEpaeTARAAPEI-5, Unigene10658\_TAEpaeTARAAPEI-5, Unigene10777\_TAEpaeTARAAPEI-5, Unigene10835\_TAEpaeTARAAPEI-5, Unigene10848\_TAEpaeTARAAPEI-5, Unigene10863\_TAEpaeTARAAPEI-5, Unigene1087\_TAEpaeTARAAPEI-5, Unigene11280\_TAEpaeTARAAPEI-5, Unigene11418\_TAEpaeTARAAPEI-5, Unigene12023\_TAEpaeTARAAPEI-5, Unigene12145\_TAEpaeTARAAPEI-5, Unigene12247\_TAEpaeTARAAPEI-5, Unigene12332\_TAEpaeTARAAPEI-5, Unigene12401\_TAEpaeTARAAPEI-5, Unigene12478\_TAEpaeTARAAPEI-5, Unigene12878\_TAEpaeTARAAPEI-5, Unigene12921\_TAEpaeTARAAPEI-5, Unigene12956\_TAEpaeTARAAPEI-5, Unigene13000\_TAEpaeTARAAPEI-5, Unigene13058\_TAEpaeTARAAPEI-5, Unigene13163\_TAEpaeTARAAPEI-5, Unigene13484\_TAEpaeTARAAPEI-5, Unigene13532\_TAEpaeTARAAPEI-5, Unigene13555\_TAEpaeTARAAPEI-5, Unigene13629\_TAEpaeTARAAPEI-5, Unigene13650\_TAEpaeTARAAPEI-5, Unigene13747\_TAEpaeTARAAPEI-5, Unigene13877\_TAEpaeTARAAPEI-5, Unigene14270\_TAEpaeTARAAPEI-5, Unigene14330\_TAEpaeTARAAPEI-5, Unigene14347\_TAEpaeTARAAPEI-5, Unigene14362\_TAEpaeTARAAPEI-5, Unigene14366\_TAEpaeTARAAPEI-5, Unigene14384\_TAEpaeTARAAPEI-5, Unigene14408\_TAEpaeTARAAPEI-5, Unigene14528\_TAEpaeTARAAPEI-5, Unigene14585\_TAEpaeTARAAPEI-5, Unigene14644\_TAEpaeTARAAPEI-5, Unigene14687\_TAEpaeTARAAPEI-5, Unigene14809\_TAEpaeTARAAPEI-5, Unigene14940\_TAEpaeTARAAPEI-5, Unigene15087\_TAEpaeTARAAPEI-5, Unigene15247\_TAEpaeTARAAPEI-5, Unigene15361\_TAEpaeTARAAPEI-5, Unigene15468\_TAEpaeTARAAPEI-5, Unigene15549\_TAEpaeTARAAPEI-5, Unigene15730\_TAEpaeTARAAPEI-5, Unigene15881\_TAEpaeTARAAPEI-5, Unigene1593\_TAEpaeTARAAPEI-5, Unigene15971\_TAEpaeTARAAPEI-5, Unigene16060\_TAEpaeTARAAPEI-5, Unigene16406\_TAEpaeTARAAPEI-5, Unigene16411\_TAEpaeTARAAPEI-5, Unigene16468\_TAEpaeTARAAPEI-5, Unigene1657\_TAEpaeTARAAPEI-5, Unigene16712\_TAEpaeTARAAPEI-5, Unigene16824\_TAEpaeTARAAPEI-5, Unigene17109\_TAEpaeTARAAPEI-5, Unigene17130\_TAEpaeTARAAPEI-5, Unigene17145\_TAEpaeTARAAPEI-5, Unigene17252\_TAEpaeTARAAPEI-5, Unigene17354\_TAEpaeTARAAPEI-5, Unigene17420\_TAEpaeTARAAPEI-5, Unigene17551\_TAEpaeTARAAPEI-5, Unigene17569\_TAEpaeTARAAPEI-5, Unigene17644\_TAEpaeTARAAPEI-5, Unigene17673\_TAEpaeTARAAPEI-5, Unigene17700\_TAEpaeTARAAPEI-5, Unigene17731\_TAEpaeTARAAPEI-5, Unigene17741\_TAEpaeTARAAPEI-5, Unigene17865\_TAEpaeTARAAPEI-5, Unigene17881\_TAEpaeTARAAPEI-5, Unigene17901\_TAEpaeTARAAPEI-5, Unigene17934\_TAEpaeTARAAPEI-5, Unigene17977\_TAEpaeTARAAPEI-5, Unigene19091\_TAEpaeTARAAPEI-5, Unigene19223\_TAEpaeTARAAPEI-5, Unigene19396\_TAEpaeTARAAPEI-5, Unigene19937\_TAEpaeTARAAPEI-5, Unigene20093\_TAEpaeTARAAPEI-5, Unigene20403\_TAEpaeTARAAPEI-5, Unigene21363\_TAEpaeTARAAPEI-5, Unigene21445\_TAEpaeTARAAPEI-5, Unigene21878\_TAEpaeTARAAPEI-5, Unigene23436\_TAEpaeTARAAPEI-5, Unigene23829\_TAEpaeTARAAPEI-5, Unigene24012\_TAEpaeTARAAPEI-5, Unigene2429\_TAEpaeTARAAPEI-5, Unigene24657\_TAEpaeTARAAPEI-5, Unigene24741\_TAEpaeTARAAPEI-5, Unigene25004\_TAEpaeTARAAPEI-5, Unigene25505\_TAEpaeTARAAPEI-5, Unigene25611\_TAEpaeTARAAPEI-5, Unigene25948\_TAEpaeTARAAPEI-5, Unigene25970\_TAEpaeTARAAPEI-5, Unigene26031\_TAEpaeTARAAPEI-5, Unigene26662\_TAEpaeTARAAPEI-5, Unigene26859\_TAEpaeTARAAPEI-5, Unigene27281\_TAEpaeTARAAPEI-5, Unigene29119\_TAEpaeTARAAPEI-5, Unigene29157\_TAEpaeTARAAPEI-5, Unigene29171\_TAEpaeTARAAPEI-5, Unigene30172\_TAEpaeTARAAPEI-5, Unigene30199\_TAEpaeTARAAPEI-5, Unigene301\_TAEpaeTARAAPEI-5, Unigene30758\_TAEpaeTARAAPEI-5, Unigene30885\_TAEpaeTARAAPEI-5, Unigene30916\_TAEpaeTARAAPEI-5, Unigene31043\_TAEpaeTARAAPEI-5, Unigene3145\_TAEpaeTARAAPEI-5, Unigene31474\_TAEpaeTARAAPEI-5, Unigene3158\_TAEpaeTARAAPEI-5, Unigene31750\_TAEpaeTARAAPEI-5, Unigene31929\_TAEpaeTARAAPEI-5, Unigene32294\_TAEpaeTARAAPEI-5, Unigene32625\_TAEpaeTARAAPEI-5, Unigene32782\_TAEpaeTARAAPEI-5, Unigene33074\_TAEpaeTARAAPEI-5, Unigene33125\_TAEpaeTARAAPEI-5, Unigene33549\_TAEpaeTARAAPEI-5, Unigene33982\_TAEpaeTARAAPEI-5, Unigene34195\_TAEpaeTARAAPEI-5, Unigene35030\_TAEpaeTARAAPEI-5, Unigene35225\_TAEpaeTARAAPEI-5, Unigene35733\_TAEpaeTARAAPEI-5, Unigene35882\_TAEpaeTARAAPEI-5, Unigene36230\_TAEpaeTARAAPEI-5, Unigene36858\_TAEpaeTARAAPEI-5, Unigene37328\_TAEpaeTARAAPEI-5, Unigene38044\_TAEpaeTARAAPEI-5, Unigene3828\_TAEpaeTARAAPEI-5, Unigene38667\_TAEpaeTARAAPEI-5, Unigene39254\_TAEpaeTARAAPEI-5, Unigene39396\_TAEpaeTARAAPEI-5, Unigene39638\_TAEpaeTARAAPEI-5, Unigene39872\_TAEpaeTARAAPEI-5, Unigene39874\_TAEpaeTARAAPEI-5, Unigene40073\_TAEpaeTARAAPEI-5, Unigene40571\_TAEpaeTARAAPEI-5, Unigene40826\_TAEpaeTARAAPEI-5, Unigene41673\_TAEpaeTARAAPEI-5, Unigene42414\_TAEpaeTARAAPEI-5, Unigene43310\_TAEpaeTARAAPEI-5, Unigene43326\_TAEpaeTARAAPEI-5, Unigene43663\_TAEpaeTARAAPEI-5, Unigene43750\_TAEpaeTARAAPEI-5, Unigene4380\_TAEpaeTARAAPEI-5, Unigene4423\_TAEpaeTARAAPEI-5, Unigene44324\_TAEpaeTARAAPEI-5, Unigene44401\_TAEpaeTARAAPEI-5, Unigene44643\_TAEpaeTARAAPEI-5, Unigene45140\_TAEpaeTARAAPEI-5, Unigene45338\_TAEpaeTARAAPEI-5, Unigene45410\_TAEpaeTARAAPEI-5, Unigene45651\_TAEpaeTARAAPEI-5, Unigene45682\_TAEpaeTARAAPEI-5, Unigene45779\_TAEpaeTARAAPEI-5, Unigene45803\_TAEpaeTARAAPEI-5, Unigene46174\_TAEpaeTARAAPEI-5, Unigene46530\_TAEpaeTARAAPEI-5, Unigene46659\_TAEpaeTARAAPEI-5, Unigene4686\_TAEpaeTARAAPEI-5, Unigene47023\_TAEpaeTARAAPEI-5, Unigene47434\_TAEpaeTARAAPEI-5, Unigene47954\_TAEpaeTARAAPEI-5, Unigene48267\_TAEpaeTARAAPEI-5, Unigene48394\_TAEpaeTARAAPEI-5, Unigene48695\_TAEpaeTARAAPEI-5, Unigene48711\_TAEpaeTARAAPEI-5, Unigene49023\_TAEpaeTARAAPEI-5, Unigene49590\_TAEpaeTARAAPEI-5, Unigene49971\_TAEpaeTARAAPEI-5, Unigene49981\_TAEpaeTARAAPEI-5, Unigene49990\_TAEpaeTARAAPEI-5, Unigene50374\_TAEpaeTARAAPEI-5, Unigene50473\_TAEpaeTARAAPEI-5, Unigene50474\_TAEpaeTARAAPEI-5, Unigene51724\_TAEpaeTARAAPEI-5, Unigene51767\_TAEpaeTARAAPEI-5, Unigene51813\_TAEpaeTARAAPEI-5, Unigene52312\_TAEpaeTARAAPEI-5, Unigene52429\_TAEpaeTARAAPEI-5, Unigene53106\_TAEpaeTARAAPEI-5, Unigene53786\_TAEpaeTARAAPEI-5, Unigene5380\_TAEpaeTARAAPEI-5, Unigene54206\_TAEpaeTARAAPEI-5, Unigene54313\_TAEpaeTARAAPEI-5, Unigene55063\_TAEpaeTARAAPEI-5, Unigene55192\_TAEpaeTARAAPEI-5, Unigene55252\_TAEpaeTARAAPEI-5, Unigene5555\_TAEpaeTARAAPEI-5, Unigene55789\_TAEpaeTARAAPEI-5, Unigene55790\_TAEpaeTARAAPEI-5, Unigene56010\_TAEpaeTARAAPEI-5, Unigene56014\_TAEpaeTARAAPEI-5, Unigene56500\_TAEpaeTARAAPEI-5, Unigene56748\_TAEpaeTARAAPEI-5, Unigene56770\_TAEpaeTARAAPEI-5, Unigene56860\_TAEpaeTARAAPEI-5, Unigene56929\_TAEpaeTARAAPEI-5, Unigene57365\_TAEpaeTARAAPEI-5, Unigene57371\_TAEpaeTARAAPEI-5, Unigene57376\_TAEpaeTARAAPEI-5, Unigene57397\_TAEpaeTARAAPEI-5, Unigene57496\_TAEpaeTARAAPEI-5, Unigene5753\_TAEpaeTARAAPEI-5, Unigene57607\_TAEpaeTARAAPEI-5, Unigene57969\_TAEpaeTARAAPEI-5, Unigene58297\_TAEpaeTARAAPEI-5, Unigene5848\_TAEpaeTARAAPEI-5, Unigene58533\_TAEpaeTARAAPEI-5, Unigene58665\_TAEpaeTARAAPEI-5, Unigene58782\_TAEpaeTARAAPEI-5, Unigene58938\_TAEpaeTARAAPEI-5, Unigene59280\_TAEpaeTARAAPEI-5, Unigene59373\_TAEpaeTARAAPEI-5, Unigene59529\_TAEpaeTARAAPEI-5, Unigene59670\_TAEpaeTARAAPEI-5, Unigene59674\_TAEpaeTARAAPEI-5, Unigene60152\_TAEpaeTARAAPEI-5, Unigene6030\_TAEpaeTARAAPEI-5, Unigene60330\_TAEpaeTARAAPEI-5, Unigene60534\_TAEpaeTARAAPEI-5, Unigene6092\_TAEpaeTARAAPEI-5, Unigene60986\_TAEpaeTARAAPEI-5, Unigene60992\_TAEpaeTARAAPEI-5, Unigene61108\_TAEpaeTARAAPEI-5, Unigene61114\_TAEpaeTARAAPEI-5, Unigene61338\_TAEpaeTARAAPEI-5, Unigene61489\_TAEpaeTARAAPEI-5, Unigene6150\_TAEpaeTARAAPEI-5, Unigene61652\_TAEpaeTARAAPEI-5, Unigene61806\_TAEpaeTARAAPEI-5, Unigene61820\_TAEpaeTARAAPEI-5, Unigene61823\_TAEpaeTARAAPEI-5, Unigene61890\_TAEpaeTARAAPEI-5, Unigene61894\_TAEpaeTARAAPEI-5, Unigene62064\_TAEpaeTARAAPEI-5, Unigene62217\_TAEpaeTARAAPEI-5, Unigene62275\_TAEpaeTARAAPEI-5, Unigene62489\_TAEpaeTARAAPEI-5, Unigene63148\_TAEpaeTARAAPEI-5, Unigene63153\_TAEpaeTARAAPEI-5, Unigene63172\_TAEpaeTARAAPEI-5, Unigene63253\_TAEpaeTARAAPEI-5, Unigene63298\_TAEpaeTARAAPEI-5, Unigene63328\_TAEpaeTARAAPEI-5, Unigene63370\_TAEpaeTARAAPEI-5, Unigene63387\_TAEpaeTARAAPEI-5, Unigene63485\_TAEpaeTARAAPEI-5, Unigene6372\_TAEpaeTARAAPEI-5, Unigene63809\_TAEpaeTARAAPEI-5, Unigene64009\_TAEpaeTARAAPEI-5, Unigene64029\_TAEpaeTARAAPEI-5, Unigene64040\_TAEpaeTARAAPEI-5, Unigene64052\_TAEpaeTARAAPEI-5, Unigene64150\_TAEpaeTARAAPEI-5, Unigene64268\_TAEpaeTARAAPEI-5, Unigene64293\_TAEpaeTARAAPEI-5, Unigene64361\_TAEpaeTARAAPEI-5, Unigene64439\_TAEpaeTARAAPEI-5, Unigene6449\_TAEpaeTARAAPEI-5, Unigene64529\_TAEpaeTARAAPEI-5, Unigene6452\_TAEpaeTARAAPEI-5, Unigene64673\_TAEpaeTARAAPEI-5, Unigene64687\_TAEpaeTARAAPEI-5, Unigene65037\_TAEpaeTARAAPEI-5, Unigene65314\_TAEpaeTARAAPEI-5, Unigene65322\_TAEpaeTARAAPEI-5, Unigene65468\_TAEpaeTARAAPEI-5, Unigene65570\_TAEpaeTARAAPEI-5, Unigene65685\_TAEpaeTARAAPEI-5, Unigene65700\_TAEpaeTARAAPEI-5, Unigene66043\_TAEpaeTARAAPEI-5, Unigene6605\_TAEpaeTARAAPEI-5, Unigene66212\_TAEpaeTARAAPEI-5, Unigene6635\_TAEpaeTARAAPEI-5, Unigene66490\_TAEpaeTARAAPEI-5, Unigene66620\_TAEpaeTARAAPEI-5, Unigene66648\_TAEpaeTARAAPEI-5, Unigene66694\_TAEpaeTARAAPEI-5, Unigene66931\_TAEpaeTARAAPEI-5, Unigene67063\_TAEpaeTARAAPEI-5, Unigene67221\_TAEpaeTARAAPEI-5, Unigene67311\_TAEpaeTARAAPEI-5, Unigene67341\_TAEpaeTARAAPEI-5, Unigene6745\_TAEpaeTARAAPEI-5, Unigene67524\_TAEpaeTARAAPEI-5, Unigene67579\_TAEpaeTARAAPEI-5, Unigene67745\_TAEpaeTARAAPEI-5, Unigene67776\_TAEpaeTARAAPEI-5, Unigene68154\_TAEpaeTARAAPEI-5, Unigene68205\_TAEpaeTARAAPEI-5, Unigene68239\_TAEpaeTARAAPEI-5, Unigene68507\_TAEpaeTARAAPEI-5, Unigene68625\_TAEpaeTARAAPEI-5, Unigene68778\_TAEpaeTARAAPEI-5, Unigene68799\_TAEpaeTARAAPEI-5, Unigene68839\_TAEpaeTARAAPEI-5, Unigene68874\_TAEpaeTARAAPEI-5, Unigene68878\_TAEpaeTARAAPEI-5, Unigene68984\_TAEpaeTARAAPEI-5, Unigene69062\_TAEpaeTARAAPEI-5, Unigene69101\_TAEpaeTARAAPEI-5, Unigene69257\_TAEpaeTARAAPEI-5, Unigene69275\_TAEpaeTARAAPEI-5, Unigene69370\_TAEpaeTARAAPEI-5, Unigene6938\_TAEpaeTARAAPEI-5, Unigene69421\_TAEpaeTARAAPEI-5, Unigene69441\_TAEpaeTARAAPEI-5, Unigene69461\_TAEpaeTARAAPEI-5, Unigene69489\_TAEpaeTARAAPEI-5, Unigene69517\_TAEpaeTARAAPEI-5, Unigene69681\_TAEpaeTARAAPEI-5, Unigene69688\_TAEpaeTARAAPEI-5, Unigene69776\_TAEpaeTARAAPEI-5, Unigene69780\_TAEpaeTARAAPEI-5, Unigene69849\_TAEpaeTARAAPEI-5, Unigene70019\_TAEpaeTARAAPEI-5, Unigene70033\_TAEpaeTARAAPEI-5, Unigene70085\_TAEpaeTARAAPEI-5, Unigene70103\_TAEpaeTARAAPEI-5, Unigene70104\_TAEpaeTARAAPEI-5, Unigene7011\_TAEpaeTARAAPEI-5, Unigene70145\_TAEpaeTARAAPEI-5, Unigene70176\_TAEpaeTARAAPEI-5, Unigene70309\_TAEpaeTARAAPEI-5, Unigene70347\_TAEpaeTARAAPEI-5, Unigene70372\_TAEpaeTARAAPEI-5, Unigene70571\_TAEpaeTARAAPEI-5, Unigene70607\_TAEpaeTARAAPEI-5, Unigene70615\_TAEpaeTARAAPEI-5, Unigene70761\_TAEpaeTARAAPEI-5, Unigene71038\_TAEpaeTARAAPEI-5, Unigene71120\_TAEpaeTARAAPEI-5, Unigene71122\_TAEpaeTARAAPEI-5, Unigene71141\_TAEpaeTARAAPEI-5, Unigene71171\_TAEpaeTARAAPEI-5, Unigene71173\_TAEpaeTARAAPEI-5, Unigene71180\_TAEpaeTARAAPEI-5, Unigene71182\_TAEpaeTARAAPEI-5, Unigene71305\_TAEpaeTARAAPEI-5, Unigene71310\_TAEpaeTARAAPEI-5, Unigene71472\_TAEpaeTARAAPEI-5, Unigene71553\_TAEpaeTARAAPEI-5, Unigene71632\_TAEpaeTARAAPEI-5, Unigene71682\_TAEpaeTARAAPEI-5, Unigene71835\_TAEpaeTARAAPEI-5, Unigene71839\_TAEpaeTARAAPEI-5, Unigene72087\_TAEpaeTARAAPEI-5, Unigene72092\_TAEpaeTARAAPEI-5, Unigene72109\_TAEpaeTARAAPEI-5, Unigene72181\_TAEpaeTARAAPEI-5, Unigene72213\_TAEpaeTARAAPEI-5, Unigene72266\_TAEpaeTARAAPEI-5, Unigene72340\_TAEpaeTARAAPEI-5, Unigene72355\_TAEpaeTARAAPEI-5, Unigene72392\_TAEpaeTARAAPEI-5, Unigene72399\_TAEpaeTARAAPEI-5, Unigene72451\_TAEpaeTARAAPEI-5, Unigene72473\_TAEpaeTARAAPEI-5, Unigene72540\_TAEpaeTARAAPEI-5, Unigene72569\_TAEpaeTARAAPEI-5, Unigene72588\_TAEpaeTARAAPEI-5, Unigene72593\_TAEpaeTARAAPEI-5, Unigene72607\_TAEpaeTARAAPEI-5, Unigene72635\_TAEpaeTARAAPEI-5, Unigene72670\_TAEpaeTARAAPEI-5, Unigene72690\_TAEpaeTARAAPEI-5, Unigene72702\_TAEpaeTARAAPEI-5, Unigene72707\_TAEpaeTARAAPEI-5, Unigene72711\_TAEpaeTARAAPEI-5, Unigene72749\_TAEpaeTARAAPEI-5, Unigene72823\_TAEpaeTARAAPEI-5, Unigene72849\_TAEpaeTARAAPEI-5, Unigene72896\_TAEpaeTARAAPEI-5, Unigene72903\_TAEpaeTARAAPEI-5, Unigene72939\_TAEpaeTARAAPEI-5, Unigene8211\_TAEpaeTARAAPEI-5, Unigene8411\_TAEpaeTARAAPEI-5, Unigene8505\_TAEpaeTARAAPEI-5, Unigene8562\_TAEpaeTARAAPEI-5, Unigene907\_TAEpaeTARAAPEI-5, Unigene9460\_TAEpaeTARAAPEI-5, Unigene9546\_TAEpaeTARAAPEI-5, Unigene9701\_TAEpaeTARAAPEI-5, Unigene9827\_TAEpaeTARAAPEI-5, Unigene9941\_TAEpaeTARAAPEI-5 |
| 15 | Ubiquitin mediated proteolysis | Unigene10090\_TAEpaeTARAAPEI-5, Unigene10141\_TAEpaeTARAAPEI-5, Unigene10539\_TAEpaeTARAAPEI-5, Unigene10551\_TAEpaeTARAAPEI-5, Unigene11123\_TAEpaeTARAAPEI-5, Unigene11132\_TAEpaeTARAAPEI-5, Unigene11172\_TAEpaeTARAAPEI-5, Unigene11381\_TAEpaeTARAAPEI-5, Unigene11839\_TAEpaeTARAAPEI-5, Unigene11880\_TAEpaeTARAAPEI-5, Unigene12199\_TAEpaeTARAAPEI-5, Unigene12203\_TAEpaeTARAAPEI-5, Unigene12381\_TAEpaeTARAAPEI-5, Unigene12421\_TAEpaeTARAAPEI-5, Unigene12495\_TAEpaeTARAAPEI-5, Unigene12618\_TAEpaeTARAAPEI-5, Unigene12675\_TAEpaeTARAAPEI-5, Unigene12719\_TAEpaeTARAAPEI-5, Unigene12835\_TAEpaeTARAAPEI-5, Unigene12970\_TAEpaeTARAAPEI-5, Unigene13015\_TAEpaeTARAAPEI-5, Unigene13721\_TAEpaeTARAAPEI-5, Unigene13923\_TAEpaeTARAAPEI-5, Unigene1435\_TAEpaeTARAAPEI-5, Unigene14416\_TAEpaeTARAAPEI-5, Unigene14481\_TAEpaeTARAAPEI-5, Unigene14782\_TAEpaeTARAAPEI-5, Unigene14943\_TAEpaeTARAAPEI-5, Unigene15060\_TAEpaeTARAAPEI-5, Unigene15181\_TAEpaeTARAAPEI-5, Unigene15186\_TAEpaeTARAAPEI-5, Unigene15194\_TAEpaeTARAAPEI-5, Unigene15269\_TAEpaeTARAAPEI-5, Unigene15439\_TAEpaeTARAAPEI-5, Unigene15450\_TAEpaeTARAAPEI-5, Unigene15750\_TAEpaeTARAAPEI-5, Unigene16013\_TAEpaeTARAAPEI-5, Unigene16386\_TAEpaeTARAAPEI-5, Unigene16610\_TAEpaeTARAAPEI-5, Unigene16892\_TAEpaeTARAAPEI-5, Unigene16986\_TAEpaeTARAAPEI-5, Unigene17044\_TAEpaeTARAAPEI-5, Unigene17221\_TAEpaeTARAAPEI-5, Unigene17358\_TAEpaeTARAAPEI-5, Unigene17498\_TAEpaeTARAAPEI-5, Unigene17614\_TAEpaeTARAAPEI-5, Unigene17648\_TAEpaeTARAAPEI-5, Unigene17680\_TAEpaeTARAAPEI-5, Unigene17718\_TAEpaeTARAAPEI-5, Unigene17746\_TAEpaeTARAAPEI-5, Unigene17868\_TAEpaeTARAAPEI-5, Unigene17912\_TAEpaeTARAAPEI-5, Unigene17914\_TAEpaeTARAAPEI-5, Unigene17967\_TAEpaeTARAAPEI-5, Unigene18038\_TAEpaeTARAAPEI-5, Unigene18070\_TAEpaeTARAAPEI-5, Unigene18700\_TAEpaeTARAAPEI-5, Unigene19545\_TAEpaeTARAAPEI-5, Unigene20587\_TAEpaeTARAAPEI-5, Unigene21151\_TAEpaeTARAAPEI-5, Unigene2140\_TAEpaeTARAAPEI-5, Unigene21423\_TAEpaeTARAAPEI-5, Unigene21976\_TAEpaeTARAAPEI-5, Unigene22121\_TAEpaeTARAAPEI-5, Unigene22868\_TAEpaeTARAAPEI-5, Unigene23452\_TAEpaeTARAAPEI-5, Unigene24119\_TAEpaeTARAAPEI-5, Unigene24536\_TAEpaeTARAAPEI-5, Unigene2459\_TAEpaeTARAAPEI-5, Unigene25231\_TAEpaeTARAAPEI-5, Unigene25402\_TAEpaeTARAAPEI-5, Unigene25405\_TAEpaeTARAAPEI-5, Unigene26005\_TAEpaeTARAAPEI-5, Unigene26233\_TAEpaeTARAAPEI-5, Unigene26484\_TAEpaeTARAAPEI-5, Unigene27077\_TAEpaeTARAAPEI-5, Unigene27310\_TAEpaeTARAAPEI-5, Unigene27449\_TAEpaeTARAAPEI-5, Unigene27470\_TAEpaeTARAAPEI-5, Unigene27477\_TAEpaeTARAAPEI-5, Unigene28503\_TAEpaeTARAAPEI-5, Unigene29077\_TAEpaeTARAAPEI-5, Unigene29946\_TAEpaeTARAAPEI-5, Unigene30271\_TAEpaeTARAAPEI-5, Unigene30332\_TAEpaeTARAAPEI-5, Unigene3109\_TAEpaeTARAAPEI-5, Unigene31348\_TAEpaeTARAAPEI-5, Unigene3159\_TAEpaeTARAAPEI-5, Unigene31854\_TAEpaeTARAAPEI-5, Unigene31866\_TAEpaeTARAAPEI-5, Unigene31957\_TAEpaeTARAAPEI-5, Unigene32463\_TAEpaeTARAAPEI-5, Unigene33015\_TAEpaeTARAAPEI-5, Unigene33029\_TAEpaeTARAAPEI-5, Unigene33153\_TAEpaeTARAAPEI-5, Unigene33479\_TAEpaeTARAAPEI-5, Unigene33627\_TAEpaeTARAAPEI-5, Unigene3453\_TAEpaeTARAAPEI-5, Unigene34731\_TAEpaeTARAAPEI-5, Unigene35066\_TAEpaeTARAAPEI-5, Unigene35147\_TAEpaeTARAAPEI-5, Unigene35524\_TAEpaeTARAAPEI-5, Unigene3558\_TAEpaeTARAAPEI-5, Unigene35860\_TAEpaeTARAAPEI-5, Unigene3621\_TAEpaeTARAAPEI-5, Unigene36475\_TAEpaeTARAAPEI-5, Unigene370\_TAEpaeTARAAPEI-5, Unigene37140\_TAEpaeTARAAPEI-5, Unigene37725\_TAEpaeTARAAPEI-5, Unigene37833\_TAEpaeTARAAPEI-5, Unigene38035\_TAEpaeTARAAPEI-5, Unigene38063\_TAEpaeTARAAPEI-5, Unigene38133\_TAEpaeTARAAPEI-5, Unigene39079\_TAEpaeTARAAPEI-5, Unigene39087\_TAEpaeTARAAPEI-5, Unigene39259\_TAEpaeTARAAPEI-5, Unigene39272\_TAEpaeTARAAPEI-5, Unigene39287\_TAEpaeTARAAPEI-5, Unigene39469\_TAEpaeTARAAPEI-5, Unigene40180\_TAEpaeTARAAPEI-5, Unigene40363\_TAEpaeTARAAPEI-5, Unigene40370\_TAEpaeTARAAPEI-5, Unigene40500\_TAEpaeTARAAPEI-5, Unigene40654\_TAEpaeTARAAPEI-5, Unigene41183\_TAEpaeTARAAPEI-5, Unigene41331\_TAEpaeTARAAPEI-5, Unigene41365\_TAEpaeTARAAPEI-5, Unigene4164\_TAEpaeTARAAPEI-5, Unigene41774\_TAEpaeTARAAPEI-5, Unigene41810\_TAEpaeTARAAPEI-5, Unigene42055\_TAEpaeTARAAPEI-5, Unigene42153\_TAEpaeTARAAPEI-5, Unigene42258\_TAEpaeTARAAPEI-5, Unigene4231\_TAEpaeTARAAPEI-5, Unigene42505\_TAEpaeTARAAPEI-5, Unigene42871\_TAEpaeTARAAPEI-5, Unigene43016\_TAEpaeTARAAPEI-5, Unigene43195\_TAEpaeTARAAPEI-5, Unigene43357\_TAEpaeTARAAPEI-5, Unigene43796\_TAEpaeTARAAPEI-5, Unigene43904\_TAEpaeTARAAPEI-5, Unigene44131\_TAEpaeTARAAPEI-5, Unigene4425\_TAEpaeTARAAPEI-5, Unigene44799\_TAEpaeTARAAPEI-5, Unigene44965\_TAEpaeTARAAPEI-5, Unigene45307\_TAEpaeTARAAPEI-5, Unigene45395\_TAEpaeTARAAPEI-5, Unigene45810\_TAEpaeTARAAPEI-5, Unigene45867\_TAEpaeTARAAPEI-5, Unigene46112\_TAEpaeTARAAPEI-5, Unigene46219\_TAEpaeTARAAPEI-5, Unigene46244\_TAEpaeTARAAPEI-5, Unigene46316\_TAEpaeTARAAPEI-5, Unigene46426\_TAEpaeTARAAPEI-5, Unigene47016\_TAEpaeTARAAPEI-5, Unigene47027\_TAEpaeTARAAPEI-5, Unigene47122\_TAEpaeTARAAPEI-5, Unigene47158\_TAEpaeTARAAPEI-5, Unigene47666\_TAEpaeTARAAPEI-5, Unigene47812\_TAEpaeTARAAPEI-5, Unigene47834\_TAEpaeTARAAPEI-5, Unigene47886\_TAEpaeTARAAPEI-5, Unigene47930\_TAEpaeTARAAPEI-5, Unigene48174\_TAEpaeTARAAPEI-5, Unigene48338\_TAEpaeTARAAPEI-5, Unigene48575\_TAEpaeTARAAPEI-5, Unigene48649\_TAEpaeTARAAPEI-5, Unigene49034\_TAEpaeTARAAPEI-5, Unigene49496\_TAEpaeTARAAPEI-5, Unigene49890\_TAEpaeTARAAPEI-5, Unigene50000\_TAEpaeTARAAPEI-5, Unigene50229\_TAEpaeTARAAPEI-5, Unigene50420\_TAEpaeTARAAPEI-5, Unigene50616\_TAEpaeTARAAPEI-5, Unigene50682\_TAEpaeTARAAPEI-5, Unigene51027\_TAEpaeTARAAPEI-5, Unigene5107\_TAEpaeTARAAPEI-5, Unigene51182\_TAEpaeTARAAPEI-5, Unigene51358\_TAEpaeTARAAPEI-5, Unigene51974\_TAEpaeTARAAPEI-5, Unigene51997\_TAEpaeTARAAPEI-5, Unigene52152\_TAEpaeTARAAPEI-5, Unigene52448\_TAEpaeTARAAPEI-5, Unigene52802\_TAEpaeTARAAPEI-5, Unigene53006\_TAEpaeTARAAPEI-5, Unigene53028\_TAEpaeTARAAPEI-5, Unigene53749\_TAEpaeTARAAPEI-5, Unigene53844\_TAEpaeTARAAPEI-5, Unigene53878\_TAEpaeTARAAPEI-5, Unigene53984\_TAEpaeTARAAPEI-5, Unigene5411\_TAEpaeTARAAPEI-5, Unigene54182\_TAEpaeTARAAPEI-5, Unigene54312\_TAEpaeTARAAPEI-5, Unigene54444\_TAEpaeTARAAPEI-5, Unigene54452\_TAEpaeTARAAPEI-5, Unigene54458\_TAEpaeTARAAPEI-5, Unigene54462\_TAEpaeTARAAPEI-5, Unigene54702\_TAEpaeTARAAPEI-5, Unigene54713\_TAEpaeTARAAPEI-5, Unigene55119\_TAEpaeTARAAPEI-5, Unigene55141\_TAEpaeTARAAPEI-5, Unigene55384\_TAEpaeTARAAPEI-5, Unigene55529\_TAEpaeTARAAPEI-5, Unigene55781\_TAEpaeTARAAPEI-5, Unigene56136\_TAEpaeTARAAPEI-5, Unigene56520\_TAEpaeTARAAPEI-5, Unigene56554\_TAEpaeTARAAPEI-5, Unigene56562\_TAEpaeTARAAPEI-5, Unigene56984\_TAEpaeTARAAPEI-5, Unigene57416\_TAEpaeTARAAPEI-5, Unigene57490\_TAEpaeTARAAPEI-5, Unigene57635\_TAEpaeTARAAPEI-5, Unigene57644\_TAEpaeTARAAPEI-5, Unigene58047\_TAEpaeTARAAPEI-5, Unigene58135\_TAEpaeTARAAPEI-5, Unigene58208\_TAEpaeTARAAPEI-5, Unigene58427\_TAEpaeTARAAPEI-5, Unigene58484\_TAEpaeTARAAPEI-5, Unigene58676\_TAEpaeTARAAPEI-5, Unigene58962\_TAEpaeTARAAPEI-5, Unigene59005\_TAEpaeTARAAPEI-5, Unigene59024\_TAEpaeTARAAPEI-5, Unigene59178\_TAEpaeTARAAPEI-5, Unigene59293\_TAEpaeTARAAPEI-5, Unigene5935\_TAEpaeTARAAPEI-5, Unigene59400\_TAEpaeTARAAPEI-5, Unigene59597\_TAEpaeTARAAPEI-5, Unigene59697\_TAEpaeTARAAPEI-5, Unigene59954\_TAEpaeTARAAPEI-5, Unigene60258\_TAEpaeTARAAPEI-5, Unigene60313\_TAEpaeTARAAPEI-5, Unigene60456\_TAEpaeTARAAPEI-5, Unigene60592\_TAEpaeTARAAPEI-5, Unigene60664\_TAEpaeTARAAPEI-5, Unigene60901\_TAEpaeTARAAPEI-5, Unigene60991\_TAEpaeTARAAPEI-5, Unigene6107\_TAEpaeTARAAPEI-5, Unigene61086\_TAEpaeTARAAPEI-5, Unigene61110\_TAEpaeTARAAPEI-5, Unigene61139\_TAEpaeTARAAPEI-5, Unigene61157\_TAEpaeTARAAPEI-5, Unigene61247\_TAEpaeTARAAPEI-5, Unigene61396\_TAEpaeTARAAPEI-5, Unigene61421\_TAEpaeTARAAPEI-5, Unigene61462\_TAEpaeTARAAPEI-5, Unigene61586\_TAEpaeTARAAPEI-5, Unigene61688\_TAEpaeTARAAPEI-5, Unigene61847\_TAEpaeTARAAPEI-5, Unigene61861\_TAEpaeTARAAPEI-5, Unigene61917\_TAEpaeTARAAPEI-5, Unigene62062\_TAEpaeTARAAPEI-5, Unigene62103\_TAEpaeTARAAPEI-5, Unigene62116\_TAEpaeTARAAPEI-5, Unigene62241\_TAEpaeTARAAPEI-5, Unigene62384\_TAEpaeTARAAPEI-5, Unigene62502\_TAEpaeTARAAPEI-5, Unigene62811\_TAEpaeTARAAPEI-5, Unigene63051\_TAEpaeTARAAPEI-5, Unigene63167\_TAEpaeTARAAPEI-5, Unigene63384\_TAEpaeTARAAPEI-5, Unigene63440\_TAEpaeTARAAPEI-5, Unigene63494\_TAEpaeTARAAPEI-5, Unigene63495\_TAEpaeTARAAPEI-5, Unigene63562\_TAEpaeTARAAPEI-5, Unigene63794\_TAEpaeTARAAPEI-5, Unigene64016\_TAEpaeTARAAPEI-5, Unigene64437\_TAEpaeTARAAPEI-5, Unigene64452\_TAEpaeTARAAPEI-5, Unigene64463\_TAEpaeTARAAPEI-5, Unigene64513\_TAEpaeTARAAPEI-5, Unigene64516\_TAEpaeTARAAPEI-5, Unigene64737\_TAEpaeTARAAPEI-5, Unigene65062\_TAEpaeTARAAPEI-5, Unigene65143\_TAEpaeTARAAPEI-5, Unigene65194\_TAEpaeTARAAPEI-5, Unigene65226\_TAEpaeTARAAPEI-5, Unigene65287\_TAEpaeTARAAPEI-5, Unigene65301\_TAEpaeTARAAPEI-5, Unigene65424\_TAEpaeTARAAPEI-5, Unigene6549\_TAEpaeTARAAPEI-5, Unigene65617\_TAEpaeTARAAPEI-5, Unigene65706\_TAEpaeTARAAPEI-5, Unigene65735\_TAEpaeTARAAPEI-5, Unigene65753\_TAEpaeTARAAPEI-5, Unigene65856\_TAEpaeTARAAPEI-5, Unigene65935\_TAEpaeTARAAPEI-5, Unigene66183\_TAEpaeTARAAPEI-5, Unigene66357\_TAEpaeTARAAPEI-5, Unigene66392\_TAEpaeTARAAPEI-5, Unigene66545\_TAEpaeTARAAPEI-5, Unigene66697\_TAEpaeTARAAPEI-5, Unigene67132\_TAEpaeTARAAPEI-5, Unigene67176\_TAEpaeTARAAPEI-5, Unigene67240\_TAEpaeTARAAPEI-5, Unigene67384\_TAEpaeTARAAPEI-5, Unigene67546\_TAEpaeTARAAPEI-5, Unigene67675\_TAEpaeTARAAPEI-5, Unigene67858\_TAEpaeTARAAPEI-5, Unigene6785\_TAEpaeTARAAPEI-5, Unigene67872\_TAEpaeTARAAPEI-5, Unigene67968\_TAEpaeTARAAPEI-5, Unigene68104\_TAEpaeTARAAPEI-5, Unigene68189\_TAEpaeTARAAPEI-5, Unigene68331\_TAEpaeTARAAPEI-5, Unigene68405\_TAEpaeTARAAPEI-5, Unigene68419\_TAEpaeTARAAPEI-5, Unigene68440\_TAEpaeTARAAPEI-5, Unigene68530\_TAEpaeTARAAPEI-5, Unigene68588\_TAEpaeTARAAPEI-5, Unigene68628\_TAEpaeTARAAPEI-5, Unigene68658\_TAEpaeTARAAPEI-5, Unigene68719\_TAEpaeTARAAPEI-5, Unigene68726\_TAEpaeTARAAPEI-5, Unigene69003\_TAEpaeTARAAPEI-5, Unigene69074\_TAEpaeTARAAPEI-5, Unigene69172\_TAEpaeTARAAPEI-5, Unigene69231\_TAEpaeTARAAPEI-5, Unigene69233\_TAEpaeTARAAPEI-5, Unigene69514\_TAEpaeTARAAPEI-5, Unigene69751\_TAEpaeTARAAPEI-5, Unigene7002\_TAEpaeTARAAPEI-5, Unigene70228\_TAEpaeTARAAPEI-5, Unigene70337\_TAEpaeTARAAPEI-5, Unigene70355\_TAEpaeTARAAPEI-5, Unigene70392\_TAEpaeTARAAPEI-5, Unigene70602\_TAEpaeTARAAPEI-5, Unigene70650\_TAEpaeTARAAPEI-5, Unigene70681\_TAEpaeTARAAPEI-5, Unigene70735\_TAEpaeTARAAPEI-5, Unigene7073\_TAEpaeTARAAPEI-5, Unigene70744\_TAEpaeTARAAPEI-5, Unigene70871\_TAEpaeTARAAPEI-5, Unigene71000\_TAEpaeTARAAPEI-5, Unigene71172\_TAEpaeTARAAPEI-5, Unigene71229\_TAEpaeTARAAPEI-5, Unigene71264\_TAEpaeTARAAPEI-5, Unigene71315\_TAEpaeTARAAPEI-5, Unigene71351\_TAEpaeTARAAPEI-5, Unigene71376\_TAEpaeTARAAPEI-5, Unigene71531\_TAEpaeTARAAPEI-5, Unigene71567\_TAEpaeTARAAPEI-5, Unigene71591\_TAEpaeTARAAPEI-5, Unigene71635\_TAEpaeTARAAPEI-5, Unigene71649\_TAEpaeTARAAPEI-5, Unigene71771\_TAEpaeTARAAPEI-5, Unigene71920\_TAEpaeTARAAPEI-5, Unigene72010\_TAEpaeTARAAPEI-5, Unigene72022\_TAEpaeTARAAPEI-5, Unigene72041\_TAEpaeTARAAPEI-5, Unigene72155\_TAEpaeTARAAPEI-5, Unigene72223\_TAEpaeTARAAPEI-5, Unigene72312\_TAEpaeTARAAPEI-5, Unigene72388\_TAEpaeTARAAPEI-5, Unigene72429\_TAEpaeTARAAPEI-5, Unigene7246\_TAEpaeTARAAPEI-5, Unigene72487\_TAEpaeTARAAPEI-5, Unigene72496\_TAEpaeTARAAPEI-5, Unigene72516\_TAEpaeTARAAPEI-5, Unigene72519\_TAEpaeTARAAPEI-5, Unigene72525\_TAEpaeTARAAPEI-5, Unigene72600\_TAEpaeTARAAPEI-5, Unigene72604\_TAEpaeTARAAPEI-5, Unigene72621\_TAEpaeTARAAPEI-5, Unigene72633\_TAEpaeTARAAPEI-5, Unigene72658\_TAEpaeTARAAPEI-5, Unigene72661\_TAEpaeTARAAPEI-5, Unigene72739\_TAEpaeTARAAPEI-5, Unigene72808\_TAEpaeTARAAPEI-5, Unigene72861\_TAEpaeTARAAPEI-5, Unigene72882\_TAEpaeTARAAPEI-5, Unigene72883\_TAEpaeTARAAPEI-5, Unigene72899\_TAEpaeTARAAPEI-5, Unigene72913\_TAEpaeTARAAPEI-5, Unigene72924\_TAEpaeTARAAPEI-5, Unigene72930\_TAEpaeTARAAPEI-5, Unigene72937\_TAEpaeTARAAPEI-5, Unigene7801\_TAEpaeTARAAPEI-5, Unigene8441\_TAEpaeTARAAPEI-5, Unigene8488\_TAEpaeTARAAPEI-5, Unigene8628\_TAEpaeTARAAPEI-5, Unigene8692\_TAEpaeTARAAPEI-5, Unigene8897\_TAEpaeTARAAPEI-5, Unigene9292\_TAEpaeTARAAPEI-5, Unigene9422\_TAEpaeTARAAPEI-5, Unigene976\_TAEpaeTARAAPEI-5, Unigene9785\_TAEpaeTARAAPEI-5, Unigene98\_TAEpaeTARAAPEI-5 |
| 16 | Alzheimer's disease | Unigene10137\_TAEpaeTARAAPEI-5, Unigene10260\_TAEpaeTARAAPEI-5, Unigene10302\_TAEpaeTARAAPEI-5, Unigene10428\_TAEpaeTARAAPEI-5, Unigene10475\_TAEpaeTARAAPEI-5, Unigene10838\_TAEpaeTARAAPEI-5, Unigene10871\_TAEpaeTARAAPEI-5, Unigene11355\_TAEpaeTARAAPEI-5, Unigene11397\_TAEpaeTARAAPEI-5, Unigene11418\_TAEpaeTARAAPEI-5, Unigene11775\_TAEpaeTARAAPEI-5, Unigene12151\_TAEpaeTARAAPEI-5, Unigene12236\_TAEpaeTARAAPEI-5, Unigene12247\_TAEpaeTARAAPEI-5, Unigene12387\_TAEpaeTARAAPEI-5, Unigene12538\_TAEpaeTARAAPEI-5, Unigene12600\_TAEpaeTARAAPEI-5, Unigene12956\_TAEpaeTARAAPEI-5, Unigene12998\_TAEpaeTARAAPEI-5, Unigene13162\_TAEpaeTARAAPEI-5, Unigene13546\_TAEpaeTARAAPEI-5, Unigene13609\_TAEpaeTARAAPEI-5, Unigene13656\_TAEpaeTARAAPEI-5, Unigene13681\_TAEpaeTARAAPEI-5, Unigene13747\_TAEpaeTARAAPEI-5, Unigene13812\_TAEpaeTARAAPEI-5, Unigene13829\_TAEpaeTARAAPEI-5, Unigene13856\_TAEpaeTARAAPEI-5, Unigene13913\_TAEpaeTARAAPEI-5, Unigene13956\_TAEpaeTARAAPEI-5, Unigene14095\_TAEpaeTARAAPEI-5, Unigene14105\_TAEpaeTARAAPEI-5, Unigene14408\_TAEpaeTARAAPEI-5, Unigene14793\_TAEpaeTARAAPEI-5, Unigene14847\_TAEpaeTARAAPEI-5, Unigene14925\_TAEpaeTARAAPEI-5, Unigene14928\_TAEpaeTARAAPEI-5, Unigene14\_TAEpaeTARAAPEI-5, Unigene15078\_TAEpaeTARAAPEI-5, Unigene15127\_TAEpaeTARAAPEI-5, Unigene15247\_TAEpaeTARAAPEI-5, Unigene15334\_TAEpaeTARAAPEI-5, Unigene15571\_TAEpaeTARAAPEI-5, Unigene15706\_TAEpaeTARAAPEI-5, Unigene15713\_TAEpaeTARAAPEI-5, Unigene15853\_TAEpaeTARAAPEI-5, Unigene16140\_TAEpaeTARAAPEI-5, Unigene1657\_TAEpaeTARAAPEI-5, Unigene16682\_TAEpaeTARAAPEI-5, Unigene16749\_TAEpaeTARAAPEI-5, Unigene167\_TAEpaeTARAAPEI-5, Unigene17071\_TAEpaeTARAAPEI-5, Unigene1710\_TAEpaeTARAAPEI-5, Unigene17265\_TAEpaeTARAAPEI-5, Unigene17277\_TAEpaeTARAAPEI-5, Unigene17301\_TAEpaeTARAAPEI-5, Unigene17326\_TAEpaeTARAAPEI-5, Unigene17529\_TAEpaeTARAAPEI-5, Unigene17558\_TAEpaeTARAAPEI-5, Unigene17678\_TAEpaeTARAAPEI-5, Unigene18006\_TAEpaeTARAAPEI-5, Unigene18014\_TAEpaeTARAAPEI-5, Unigene1839\_TAEpaeTARAAPEI-5, Unigene1953\_TAEpaeTARAAPEI-5, Unigene19640\_TAEpaeTARAAPEI-5, Unigene20328\_TAEpaeTARAAPEI-5, Unigene20503\_TAEpaeTARAAPEI-5, Unigene20996\_TAEpaeTARAAPEI-5, Unigene2109\_TAEpaeTARAAPEI-5, Unigene21299\_TAEpaeTARAAPEI-5, Unigene21363\_TAEpaeTARAAPEI-5, Unigene21445\_TAEpaeTARAAPEI-5, Unigene21511\_TAEpaeTARAAPEI-5, Unigene22021\_TAEpaeTARAAPEI-5, Unigene22085\_TAEpaeTARAAPEI-5, Unigene22196\_TAEpaeTARAAPEI-5, Unigene22499\_TAEpaeTARAAPEI-5, Unigene22557\_TAEpaeTARAAPEI-5, Unigene22658\_TAEpaeTARAAPEI-5, Unigene22944\_TAEpaeTARAAPEI-5, Unigene23884\_TAEpaeTARAAPEI-5, Unigene24007\_TAEpaeTARAAPEI-5, Unigene24220\_TAEpaeTARAAPEI-5, Unigene24330\_TAEpaeTARAAPEI-5, Unigene24831\_TAEpaeTARAAPEI-5, Unigene25288\_TAEpaeTARAAPEI-5, Unigene25611\_TAEpaeTARAAPEI-5, Unigene26004\_TAEpaeTARAAPEI-5, Unigene26124\_TAEpaeTARAAPEI-5, Unigene26135\_TAEpaeTARAAPEI-5, Unigene26199\_TAEpaeTARAAPEI-5, Unigene26231\_TAEpaeTARAAPEI-5, Unigene26662\_TAEpaeTARAAPEI-5, Unigene26939\_TAEpaeTARAAPEI-5, Unigene27084\_TAEpaeTARAAPEI-5, Unigene27376\_TAEpaeTARAAPEI-5, Unigene27593\_TAEpaeTARAAPEI-5, Unigene27629\_TAEpaeTARAAPEI-5, Unigene277\_TAEpaeTARAAPEI-5, Unigene27874\_TAEpaeTARAAPEI-5, Unigene2852\_TAEpaeTARAAPEI-5, Unigene28660\_TAEpaeTARAAPEI-5, Unigene28988\_TAEpaeTARAAPEI-5, Unigene29008\_TAEpaeTARAAPEI-5, Unigene29119\_TAEpaeTARAAPEI-5, Unigene29132\_TAEpaeTARAAPEI-5, Unigene29157\_TAEpaeTARAAPEI-5, Unigene29193\_TAEpaeTARAAPEI-5, Unigene29381\_TAEpaeTARAAPEI-5, Unigene293\_TAEpaeTARAAPEI-5, Unigene29460\_TAEpaeTARAAPEI-5, Unigene30015\_TAEpaeTARAAPEI-5, Unigene30101\_TAEpaeTARAAPEI-5, Unigene30135\_TAEpaeTARAAPEI-5, Unigene30744\_TAEpaeTARAAPEI-5, Unigene31344\_TAEpaeTARAAPEI-5, Unigene31619\_TAEpaeTARAAPEI-5, Unigene31709\_TAEpaeTARAAPEI-5, Unigene32338\_TAEpaeTARAAPEI-5, Unigene32425\_TAEpaeTARAAPEI-5, Unigene32504\_TAEpaeTARAAPEI-5, Unigene32899\_TAEpaeTARAAPEI-5, Unigene32934\_TAEpaeTARAAPEI-5, Unigene33549\_TAEpaeTARAAPEI-5, Unigene33932\_TAEpaeTARAAPEI-5, Unigene34000\_TAEpaeTARAAPEI-5, Unigene34024\_TAEpaeTARAAPEI-5, Unigene34043\_TAEpaeTARAAPEI-5, Unigene34285\_TAEpaeTARAAPEI-5, Unigene34374\_TAEpaeTARAAPEI-5, Unigene34543\_TAEpaeTARAAPEI-5, Unigene34593\_TAEpaeTARAAPEI-5, Unigene35153\_TAEpaeTARAAPEI-5, Unigene35256\_TAEpaeTARAAPEI-5, Unigene35733\_TAEpaeTARAAPEI-5, Unigene35956\_TAEpaeTARAAPEI-5, Unigene36803\_TAEpaeTARAAPEI-5, Unigene37059\_TAEpaeTARAAPEI-5, Unigene37156\_TAEpaeTARAAPEI-5, Unigene37318\_TAEpaeTARAAPEI-5, Unigene37367\_TAEpaeTARAAPEI-5, Unigene37487\_TAEpaeTARAAPEI-5, Unigene37671\_TAEpaeTARAAPEI-5, Unigene37716\_TAEpaeTARAAPEI-5, Unigene37729\_TAEpaeTARAAPEI-5, Unigene37869\_TAEpaeTARAAPEI-5, Unigene38325\_TAEpaeTARAAPEI-5, Unigene38618\_TAEpaeTARAAPEI-5, Unigene38716\_TAEpaeTARAAPEI-5, Unigene39041\_TAEpaeTARAAPEI-5, Unigene39301\_TAEpaeTARAAPEI-5, Unigene39396\_TAEpaeTARAAPEI-5, Unigene39522\_TAEpaeTARAAPEI-5, Unigene39680\_TAEpaeTARAAPEI-5, Unigene39855\_TAEpaeTARAAPEI-5, Unigene39867\_TAEpaeTARAAPEI-5, Unigene39976\_TAEpaeTARAAPEI-5, Unigene40806\_TAEpaeTARAAPEI-5, Unigene41084\_TAEpaeTARAAPEI-5, Unigene41388\_TAEpaeTARAAPEI-5, Unigene41451\_TAEpaeTARAAPEI-5, Unigene41541\_TAEpaeTARAAPEI-5, Unigene41593\_TAEpaeTARAAPEI-5, Unigene42193\_TAEpaeTARAAPEI-5, Unigene42360\_TAEpaeTARAAPEI-5, Unigene4275\_TAEpaeTARAAPEI-5, Unigene43099\_TAEpaeTARAAPEI-5, Unigene43373\_TAEpaeTARAAPEI-5, Unigene43764\_TAEpaeTARAAPEI-5, Unigene43795\_TAEpaeTARAAPEI-5, Unigene43927\_TAEpaeTARAAPEI-5, Unigene44056\_TAEpaeTARAAPEI-5, Unigene44522\_TAEpaeTARAAPEI-5, Unigene44777\_TAEpaeTARAAPEI-5, Unigene44905\_TAEpaeTARAAPEI-5, Unigene44928\_TAEpaeTARAAPEI-5, Unigene45338\_TAEpaeTARAAPEI-5, Unigene45493\_TAEpaeTARAAPEI-5, Unigene45577\_TAEpaeTARAAPEI-5, Unigene45636\_TAEpaeTARAAPEI-5, Unigene46319\_TAEpaeTARAAPEI-5, Unigene46530\_TAEpaeTARAAPEI-5, Unigene46564\_TAEpaeTARAAPEI-5, Unigene46779\_TAEpaeTARAAPEI-5, Unigene46962\_TAEpaeTARAAPEI-5, Unigene47512\_TAEpaeTARAAPEI-5, Unigene48176\_TAEpaeTARAAPEI-5, Unigene4845\_TAEpaeTARAAPEI-5, Unigene48711\_TAEpaeTARAAPEI-5, Unigene48734\_TAEpaeTARAAPEI-5, Unigene48744\_TAEpaeTARAAPEI-5, Unigene48845\_TAEpaeTARAAPEI-5, Unigene48850\_TAEpaeTARAAPEI-5, Unigene49141\_TAEpaeTARAAPEI-5, Unigene49327\_TAEpaeTARAAPEI-5, Unigene49657\_TAEpaeTARAAPEI-5, Unigene49761\_TAEpaeTARAAPEI-5, Unigene49990\_TAEpaeTARAAPEI-5, Unigene50088\_TAEpaeTARAAPEI-5, Unigene50104\_TAEpaeTARAAPEI-5, Unigene50473\_TAEpaeTARAAPEI-5, Unigene50742\_TAEpaeTARAAPEI-5, Unigene50753\_TAEpaeTARAAPEI-5, Unigene50986\_TAEpaeTARAAPEI-5, Unigene51029\_TAEpaeTARAAPEI-5, Unigene51223\_TAEpaeTARAAPEI-5, Unigene5143\_TAEpaeTARAAPEI-5, Unigene51561\_TAEpaeTARAAPEI-5, Unigene51659\_TAEpaeTARAAPEI-5, Unigene51772\_TAEpaeTARAAPEI-5, Unigene51977\_TAEpaeTARAAPEI-5, Unigene52012\_TAEpaeTARAAPEI-5, Unigene52036\_TAEpaeTARAAPEI-5, Unigene52429\_TAEpaeTARAAPEI-5, Unigene52548\_TAEpaeTARAAPEI-5, Unigene52566\_TAEpaeTARAAPEI-5, Unigene52781\_TAEpaeTARAAPEI-5, Unigene5279\_TAEpaeTARAAPEI-5, Unigene53064\_TAEpaeTARAAPEI-5, Unigene53161\_TAEpaeTARAAPEI-5, Unigene53182\_TAEpaeTARAAPEI-5, Unigene53512\_TAEpaeTARAAPEI-5, Unigene53530\_TAEpaeTARAAPEI-5, Unigene53825\_TAEpaeTARAAPEI-5, Unigene53\_TAEpaeTARAAPEI-5, Unigene54017\_TAEpaeTARAAPEI-5, Unigene54035\_TAEpaeTARAAPEI-5, Unigene54156\_TAEpaeTARAAPEI-5, Unigene54427\_TAEpaeTARAAPEI-5, Unigene54593\_TAEpaeTARAAPEI-5, Unigene54783\_TAEpaeTARAAPEI-5, Unigene54792\_TAEpaeTARAAPEI-5, Unigene55000\_TAEpaeTARAAPEI-5, Unigene55037\_TAEpaeTARAAPEI-5, Unigene55249\_TAEpaeTARAAPEI-5, Unigene55389\_TAEpaeTARAAPEI-5, Unigene55419\_TAEpaeTARAAPEI-5, Unigene55554\_TAEpaeTARAAPEI-5, Unigene55998\_TAEpaeTARAAPEI-5, Unigene559\_TAEpaeTARAAPEI-5, Unigene56010\_TAEpaeTARAAPEI-5, Unigene56014\_TAEpaeTARAAPEI-5, Unigene56039\_TAEpaeTARAAPEI-5, Unigene56793\_TAEpaeTARAAPEI-5, Unigene57446\_TAEpaeTARAAPEI-5, Unigene57736\_TAEpaeTARAAPEI-5, Unigene57938\_TAEpaeTARAAPEI-5, Unigene57946\_TAEpaeTARAAPEI-5, Unigene58164\_TAEpaeTARAAPEI-5, Unigene5848\_TAEpaeTARAAPEI-5, Unigene58508\_TAEpaeTARAAPEI-5, Unigene58533\_TAEpaeTARAAPEI-5, Unigene58568\_TAEpaeTARAAPEI-5, Unigene58651\_TAEpaeTARAAPEI-5, Unigene58763\_TAEpaeTARAAPEI-5, Unigene58850\_TAEpaeTARAAPEI-5, Unigene58941\_TAEpaeTARAAPEI-5, Unigene59429\_TAEpaeTARAAPEI-5, Unigene59590\_TAEpaeTARAAPEI-5, Unigene59686\_TAEpaeTARAAPEI-5, Unigene59807\_TAEpaeTARAAPEI-5, Unigene60103\_TAEpaeTARAAPEI-5, Unigene60115\_TAEpaeTARAAPEI-5, Unigene60468\_TAEpaeTARAAPEI-5, Unigene60579\_TAEpaeTARAAPEI-5, Unigene60622\_TAEpaeTARAAPEI-5, Unigene60755\_TAEpaeTARAAPEI-5, Unigene60925\_TAEpaeTARAAPEI-5, Unigene60946\_TAEpaeTARAAPEI-5, Unigene61268\_TAEpaeTARAAPEI-5, Unigene61489\_TAEpaeTARAAPEI-5, Unigene61621\_TAEpaeTARAAPEI-5, Unigene61672\_TAEpaeTARAAPEI-5, Unigene61674\_TAEpaeTARAAPEI-5, Unigene61680\_TAEpaeTARAAPEI-5, Unigene61892\_TAEpaeTARAAPEI-5, Unigene61894\_TAEpaeTARAAPEI-5, Unigene62295\_TAEpaeTARAAPEI-5, Unigene62463\_TAEpaeTARAAPEI-5, Unigene62549\_TAEpaeTARAAPEI-5, Unigene62702\_TAEpaeTARAAPEI-5, Unigene62789\_TAEpaeTARAAPEI-5, Unigene62816\_TAEpaeTARAAPEI-5, Unigene6294\_TAEpaeTARAAPEI-5, Unigene62975\_TAEpaeTARAAPEI-5, Unigene62989\_TAEpaeTARAAPEI-5, Unigene63328\_TAEpaeTARAAPEI-5, Unigene63387\_TAEpaeTARAAPEI-5, Unigene63607\_TAEpaeTARAAPEI-5, Unigene64009\_TAEpaeTARAAPEI-5, Unigene64039\_TAEpaeTARAAPEI-5, Unigene64126\_TAEpaeTARAAPEI-5, Unigene64297\_TAEpaeTARAAPEI-5, Unigene64423\_TAEpaeTARAAPEI-5, Unigene64449\_TAEpaeTARAAPEI-5, Unigene64467\_TAEpaeTARAAPEI-5, Unigene64782\_TAEpaeTARAAPEI-5, Unigene64811\_TAEpaeTARAAPEI-5, Unigene64852\_TAEpaeTARAAPEI-5, Unigene64865\_TAEpaeTARAAPEI-5, Unigene64995\_TAEpaeTARAAPEI-5, Unigene65233\_TAEpaeTARAAPEI-5, Unigene65292\_TAEpaeTARAAPEI-5, Unigene65343\_TAEpaeTARAAPEI-5, Unigene65501\_TAEpaeTARAAPEI-5, Unigene65670\_TAEpaeTARAAPEI-5, Unigene65757\_TAEpaeTARAAPEI-5, Unigene66161\_TAEpaeTARAAPEI-5, Unigene66398\_TAEpaeTARAAPEI-5, Unigene66427\_TAEpaeTARAAPEI-5, Unigene66490\_TAEpaeTARAAPEI-5, Unigene66562\_TAEpaeTARAAPEI-5, Unigene66625\_TAEpaeTARAAPEI-5, Unigene66858\_TAEpaeTARAAPEI-5, Unigene67068\_TAEpaeTARAAPEI-5, Unigene67126\_TAEpaeTARAAPEI-5, Unigene67459\_TAEpaeTARAAPEI-5, Unigene67480\_TAEpaeTARAAPEI-5, Unigene67550\_TAEpaeTARAAPEI-5, Unigene67597\_TAEpaeTARAAPEI-5, Unigene67861\_TAEpaeTARAAPEI-5, Unigene68029\_TAEpaeTARAAPEI-5, Unigene68033\_TAEpaeTARAAPEI-5, Unigene68139\_TAEpaeTARAAPEI-5, Unigene68156\_TAEpaeTARAAPEI-5, Unigene68217\_TAEpaeTARAAPEI-5, Unigene68468\_TAEpaeTARAAPEI-5, Unigene68565\_TAEpaeTARAAPEI-5, Unigene68878\_TAEpaeTARAAPEI-5, Unigene69257\_TAEpaeTARAAPEI-5, Unigene69285\_TAEpaeTARAAPEI-5, Unigene69403\_TAEpaeTARAAPEI-5, Unigene69517\_TAEpaeTARAAPEI-5, Unigene69607\_TAEpaeTARAAPEI-5, Unigene69681\_TAEpaeTARAAPEI-5, Unigene69688\_TAEpaeTARAAPEI-5, Unigene69792\_TAEpaeTARAAPEI-5, Unigene69914\_TAEpaeTARAAPEI-5, Unigene70135\_TAEpaeTARAAPEI-5, Unigene70224\_TAEpaeTARAAPEI-5, Unigene70521\_TAEpaeTARAAPEI-5, Unigene70561\_TAEpaeTARAAPEI-5, Unigene7057\_TAEpaeTARAAPEI-5, Unigene70657\_TAEpaeTARAAPEI-5, Unigene70792\_TAEpaeTARAAPEI-5, Unigene70930\_TAEpaeTARAAPEI-5, Unigene71180\_TAEpaeTARAAPEI-5, Unigene71230\_TAEpaeTARAAPEI-5, Unigene71368\_TAEpaeTARAAPEI-5, Unigene71440\_TAEpaeTARAAPEI-5, Unigene71461\_TAEpaeTARAAPEI-5, Unigene71473\_TAEpaeTARAAPEI-5, Unigene71482\_TAEpaeTARAAPEI-5, Unigene71587\_TAEpaeTARAAPEI-5, Unigene71672\_TAEpaeTARAAPEI-5, Unigene71682\_TAEpaeTARAAPEI-5, Unigene71700\_TAEpaeTARAAPEI-5, Unigene71797\_TAEpaeTARAAPEI-5, Unigene71803\_TAEpaeTARAAPEI-5, Unigene71841\_TAEpaeTARAAPEI-5, Unigene72083\_TAEpaeTARAAPEI-5, Unigene7237\_TAEpaeTARAAPEI-5, Unigene72399\_TAEpaeTARAAPEI-5, Unigene72458\_TAEpaeTARAAPEI-5, Unigene72472\_TAEpaeTARAAPEI-5, Unigene72520\_TAEpaeTARAAPEI-5, Unigene72573\_TAEpaeTARAAPEI-5, Unigene72662\_TAEpaeTARAAPEI-5, Unigene72691\_TAEpaeTARAAPEI-5, Unigene72696\_TAEpaeTARAAPEI-5, Unigene72758\_TAEpaeTARAAPEI-5, Unigene7463\_TAEpaeTARAAPEI-5, Unigene7781\_TAEpaeTARAAPEI-5, Unigene8049\_TAEpaeTARAAPEI-5, Unigene8070\_TAEpaeTARAAPEI-5, Unigene8206\_TAEpaeTARAAPEI-5, Unigene8411\_TAEpaeTARAAPEI-5, Unigene858\_TAEpaeTARAAPEI-5, Unigene8862\_TAEpaeTARAAPEI-5, Unigene8878\_TAEpaeTARAAPEI-5, Unigene9365\_TAEpaeTARAAPEI-5, Unigene9701\_TAEpaeTARAAPEI-5, Unigene9738\_TAEpaeTARAAPEI-5, Unigene9922\_TAEpaeTARAAPEI-5, Unigene9941\_TAEpaeTARAAPEI-5 |
| 17 | Wnt signaling pathway | Unigene10085\_TAEpaeTARAAPEI-5, Unigene10156\_TAEpaeTARAAPEI-5, Unigene10325\_TAEpaeTARAAPEI-5, Unigene10474\_TAEpaeTARAAPEI-5, Unigene10475\_TAEpaeTARAAPEI-5, Unigene10547\_TAEpaeTARAAPEI-5, Unigene11257\_TAEpaeTARAAPEI-5, Unigene1129\_TAEpaeTARAAPEI-5, Unigene11355\_TAEpaeTARAAPEI-5, Unigene11397\_TAEpaeTARAAPEI-5, Unigene11417\_TAEpaeTARAAPEI-5, Unigene11418\_TAEpaeTARAAPEI-5, Unigene11507\_TAEpaeTARAAPEI-5, Unigene12492\_TAEpaeTARAAPEI-5, Unigene12620\_TAEpaeTARAAPEI-5, Unigene12888\_TAEpaeTARAAPEI-5, Unigene12965\_TAEpaeTARAAPEI-5, Unigene13055\_TAEpaeTARAAPEI-5, Unigene13082\_TAEpaeTARAAPEI-5, Unigene13231\_TAEpaeTARAAPEI-5, Unigene13251\_TAEpaeTARAAPEI-5, Unigene13352\_TAEpaeTARAAPEI-5, Unigene13439\_TAEpaeTARAAPEI-5, Unigene13473\_TAEpaeTARAAPEI-5, Unigene13629\_TAEpaeTARAAPEI-5, Unigene13681\_TAEpaeTARAAPEI-5, Unigene14027\_TAEpaeTARAAPEI-5, Unigene14092\_TAEpaeTARAAPEI-5, Unigene14178\_TAEpaeTARAAPEI-5, Unigene14301\_TAEpaeTARAAPEI-5, Unigene14333\_TAEpaeTARAAPEI-5, Unigene14408\_TAEpaeTARAAPEI-5, Unigene14809\_TAEpaeTARAAPEI-5, Unigene14945\_TAEpaeTARAAPEI-5, Unigene15060\_TAEpaeTARAAPEI-5, Unigene15450\_TAEpaeTARAAPEI-5, Unigene15750\_TAEpaeTARAAPEI-5, Unigene1611\_TAEpaeTARAAPEI-5, Unigene16371\_TAEpaeTARAAPEI-5, Unigene16468\_TAEpaeTARAAPEI-5, Unigene16483\_TAEpaeTARAAPEI-5, Unigene16610\_TAEpaeTARAAPEI-5, Unigene16815\_TAEpaeTARAAPEI-5, Unigene16817\_TAEpaeTARAAPEI-5, Unigene17145\_TAEpaeTARAAPEI-5, Unigene17167\_TAEpaeTARAAPEI-5, Unigene17220\_TAEpaeTARAAPEI-5, Unigene17397\_TAEpaeTARAAPEI-5, Unigene17673\_TAEpaeTARAAPEI-5, Unigene17700\_TAEpaeTARAAPEI-5, Unigene17706\_TAEpaeTARAAPEI-5, Unigene17970\_TAEpaeTARAAPEI-5, Unigene18102\_TAEpaeTARAAPEI-5, Unigene18420\_TAEpaeTARAAPEI-5, Unigene18551\_TAEpaeTARAAPEI-5, Unigene19903\_TAEpaeTARAAPEI-5, Unigene19937\_TAEpaeTARAAPEI-5, Unigene19985\_TAEpaeTARAAPEI-5, Unigene20403\_TAEpaeTARAAPEI-5, Unigene20934\_TAEpaeTARAAPEI-5, Unigene20955\_TAEpaeTARAAPEI-5, Unigene21356\_TAEpaeTARAAPEI-5, Unigene21764\_TAEpaeTARAAPEI-5, Unigene22459\_TAEpaeTARAAPEI-5, Unigene2291\_TAEpaeTARAAPEI-5, Unigene23040\_TAEpaeTARAAPEI-5, Unigene23237\_TAEpaeTARAAPEI-5, Unigene23338\_TAEpaeTARAAPEI-5, Unigene24012\_TAEpaeTARAAPEI-5, Unigene2429\_TAEpaeTARAAPEI-5, Unigene24488\_TAEpaeTARAAPEI-5, Unigene25611\_TAEpaeTARAAPEI-5, Unigene25933\_TAEpaeTARAAPEI-5, Unigene26365\_TAEpaeTARAAPEI-5, Unigene26458\_TAEpaeTARAAPEI-5, Unigene27310\_TAEpaeTARAAPEI-5, Unigene27376\_TAEpaeTARAAPEI-5, Unigene27470\_TAEpaeTARAAPEI-5, Unigene27611\_TAEpaeTARAAPEI-5, Unigene27682\_TAEpaeTARAAPEI-5, Unigene27929\_TAEpaeTARAAPEI-5, Unigene29946\_TAEpaeTARAAPEI-5, Unigene30199\_TAEpaeTARAAPEI-5, Unigene30655\_TAEpaeTARAAPEI-5, Unigene31474\_TAEpaeTARAAPEI-5, Unigene32257\_TAEpaeTARAAPEI-5, Unigene32381\_TAEpaeTARAAPEI-5, Unigene3258\_TAEpaeTARAAPEI-5, Unigene33549\_TAEpaeTARAAPEI-5, Unigene33932\_TAEpaeTARAAPEI-5, Unigene34047\_TAEpaeTARAAPEI-5, Unigene3419\_TAEpaeTARAAPEI-5, Unigene34267\_TAEpaeTARAAPEI-5, Unigene34344\_TAEpaeTARAAPEI-5, Unigene34523\_TAEpaeTARAAPEI-5, Unigene34593\_TAEpaeTARAAPEI-5, Unigene34847\_TAEpaeTARAAPEI-5, Unigene35250\_TAEpaeTARAAPEI-5, Unigene35256\_TAEpaeTARAAPEI-5, Unigene35498\_TAEpaeTARAAPEI-5, Unigene35509\_TAEpaeTARAAPEI-5, Unigene35785\_TAEpaeTARAAPEI-5, Unigene35956\_TAEpaeTARAAPEI-5, Unigene36580\_TAEpaeTARAAPEI-5, Unigene36984\_TAEpaeTARAAPEI-5, Unigene37059\_TAEpaeTARAAPEI-5, Unigene37209\_TAEpaeTARAAPEI-5, Unigene37247\_TAEpaeTARAAPEI-5, Unigene37318\_TAEpaeTARAAPEI-5, Unigene37328\_TAEpaeTARAAPEI-5, Unigene37376\_TAEpaeTARAAPEI-5, Unigene37692\_TAEpaeTARAAPEI-5, Unigene37869\_TAEpaeTARAAPEI-5, Unigene37937\_TAEpaeTARAAPEI-5, Unigene37980\_TAEpaeTARAAPEI-5, Unigene38035\_TAEpaeTARAAPEI-5, Unigene38063\_TAEpaeTARAAPEI-5, Unigene38165\_TAEpaeTARAAPEI-5, Unigene38182\_TAEpaeTARAAPEI-5, Unigene38485\_TAEpaeTARAAPEI-5, Unigene39051\_TAEpaeTARAAPEI-5, Unigene39079\_TAEpaeTARAAPEI-5, Unigene39087\_TAEpaeTARAAPEI-5, Unigene39396\_TAEpaeTARAAPEI-5, Unigene39983\_TAEpaeTARAAPEI-5, Unigene40596\_TAEpaeTARAAPEI-5, Unigene40826\_TAEpaeTARAAPEI-5, Unigene41451\_TAEpaeTARAAPEI-5, Unigene41541\_TAEpaeTARAAPEI-5, Unigene41673\_TAEpaeTARAAPEI-5, Unigene41799\_TAEpaeTARAAPEI-5, Unigene42012\_TAEpaeTARAAPEI-5, Unigene42098\_TAEpaeTARAAPEI-5, Unigene42766\_TAEpaeTARAAPEI-5, Unigene43076\_TAEpaeTARAAPEI-5, Unigene43078\_TAEpaeTARAAPEI-5, Unigene43135\_TAEpaeTARAAPEI-5, Unigene43198\_TAEpaeTARAAPEI-5, Unigene43328\_TAEpaeTARAAPEI-5, Unigene4371\_TAEpaeTARAAPEI-5, Unigene4380\_TAEpaeTARAAPEI-5, Unigene4384\_TAEpaeTARAAPEI-5, Unigene43942\_TAEpaeTARAAPEI-5, Unigene44202\_TAEpaeTARAAPEI-5, Unigene4425\_TAEpaeTARAAPEI-5, Unigene44401\_TAEpaeTARAAPEI-5, Unigene45239\_TAEpaeTARAAPEI-5, Unigene45401\_TAEpaeTARAAPEI-5, Unigene45468\_TAEpaeTARAAPEI-5, Unigene45712\_TAEpaeTARAAPEI-5, Unigene45803\_TAEpaeTARAAPEI-5, Unigene46224\_TAEpaeTARAAPEI-5, Unigene46452\_TAEpaeTARAAPEI-5, Unigene46564\_TAEpaeTARAAPEI-5, Unigene46657\_TAEpaeTARAAPEI-5, Unigene46788\_TAEpaeTARAAPEI-5, Unigene46844\_TAEpaeTARAAPEI-5, Unigene46966\_TAEpaeTARAAPEI-5, Unigene47023\_TAEpaeTARAAPEI-5, Unigene47218\_TAEpaeTARAAPEI-5, Unigene47512\_TAEpaeTARAAPEI-5, Unigene47595\_TAEpaeTARAAPEI-5, Unigene47834\_TAEpaeTARAAPEI-5, Unigene47930\_TAEpaeTARAAPEI-5, Unigene47945\_TAEpaeTARAAPEI-5, Unigene48018\_TAEpaeTARAAPEI-5, Unigene48394\_TAEpaeTARAAPEI-5, Unigene48503\_TAEpaeTARAAPEI-5, Unigene48575\_TAEpaeTARAAPEI-5, Unigene48725\_TAEpaeTARAAPEI-5, Unigene49135\_TAEpaeTARAAPEI-5, Unigene4974\_TAEpaeTARAAPEI-5, Unigene49761\_TAEpaeTARAAPEI-5, Unigene49887\_TAEpaeTARAAPEI-5, Unigene50174\_TAEpaeTARAAPEI-5, Unigene50566\_TAEpaeTARAAPEI-5, Unigene50651\_TAEpaeTARAAPEI-5, Unigene51072\_TAEpaeTARAAPEI-5, Unigene51435\_TAEpaeTARAAPEI-5, Unigene51640\_TAEpaeTARAAPEI-5, Unigene5194\_TAEpaeTARAAPEI-5, Unigene51987\_TAEpaeTARAAPEI-5, Unigene52304\_TAEpaeTARAAPEI-5, Unigene52429\_TAEpaeTARAAPEI-5, Unigene52452\_TAEpaeTARAAPEI-5, Unigene52648\_TAEpaeTARAAPEI-5, Unigene52814\_TAEpaeTARAAPEI-5, Unigene52835\_TAEpaeTARAAPEI-5, Unigene52847\_TAEpaeTARAAPEI-5, Unigene53106\_TAEpaeTARAAPEI-5, Unigene53399\_TAEpaeTARAAPEI-5, Unigene53446\_TAEpaeTARAAPEI-5, Unigene53530\_TAEpaeTARAAPEI-5, Unigene53786\_TAEpaeTARAAPEI-5, Unigene53942\_TAEpaeTARAAPEI-5, Unigene54063\_TAEpaeTARAAPEI-5, Unigene54206\_TAEpaeTARAAPEI-5, Unigene54544\_TAEpaeTARAAPEI-5, Unigene54588\_TAEpaeTARAAPEI-5, Unigene54655\_TAEpaeTARAAPEI-5, Unigene55027\_TAEpaeTARAAPEI-5, Unigene55063\_TAEpaeTARAAPEI-5, Unigene55234\_TAEpaeTARAAPEI-5, Unigene55494\_TAEpaeTARAAPEI-5, Unigene55529\_TAEpaeTARAAPEI-5, Unigene55533\_TAEpaeTARAAPEI-5, Unigene55689\_TAEpaeTARAAPEI-5, Unigene55799\_TAEpaeTARAAPEI-5, Unigene55873\_TAEpaeTARAAPEI-5, Unigene55954\_TAEpaeTARAAPEI-5, Unigene56014\_TAEpaeTARAAPEI-5, Unigene56308\_TAEpaeTARAAPEI-5, Unigene57135\_TAEpaeTARAAPEI-5, Unigene57235\_TAEpaeTARAAPEI-5, Unigene57371\_TAEpaeTARAAPEI-5, Unigene57392\_TAEpaeTARAAPEI-5, Unigene57397\_TAEpaeTARAAPEI-5, Unigene57471\_TAEpaeTARAAPEI-5, Unigene57542\_TAEpaeTARAAPEI-5, Unigene57806\_TAEpaeTARAAPEI-5, Unigene57935\_TAEpaeTARAAPEI-5, Unigene57969\_TAEpaeTARAAPEI-5, Unigene57974\_TAEpaeTARAAPEI-5, Unigene58427\_TAEpaeTARAAPEI-5, Unigene58484\_TAEpaeTARAAPEI-5, Unigene59211\_TAEpaeTARAAPEI-5, Unigene59282\_TAEpaeTARAAPEI-5, Unigene59293\_TAEpaeTARAAPEI-5, Unigene59310\_TAEpaeTARAAPEI-5, Unigene59314\_TAEpaeTARAAPEI-5, Unigene59391\_TAEpaeTARAAPEI-5, Unigene59850\_TAEpaeTARAAPEI-5, Unigene59993\_TAEpaeTARAAPEI-5, Unigene60103\_TAEpaeTARAAPEI-5, Unigene60126\_TAEpaeTARAAPEI-5, Unigene60254\_TAEpaeTARAAPEI-5, Unigene60456\_TAEpaeTARAAPEI-5, Unigene60471\_TAEpaeTARAAPEI-5, Unigene60882\_TAEpaeTARAAPEI-5, Unigene60901\_TAEpaeTARAAPEI-5, Unigene61029\_TAEpaeTARAAPEI-5, Unigene61076\_TAEpaeTARAAPEI-5, Unigene6116\_TAEpaeTARAAPEI-5, Unigene61347\_TAEpaeTARAAPEI-5, Unigene61391\_TAEpaeTARAAPEI-5, Unigene61763\_TAEpaeTARAAPEI-5, Unigene61861\_TAEpaeTARAAPEI-5, Unigene6194\_TAEpaeTARAAPEI-5, Unigene62103\_TAEpaeTARAAPEI-5, Unigene62268\_TAEpaeTARAAPEI-5, Unigene62275\_TAEpaeTARAAPEI-5, Unigene62671\_TAEpaeTARAAPEI-5, Unigene62793\_TAEpaeTARAAPEI-5, Unigene62975\_TAEpaeTARAAPEI-5, Unigene63280\_TAEpaeTARAAPEI-5, Unigene63370\_TAEpaeTARAAPEI-5, Unigene63495\_TAEpaeTARAAPEI-5, Unigene63523\_TAEpaeTARAAPEI-5, Unigene63527\_TAEpaeTARAAPEI-5, Unigene63745\_TAEpaeTARAAPEI-5, Unigene64293\_TAEpaeTARAAPEI-5, Unigene64422\_TAEpaeTARAAPEI-5, Unigene64437\_TAEpaeTARAAPEI-5, Unigene64687\_TAEpaeTARAAPEI-5, Unigene6471\_TAEpaeTARAAPEI-5, Unigene65279\_TAEpaeTARAAPEI-5, Unigene65594\_TAEpaeTARAAPEI-5, Unigene65617\_TAEpaeTARAAPEI-5, Unigene65703\_TAEpaeTARAAPEI-5, Unigene65827\_TAEpaeTARAAPEI-5, Unigene65920\_TAEpaeTARAAPEI-5, Unigene66152\_TAEpaeTARAAPEI-5, Unigene66211\_TAEpaeTARAAPEI-5, Unigene66212\_TAEpaeTARAAPEI-5, Unigene66291\_TAEpaeTARAAPEI-5, Unigene66540\_TAEpaeTARAAPEI-5, Unigene66651\_TAEpaeTARAAPEI-5, Unigene66675\_TAEpaeTARAAPEI-5, Unigene66904\_TAEpaeTARAAPEI-5, Unigene66955\_TAEpaeTARAAPEI-5, Unigene67066\_TAEpaeTARAAPEI-5, Unigene67196\_TAEpaeTARAAPEI-5, Unigene6726\_TAEpaeTARAAPEI-5, Unigene67341\_TAEpaeTARAAPEI-5, Unigene6745\_TAEpaeTARAAPEI-5, Unigene67524\_TAEpaeTARAAPEI-5, Unigene67635\_TAEpaeTARAAPEI-5, Unigene67781\_TAEpaeTARAAPEI-5, Unigene67951\_TAEpaeTARAAPEI-5, Unigene68026\_TAEpaeTARAAPEI-5, Unigene68071\_TAEpaeTARAAPEI-5, Unigene68103\_TAEpaeTARAAPEI-5, Unigene68142\_TAEpaeTARAAPEI-5, Unigene68491\_TAEpaeTARAAPEI-5, Unigene68616\_TAEpaeTARAAPEI-5, Unigene68739\_TAEpaeTARAAPEI-5, Unigene68841\_TAEpaeTARAAPEI-5, Unigene68903\_TAEpaeTARAAPEI-5, Unigene68964\_TAEpaeTARAAPEI-5, Unigene69044\_TAEpaeTARAAPEI-5, Unigene69372\_TAEpaeTARAAPEI-5, Unigene6938\_TAEpaeTARAAPEI-5, Unigene69439\_TAEpaeTARAAPEI-5, Unigene69550\_TAEpaeTARAAPEI-5, Unigene69577\_TAEpaeTARAAPEI-5, Unigene69580\_TAEpaeTARAAPEI-5, Unigene69632\_TAEpaeTARAAPEI-5, Unigene69684\_TAEpaeTARAAPEI-5, Unigene69730\_TAEpaeTARAAPEI-5, Unigene69930\_TAEpaeTARAAPEI-5, Unigene70029\_TAEpaeTARAAPEI-5, Unigene70104\_TAEpaeTARAAPEI-5, Unigene70224\_TAEpaeTARAAPEI-5, Unigene70309\_TAEpaeTARAAPEI-5, Unigene70495\_TAEpaeTARAAPEI-5, Unigene70535\_TAEpaeTARAAPEI-5, Unigene70546\_TAEpaeTARAAPEI-5, Unigene70596\_TAEpaeTARAAPEI-5, Unigene70615\_TAEpaeTARAAPEI-5, Unigene70893\_TAEpaeTARAAPEI-5, Unigene70977\_TAEpaeTARAAPEI-5, Unigene71127\_TAEpaeTARAAPEI-5, Unigene71194\_TAEpaeTARAAPEI-5, Unigene71224\_TAEpaeTARAAPEI-5, Unigene71238\_TAEpaeTARAAPEI-5, Unigene71368\_TAEpaeTARAAPEI-5, Unigene71440\_TAEpaeTARAAPEI-5, Unigene71442\_TAEpaeTARAAPEI-5, Unigene71472\_TAEpaeTARAAPEI-5, Unigene71553\_TAEpaeTARAAPEI-5, Unigene71617\_TAEpaeTARAAPEI-5, Unigene71641\_TAEpaeTARAAPEI-5, Unigene71682\_TAEpaeTARAAPEI-5, Unigene71696\_TAEpaeTARAAPEI-5, Unigene71804\_TAEpaeTARAAPEI-5, Unigene71822\_TAEpaeTARAAPEI-5, Unigene71828\_TAEpaeTARAAPEI-5, Unigene71839\_TAEpaeTARAAPEI-5, Unigene71841\_TAEpaeTARAAPEI-5, Unigene72005\_TAEpaeTARAAPEI-5, Unigene72058\_TAEpaeTARAAPEI-5, Unigene72145\_TAEpaeTARAAPEI-5, Unigene72164\_TAEpaeTARAAPEI-5, Unigene72168\_TAEpaeTARAAPEI-5, Unigene72261\_TAEpaeTARAAPEI-5, Unigene72315\_TAEpaeTARAAPEI-5, Unigene72322\_TAEpaeTARAAPEI-5, Unigene72323\_TAEpaeTARAAPEI-5, Unigene72337\_TAEpaeTARAAPEI-5, Unigene72340\_TAEpaeTARAAPEI-5, Unigene72399\_TAEpaeTARAAPEI-5, Unigene72473\_TAEpaeTARAAPEI-5, Unigene72487\_TAEpaeTARAAPEI-5, Unigene72540\_TAEpaeTARAAPEI-5, Unigene72568\_TAEpaeTARAAPEI-5, Unigene72670\_TAEpaeTARAAPEI-5, Unigene72675\_TAEpaeTARAAPEI-5, Unigene72691\_TAEpaeTARAAPEI-5, Unigene72711\_TAEpaeTARAAPEI-5, Unigene72736\_TAEpaeTARAAPEI-5, Unigene72751\_TAEpaeTARAAPEI-5, Unigene72752\_TAEpaeTARAAPEI-5, Unigene72768\_TAEpaeTARAAPEI-5, Unigene72786\_TAEpaeTARAAPEI-5, Unigene72853\_TAEpaeTARAAPEI-5, Unigene72926\_TAEpaeTARAAPEI-5, Unigene72947\_TAEpaeTARAAPEI-5, Unigene7463\_TAEpaeTARAAPEI-5, Unigene7574\_TAEpaeTARAAPEI-5, Unigene8228\_TAEpaeTARAAPEI-5, Unigene8302\_TAEpaeTARAAPEI-5, Unigene8505\_TAEpaeTARAAPEI-5, Unigene8873\_TAEpaeTARAAPEI-5, Unigene907\_TAEpaeTARAAPEI-5, Unigene9501\_TAEpaeTARAAPEI-5, Unigene9642\_TAEpaeTARAAPEI-5 |
| 18 | Lysosome | Unigene1034\_TAEpaeTARAAPEI-5, Unigene10695\_TAEpaeTARAAPEI-5, Unigene1082\_TAEpaeTARAAPEI-5, Unigene10999\_TAEpaeTARAAPEI-5, Unigene11396\_TAEpaeTARAAPEI-5, Unigene12235\_TAEpaeTARAAPEI-5, Unigene12303\_TAEpaeTARAAPEI-5, Unigene12448\_TAEpaeTARAAPEI-5, Unigene12505\_TAEpaeTARAAPEI-5, Unigene12634\_TAEpaeTARAAPEI-5, Unigene12706\_TAEpaeTARAAPEI-5, Unigene1274\_TAEpaeTARAAPEI-5, Unigene12830\_TAEpaeTARAAPEI-5, Unigene12939\_TAEpaeTARAAPEI-5, Unigene13246\_TAEpaeTARAAPEI-5, Unigene13298\_TAEpaeTARAAPEI-5, Unigene13320\_TAEpaeTARAAPEI-5, Unigene14193\_TAEpaeTARAAPEI-5, Unigene143\_TAEpaeTARAAPEI-5, Unigene14417\_TAEpaeTARAAPEI-5, Unigene14566\_TAEpaeTARAAPEI-5, Unigene14678\_TAEpaeTARAAPEI-5, Unigene15012\_TAEpaeTARAAPEI-5, Unigene15055\_TAEpaeTARAAPEI-5, Unigene1512\_TAEpaeTARAAPEI-5, Unigene15537\_TAEpaeTARAAPEI-5, Unigene1567\_TAEpaeTARAAPEI-5, Unigene15687\_TAEpaeTARAAPEI-5, Unigene15733\_TAEpaeTARAAPEI-5, Unigene15774\_TAEpaeTARAAPEI-5, Unigene15783\_TAEpaeTARAAPEI-5, Unigene1580\_TAEpaeTARAAPEI-5, Unigene15823\_TAEpaeTARAAPEI-5, Unigene15941\_TAEpaeTARAAPEI-5, Unigene15996\_TAEpaeTARAAPEI-5, Unigene16158\_TAEpaeTARAAPEI-5, Unigene16210\_TAEpaeTARAAPEI-5, Unigene16311\_TAEpaeTARAAPEI-5, Unigene16500\_TAEpaeTARAAPEI-5, Unigene16515\_TAEpaeTARAAPEI-5, Unigene16532\_TAEpaeTARAAPEI-5, Unigene16717\_TAEpaeTARAAPEI-5, Unigene16775\_TAEpaeTARAAPEI-5, Unigene16853\_TAEpaeTARAAPEI-5, Unigene16872\_TAEpaeTARAAPEI-5, Unigene16965\_TAEpaeTARAAPEI-5, Unigene17108\_TAEpaeTARAAPEI-5, Unigene17172\_TAEpaeTARAAPEI-5, Unigene17294\_TAEpaeTARAAPEI-5, Unigene17322\_TAEpaeTARAAPEI-5, Unigene17430\_TAEpaeTARAAPEI-5, Unigene17679\_TAEpaeTARAAPEI-5, Unigene17966\_TAEpaeTARAAPEI-5, Unigene17980\_TAEpaeTARAAPEI-5, Unigene18020\_TAEpaeTARAAPEI-5, Unigene18306\_TAEpaeTARAAPEI-5, Unigene18702\_TAEpaeTARAAPEI-5, Unigene1\_TAEpaeTARAAPEI-5, Unigene20068\_TAEpaeTARAAPEI-5, Unigene20543\_TAEpaeTARAAPEI-5, Unigene21183\_TAEpaeTARAAPEI-5, Unigene21252\_TAEpaeTARAAPEI-5, Unigene21780\_TAEpaeTARAAPEI-5, Unigene22646\_TAEpaeTARAAPEI-5, Unigene2265\_TAEpaeTARAAPEI-5, Unigene23038\_TAEpaeTARAAPEI-5, Unigene23345\_TAEpaeTARAAPEI-5, Unigene24306\_TAEpaeTARAAPEI-5, Unigene24313\_TAEpaeTARAAPEI-5, Unigene24316\_TAEpaeTARAAPEI-5, Unigene2474\_TAEpaeTARAAPEI-5, Unigene25592\_TAEpaeTARAAPEI-5, Unigene25731\_TAEpaeTARAAPEI-5, Unigene263\_TAEpaeTARAAPEI-5, Unigene26726\_TAEpaeTARAAPEI-5, Unigene26907\_TAEpaeTARAAPEI-5, Unigene27598\_TAEpaeTARAAPEI-5, Unigene27690\_TAEpaeTARAAPEI-5, Unigene27786\_TAEpaeTARAAPEI-5, Unigene27846\_TAEpaeTARAAPEI-5, Unigene27983\_TAEpaeTARAAPEI-5, Unigene28463\_TAEpaeTARAAPEI-5, Unigene28874\_TAEpaeTARAAPEI-5, Unigene28951\_TAEpaeTARAAPEI-5, Unigene28994\_TAEpaeTARAAPEI-5, Unigene28\_TAEpaeTARAAPEI-5, Unigene29247\_TAEpaeTARAAPEI-5, Unigene30191\_TAEpaeTARAAPEI-5, Unigene30560\_TAEpaeTARAAPEI-5, Unigene31040\_TAEpaeTARAAPEI-5, Unigene31130\_TAEpaeTARAAPEI-5, Unigene31263\_TAEpaeTARAAPEI-5, Unigene31467\_TAEpaeTARAAPEI-5, Unigene31806\_TAEpaeTARAAPEI-5, Unigene33104\_TAEpaeTARAAPEI-5, Unigene33506\_TAEpaeTARAAPEI-5, Unigene33517\_TAEpaeTARAAPEI-5, Unigene336\_TAEpaeTARAAPEI-5, Unigene3371\_TAEpaeTARAAPEI-5, Unigene33801\_TAEpaeTARAAPEI-5, Unigene34212\_TAEpaeTARAAPEI-5, Unigene34426\_TAEpaeTARAAPEI-5, Unigene34452\_TAEpaeTARAAPEI-5, Unigene3447\_TAEpaeTARAAPEI-5, Unigene34549\_TAEpaeTARAAPEI-5, Unigene34644\_TAEpaeTARAAPEI-5, Unigene34671\_TAEpaeTARAAPEI-5, Unigene35399\_TAEpaeTARAAPEI-5, Unigene3628\_TAEpaeTARAAPEI-5, Unigene36454\_TAEpaeTARAAPEI-5, Unigene36616\_TAEpaeTARAAPEI-5, Unigene3674\_TAEpaeTARAAPEI-5, Unigene37067\_TAEpaeTARAAPEI-5, Unigene37139\_TAEpaeTARAAPEI-5, Unigene37554\_TAEpaeTARAAPEI-5, Unigene38499\_TAEpaeTARAAPEI-5, Unigene38565\_TAEpaeTARAAPEI-5, Unigene38576\_TAEpaeTARAAPEI-5, Unigene39335\_TAEpaeTARAAPEI-5, Unigene39546\_TAEpaeTARAAPEI-5, Unigene39850\_TAEpaeTARAAPEI-5, Unigene39978\_TAEpaeTARAAPEI-5, Unigene40104\_TAEpaeTARAAPEI-5, Unigene40405\_TAEpaeTARAAPEI-5, Unigene40465\_TAEpaeTARAAPEI-5, Unigene40581\_TAEpaeTARAAPEI-5, Unigene40780\_TAEpaeTARAAPEI-5, Unigene40988\_TAEpaeTARAAPEI-5, Unigene41199\_TAEpaeTARAAPEI-5, Unigene41803\_TAEpaeTARAAPEI-5, Unigene41867\_TAEpaeTARAAPEI-5, Unigene42186\_TAEpaeTARAAPEI-5, Unigene42441\_TAEpaeTARAAPEI-5, Unigene42876\_TAEpaeTARAAPEI-5, Unigene42919\_TAEpaeTARAAPEI-5, Unigene43139\_TAEpaeTARAAPEI-5, Unigene43279\_TAEpaeTARAAPEI-5, Unigene43634\_TAEpaeTARAAPEI-5, Unigene43808\_TAEpaeTARAAPEI-5, Unigene44274\_TAEpaeTARAAPEI-5, Unigene44483\_TAEpaeTARAAPEI-5, Unigene44528\_TAEpaeTARAAPEI-5, Unigene44642\_TAEpaeTARAAPEI-5, Unigene44739\_TAEpaeTARAAPEI-5, Unigene45033\_TAEpaeTARAAPEI-5, Unigene45038\_TAEpaeTARAAPEI-5, Unigene45047\_TAEpaeTARAAPEI-5, Unigene45202\_TAEpaeTARAAPEI-5, Unigene45353\_TAEpaeTARAAPEI-5, Unigene45609\_TAEpaeTARAAPEI-5, Unigene45652\_TAEpaeTARAAPEI-5, Unigene45785\_TAEpaeTARAAPEI-5, Unigene45871\_TAEpaeTARAAPEI-5, Unigene45976\_TAEpaeTARAAPEI-5, Unigene46230\_TAEpaeTARAAPEI-5, Unigene46264\_TAEpaeTARAAPEI-5, Unigene46567\_TAEpaeTARAAPEI-5, Unigene46603\_TAEpaeTARAAPEI-5, Unigene46730\_TAEpaeTARAAPEI-5, Unigene46926\_TAEpaeTARAAPEI-5, Unigene47501\_TAEpaeTARAAPEI-5, Unigene47815\_TAEpaeTARAAPEI-5, Unigene47863\_TAEpaeTARAAPEI-5, Unigene47876\_TAEpaeTARAAPEI-5, Unigene48384\_TAEpaeTARAAPEI-5, Unigene48948\_TAEpaeTARAAPEI-5, Unigene49074\_TAEpaeTARAAPEI-5, Unigene49826\_TAEpaeTARAAPEI-5, Unigene50315\_TAEpaeTARAAPEI-5, Unigene50348\_TAEpaeTARAAPEI-5, Unigene50618\_TAEpaeTARAAPEI-5, Unigene50640\_TAEpaeTARAAPEI-5, Unigene50816\_TAEpaeTARAAPEI-5, Unigene50826\_TAEpaeTARAAPEI-5, Unigene51098\_TAEpaeTARAAPEI-5, Unigene5130\_TAEpaeTARAAPEI-5, Unigene51645\_TAEpaeTARAAPEI-5, Unigene51670\_TAEpaeTARAAPEI-5, Unigene51886\_TAEpaeTARAAPEI-5, Unigene52180\_TAEpaeTARAAPEI-5, Unigene52278\_TAEpaeTARAAPEI-5, Unigene52519\_TAEpaeTARAAPEI-5, Unigene52998\_TAEpaeTARAAPEI-5, Unigene53178\_TAEpaeTARAAPEI-5, Unigene53299\_TAEpaeTARAAPEI-5, Unigene53467\_TAEpaeTARAAPEI-5, Unigene53536\_TAEpaeTARAAPEI-5, Unigene53552\_TAEpaeTARAAPEI-5, Unigene53883\_TAEpaeTARAAPEI-5, Unigene53944\_TAEpaeTARAAPEI-5, Unigene54034\_TAEpaeTARAAPEI-5, Unigene54243\_TAEpaeTARAAPEI-5, Unigene54530\_TAEpaeTARAAPEI-5, Unigene54577\_TAEpaeTARAAPEI-5, Unigene54632\_TAEpaeTARAAPEI-5, Unigene54850\_TAEpaeTARAAPEI-5, Unigene55016\_TAEpaeTARAAPEI-5, Unigene55207\_TAEpaeTARAAPEI-5, Unigene55363\_TAEpaeTARAAPEI-5, Unigene55369\_TAEpaeTARAAPEI-5, Unigene55449\_TAEpaeTARAAPEI-5, Unigene55560\_TAEpaeTARAAPEI-5, Unigene55636\_TAEpaeTARAAPEI-5, Unigene56146\_TAEpaeTARAAPEI-5, Unigene56160\_TAEpaeTARAAPEI-5, Unigene56164\_TAEpaeTARAAPEI-5, Unigene56268\_TAEpaeTARAAPEI-5, Unigene56347\_TAEpaeTARAAPEI-5, Unigene56366\_TAEpaeTARAAPEI-5, Unigene56450\_TAEpaeTARAAPEI-5, Unigene56551\_TAEpaeTARAAPEI-5, Unigene56730\_TAEpaeTARAAPEI-5, Unigene57035\_TAEpaeTARAAPEI-5, Unigene57195\_TAEpaeTARAAPEI-5, Unigene57239\_TAEpaeTARAAPEI-5, Unigene574\_TAEpaeTARAAPEI-5, Unigene57803\_TAEpaeTARAAPEI-5, Unigene57907\_TAEpaeTARAAPEI-5, Unigene57998\_TAEpaeTARAAPEI-5, Unigene58064\_TAEpaeTARAAPEI-5, Unigene58275\_TAEpaeTARAAPEI-5, Unigene58514\_TAEpaeTARAAPEI-5, Unigene58614\_TAEpaeTARAAPEI-5, Unigene59006\_TAEpaeTARAAPEI-5, Unigene59080\_TAEpaeTARAAPEI-5, Unigene59611\_TAEpaeTARAAPEI-5, Unigene59690\_TAEpaeTARAAPEI-5, Unigene59748\_TAEpaeTARAAPEI-5, Unigene5976\_TAEpaeTARAAPEI-5, Unigene59782\_TAEpaeTARAAPEI-5, Unigene59905\_TAEpaeTARAAPEI-5, Unigene60182\_TAEpaeTARAAPEI-5, Unigene60936\_TAEpaeTARAAPEI-5, Unigene61077\_TAEpaeTARAAPEI-5, Unigene61259\_TAEpaeTARAAPEI-5, Unigene61553\_TAEpaeTARAAPEI-5, Unigene61676\_TAEpaeTARAAPEI-5, Unigene61846\_TAEpaeTARAAPEI-5, Unigene61958\_TAEpaeTARAAPEI-5, Unigene62168\_TAEpaeTARAAPEI-5, Unigene62197\_TAEpaeTARAAPEI-5, Unigene62288\_TAEpaeTARAAPEI-5, Unigene62308\_TAEpaeTARAAPEI-5, Unigene6231\_TAEpaeTARAAPEI-5, Unigene6234\_TAEpaeTARAAPEI-5, Unigene62359\_TAEpaeTARAAPEI-5, Unigene62593\_TAEpaeTARAAPEI-5, Unigene62796\_TAEpaeTARAAPEI-5, Unigene62915\_TAEpaeTARAAPEI-5, Unigene62990\_TAEpaeTARAAPEI-5, Unigene63544\_TAEpaeTARAAPEI-5, Unigene63552\_TAEpaeTARAAPEI-5, Unigene63671\_TAEpaeTARAAPEI-5, Unigene63744\_TAEpaeTARAAPEI-5, Unigene63888\_TAEpaeTARAAPEI-5, Unigene6389\_TAEpaeTARAAPEI-5, Unigene63921\_TAEpaeTARAAPEI-5, Unigene64106\_TAEpaeTARAAPEI-5, Unigene64311\_TAEpaeTARAAPEI-5, Unigene64320\_TAEpaeTARAAPEI-5, Unigene64363\_TAEpaeTARAAPEI-5, Unigene64465\_TAEpaeTARAAPEI-5, Unigene64544\_TAEpaeTARAAPEI-5, Unigene64563\_TAEpaeTARAAPEI-5, Unigene64717\_TAEpaeTARAAPEI-5, Unigene64891\_TAEpaeTARAAPEI-5, Unigene64965\_TAEpaeTARAAPEI-5, Unigene65016\_TAEpaeTARAAPEI-5, Unigene65163\_TAEpaeTARAAPEI-5, Unigene6516\_TAEpaeTARAAPEI-5, Unigene65211\_TAEpaeTARAAPEI-5, Unigene65277\_TAEpaeTARAAPEI-5, Unigene65285\_TAEpaeTARAAPEI-5, Unigene65344\_TAEpaeTARAAPEI-5, Unigene65361\_TAEpaeTARAAPEI-5, Unigene65427\_TAEpaeTARAAPEI-5, Unigene65429\_TAEpaeTARAAPEI-5, Unigene65446\_TAEpaeTARAAPEI-5, Unigene65453\_TAEpaeTARAAPEI-5, Unigene65492\_TAEpaeTARAAPEI-5, Unigene65751\_TAEpaeTARAAPEI-5, Unigene65987\_TAEpaeTARAAPEI-5, Unigene66018\_TAEpaeTARAAPEI-5, Unigene66141\_TAEpaeTARAAPEI-5, Unigene66261\_TAEpaeTARAAPEI-5, Unigene66524\_TAEpaeTARAAPEI-5, Unigene66536\_TAEpaeTARAAPEI-5, Unigene6655\_TAEpaeTARAAPEI-5, Unigene66610\_TAEpaeTARAAPEI-5, Unigene6663\_TAEpaeTARAAPEI-5, Unigene66691\_TAEpaeTARAAPEI-5, Unigene66699\_TAEpaeTARAAPEI-5, Unigene66716\_TAEpaeTARAAPEI-5, Unigene66728\_TAEpaeTARAAPEI-5, Unigene66865\_TAEpaeTARAAPEI-5, Unigene66877\_TAEpaeTARAAPEI-5, Unigene67050\_TAEpaeTARAAPEI-5, Unigene67103\_TAEpaeTARAAPEI-5, Unigene67142\_TAEpaeTARAAPEI-5, Unigene67200\_TAEpaeTARAAPEI-5, Unigene67346\_TAEpaeTARAAPEI-5, Unigene67362\_TAEpaeTARAAPEI-5, Unigene67439\_TAEpaeTARAAPEI-5, Unigene67443\_TAEpaeTARAAPEI-5, Unigene67507\_TAEpaeTARAAPEI-5, Unigene6760\_TAEpaeTARAAPEI-5, Unigene67696\_TAEpaeTARAAPEI-5, Unigene67807\_TAEpaeTARAAPEI-5, Unigene67825\_TAEpaeTARAAPEI-5, Unigene68211\_TAEpaeTARAAPEI-5, Unigene68288\_TAEpaeTARAAPEI-5, Unigene68463\_TAEpaeTARAAPEI-5, Unigene68552\_TAEpaeTARAAPEI-5, Unigene68618\_TAEpaeTARAAPEI-5, Unigene68844\_TAEpaeTARAAPEI-5, Unigene68846\_TAEpaeTARAAPEI-5, Unigene69064\_TAEpaeTARAAPEI-5, Unigene69176\_TAEpaeTARAAPEI-5, Unigene69179\_TAEpaeTARAAPEI-5, Unigene69227\_TAEpaeTARAAPEI-5, Unigene69298\_TAEpaeTARAAPEI-5, Unigene69721\_TAEpaeTARAAPEI-5, Unigene69722\_TAEpaeTARAAPEI-5, Unigene69812\_TAEpaeTARAAPEI-5, Unigene69943\_TAEpaeTARAAPEI-5, Unigene70000\_TAEpaeTARAAPEI-5, Unigene70400\_TAEpaeTARAAPEI-5, Unigene70520\_TAEpaeTARAAPEI-5, Unigene70687\_TAEpaeTARAAPEI-5, Unigene70810\_TAEpaeTARAAPEI-5, Unigene70861\_TAEpaeTARAAPEI-5, Unigene70879\_TAEpaeTARAAPEI-5, Unigene71116\_TAEpaeTARAAPEI-5, Unigene71133\_TAEpaeTARAAPEI-5, Unigene71136\_TAEpaeTARAAPEI-5, Unigene71280\_TAEpaeTARAAPEI-5, Unigene71314\_TAEpaeTARAAPEI-5, Unigene71357\_TAEpaeTARAAPEI-5, Unigene71396\_TAEpaeTARAAPEI-5, Unigene71397\_TAEpaeTARAAPEI-5, Unigene71487\_TAEpaeTARAAPEI-5, Unigene71535\_TAEpaeTARAAPEI-5, Unigene71556\_TAEpaeTARAAPEI-5, Unigene71582\_TAEpaeTARAAPEI-5, Unigene7158\_TAEpaeTARAAPEI-5, Unigene71592\_TAEpaeTARAAPEI-5, Unigene71673\_TAEpaeTARAAPEI-5, Unigene71766\_TAEpaeTARAAPEI-5, Unigene71855\_TAEpaeTARAAPEI-5, Unigene71881\_TAEpaeTARAAPEI-5, Unigene71989\_TAEpaeTARAAPEI-5, Unigene71991\_TAEpaeTARAAPEI-5, Unigene72032\_TAEpaeTARAAPEI-5, Unigene72116\_TAEpaeTARAAPEI-5, Unigene72146\_TAEpaeTARAAPEI-5, Unigene72206\_TAEpaeTARAAPEI-5, Unigene72324\_TAEpaeTARAAPEI-5, Unigene72475\_TAEpaeTARAAPEI-5, Unigene72641\_TAEpaeTARAAPEI-5, Unigene75\_TAEpaeTARAAPEI-5, Unigene7626\_TAEpaeTARAAPEI-5, Unigene7724\_TAEpaeTARAAPEI-5, Unigene7901\_TAEpaeTARAAPEI-5, Unigene8546\_TAEpaeTARAAPEI-5, Unigene8797\_TAEpaeTARAAPEI-5, Unigene8892\_TAEpaeTARAAPEI-5, Unigene9074\_TAEpaeTARAAPEI-5, Unigene9320\_TAEpaeTARAAPEI-5, Unigene9498\_TAEpaeTARAAPEI-5, Unigene9650\_TAEpaeTARAAPEI-5, Unigene9902\_TAEpaeTARAAPEI-5 |
| 19 | Dilated cardiomyopathy | Unigene10250\_TAEpaeTARAAPEI-5, Unigene10302\_TAEpaeTARAAPEI-5, Unigene10544\_TAEpaeTARAAPEI-5, Unigene10616\_TAEpaeTARAAPEI-5, Unigene10658\_TAEpaeTARAAPEI-5, Unigene10689\_TAEpaeTARAAPEI-5, Unigene10720\_TAEpaeTARAAPEI-5, Unigene10835\_TAEpaeTARAAPEI-5, Unigene10863\_TAEpaeTARAAPEI-5, Unigene1087\_TAEpaeTARAAPEI-5, Unigene11214\_TAEpaeTARAAPEI-5, Unigene11280\_TAEpaeTARAAPEI-5, Unigene11534\_TAEpaeTARAAPEI-5, Unigene12145\_TAEpaeTARAAPEI-5, Unigene12167\_TAEpaeTARAAPEI-5, Unigene12235\_TAEpaeTARAAPEI-5, Unigene12247\_TAEpaeTARAAPEI-5, Unigene12401\_TAEpaeTARAAPEI-5, Unigene12600\_TAEpaeTARAAPEI-5, Unigene12878\_TAEpaeTARAAPEI-5, Unigene12921\_TAEpaeTARAAPEI-5, Unigene12947\_TAEpaeTARAAPEI-5, Unigene13000\_TAEpaeTARAAPEI-5, Unigene13163\_TAEpaeTARAAPEI-5, Unigene13484\_TAEpaeTARAAPEI-5, Unigene13532\_TAEpaeTARAAPEI-5, Unigene13666\_TAEpaeTARAAPEI-5, Unigene13919\_TAEpaeTARAAPEI-5, Unigene14224\_TAEpaeTARAAPEI-5, Unigene14362\_TAEpaeTARAAPEI-5, Unigene15053\_TAEpaeTARAAPEI-5, Unigene15107\_TAEpaeTARAAPEI-5, Unigene15331\_TAEpaeTARAAPEI-5, Unigene15361\_TAEpaeTARAAPEI-5, Unigene15549\_TAEpaeTARAAPEI-5, Unigene15602\_TAEpaeTARAAPEI-5, Unigene15785\_TAEpaeTARAAPEI-5, Unigene15881\_TAEpaeTARAAPEI-5, Unigene1593\_TAEpaeTARAAPEI-5, Unigene15971\_TAEpaeTARAAPEI-5, Unigene16134\_TAEpaeTARAAPEI-5, Unigene16406\_TAEpaeTARAAPEI-5, Unigene16411\_TAEpaeTARAAPEI-5, Unigene16509\_TAEpaeTARAAPEI-5, Unigene16802\_TAEpaeTARAAPEI-5, Unigene17109\_TAEpaeTARAAPEI-5, Unigene17145\_TAEpaeTARAAPEI-5, Unigene17252\_TAEpaeTARAAPEI-5, Unigene17354\_TAEpaeTARAAPEI-5, Unigene17420\_TAEpaeTARAAPEI-5, Unigene17551\_TAEpaeTARAAPEI-5, Unigene17640\_TAEpaeTARAAPEI-5, Unigene17644\_TAEpaeTARAAPEI-5, Unigene17731\_TAEpaeTARAAPEI-5, Unigene17741\_TAEpaeTARAAPEI-5, Unigene17865\_TAEpaeTARAAPEI-5, Unigene17901\_TAEpaeTARAAPEI-5, Unigene17977\_TAEpaeTARAAPEI-5, Unigene17995\_TAEpaeTARAAPEI-5, Unigene18266\_TAEpaeTARAAPEI-5, Unigene19396\_TAEpaeTARAAPEI-5, Unigene19465\_TAEpaeTARAAPEI-5, Unigene19937\_TAEpaeTARAAPEI-5, Unigene20000\_TAEpaeTARAAPEI-5, Unigene20359\_TAEpaeTARAAPEI-5, Unigene20403\_TAEpaeTARAAPEI-5, Unigene20655\_TAEpaeTARAAPEI-5, Unigene21513\_TAEpaeTARAAPEI-5, Unigene22021\_TAEpaeTARAAPEI-5, Unigene22358\_TAEpaeTARAAPEI-5, Unigene23436\_TAEpaeTARAAPEI-5, Unigene24220\_TAEpaeTARAAPEI-5, Unigene2439\_TAEpaeTARAAPEI-5, Unigene24538\_TAEpaeTARAAPEI-5, Unigene25970\_TAEpaeTARAAPEI-5, Unigene26031\_TAEpaeTARAAPEI-5, Unigene26359\_TAEpaeTARAAPEI-5, Unigene2701\_TAEpaeTARAAPEI-5, Unigene27281\_TAEpaeTARAAPEI-5, Unigene27972\_TAEpaeTARAAPEI-5, Unigene2889\_TAEpaeTARAAPEI-5, Unigene28987\_TAEpaeTARAAPEI-5, Unigene29157\_TAEpaeTARAAPEI-5, Unigene29171\_TAEpaeTARAAPEI-5, Unigene30172\_TAEpaeTARAAPEI-5, Unigene30758\_TAEpaeTARAAPEI-5, Unigene30885\_TAEpaeTARAAPEI-5, Unigene30916\_TAEpaeTARAAPEI-5, Unigene31474\_TAEpaeTARAAPEI-5, Unigene3158\_TAEpaeTARAAPEI-5, Unigene31750\_TAEpaeTARAAPEI-5, Unigene31929\_TAEpaeTARAAPEI-5, Unigene32212\_TAEpaeTARAAPEI-5, Unigene32294\_TAEpaeTARAAPEI-5, Unigene32782\_TAEpaeTARAAPEI-5, Unigene32999\_TAEpaeTARAAPEI-5, Unigene33030\_TAEpaeTARAAPEI-5, Unigene33057\_TAEpaeTARAAPEI-5, Unigene33174\_TAEpaeTARAAPEI-5, Unigene3447\_TAEpaeTARAAPEI-5, Unigene34543\_TAEpaeTARAAPEI-5, Unigene35882\_TAEpaeTARAAPEI-5, Unigene3622\_TAEpaeTARAAPEI-5, Unigene36648\_TAEpaeTARAAPEI-5, Unigene36858\_TAEpaeTARAAPEI-5, Unigene36887\_TAEpaeTARAAPEI-5, Unigene37149\_TAEpaeTARAAPEI-5, Unigene37671\_TAEpaeTARAAPEI-5, Unigene37870\_TAEpaeTARAAPEI-5, Unigene38160\_TAEpaeTARAAPEI-5, Unigene38258\_TAEpaeTARAAPEI-5, Unigene3828\_TAEpaeTARAAPEI-5, Unigene38499\_TAEpaeTARAAPEI-5, Unigene38667\_TAEpaeTARAAPEI-5, Unigene39227\_TAEpaeTARAAPEI-5, Unigene39254\_TAEpaeTARAAPEI-5, Unigene39341\_TAEpaeTARAAPEI-5, Unigene39638\_TAEpaeTARAAPEI-5, Unigene39872\_TAEpaeTARAAPEI-5, Unigene39926\_TAEpaeTARAAPEI-5, Unigene40073\_TAEpaeTARAAPEI-5, Unigene40763\_TAEpaeTARAAPEI-5, Unigene40775\_TAEpaeTARAAPEI-5, Unigene41593\_TAEpaeTARAAPEI-5, Unigene43003\_TAEpaeTARAAPEI-5, Unigene43326\_TAEpaeTARAAPEI-5, Unigene43663\_TAEpaeTARAAPEI-5, Unigene43738\_TAEpaeTARAAPEI-5, Unigene43795\_TAEpaeTARAAPEI-5, Unigene4423\_TAEpaeTARAAPEI-5, Unigene44324\_TAEpaeTARAAPEI-5, Unigene44457\_TAEpaeTARAAPEI-5, Unigene44963\_TAEpaeTARAAPEI-5, Unigene45049\_TAEpaeTARAAPEI-5, Unigene45410\_TAEpaeTARAAPEI-5, Unigene45651\_TAEpaeTARAAPEI-5, Unigene45779\_TAEpaeTARAAPEI-5, Unigene46118\_TAEpaeTARAAPEI-5, Unigene46169\_TAEpaeTARAAPEI-5, Unigene46659\_TAEpaeTARAAPEI-5, Unigene47434\_TAEpaeTARAAPEI-5, Unigene47449\_TAEpaeTARAAPEI-5, Unigene47954\_TAEpaeTARAAPEI-5, Unigene48481\_TAEpaeTARAAPEI-5, Unigene48695\_TAEpaeTARAAPEI-5, Unigene48705\_TAEpaeTARAAPEI-5, Unigene48917\_TAEpaeTARAAPEI-5, Unigene49598\_TAEpaeTARAAPEI-5, Unigene49971\_TAEpaeTARAAPEI-5, Unigene51767\_TAEpaeTARAAPEI-5, Unigene51813\_TAEpaeTARAAPEI-5, Unigene52269\_TAEpaeTARAAPEI-5, Unigene52306\_TAEpaeTARAAPEI-5, Unigene52486\_TAEpaeTARAAPEI-5, Unigene52928\_TAEpaeTARAAPEI-5, Unigene53106\_TAEpaeTARAAPEI-5, Unigene5380\_TAEpaeTARAAPEI-5, Unigene53837\_TAEpaeTARAAPEI-5, Unigene54156\_TAEpaeTARAAPEI-5, Unigene54302\_TAEpaeTARAAPEI-5, Unigene54313\_TAEpaeTARAAPEI-5, Unigene55063\_TAEpaeTARAAPEI-5, Unigene5555\_TAEpaeTARAAPEI-5, Unigene55789\_TAEpaeTARAAPEI-5, Unigene55790\_TAEpaeTARAAPEI-5, Unigene56274\_TAEpaeTARAAPEI-5, Unigene56500\_TAEpaeTARAAPEI-5, Unigene56551\_TAEpaeTARAAPEI-5, Unigene56591\_TAEpaeTARAAPEI-5, Unigene56770\_TAEpaeTARAAPEI-5, Unigene56793\_TAEpaeTARAAPEI-5, Unigene56816\_TAEpaeTARAAPEI-5, Unigene56917\_TAEpaeTARAAPEI-5, Unigene57076\_TAEpaeTARAAPEI-5, Unigene57371\_TAEpaeTARAAPEI-5, Unigene57376\_TAEpaeTARAAPEI-5, Unigene57397\_TAEpaeTARAAPEI-5, Unigene57528\_TAEpaeTARAAPEI-5, Unigene57868\_TAEpaeTARAAPEI-5, Unigene58297\_TAEpaeTARAAPEI-5, Unigene58568\_TAEpaeTARAAPEI-5, Unigene58651\_TAEpaeTARAAPEI-5, Unigene58938\_TAEpaeTARAAPEI-5, Unigene59482\_TAEpaeTARAAPEI-5, Unigene59529\_TAEpaeTARAAPEI-5, Unigene5956\_TAEpaeTARAAPEI-5, Unigene59670\_TAEpaeTARAAPEI-5, Unigene59674\_TAEpaeTARAAPEI-5, Unigene6030\_TAEpaeTARAAPEI-5, Unigene60330\_TAEpaeTARAAPEI-5, Unigene60534\_TAEpaeTARAAPEI-5, Unigene6092\_TAEpaeTARAAPEI-5, Unigene60986\_TAEpaeTARAAPEI-5, Unigene61103\_TAEpaeTARAAPEI-5, Unigene61108\_TAEpaeTARAAPEI-5, Unigene61114\_TAEpaeTARAAPEI-5, Unigene61489\_TAEpaeTARAAPEI-5, Unigene6150\_TAEpaeTARAAPEI-5, Unigene61806\_TAEpaeTARAAPEI-5, Unigene61820\_TAEpaeTARAAPEI-5, Unigene61823\_TAEpaeTARAAPEI-5, Unigene62064\_TAEpaeTARAAPEI-5, Unigene62072\_TAEpaeTARAAPEI-5, Unigene62171\_TAEpaeTARAAPEI-5, Unigene62217\_TAEpaeTARAAPEI-5, Unigene62690\_TAEpaeTARAAPEI-5, Unigene62920\_TAEpaeTARAAPEI-5, Unigene63148\_TAEpaeTARAAPEI-5, Unigene63412\_TAEpaeTARAAPEI-5, Unigene63607\_TAEpaeTARAAPEI-5, Unigene63806\_TAEpaeTARAAPEI-5, Unigene64029\_TAEpaeTARAAPEI-5, Unigene64040\_TAEpaeTARAAPEI-5, Unigene64361\_TAEpaeTARAAPEI-5, Unigene64410\_TAEpaeTARAAPEI-5, Unigene64439\_TAEpaeTARAAPEI-5, Unigene64467\_TAEpaeTARAAPEI-5, Unigene6449\_TAEpaeTARAAPEI-5, Unigene64529\_TAEpaeTARAAPEI-5, Unigene64865\_TAEpaeTARAAPEI-5, Unigene65037\_TAEpaeTARAAPEI-5, Unigene65314\_TAEpaeTARAAPEI-5, Unigene65322\_TAEpaeTARAAPEI-5, Unigene65570\_TAEpaeTARAAPEI-5, Unigene65670\_TAEpaeTARAAPEI-5, Unigene65685\_TAEpaeTARAAPEI-5, Unigene65701\_TAEpaeTARAAPEI-5, Unigene66041\_TAEpaeTARAAPEI-5, Unigene66043\_TAEpaeTARAAPEI-5, Unigene6605\_TAEpaeTARAAPEI-5, Unigene66212\_TAEpaeTARAAPEI-5, Unigene66398\_TAEpaeTARAAPEI-5, Unigene66694\_TAEpaeTARAAPEI-5, Unigene66888\_TAEpaeTARAAPEI-5, Unigene66931\_TAEpaeTARAAPEI-5, Unigene67063\_TAEpaeTARAAPEI-5, Unigene67074\_TAEpaeTARAAPEI-5, Unigene67204\_TAEpaeTARAAPEI-5, Unigene67236\_TAEpaeTARAAPEI-5, Unigene67745\_TAEpaeTARAAPEI-5, Unigene67748\_TAEpaeTARAAPEI-5, Unigene67889\_TAEpaeTARAAPEI-5, Unigene6798\_TAEpaeTARAAPEI-5, Unigene68274\_TAEpaeTARAAPEI-5, Unigene68614\_TAEpaeTARAAPEI-5, Unigene68625\_TAEpaeTARAAPEI-5, Unigene68744\_TAEpaeTARAAPEI-5, Unigene68874\_TAEpaeTARAAPEI-5, Unigene68973\_TAEpaeTARAAPEI-5, Unigene68984\_TAEpaeTARAAPEI-5, Unigene68994\_TAEpaeTARAAPEI-5, Unigene69062\_TAEpaeTARAAPEI-5, Unigene69275\_TAEpaeTARAAPEI-5, Unigene69370\_TAEpaeTARAAPEI-5, Unigene6938\_TAEpaeTARAAPEI-5, Unigene6940\_TAEpaeTARAAPEI-5, Unigene69421\_TAEpaeTARAAPEI-5, Unigene69441\_TAEpaeTARAAPEI-5, Unigene69776\_TAEpaeTARAAPEI-5, Unigene69849\_TAEpaeTARAAPEI-5, Unigene69955\_TAEpaeTARAAPEI-5, Unigene69963\_TAEpaeTARAAPEI-5, Unigene70033\_TAEpaeTARAAPEI-5, Unigene70053\_TAEpaeTARAAPEI-5, Unigene70074\_TAEpaeTARAAPEI-5, Unigene70103\_TAEpaeTARAAPEI-5, Unigene7011\_TAEpaeTARAAPEI-5, Unigene70176\_TAEpaeTARAAPEI-5, Unigene70182\_TAEpaeTARAAPEI-5, Unigene70243\_TAEpaeTARAAPEI-5, Unigene70316\_TAEpaeTARAAPEI-5, Unigene70372\_TAEpaeTARAAPEI-5, Unigene70480\_TAEpaeTARAAPEI-5, Unigene70509\_TAEpaeTARAAPEI-5, Unigene70533\_TAEpaeTARAAPEI-5, Unigene70544\_TAEpaeTARAAPEI-5, Unigene70607\_TAEpaeTARAAPEI-5, Unigene70615\_TAEpaeTARAAPEI-5, Unigene70647\_TAEpaeTARAAPEI-5, Unigene70648\_TAEpaeTARAAPEI-5, Unigene70692\_TAEpaeTARAAPEI-5, Unigene70761\_TAEpaeTARAAPEI-5, Unigene70801\_TAEpaeTARAAPEI-5, Unigene70941\_TAEpaeTARAAPEI-5, Unigene70959\_TAEpaeTARAAPEI-5, Unigene71011\_TAEpaeTARAAPEI-5, Unigene71038\_TAEpaeTARAAPEI-5, Unigene71084\_TAEpaeTARAAPEI-5, Unigene71102\_TAEpaeTARAAPEI-5, Unigene71120\_TAEpaeTARAAPEI-5, Unigene71122\_TAEpaeTARAAPEI-5, Unigene71182\_TAEpaeTARAAPEI-5, Unigene71200\_TAEpaeTARAAPEI-5, Unigene71263\_TAEpaeTARAAPEI-5, Unigene71305\_TAEpaeTARAAPEI-5, Unigene71310\_TAEpaeTARAAPEI-5, Unigene71380\_TAEpaeTARAAPEI-5, Unigene71472\_TAEpaeTARAAPEI-5, Unigene71473\_TAEpaeTARAAPEI-5, Unigene71482\_TAEpaeTARAAPEI-5, Unigene71574\_TAEpaeTARAAPEI-5, Unigene71585\_TAEpaeTARAAPEI-5, Unigene71621\_TAEpaeTARAAPEI-5, Unigene71633\_TAEpaeTARAAPEI-5, Unigene71752\_TAEpaeTARAAPEI-5, Unigene71835\_TAEpaeTARAAPEI-5, Unigene71895\_TAEpaeTARAAPEI-5, Unigene71926\_TAEpaeTARAAPEI-5, Unigene72045\_TAEpaeTARAAPEI-5, Unigene72087\_TAEpaeTARAAPEI-5, Unigene72157\_TAEpaeTARAAPEI-5, Unigene72178\_TAEpaeTARAAPEI-5, Unigene72181\_TAEpaeTARAAPEI-5, Unigene72211\_TAEpaeTARAAPEI-5, Unigene72213\_TAEpaeTARAAPEI-5, Unigene72266\_TAEpaeTARAAPEI-5, Unigene72355\_TAEpaeTARAAPEI-5, Unigene72392\_TAEpaeTARAAPEI-5, Unigene72449\_TAEpaeTARAAPEI-5, Unigene72471\_TAEpaeTARAAPEI-5, Unigene72473\_TAEpaeTARAAPEI-5, Unigene72569\_TAEpaeTARAAPEI-5, Unigene72588\_TAEpaeTARAAPEI-5, Unigene72591\_TAEpaeTARAAPEI-5, Unigene72635\_TAEpaeTARAAPEI-5, Unigene72690\_TAEpaeTARAAPEI-5, Unigene72702\_TAEpaeTARAAPEI-5, Unigene72707\_TAEpaeTARAAPEI-5, Unigene72732\_TAEpaeTARAAPEI-5, Unigene72803\_TAEpaeTARAAPEI-5, Unigene72823\_TAEpaeTARAAPEI-5, Unigene72839\_TAEpaeTARAAPEI-5, Unigene72849\_TAEpaeTARAAPEI-5, Unigene72875\_TAEpaeTARAAPEI-5, Unigene72877\_TAEpaeTARAAPEI-5, Unigene72896\_TAEpaeTARAAPEI-5, Unigene72901\_TAEpaeTARAAPEI-5, Unigene72903\_TAEpaeTARAAPEI-5, Unigene7406\_TAEpaeTARAAPEI-5, Unigene8411\_TAEpaeTARAAPEI-5, Unigene8505\_TAEpaeTARAAPEI-5, Unigene8876\_TAEpaeTARAAPEI-5, Unigene9304\_TAEpaeTARAAPEI-5, Unigene9365\_TAEpaeTARAAPEI-5, Unigene9391\_TAEpaeTARAAPEI-5, Unigene9502\_TAEpaeTARAAPEI-5, Unigene9723\_TAEpaeTARAAPEI-5, Unigene9731\_TAEpaeTARAAPEI-5 |
| 20 | Pathogenic Escherichia coli infection | Unigene10250\_TAEpaeTARAAPEI-5, Unigene10547\_TAEpaeTARAAPEI-5, Unigene10616\_TAEpaeTARAAPEI-5, Unigene10688\_TAEpaeTARAAPEI-5, Unigene10707\_TAEpaeTARAAPEI-5, Unigene11053\_TAEpaeTARAAPEI-5, Unigene11205\_TAEpaeTARAAPEI-5, Unigene11534\_TAEpaeTARAAPEI-5, Unigene11772\_TAEpaeTARAAPEI-5, Unigene12362\_TAEpaeTARAAPEI-5, Unigene12947\_TAEpaeTARAAPEI-5, Unigene13091\_TAEpaeTARAAPEI-5, Unigene13159\_TAEpaeTARAAPEI-5, Unigene13194\_TAEpaeTARAAPEI-5, Unigene13629\_TAEpaeTARAAPEI-5, Unigene13787\_TAEpaeTARAAPEI-5, Unigene13807\_TAEpaeTARAAPEI-5, Unigene13859\_TAEpaeTARAAPEI-5, Unigene14218\_TAEpaeTARAAPEI-5, Unigene14719\_TAEpaeTARAAPEI-5, Unigene14720\_TAEpaeTARAAPEI-5, Unigene14809\_TAEpaeTARAAPEI-5, Unigene15053\_TAEpaeTARAAPEI-5, Unigene15153\_TAEpaeTARAAPEI-5, Unigene15547\_TAEpaeTARAAPEI-5, Unigene15676\_TAEpaeTARAAPEI-5, Unigene1570\_TAEpaeTARAAPEI-5, Unigene15985\_TAEpaeTARAAPEI-5, Unigene16103\_TAEpaeTARAAPEI-5, Unigene16341\_TAEpaeTARAAPEI-5, Unigene16418\_TAEpaeTARAAPEI-5, Unigene16468\_TAEpaeTARAAPEI-5, Unigene17034\_TAEpaeTARAAPEI-5, Unigene17145\_TAEpaeTARAAPEI-5, Unigene17271\_TAEpaeTARAAPEI-5, Unigene17551\_TAEpaeTARAAPEI-5, Unigene17614\_TAEpaeTARAAPEI-5, Unigene17657\_TAEpaeTARAAPEI-5, Unigene17673\_TAEpaeTARAAPEI-5, Unigene17700\_TAEpaeTARAAPEI-5, Unigene17840\_TAEpaeTARAAPEI-5, Unigene17912\_TAEpaeTARAAPEI-5, Unigene19465\_TAEpaeTARAAPEI-5, Unigene19476\_TAEpaeTARAAPEI-5, Unigene19552\_TAEpaeTARAAPEI-5, Unigene20000\_TAEpaeTARAAPEI-5, Unigene20565\_TAEpaeTARAAPEI-5, Unigene20573\_TAEpaeTARAAPEI-5, Unigene21513\_TAEpaeTARAAPEI-5, Unigene2206\_TAEpaeTARAAPEI-5, Unigene22165\_TAEpaeTARAAPEI-5, Unigene22358\_TAEpaeTARAAPEI-5, Unigene23108\_TAEpaeTARAAPEI-5, Unigene23498\_TAEpaeTARAAPEI-5, Unigene24012\_TAEpaeTARAAPEI-5, Unigene24082\_TAEpaeTARAAPEI-5, Unigene2429\_TAEpaeTARAAPEI-5, Unigene24302\_TAEpaeTARAAPEI-5, Unigene2439\_TAEpaeTARAAPEI-5, Unigene24530\_TAEpaeTARAAPEI-5, Unigene24538\_TAEpaeTARAAPEI-5, Unigene25121\_TAEpaeTARAAPEI-5, Unigene25540\_TAEpaeTARAAPEI-5, Unigene26359\_TAEpaeTARAAPEI-5, Unigene27154\_TAEpaeTARAAPEI-5, Unigene2783\_TAEpaeTARAAPEI-5, Unigene27972\_TAEpaeTARAAPEI-5, Unigene28094\_TAEpaeTARAAPEI-5, Unigene2889\_TAEpaeTARAAPEI-5, Unigene29377\_TAEpaeTARAAPEI-5, Unigene29539\_TAEpaeTARAAPEI-5, Unigene2955\_TAEpaeTARAAPEI-5, Unigene29936\_TAEpaeTARAAPEI-5, Unigene30199\_TAEpaeTARAAPEI-5, Unigene30997\_TAEpaeTARAAPEI-5, Unigene31137\_TAEpaeTARAAPEI-5, Unigene32212\_TAEpaeTARAAPEI-5, Unigene32308\_TAEpaeTARAAPEI-5, Unigene32545\_TAEpaeTARAAPEI-5, Unigene32999\_TAEpaeTARAAPEI-5, Unigene33030\_TAEpaeTARAAPEI-5, Unigene33057\_TAEpaeTARAAPEI-5, Unigene33174\_TAEpaeTARAAPEI-5, Unigene33367\_TAEpaeTARAAPEI-5, Unigene33402\_TAEpaeTARAAPEI-5, Unigene33844\_TAEpaeTARAAPEI-5, Unigene33880\_TAEpaeTARAAPEI-5, Unigene3505\_TAEpaeTARAAPEI-5, Unigene35171\_TAEpaeTARAAPEI-5, Unigene35473\_TAEpaeTARAAPEI-5, Unigene35478\_TAEpaeTARAAPEI-5, Unigene35721\_TAEpaeTARAAPEI-5, Unigene36648\_TAEpaeTARAAPEI-5, Unigene37020\_TAEpaeTARAAPEI-5, Unigene37038\_TAEpaeTARAAPEI-5, Unigene37286\_TAEpaeTARAAPEI-5, Unigene37328\_TAEpaeTARAAPEI-5, Unigene37834\_TAEpaeTARAAPEI-5, Unigene37980\_TAEpaeTARAAPEI-5, Unigene38258\_TAEpaeTARAAPEI-5, Unigene38420\_TAEpaeTARAAPEI-5, Unigene3888\_TAEpaeTARAAPEI-5, Unigene39227\_TAEpaeTARAAPEI-5, Unigene39246\_TAEpaeTARAAPEI-5, Unigene39328\_TAEpaeTARAAPEI-5, Unigene39926\_TAEpaeTARAAPEI-5, Unigene40008\_TAEpaeTARAAPEI-5, Unigene40662\_TAEpaeTARAAPEI-5, Unigene40763\_TAEpaeTARAAPEI-5, Unigene40775\_TAEpaeTARAAPEI-5, Unigene40826\_TAEpaeTARAAPEI-5, Unigene41472\_TAEpaeTARAAPEI-5, Unigene4154\_TAEpaeTARAAPEI-5, Unigene41673\_TAEpaeTARAAPEI-5, Unigene41713\_TAEpaeTARAAPEI-5, Unigene41864\_TAEpaeTARAAPEI-5, Unigene42790\_TAEpaeTARAAPEI-5, Unigene43003\_TAEpaeTARAAPEI-5, Unigene43076\_TAEpaeTARAAPEI-5, Unigene43326\_TAEpaeTARAAPEI-5, Unigene4380\_TAEpaeTARAAPEI-5, Unigene43870\_TAEpaeTARAAPEI-5, Unigene44401\_TAEpaeTARAAPEI-5, Unigene4451\_TAEpaeTARAAPEI-5, Unigene44963\_TAEpaeTARAAPEI-5, Unigene45049\_TAEpaeTARAAPEI-5, Unigene45121\_TAEpaeTARAAPEI-5, Unigene45803\_TAEpaeTARAAPEI-5, Unigene45824\_TAEpaeTARAAPEI-5, Unigene4605\_TAEpaeTARAAPEI-5, Unigene46336\_TAEpaeTARAAPEI-5, Unigene46337\_TAEpaeTARAAPEI-5, Unigene46884\_TAEpaeTARAAPEI-5, Unigene47023\_TAEpaeTARAAPEI-5, Unigene47055\_TAEpaeTARAAPEI-5, Unigene47449\_TAEpaeTARAAPEI-5, Unigene47595\_TAEpaeTARAAPEI-5, Unigene47690\_TAEpaeTARAAPEI-5, Unigene48260\_TAEpaeTARAAPEI-5, Unigene48353\_TAEpaeTARAAPEI-5, Unigene48394\_TAEpaeTARAAPEI-5, Unigene48705\_TAEpaeTARAAPEI-5, Unigene4903\_TAEpaeTARAAPEI-5, Unigene49193\_TAEpaeTARAAPEI-5, Unigene49966\_TAEpaeTARAAPEI-5, Unigene50024\_TAEpaeTARAAPEI-5, Unigene50545\_TAEpaeTARAAPEI-5, Unigene51078\_TAEpaeTARAAPEI-5, Unigene51588\_TAEpaeTARAAPEI-5, Unigene51704\_TAEpaeTARAAPEI-5, Unigene51722\_TAEpaeTARAAPEI-5, Unigene51903\_TAEpaeTARAAPEI-5, Unigene51982\_TAEpaeTARAAPEI-5, Unigene52030\_TAEpaeTARAAPEI-5, Unigene52306\_TAEpaeTARAAPEI-5, Unigene52333\_TAEpaeTARAAPEI-5, Unigene52445\_TAEpaeTARAAPEI-5, Unigene5248\_TAEpaeTARAAPEI-5, Unigene52542\_TAEpaeTARAAPEI-5, Unigene52940\_TAEpaeTARAAPEI-5, Unigene53188\_TAEpaeTARAAPEI-5, Unigene53430\_TAEpaeTARAAPEI-5, Unigene53756\_TAEpaeTARAAPEI-5, Unigene53776\_TAEpaeTARAAPEI-5, Unigene53786\_TAEpaeTARAAPEI-5, Unigene54161\_TAEpaeTARAAPEI-5, Unigene54206\_TAEpaeTARAAPEI-5, Unigene54363\_TAEpaeTARAAPEI-5, Unigene54619\_TAEpaeTARAAPEI-5, Unigene54912\_TAEpaeTARAAPEI-5, Unigene55145\_TAEpaeTARAAPEI-5, Unigene55561\_TAEpaeTARAAPEI-5, Unigene55783\_TAEpaeTARAAPEI-5, Unigene56110\_TAEpaeTARAAPEI-5, Unigene56576\_TAEpaeTARAAPEI-5, Unigene56728\_TAEpaeTARAAPEI-5, Unigene56943\_TAEpaeTARAAPEI-5, Unigene56995\_TAEpaeTARAAPEI-5, Unigene57076\_TAEpaeTARAAPEI-5, Unigene57203\_TAEpaeTARAAPEI-5, Unigene57223\_TAEpaeTARAAPEI-5, Unigene57492\_TAEpaeTARAAPEI-5, Unigene57670\_TAEpaeTARAAPEI-5, Unigene57969\_TAEpaeTARAAPEI-5, Unigene58414\_TAEpaeTARAAPEI-5, Unigene58467\_TAEpaeTARAAPEI-5, Unigene58739\_TAEpaeTARAAPEI-5, Unigene58837\_TAEpaeTARAAPEI-5, Unigene589\_TAEpaeTARAAPEI-5, Unigene59088\_TAEpaeTARAAPEI-5, Unigene59225\_TAEpaeTARAAPEI-5, Unigene59328\_TAEpaeTARAAPEI-5, Unigene59795\_TAEpaeTARAAPEI-5, Unigene60131\_TAEpaeTARAAPEI-5, Unigene60165\_TAEpaeTARAAPEI-5, Unigene60289\_TAEpaeTARAAPEI-5, Unigene60606\_TAEpaeTARAAPEI-5, Unigene60837\_TAEpaeTARAAPEI-5, Unigene60871\_TAEpaeTARAAPEI-5, Unigene61029\_TAEpaeTARAAPEI-5, Unigene61772\_TAEpaeTARAAPEI-5, Unigene61853\_TAEpaeTARAAPEI-5, Unigene61989\_TAEpaeTARAAPEI-5, Unigene62090\_TAEpaeTARAAPEI-5, Unigene62263\_TAEpaeTARAAPEI-5, Unigene62275\_TAEpaeTARAAPEI-5, Unigene62504\_TAEpaeTARAAPEI-5, Unigene6260\_TAEpaeTARAAPEI-5, Unigene62859\_TAEpaeTARAAPEI-5, Unigene62920\_TAEpaeTARAAPEI-5, Unigene63299\_TAEpaeTARAAPEI-5, Unigene63370\_TAEpaeTARAAPEI-5, Unigene63472\_TAEpaeTARAAPEI-5, Unigene6350\_TAEpaeTARAAPEI-5, Unigene63565\_TAEpaeTARAAPEI-5, Unigene63590\_TAEpaeTARAAPEI-5, Unigene63731\_TAEpaeTARAAPEI-5, Unigene63956\_TAEpaeTARAAPEI-5, Unigene64114\_TAEpaeTARAAPEI-5, Unigene64199\_TAEpaeTARAAPEI-5, Unigene64260\_TAEpaeTARAAPEI-5, Unigene64293\_TAEpaeTARAAPEI-5, Unigene64601\_TAEpaeTARAAPEI-5, Unigene64602\_TAEpaeTARAAPEI-5, Unigene64687\_TAEpaeTARAAPEI-5, Unigene65120\_TAEpaeTARAAPEI-5, Unigene65318\_TAEpaeTARAAPEI-5, Unigene65636\_TAEpaeTARAAPEI-5, Unigene65658\_TAEpaeTARAAPEI-5, Unigene65717\_TAEpaeTARAAPEI-5, Unigene65950\_TAEpaeTARAAPEI-5, Unigene65955\_TAEpaeTARAAPEI-5, Unigene6595\_TAEpaeTARAAPEI-5, Unigene66167\_TAEpaeTARAAPEI-5, Unigene66209\_TAEpaeTARAAPEI-5, Unigene66212\_TAEpaeTARAAPEI-5, Unigene66291\_TAEpaeTARAAPEI-5, Unigene66309\_TAEpaeTARAAPEI-5, Unigene66318\_TAEpaeTARAAPEI-5, Unigene66440\_TAEpaeTARAAPEI-5, Unigene66464\_TAEpaeTARAAPEI-5, Unigene66665\_TAEpaeTARAAPEI-5, Unigene66907\_TAEpaeTARAAPEI-5, Unigene67019\_TAEpaeTARAAPEI-5, Unigene67341\_TAEpaeTARAAPEI-5, Unigene67405\_TAEpaeTARAAPEI-5, Unigene6745\_TAEpaeTARAAPEI-5, Unigene67497\_TAEpaeTARAAPEI-5, Unigene67524\_TAEpaeTARAAPEI-5, Unigene67641\_TAEpaeTARAAPEI-5, Unigene67748\_TAEpaeTARAAPEI-5, Unigene67781\_TAEpaeTARAAPEI-5, Unigene67962\_TAEpaeTARAAPEI-5, Unigene68085\_TAEpaeTARAAPEI-5, Unigene68096\_TAEpaeTARAAPEI-5, Unigene68103\_TAEpaeTARAAPEI-5, Unigene68274\_TAEpaeTARAAPEI-5, Unigene68980\_TAEpaeTARAAPEI-5, Unigene69008\_TAEpaeTARAAPEI-5, Unigene69009\_TAEpaeTARAAPEI-5, Unigene69101\_TAEpaeTARAAPEI-5, Unigene69314\_TAEpaeTARAAPEI-5, Unigene69370\_TAEpaeTARAAPEI-5, Unigene6938\_TAEpaeTARAAPEI-5, Unigene6940\_TAEpaeTARAAPEI-5, Unigene69470\_TAEpaeTARAAPEI-5, Unigene69623\_TAEpaeTARAAPEI-5, Unigene69636\_TAEpaeTARAAPEI-5, Unigene69775\_TAEpaeTARAAPEI-5, Unigene69908\_TAEpaeTARAAPEI-5, Unigene69925\_TAEpaeTARAAPEI-5, Unigene70047\_TAEpaeTARAAPEI-5, Unigene70074\_TAEpaeTARAAPEI-5, Unigene70089\_TAEpaeTARAAPEI-5, Unigene70104\_TAEpaeTARAAPEI-5, Unigene70149\_TAEpaeTARAAPEI-5, Unigene70154\_TAEpaeTARAAPEI-5, Unigene70206\_TAEpaeTARAAPEI-5, Unigene70309\_TAEpaeTARAAPEI-5, Unigene70316\_TAEpaeTARAAPEI-5, Unigene70319\_TAEpaeTARAAPEI-5, Unigene70435\_TAEpaeTARAAPEI-5, Unigene70524\_TAEpaeTARAAPEI-5, Unigene70533\_TAEpaeTARAAPEI-5, Unigene70536\_TAEpaeTARAAPEI-5, Unigene70559\_TAEpaeTARAAPEI-5, Unigene70615\_TAEpaeTARAAPEI-5, Unigene70659\_TAEpaeTARAAPEI-5, Unigene70663\_TAEpaeTARAAPEI-5, Unigene70904\_TAEpaeTARAAPEI-5, Unigene70941\_TAEpaeTARAAPEI-5, Unigene71142\_TAEpaeTARAAPEI-5, Unigene71246\_TAEpaeTARAAPEI-5, Unigene71271\_TAEpaeTARAAPEI-5, Unigene71272\_TAEpaeTARAAPEI-5, Unigene71420\_TAEpaeTARAAPEI-5, Unigene71553\_TAEpaeTARAAPEI-5, Unigene71562\_TAEpaeTARAAPEI-5, Unigene71617\_TAEpaeTARAAPEI-5, Unigene71621\_TAEpaeTARAAPEI-5, Unigene71627\_TAEpaeTARAAPEI-5, Unigene71723\_TAEpaeTARAAPEI-5, Unigene71760\_TAEpaeTARAAPEI-5, Unigene71839\_TAEpaeTARAAPEI-5, Unigene71869\_TAEpaeTARAAPEI-5, Unigene72042\_TAEpaeTARAAPEI-5, Unigene72113\_TAEpaeTARAAPEI-5, Unigene72141\_TAEpaeTARAAPEI-5, Unigene7227\_TAEpaeTARAAPEI-5, Unigene72322\_TAEpaeTARAAPEI-5, Unigene72325\_TAEpaeTARAAPEI-5, Unigene72337\_TAEpaeTARAAPEI-5, Unigene72340\_TAEpaeTARAAPEI-5, Unigene72362\_TAEpaeTARAAPEI-5, Unigene72442\_TAEpaeTARAAPEI-5, Unigene72452\_TAEpaeTARAAPEI-5, Unigene72473\_TAEpaeTARAAPEI-5, Unigene72497\_TAEpaeTARAAPEI-5, Unigene72519\_TAEpaeTARAAPEI-5, Unigene72540\_TAEpaeTARAAPEI-5, Unigene72647\_TAEpaeTARAAPEI-5, Unigene72655\_TAEpaeTARAAPEI-5, Unigene72670\_TAEpaeTARAAPEI-5, Unigene72689\_TAEpaeTARAAPEI-5, Unigene72711\_TAEpaeTARAAPEI-5, Unigene72730\_TAEpaeTARAAPEI-5, Unigene72750\_TAEpaeTARAAPEI-5, Unigene72924\_TAEpaeTARAAPEI-5, Unigene7309\_TAEpaeTARAAPEI-5, Unigene7399\_TAEpaeTARAAPEI-5, Unigene7844\_TAEpaeTARAAPEI-5, Unigene7994\_TAEpaeTARAAPEI-5, Unigene8692\_TAEpaeTARAAPEI-5, Unigene8876\_TAEpaeTARAAPEI-5, Unigene907\_TAEpaeTARAAPEI-5, Unigene9304\_TAEpaeTARAAPEI-5, Unigene9443\_TAEpaeTARAAPEI-5, Unigene9723\_TAEpaeTARAAPEI-5 |
[truncated: 614,640 more chars]
