# Supplementary figures and images for: Annotation of the Transcriptome from Taenia pisiformis and Its Comparative Analysis with Three Taeniidae Species
Source: PLoS One. 2012 Apr 13;7(4):e32283. doi: 10.1371/journal.pone.0032283 (PMC3326008; doi:10.1371/journal.pone.0032283)

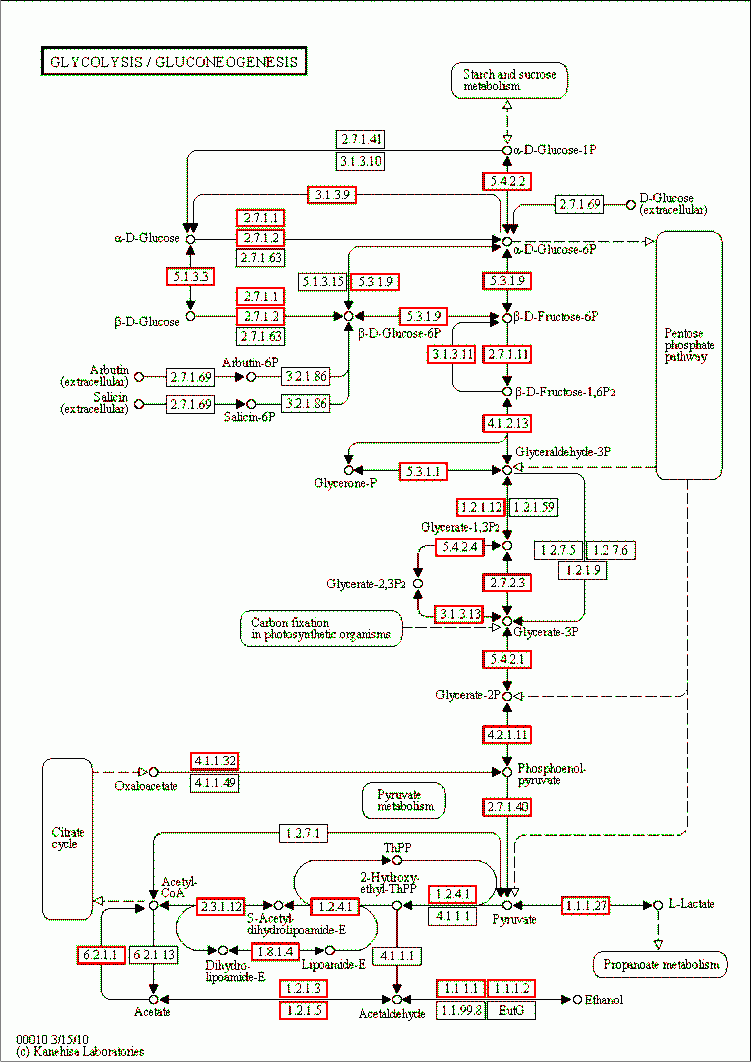


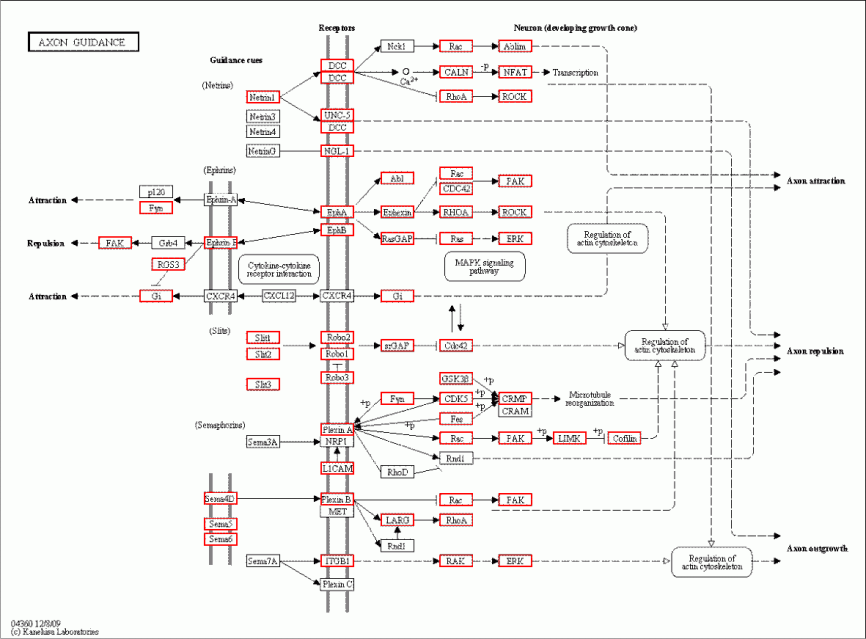

Supplement: Dataset S7 — The glycolysis/gluconeogenesis and axon guidance pathway of Taenia pisiformis . There were three key enzymes in glycolysis/gluconeogenesis: hexokinase (EC 2.7.1.1), phosphofructokinase (EC 4.1.2.13), and pyruvate kinase (EC 2.7.1.40). Additionally, there were four highly conservative axon guidance molecular families, netrins (netrin 1), slits (slits 1, slits 2, and slits 3), semaphorins (sema4D, sema5, and sema6), and ephrins (ephrin E). (DOCX) [file pone.0032283.s008.docx]
