# Supplementary material for: Annotation of the Transcriptome from Taenia pisiformis and Its Comparative Analysis with Three Taeniidae Species
Source: PLoS One. 2012 Apr 13;7(4):e32283. doi: 10.1371/journal.pone.0032283 (PMC3326008; doi:10.1371/journal.pone.0032283)
Supplement: Dataset S12 — 21 KEGG pathways of Taenia pisiformis , Taenia solium , Echinococcus granulosis and Echinococcus multilocularis . M was aligned in the KEGG database using BLASTx, and 109 genes obtained 21 pathways. (HTM) [file pone.0032283.s013.htm]

M.fa

1. M.fa

| # | Pathway | Count (109) | Pathway ID |
| 1 | Amoebiasis | 20 | ko05146 |
| 2 | Vibrio cholerae infection | 20 | ko05110 |
| 3 | Dorso-ventral axis formation | 18 | ko04320 |
| 4 | Salivary secretion | 14 | ko04970 |
| 5 | Regulation of actin cytoskeleton | 9 | ko04810 |
| 6 | Pathogenic Escherichia coli infection | 4 | ko05130 |
| 7 | Spliceosome | 4 | ko03040 |
| 8 | Shigellosis | 4 | ko05131 |
| 9 | Fc gamma R-mediated phagocytosis | 3 | ko04666 |
| 10 | Chemokine signaling pathway | 3 | ko04062 |
| 11 | Bacterial invasion of epithelial cells | 3 | ko05100 |
| 12 | Adherens junction | 3 | ko04520 |
| 13 | Basal cell carcinoma | 2 | ko05217 |
| 14 | Wnt signaling pathway | 2 | ko04310 |
| 15 | Systemic lupus erythematosus | 2 | ko05322 |
| 16 | Endometrial cancer | 2 | ko05213 |
| 17 | Pathways in cancer | 2 | ko05200 |
| 18 | Colorectal cancer | 2 | ko05210 |
| 19 | Focal adhesion | 1 | ko04510 |
| 20 | MAPK signaling pathway | 1 | ko04010 |
| 21 | RNA degradation | 1 | ko03018 |

| # | Pathway | Differentially expressed genes |
| --- | --- | --- |
| 1 | Amoebiasis | EGCWgr-12b09.p1k, EGCWgr-12b09.q1k, EGCWgr-12f05.q1k, EGCWgr-13d03.q1k, EGCWgr-13d04.q1k, EGCWgr-13d05.p1k, EGCWgr-13d05.q1k, EGCWgr-13e02.q1k, EGCWgr-13f05.q1k, EGCWgr-13f06.q1k, EGCWgr-13f07.q1k, EGCWgr-13f08.p1k, EGCWgr-13f08.q1k, EGCWgr-14e07.q1k, EGCWgr-14f09.q1k, EGCWgr-14f12.q1k, EGCWgr-14g11.q1k, EGCWgr-3b08.q1k, EGCWgr-7a07.q1k, EGCWgr-7h12.q1k |
| 2 | Vibrio cholerae infection | EGCWgr-12b09.p1k, EGCWgr-12b09.q1k, EGCWgr-12f05.q1k, EGCWgr-13d03.q1k, EGCWgr-13d04.q1k, EGCWgr-13d05.p1k, EGCWgr-13d05.q1k, EGCWgr-13e02.q1k, EGCWgr-13f05.q1k, EGCWgr-13f06.q1k, EGCWgr-13f07.q1k, EGCWgr-13f08.p1k, EGCWgr-13f08.q1k, EGCWgr-14e07.q1k, EGCWgr-14f09.q1k, EGCWgr-14f12.q1k, EGCWgr-14g11.q1k, EGCWgr-3b08.q1k, EGCWgr-7a07.q1k, EGCWgr-7h12.q1k |
| 3 | Dorso-ventral axis formation | EGPSPsl-10h10.p1k, EGPSPsl-11b11.p1k, EGPSPsl-13d11.p1k, EGPSgr-13d05.p1k, EGPSsl-2b10.p1k, EGPSsl-5f11.p1k, gi|187222562|gb|EX150493.1|EX150493, gi|187232850|gb|EX151028.1|EX151028, gi|187232853|gb|EX151031.1|EX151031, gi|187232854|gb|EX151032.1|EX151032, gi|187232855|gb|EX151033.1|EX151033, gi|187233310|gb|EX151026.1|EX151026, gi|187233311|gb|EX151027.1|EX151027, gi|187233314|gb|EX151037.1|EX151037, gi|187233315|gb|EX151038.1|EX151038, gi|187233316|gb|EX151039.1|EX151039, gi|187233317|gb|EX151040.1|EX151040, gi|187233342|gb|EX151086.1|EX151086 |
| 4 | Salivary secretion | EGCWgr-9a06.p1k, EGPSPgr-8g08.p1k, EGPSPsl-10c01.p1k, EGPSPsl-12c02.p1k, EGPSPsl-13b08.p1k, EGPSPsl-17e06.p1k, EGPSPsl-1c04.p1k, EGPSPsl-4b09.p1k, EGPSPsl-4d08.p1k, EGPSsl-10a10.p1k, EGPSsl-1b01.p1k, EGPSsl-3e03.p1k, EGPSsl-4a04.p1k, EGPSsl-8d06.p1k |
| 5 | Regulation of actin cytoskeleton | EGCWgr-12b04.p1k, EGCWgr-5c03.p1k, EGPSPgr-11d03.q1k, EGPSPgr-5h11.p1k, EGPSPsl-12d09.p1k, EGPSPsl-16h01.p1k, EGPSgr-14a05.q1k, EGPSgr-7b07.p1k, EGPSsl-5f07.p1k |
| 6 | Pathogenic Escherichia coli infection | EGCWgr-5c03.p1k, EGPSPgr-11d03.q1k, EGPSPsl-16h01.p1k, gi|129293873|gb|EL750653.1|EL750653 |
| 7 | Spliceosome | EGPSPgr-11d03.q1k, EGPSPgr-6h01.p1k, EGPSsl-2b10.p1k, EGPSsl-2c12.p1k |
| 8 | Shigellosis | EGCWgr-5c03.p1k, EGPSPgr-11d03.q1k, EGPSPsl-12d09.p1k, EGPSPsl-16h01.p1k |
| 9 | Fc gamma R-mediated phagocytosis | EGCWgr-5c03.p1k, EGPSPgr-11d03.q1k, EGPSPsl-16h01.p1k |
| 10 | Chemokine signaling pathway | EGCWgr-5c03.p1k, EGPSPgr-11d03.q1k, EGPSPsl-16h01.p1k |
| 11 | Bacterial invasion of epithelial cells | EGCWgr-5c03.p1k, EGPSPgr-11d03.q1k, EGPSPsl-16h01.p1k |
| 12 | Adherens junction | EGCWgr-5c03.p1k, EGPSPgr-11d03.q1k, EGPSPsl-16h01.p1k |
| 13 | Basal cell carcinoma | EGCWgr-12b04.p1k, EGPSgr-14a05.q1k |
| 14 | Wnt signaling pathway | EGCWgr-12b04.p1k, EGPSgr-14a05.q1k |
| 15 | Systemic lupus erythematosus | EGPSPgr-11h10.q1k, EGPSPgr-12a05.q1k |
| 16 | Endometrial cancer | EGCWgr-12b04.p1k, EGPSgr-14a05.q1k |
| 17 | Pathways in cancer | EGCWgr-12b04.p1k, EGPSgr-14a05.q1k |
| 18 | Colorectal cancer | EGCWgr-12b04.p1k, EGPSgr-14a05.q1k |
| 19 | Focal adhesion | EGPSPsl-12d09.p1k |
| 20 | MAPK signaling pathway | gi|129289086|gb|EL745894.1|EL745894 |
| 21 | RNA degradation | gi|187232751|gb|EX151062.1|EX151062 |
