# Supplementary material for: Annotation of the Transcriptome from Taenia pisiformis and Its Comparative Analysis with Three Taeniidae Species
Source: PLoS One. 2012 Apr 13;7(4):e32283. doi: 10.1371/journal.pone.0032283 (PMC3326008; doi:10.1371/journal.pone.0032283)
Supplement: Dataset S13 — The process of the SOAPdenovo assembly. (DOCX) [file pone.0032283.s014.docx]

SOAPdenovo first combined reads with a certain length of overlap to form longer fragments without N bases, named contigs. The reads were then mapped back to contigs; with paired-end reads it was able to detect contigs from the same transcript as well as the distances between these contigs. Next, SOAPdenovo connected the contigs using N bases to represent unknown sequences between each two contigs, and created scaffolds. Paired-end reads were used again for the gap-filling of scaffolds to obtain sequences with the fewest Ns that could not be extended on either end. Such sequences were defined as unigenes. When multiple samples from the same species were sequenced, sequence splicing and redundancy removing could further process unigenes from the assembly of each sample. This was carried out using sequence clustering software to acquire non-redundant unigenes as long as possible.
